# Functional equivalence of germ plasm organizers

**DOI:** 10.1371/journal.pgen.1007696

**Published:** 2018-11-06

**Authors:** Pritesh Krishnakumar, Stephan Riemer, Roshan Perera, Thomas Lingner, Alexander Goloborodko, Hazem Khalifa, Franck Bontems, Felix Kaufholz, Mohamed A. El-Brolosy, Roland Dosch

**Affiliations:** 1 Institute for Developmental Biochemistry, University Medical Center, Göttingen, Germany; 2 Laboratory of Metabolism, Department of Internal Medicine Specialties, Faculty of Medicine, University of Geneva, Switzerland; 3 Institute of Human Genetics, University Medical Center, Göttingen, Germany; Fred Hutchinson Cancer Research Center, UNITED STATES

## Abstract

The proteins Oskar (Osk) in *Drosophila* and Bucky ball (Buc) in zebrafish act as germ plasm organizers. Both proteins recapitulate germ plasm activities but seem to be unique to their animal groups. Here, we discover that Osk and Buc show similar activities during germ cell specification. *Drosophila* Osk induces additional PGCs in zebrafish. Surprisingly, Osk and Buc do not show homologous protein motifs that would explain their related function. Nonetheless, we detect that both proteins contain stretches of intrinsically disordered regions (IDRs), which seem to be involved in protein aggregation. IDRs are known to rapidly change their sequence during evolution, which might obscure biochemical interaction motifs. Indeed, we show that Buc binds to the known Oskar interactors Vasa protein and *nanos* mRNA indicating conserved biochemical activities. These data provide a molecular framework for two proteins with unrelated sequence but with equivalent function to assemble a conserved core-complex nucleating germ plasm.

## Author summary

Multicellular organisms use gametes for their propagation. Gametes are formed from germ cells, which are specified during embryogenesis in some animals by the inheritance of RNP granules known as germ plasm. Transplantation of germ plasm induces extra germ cells, whereas germ plasm ablation leads to the loss of gametes and sterility. Therefore, germ plasm is key for germ cell formation and reproduction. However, the molecular mechanisms of germ cell specification by germ plasm in the vertebrate embryo remain an unsolved question. Proteins, which assemble the germ plasm, are known as germ plasm organizers. Here, we show that the two germ plasm organizers Oskar from the fly and Bucky ball from the fish show similar functions by using a cross species approach. Both are intrinsically disordered proteins, which rapidly changed their sequence during evolution. Moreover, both proteins still interact with conserved components of the germ cell specification pathway. These data might provide a first example of two proteins with the same biological role, but distinct sequence.

## Introduction

Living systems have the unique ability to reproduce copies of themselves. In animals, the reproductive cells or their precursors, the primordial germ cells (PGCs) are specified by two different modes during embryogenesis [reviewed in 1]. In the inductive mode, the embryo generates signals, which specify a subset of cells to differentiate into PGCs. This was initially described for mouse and axolotl, which seems to be the ancestral mode [reviewed in 2]. In the alternative, maternal-inheritance mode, the mother deposits a cytoplasmic determinant termed germ plasm into the oocyte [reviewed in 3, 4]. After fertilization, germ plasm is inherited by a subset of embryonic cells, which then differentiate into PGCs as shown for example in *Drosophila*, *C*. *elegans*, *Xenopus*, and zebrafish. Ablation and transplantation experiments demonstrated that germ plasm is necessary and sufficient for PGC specification [reviewed in 4, 5, 6].

Germ plasm activities can be triggered by a single *Drosophila* protein termed Oskar (Osk) [reviewed in 7]. Osk mutants fail to assemble germ plasm [8, 9], whereas mis-localization of Osk induces ectopic PGCs [10, 11]. Structural and biochemical studies revealed that Osk binds RNA and more recently, that it increases the helicase activity of its interaction partner Vasa [12–15]. Despite its potent activity as a germ plasm organizer, Osk homologs were not discovered outside of insect genomes [reviewed in 16].

In vertebrates, we identified the zebrafish *bucky ball* (*buc*) gene, which appears similar at the genetic level to Osk in *Drosophila* [17]. Buc mutants fail to assemble germ plasm, whereas its overexpression induces ectopic PGCs [17, 18]. Biochemical studies with Buc suggest that it acts as a scaffold bringing together RNA binding proteins like Hermes [19–24]. Interesting results with the frog homolog Velo1 showed that its N-terminal prion-like domain forms SDS-resistant granules and that these amyloid-like aggregates recruit RNA [25, 26]. Similar to Osk, Buc is a fast-evolving protein, which has not been found outside of vertebrate genomes [17, 27]. It is therefore not surprising that no sequence similarity between Osk and Buc was previously discovered [28]. Nonetheless, the striking overlap in their function in *Drosophila* or zebrafish was frequently highlighted [e.g. 29], but experiments directly addressing the functional conservation of Osk and Buc are not available.

Here we provide a biochemical basis for the functional equivalence of both germ plasm organizers. We show that overexpression of *Drosophila* Osk leads to the formation of ectopic PGCs in zebrafish. Although Buc and Osk share this unique activity, we did not detect conserved motifs in extensive sequence comparisons. However, we find that both germ plasm organizers share protein stretches of intrinsically disordered regions (IDRs). Upon overexpression, we observe that Osk and Buc formed protein aggregates similar to liquid-liquid phase separations or hydrogels as previously shown for other IDPs [30, 31]. Moreover, when we treated early zebrafish embryos with hydrogel disruptors, we observe the fragmentation of Buc aggregates. IDRs change their sequence rapidly during evolution, which obscures conserved interaction motifs [32]. We indeed discover that known biochemical interactors of Osk, *i*.*e*. Vasa protein and *nanos* mRNA, also interact with Buc. These data show that the functional equivalence of germ plasm organizers is based on similar biochemical interactions and could represent the first case of an unrelated protein pair with hidden evolutionary homology.

## Results

### Oskar induces primordial germ cell formation in zebrafish

**Fig 1:**
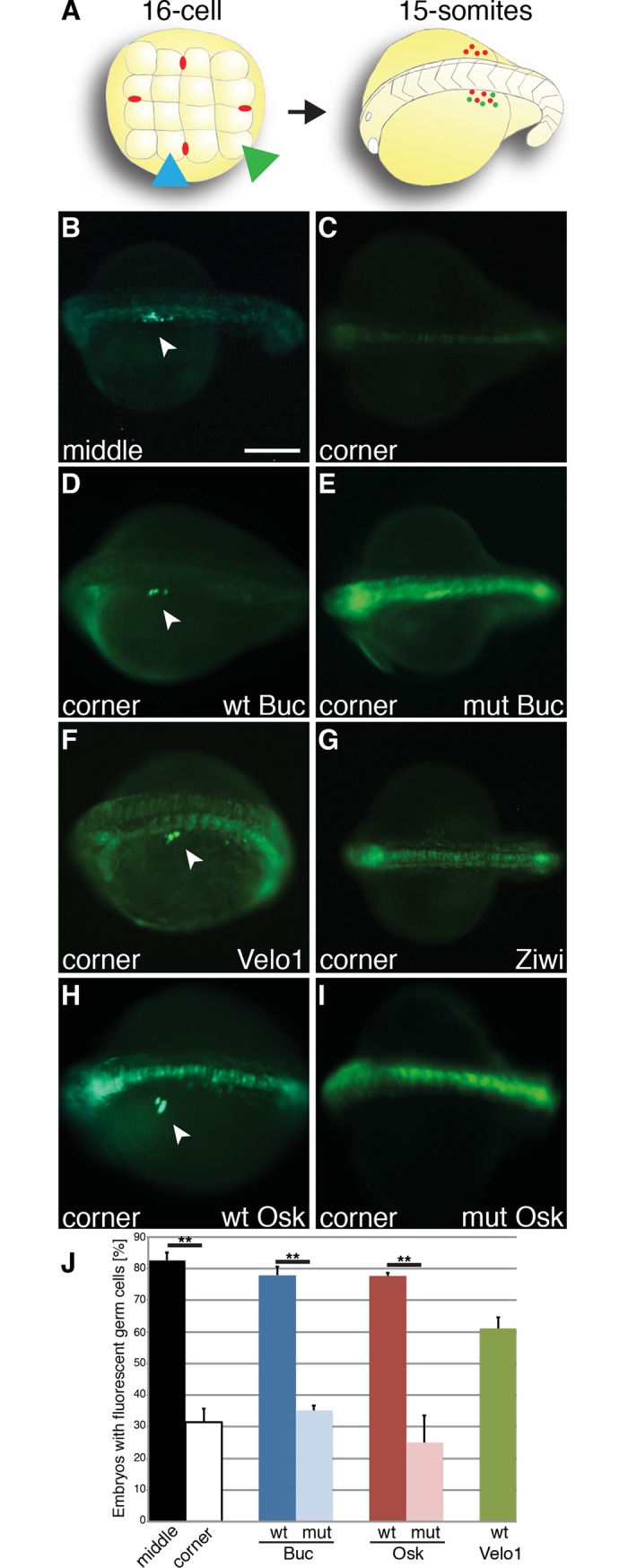
*Drosophila* Oskar specifies germ cell formation in zebrafish. (A) Scheme of germ cell induction assay. Left panel: Animal view of a 16-cell embryo injected with PGC-reporter into a middle blastomere (blue arrowhead) containing endogenous germ plasm (red dots) or into a somatic cell (corner blastomere; green arrowhead). Right panel: Oblique, dorsal view of a 15-somite stage embryo (18 hours post fertilization, hpf), anterior to the left. Fluorescent germ cells (white arrowhead) emerge by targeting the reporter to a PGC or transforming a somatic cell into a PGC. (B, C) Live 18 hpf embryo after injection of PGC-reporter into a middle (B; 83±2.4%; n = 70) or corner blastomere (C; 31±4.5%; n = 70; p = 0.005). As previously shown, the *gfp-nos3’UTR* reporter also frequently labeled the midline at this stage [33]. (D, E) Wild-type Buc (aa1-639) reprograms the somatic corner blastomere to the germline (D; wt = 78±2.6%; n = 71), but not mutant Buc (Buc^p43^). *Buc*^*p43*^ mRNA sequence is identical to wt, but carries a point mutation (Y362STOP) [17] (E; mut = 35±1.6%; n = 68; p = 0.001). (F, G) *Xenopus* Velo1 acts as a functional homolog (F; 61±3.5%; n = 41) but not zebrafish Ziwi (G). (H, I) *Drosophila* sOsk induces germ cell formation (H; wt = 78±1.1%; n = 81), but not mutant sOsk^084^ (aa139-254) (I; mut = 25±8.7%; n = 62; p = 0.01). Scale bar (B-I): 200 μm. (J) Quantification of injection results (three independent experiments for each RNA). Error bars represent standard deviation of the mean. Student’s t-test; P-value: **<0.01.

We first analyzed the functional equivalence of germ plasm proteins by analyzing their activity to reprogram somatic cells into PGCs. The germ cell induction assay exploits that the first somatic cells in zebrafish segregate from the germline at the 16-cell stage (Fig 1A) [17]. Injecting *gfp-nos3’UTR* reporter mRNA [33] into a middle blastomere containing endogenous germ plasm highlighted PGCs in 18 hours post fertilization (hpf) embryos (Fig 1B and 1J). By contrast, injection into a somatic cell (corner blastomere) leads to background activation of the PGC-reporter as previously published (Fig 1C and 1J) [17]. Co-injecting wild-type *buc* mRNA encoding amino acid 1–639 into somatic cells was sufficient to significantly increase PGC specification, but mutant *buc* mRNA coding for aa 1–361 was not different from negative controls (Fig 1D, 1E and 1J).

The *Xenopus* Velo1 protein was recently postulated to act as a functional homolog of Buc [25, 26]. To test this hypothesis experimentally, we overexpressed *Xenopus* Velo1 in zebrafish embryos and found ectopic germ cells (Fig 1F and 1J). To determine the specificity of the germ cell induction assay, we injected the zebrafish Piwi homolog Ziwi, which is also a germ plasm component [34]. However, Ziwi showed no activity confirming that not every germ plasm component is active in the germ cell induction assay (Fig 1G).

The germ plasm organizers Osk and Buc have a remarkable genetic similarity [reviewed in 6, 7, 35], but conserved sequences were not discovered [28]. To compare their function experimentally, we tested Osk in the germ cell induction assay. Fascinatingly, short Oskar (sOsk, aa 139–606), which is the active variant in *Drosophila* for specifying germ cells, also induced PGCs in zebrafish. By contrast, mutant sOsk (aa 139–254) showed no effect (Fig 1H–1J). The mutant controls are derived from the *osk*^*84*^ and the *buc*^*p43*^ alleles, which have identical RNA sequences to their wild-type counterparts besides a point mutation generating a premature STOP-codon [9, 17]. Thus, overexpression of *buc* or *osk* mRNA *per se* is not sufficient to induce germ cells. Injecting GFP-fusions of these mRNAs lead to fluorescent embryos suggesting that they are indeed translated into protein (S1A Fig). Quantification indicated a similar number of specified PGCs by Osk and Buc (S1B Fig). These results suggest that the germ plasm organizers sOsk, Buc and their homologs share the unique activity to specify germ cells.

**Fig 2:**
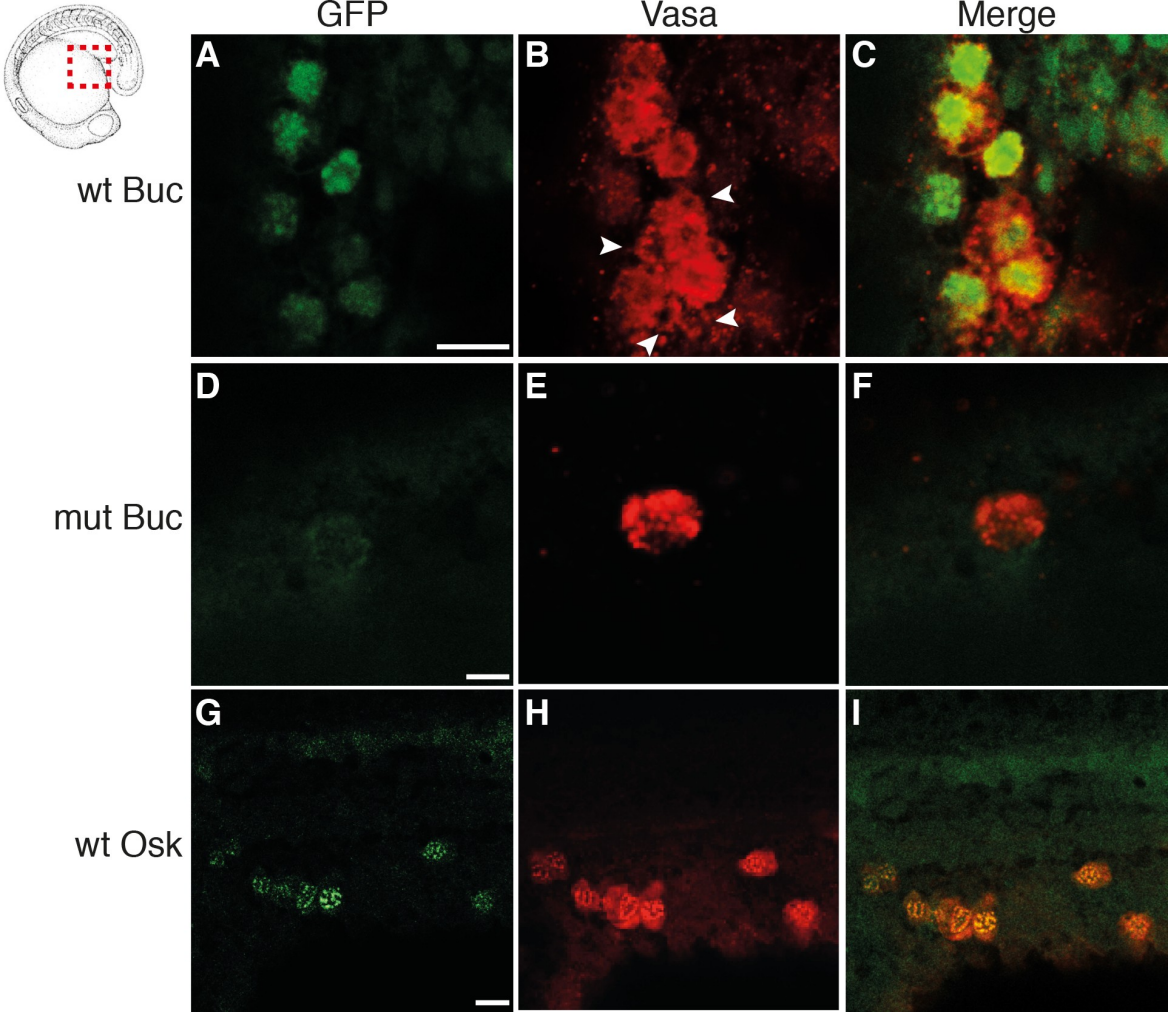
Buc and Osk induced PGCs express Vasa protein. Lateral view, anterior to the left of area indicated in icon of 18-somite stage embryo after 16-cell assay with wt Buc(1–639) (A-C), mutant Buc(1–361) (D-F) or sOsk (G-I). Embryos were analyzed for GFP (green) and Vasa (red) protein expression. Arrowheads indicate endogenous PGCs (Vasa positive and GFP negative). Scale bar: 20 μm.

Although these ectopic cells express the PGC reporter and migrate to the prospective gonad, it was recently shown that reprogrammed germ cells retain GFP fluorescence, while they differentiate into somatic tissue types of the three germ layers [36]. Similar to Buc [17], Osk induced an increased number of *vasa* mRNA positive cells at 3 hpf (S2 Fig). Moreover, we analyzed Vasa protein expression of cells expressing the PGC reporter in the 16-cell assay. Notably, GFP positive cells after Buc or Osk injection also expressed Vasa protein (Fig 2A–2C and 2G–2I). By contrast, embryos injected with mutant Buc (1–361) only stained the endogenous germ cells with Vasa, but ectopic GFP-positive germ cells were not detected (Fig 2D–2F). Taken together, we concluded that the cells specified by Buc and Osk differentiate into PGCs.

### Osk and Buc show no conserved sequence motif

**Fig 3:**
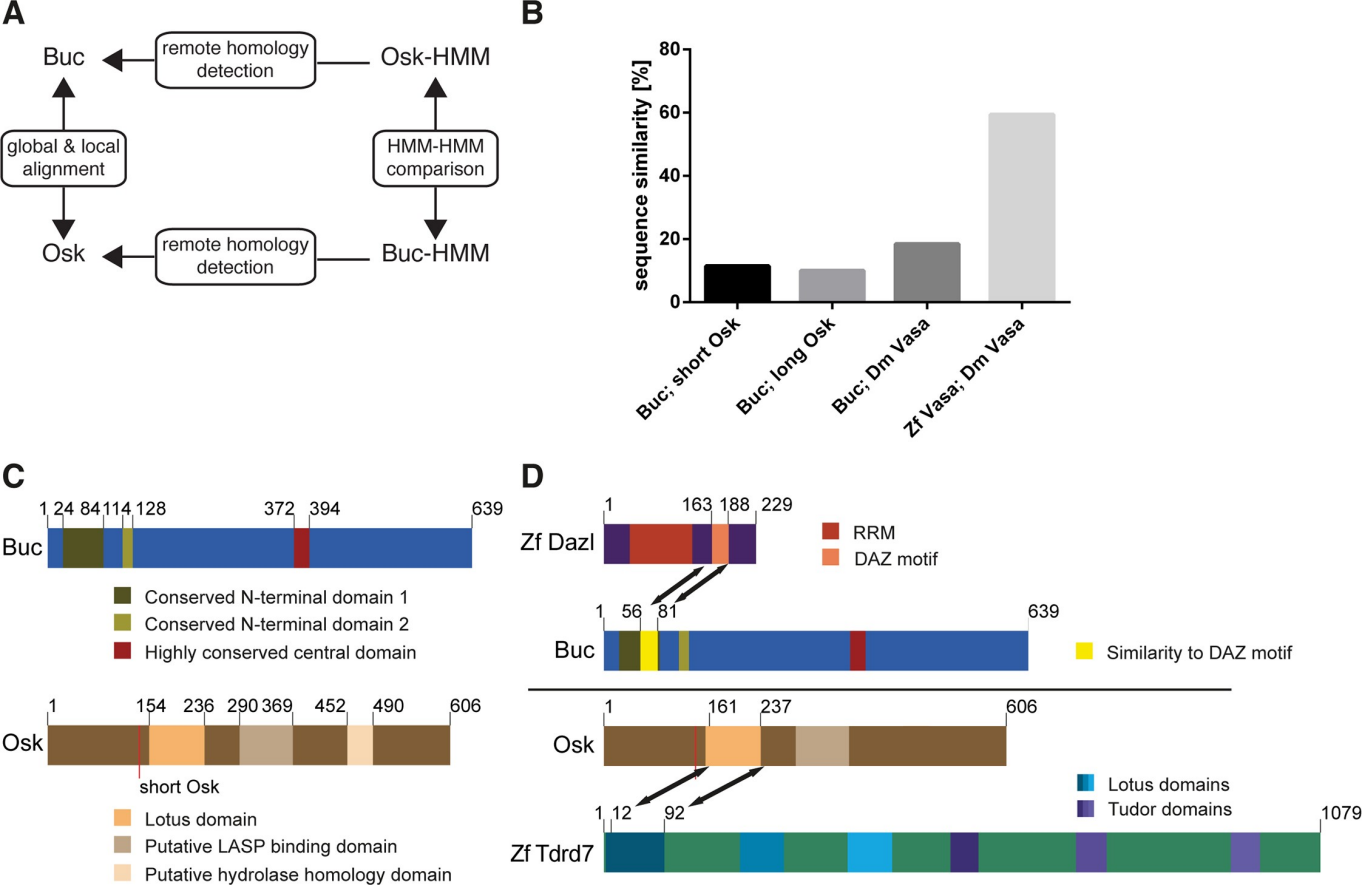
Drosophila Osk and zebrafish Buc display unrelated protein sequences. (A) Strategy for Osk-Buc sequence comparison using first global and local alignment algorithms, then hidden Markov models (HMM) of both proteins to detect remote homologies, and eventually a comparison of HMM to each other. (B) Graph comparing sequence similarity of Buc with short (aa139-606) and long Osk (aa1-606) isoforms based on global pairwise sequence alignments. Alignment of Buc and Drosophila Vasa (Dm Vasa) serves as negative, whereas zebrafish (Zf Vasa) and Dm Vasa as positive control. (C) Scheme of conserved domains in Buc identified by alignments of 14 protein sequences. Conserved motifs in Osk protein. Red line indicates alternative translation initiation of short Osk at Met139. (D) Scheme indicating significant remote sequence similarity based on HMMER analysis for Buc with zebrafish Dazl and for Osk with the Lotus domain of zebrafish Tdrd7. Numbers indicate amino acid positions.

According to the sequence-structure-function paradigm, proteins with the same activity contain homologous sequence motifs to interact with similar binding partners [reviewed in 37, 38]. Conserved amino acid sequences were previously described for *Xenopus* Velo and zebrafish Buc, but not between *Drosophila* Osk and Buc [17, 28]. We therefore pursued a stepwise strategy for their direct, bioinformatic comparison starting with pairwise alignments (Fig 3A). We detected only 11.5% similarity between both proteins (Fig 3B; S1 Table). The long Osk (lOsk) isoform, which is inactive in germ cell induction in *Drosophila* [39], reduced similarity to Buc even further down to 10%. A comparison of zebrafish Buc with *Drosophila* Vasa as an unrelated sequence showed 18.5% similarity, while Vasa homologs in zebrafish and *Drosophila* were 59.4% similar (Fig 3B).

In previous studies, the alignment of orthologs from different species revealed conserved domains and thereby hidden similarities [40, 41]. We aligned the sequences of 14 vertebrate Buc orthologs discovering two conserved motifs (aa 24–84 and 114–128) within the previously described BUVE-sequence (aa 23–136) [17] and another novel motif in the center of Buc (aa 372–394) (Fig 3C). The same approach with Osk detected published motifs: the LOTUS-domain (aa 154–236 lOsk) [42, 43], the Lasp binding region (aa 290–369) [44], and the putative hydrolase homology sequence within the OSK domain (aa 452–490) [12, 14, 45] (Fig 3C). We then generated profile hidden Markov models (HMM) of both proteins, but to our surprise did not detect significant hits by searching the *Drosophila* genome for sequence similarities with the Buc-HMM. The Buc-HMM consensus sequence, however, showed 43% identity of aa 56–81 in Buc to the DAZ motif in zebrafish Dazl (Fig 3D; S3 Table) [46]. Searching with Osk-HMM identified Tdrd5 and -7 in zebrafish and Tejas in *Drosophila*, which all contain LOTUS-motifs, but no similarity to Buc (Fig 3D; S3 Table). Finally, comparing the HMM-models of sOsk and Buc to each other did also not discover conserved motifs (S3 Table). Taken together, our extensive bioinformatic analysis did not detect hidden sequence similarities between the two germ plasm organizers Osk and Buc and hence, could not explain their similar activity.

### Osk and Buc encode intrinsically disordered proteins

**Fig 4:**
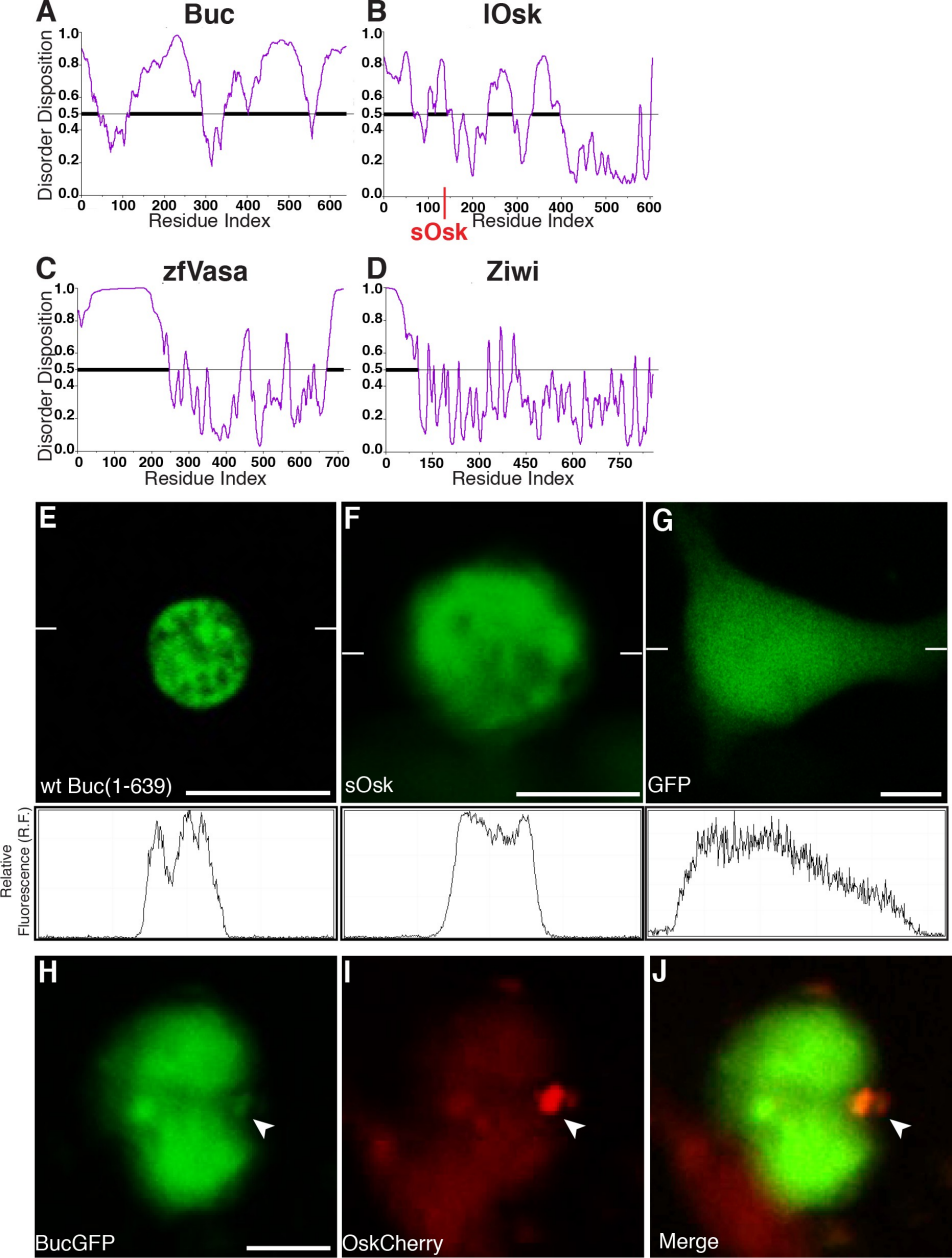
Buc and Osk contain intrinsically disordered regions. Predicted protein disorder in (A) Buc, (B) Osk, (C), Vasa, and (D) Ziwi. Disorder disposition (y-axis) plotted against the amino acid residue index (x-axis). Values above the 0.5 threshold (grey bar) show the propensity for disordered regions (bold line). The red line at aa 139 in Osk indicates the alternative translation initiation site for short Oskar. Protein aggregates upon transfection of HEK cells with monomeric GFP (mGFP) fused to (E) Buc, (F) sOsk or (G) unfused. The profiles below the pictures show levels of fluorescent intensity along the line indicated by white dashes. Buc-mGFP (green; H) and Osk-Cherry aggregates (red, I) overlap (J, yellow, white arrowhead). Scale bar (E-J): 10μm.

Intrinsically disordered proteins (IDPs) seem to be an exception to the conventional sequence-structure-function paradigm [reviewed in 47]. IDPs are defined by a disordered stretch of at least 30 residues [reviewed in 48]. Indeed, the *Xenopus* homolog Velo1 was shown to contain a low-complexity motif within the BUVE domain, which forms insoluble amyloids [25]. In addition, Osk and Buc were proposed to encode intrinsically disordered proteins [12, 49]. Similar to Osk and Buc, IDPs frequently evolve faster than structured proteins. Furthermore, IDPs can form liquid-liquid phase separations or hydrogels as found in RNP-granules or the germ plasm [30, 31, 48, 50, 51, reviewed in 52, 53, 54]. As the intrinsic disorder of Osk and Buc was previously not shown, we analyzed the intrinsic disorder prediction of Osk and Buc using the PONDR-VSL2 algorithm [55]. PONDR-VSL2 is a metapredictor, which conservatively combines the results of prediction algorithms. Both protein sequences displayed large disordered regions (Fig 4A and 4B). Interestingly, the previously identified prion-like domain in the N-terminus (aa 1–150) [25] appeared in this disorder prediction as the largest ordered sequence in Buc (Fig 4A). Prion-like domains and IDR are considered low complexity sequences suggesting that Buc almost entirely consists of low complexity sequences. We used zebrafish Vasa as a positive control for IDP prediction, which showed the known unstructured domain of about 200 aa at its N-terminus [56, 57], whereas Ziwi was largely structured (Fig 4C and 4D).

Osk does not display prion-like domains [25] but was recently shown to form aggregates in insect S2-cells supporting its prediction as an IDP [13]. To investigate, whether Buc forms similar protein aggregates, we transfected HEK293 cells with plasmids encoding fusions with monomeric GFP and eGFP. Buc and Osk formed protein aggregates, whereas the GFP control was uniformly distributed (Fig 4E–4G; S3A–S4C Figs). Moreover, when we cotransfected Buc-mGFP with Osk-Cherry, we found partially overlapping aggregates of Buc and Osk (Fig 4H–4J). These data indicate that Osk and Buc encode IDPs with a propensity to form cellular protein aggregates.

### Buc forms hydrogels in zebrafish embryos

**Fig 5:**
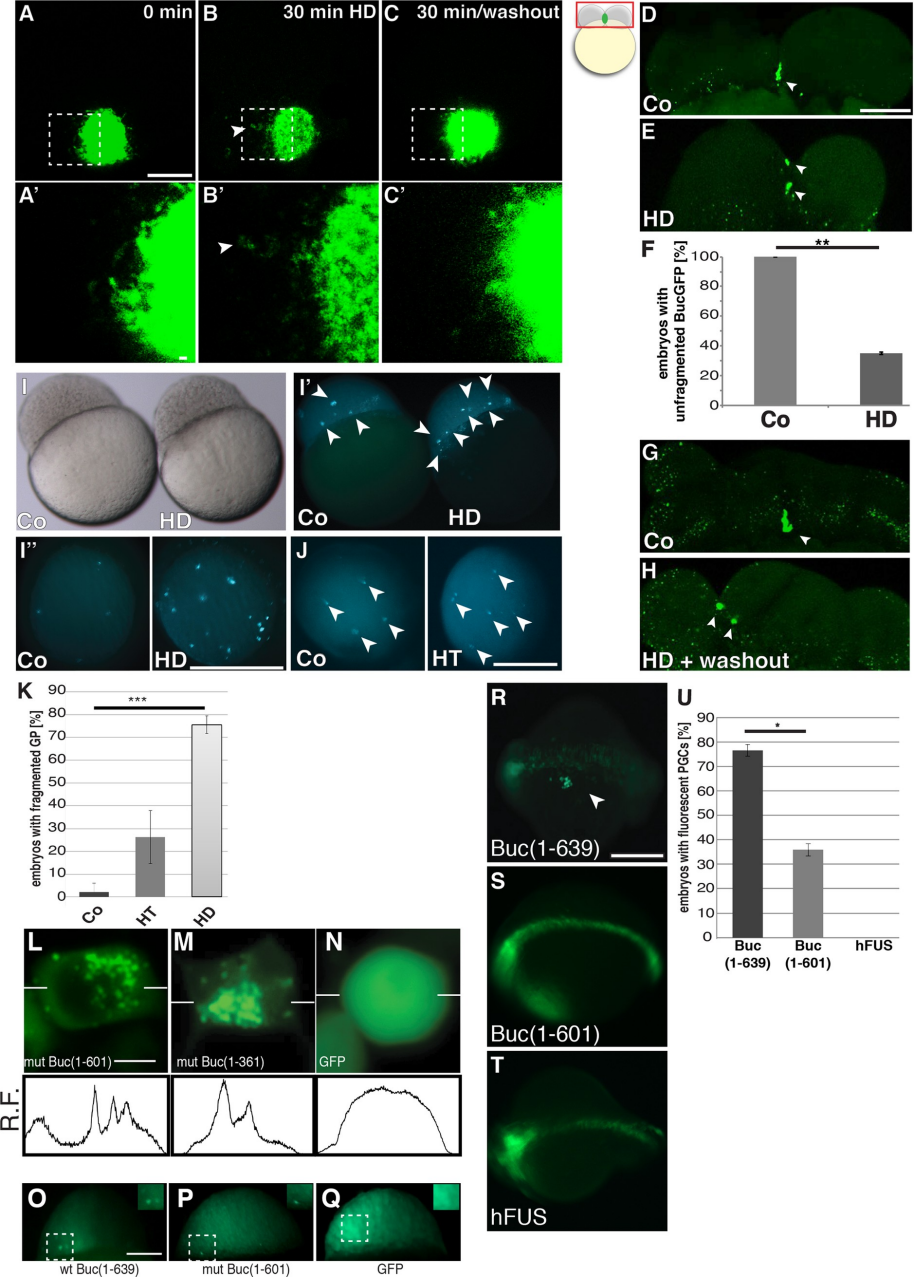
Pharmacological disruption of IDR-interactions leads to partially fragmented Buc-GFP aggregates. (A-C) Balbiani body of living Buc-GFP transgenic oocytes, either before (A), after a 30 min treatment with 5% 1,6-hexanediol (HD) (B), or 30 min after washout of the drug (lateral view, animal to the top). Arrowheads in B and B' indicate Buc-GFP granule outside the Balbiani body. Scale bar (A-C): 20 μm (A'-C'): 2 μm. (D-K) Germ plasm of transgenic Buc-GFP embryos after hexanediol treatment (HD). (D, E) lateral view of living 2-cell embryo as shown in boxed area of icon. Control embryos show unfragmented Buc-GFP aggregates (green) (D arrowhead), whereas 5% hexanediol for 30 min leads to fragmentation(arrowheads). (F) Quantification of embryos with unfragmented Buc-GFP in control (Co; 100±0%; n = 20) and embryos treated for 30 min with hexanediol (HD; 35.0±0.8%; n = 20; p = 0.0065). Student's t-test; P-value: **<0.01. (G, H) lateral view of living 4-cell embryos. Control embryo with unfragmented BucGFP (green, arrowhead), whereas Buc-GFP stays fragmented 30 min after washout of hexanediol (green; arrowheads). Scale bar (D-H): 100 μm. (I-K) Buc-GFP aggregates in 3 hpf embryos transgenic for Buc-eGFP. (I) The morphology of control (Co) and hexanediol-treated embryos (HD). Lateral view, animal to the top. (I', I'') Fragmented Buc-GFP aggregates (white arrowheads) persist until 3 hpf (I') lateral view, (I'') animal view. (J) Treatment with hexanetriol (HT) also leads to fragmented germ plasm (right embryo in J; animal view). Scale bar (I-J): 500 μm. (K) Quantification of germ plasm fragmentation (more than four puncta) at 3 hpf in controls (Co; 2.2±3.9%; n = 45), hexanetriol (HT; 26.3±11.5; n = 45) and hexanediol (HD; 75.5±3.9; n = 45; p = 1.9e-08). Error bars represent standard deviation of the mean. Student’s t-test; P-value: ***<0.001. (L-N) Protein aggregates upon transfection of HEK cells with (L) Buc(aa1-601)-GFP (50.32±2.95%; n = 70 percentage of transfected cells showing aggregated GFP signal), (M) Buc(aa1-361)-GFP (77.9±8.8%; n = 89) and (N) GFP (0%; n = 81). Scale bar (L-N): 10μm. (O-Q) Buc aggregation in zebrafish embryos. Embryos at 3 hpf after injection of mRNA encoding wt Buc(aa1-639)-eGFP (O), Buc(aa1-601)-eGFP (P) or eGFP(Q) at the one cell stage (lateral view, animal to the top). Scale bar (O-Q): 200 μm. Note the aggregation of wt Buc (aa1-639) and Buc (aa1-601) compared to GFP (insets; 25x magnification of stippled box). (R-U) IDRs are not sufficient for germ cell induction. Embryos form germ cells (white arrowheads) after injection with wt *buc* mRNA (aa 1–639) (R; 76.6±2.3%; n = 60), but less with mutant Buc (K; aa1-601) containing most IDRs (S; 35.9±2.6%; n = 60; p = 0.04) or an unrelated IDP (human FUS; T; 0±0; n = 26). Scale bar (J-L): 200 μm. (I) Quantification of injection results (three independent experiments for each RNA). Error bars represent standard deviation of the mean. Student’s t-test; P-value: *<0.05.

A short treatment with the aliphatic solvent 1,6-hexanediol dissolves hydrogels formed by IDPs as described for germline P-granules in the *C*. *elegans* ovary, but not amyloid-like aggregates like the Balbiani body in *Xenopus* oocytes [25, 58, 59]. To distinguish whether germ plasm in zebrafish forms amyloid-like aggregates or hydrogels, we treated ovaries of Buc-GFP transgenic females with hexanediol (HD). Using time lapse-microscopy we observed that a 30 min exposure to hexanediol did not disperse the Balbiani body (Fig 5A and 5B; S1–S4 Movies). Extending the treatment to 3 hrs or doubling the hexanediol concentration to 10% did also not dissolve the Balbiani body (S4A–S4D Fig). This result corroborates the amyloid-like character of Buc aggregates [25]. Nonetheless, we noted that some Buc-GFP granules drained off the Balbiani body leaving behind a perforated Buc-GFP scaffold (Fig 5B). Interestingly, 30 minutes after washing out hexanediol, the Balbiani body was restored similar to untreated oocytes (Fig 5C). Hexanediol did not affect oocyte microtubules or microfilaments (S4E–S4L Fig) in line with previous studies on *Xenopus* oocytes showing that none of these cytoskeletal elements seems to be required for Balbiani body integrity [60, 61]. The hexanediol experiments suggest that Buc condensates in the Balbiani body have a partially liquid and partially solid character during zebrafish oogenesis, which is consistent with a continuous hardening model of protein condensates [reviewed in 62].

The *Xenopus* germ plasm was proposed to acquire a more liquid character at the end of oogenesis [25]. Indeed, our own time-lapse imaging results with embryos from Buc-GFP transgenic mothers support the liquid behavior of embryonic germ plasm in zebrafish [63]. We therefore treated embryos with hexanediol and observed the integrity of germ plasm by time-lapse microscopy. To our surprise, the embryonic germ plasm never completely dissolved like shown for the *C*. *elegans* ovarian P-granules, but only fragmented (Fig 5D–5F). In contrast to the oocyte however, the germ plasm did not reaggregate after washout of the drug (Fig 5G and 5H). When we analyzed the surviving embryos at 3 hpf, the majority showed numerous fragments of Buc-GFP aggregates, whereas control-treated embryos showed no change (Fig 5I and 5K). 1,2,3-hexanetriol (HT) is chemically similar and frequently used as a control for the specificity of hexanediol [59]. Indeed, the more polar structure of hexanetriol disrupted Buc-GFP aggregates less efficiently than hexanediol (Fig 5J and 5K). These results support the hypothesis that zebrafish germ plasm forms an intracellular hydrogel, whose aggregation is probably mediated by intrinsically disordered regions (IDRs) of Buc.

We next addressed whether Buc aggregation is sufficient for germ cell specification. Buc(1–601)-GFP lacks 38 C-terminal amino acids thereby retaining most of the IDRs (Fig 4A). Buc(1–601) still forms protein aggregates in HEK293 cells (Fig 5L). Reducing Buc further to aa 1–361 still leads to protein aggregation compared to a GFP control (Fig 5M and 5N). The aggregation of wt Buc(aa1-639) and mutant Buc(aa1-601) was confirmed in zebrafish embryos (Fig 5O–5Q). However, Buc (aa1-601) injected embryos did not show ectopic PGCs (Fig 5R, 5S and 5U). Furthermore, the intrinsically disordered RNA-binding protein FUS [reviewed in 64] did not induce the PGC reporter (Fig 5T and 5U) suggesting that aggregation is not sufficient to specify germ cells and that other biochemical interactions are critical for germ cell specification.

### Buc and Osk bind zebrafish Vasa

**Fig 6:**
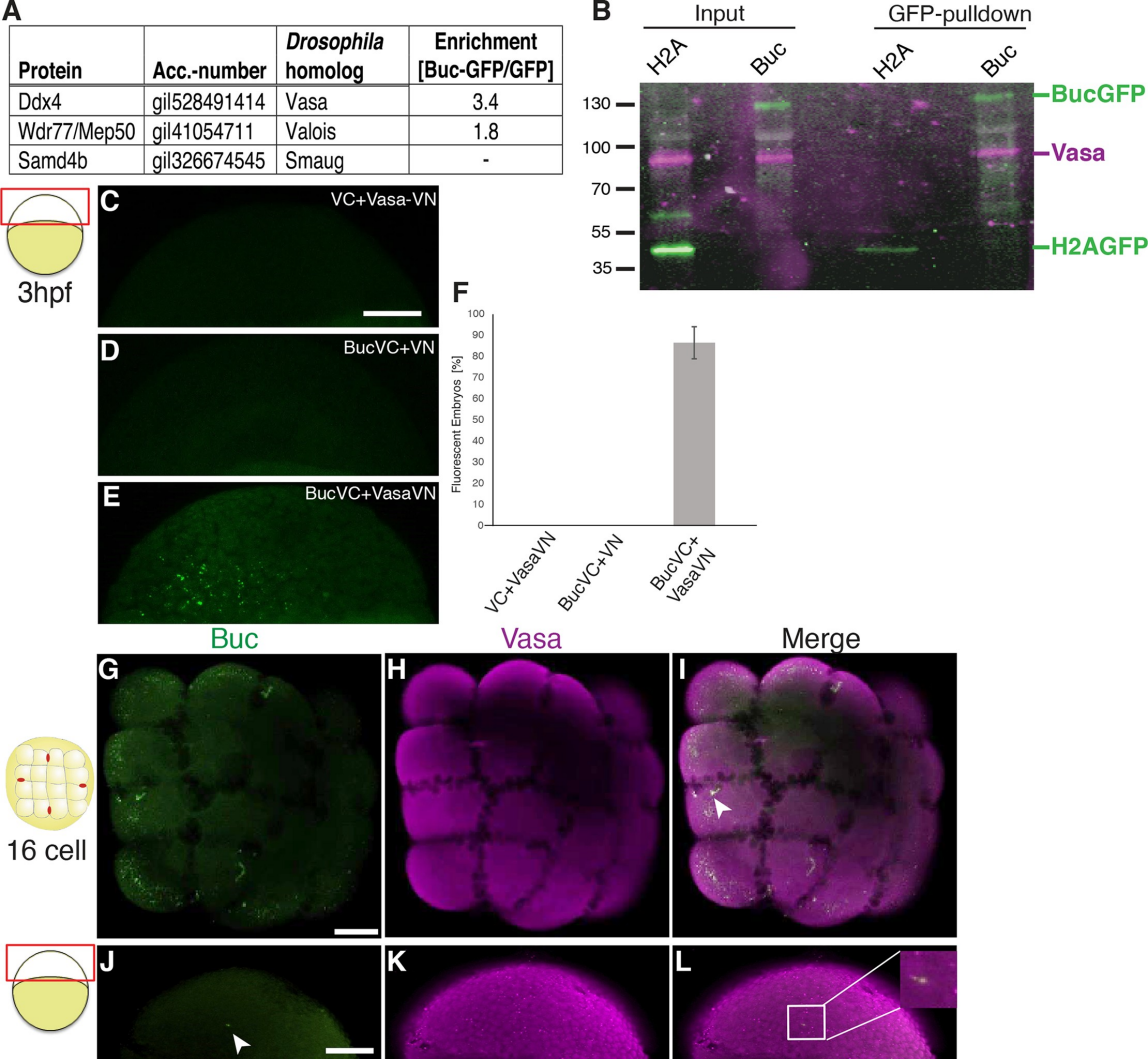
Buc binds zebrafish Vasa. (A) Zebrafish homologs of known Oskar binding proteins in the Buc-interactome detected Vasa (Ddx4) and Valois (Wdr77/Mep50), but not Smaug (Samd4b). Enrichment indicates the ratio of unique peptide counts after Buc-GFP pulldown to GFP-control samples. (B) Buc binds to Vasa *in vivo* during germ cell specification. Immunoprecipitations from 3 hpf H2A-GFP (42 kD) or Buc-GFP (130 kD) transgenic embryos blotted with GFP (green) and Vasa (magenta) (input = 20% of pulldown). (C-F) Buc and Vasa interact in bimolecular fluorescent complementation assays (BiFC). (C-E) live embryos at 3 hpf as indicated by the cartoon on the left, are not fluorescent (green) upon injection of mRNA encoding VC with Vasa-VN (C; 0±0%; n = 67) or Buc-VC with VN (D; 0±0%; n = 56), but form fluorescent Venus protein with Buc-VC and Vasa-VN (E; 86.5±7.5%; n = 53). Scale bar (C-E): 100 μm. (F) Quantification of BiFC assay (three independent experiments for each RNA). Error bars represent standard deviation of the mean. (G-L) Immunostaining of 16-cell stage (G-I) or 3 hpf (J-L) embryo as indicated by the cartoon on the left showing expression of Buc (green) and Vasa (magenta), inset in (L) shows a 10x magnification of the boxed area. Scale bar (G-L): 200μm.

The similar function of Osk and Buc postulates that they perform similar biochemical interactions, which then initiate the PGC-specification program. However, the fast sequence evolution of the IDRs in both proteins may obscure sequence similarities detectable by current alignment algorithms, which then bind to conserved interactors. Osk binds to Smaug, Valois, and Vasa protein [15, 65, 66]. To test whether these proteins are conserved in the Buc interactome, we immunoprecipitated Buc-eGFP from zebrafish embryos. To avoid non-specific interactions after overexpression, we used Buc-GFP transgenic fish, which express Buc under control of its own promotor [63]. We then identified binding partners by mass-spectrometry and searched the Buc interactome for the zebrafish homologs of Osk binding partners (Fig 6A). Interestingly, we found MACF1 highly enriched in the Buc interactome (S3 Table). Zebrafish mutants in *macf1* and *buc* show defects in embryonic polarity and Balbiani body localization [67–70] supporting the specificity of the biochemical interaction. Another good indicator for the specificity of the pulldown was the detection of the germ plasm component Ziwi (piwil1) [34], which was not enriched in the Buc sample (S3 Table). This result indicates that we did not bring down the entire germ plasm during Buc pull-down. Among the zebrafish homologs of Osk binding partners, we focused on Vasa for further analysis, since its stronger enrichment suggested a greater probability to interact with Buc.

Exciting structural studies showed that Vasa interacts with the extended LOTUS domain of Osk [13]. More specifically, helix α2 (aa156-167) and α5 (aa226-234) in the LOTUS-extension of Osk are required for Vasa interaction. Interestingly, α5 encodes an IDR, which folds into a helix on interacting with Vasa. Since we could not detect these peptide sequences in Buc with bioinformatics, we verified biochemically that Buc interacts with Vasa during the period of germ cell specification. We pulled down Buc-GFP from embryonic extracts of transgenic embryos at 3 hpf (Fig 6B). As controls we used the H2A-GFP transgenic line, which is one of the few strains in zebrafish expressing a GFP-fusion under maternal control similar to Buc-GFP [71]. We detected Vasa in Western blots after Buc-GFP pulldowns, but not with H2A-GFP controls suggesting that Vasa interacts with Buc *in vivo* during PGC specification.

To further corroborate the interaction of Buc and Vasa *in vivo*, we used bimolecular fluorescence complementation (BiFC) in early zebrafish embryos [20, 72, 73]. BiFC takes advantage of a split Venus protein called VN (N-terminal) and VC (C-terminal), which then complement into a functional fluorescent protein, if they are brought in close proximity. Coinjecting Vasa-VN with VC-Venus or Buc-VC with VN-Venus fragments did not form a fluorescent protein confirming the specificity of BiFC (Fig 6C and 6D). By contrast, overexpression of Buc-VC with Vasa-VN formed fluorescent aggregates in zebrafish embryos supporting the hypothesis that Buc binds Vasa *in vivo* (Fig 6E and 6F).

Vasa protein was previously described to be ubiquitously expressed during the maternally controlled embryogenesis [74, 75], while Buc protein is confined to the four germ plasm spots [17, 63, 76]. To support their biochemical interaction, we determined whether endogenous Buc and Vasa protein expression overlap during germ cell specification. Labelling zebrafish embryos by antibody staining showed that Vasa is ubiquitous at the 16-cell stage and at 3 hpf as previously described (Fig 6G–6L). Buc localization overlaps with Vasa only in the germ plasm, which further supports the hypothesis that Buc and Vasa might interact *in vivo*.

**Fig 7:**
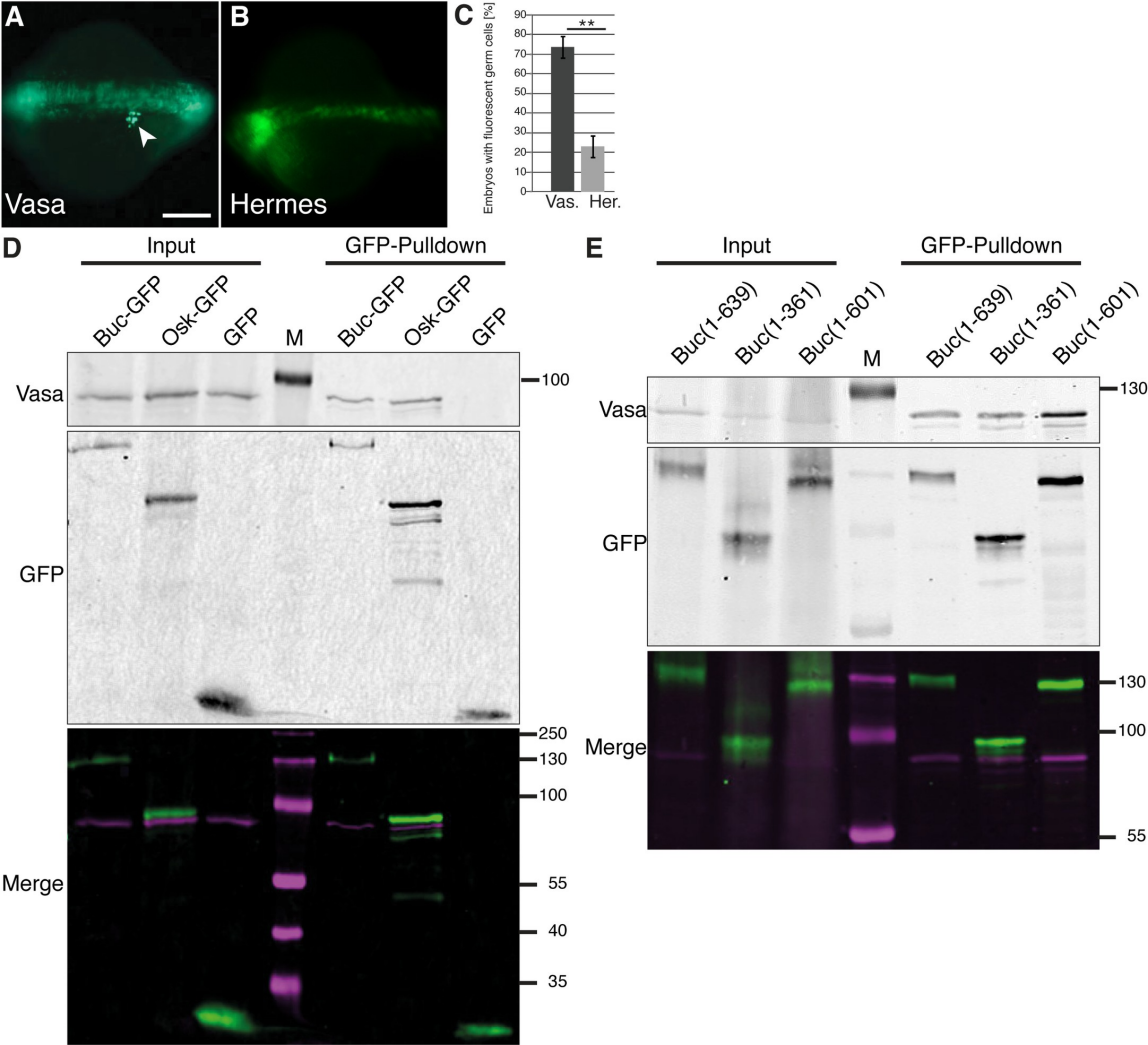
Zebrafish Vasa induces germ cells and binds to *Drosophila* Osk. (A-C) 16-cell assay showing germ cell formation (white arrowhead) after injection with *vasa* mRNA (A; 73.9±5.3%; n = 60; p = 0.01) but not with *hermes* (B; 22.9±4.8%; n = 60). Scale bar (A, B 200 μm (C) Quantification of injection results (three independent experiments for each RNA). Error bars represent standard deviation of the mean. Student’s t-test; P-value: **<0.01. (D) Western blot of Buc-GFP, Osk-GFP, and GFP-control together with Vasa after *in vitro* translation (input = 40% of pulldown) and after GFP-pulldown. Vasa (upper panel, magenta in merged panel) interacts with Buc and Osk, but not GFP controls (middle panel, green in merged panel). (M: molecular weight marker lane) (E) Western blot of Buc-GFP, Buc^p43^-GFP (1–361), and Buc^p106^-GFP (1–601) (middle panel, green in merged panel) together with Vasa (upper panel, magenta in merged panel) after *in vitro* translation (input = 40% of pulldown) and after GFP-pulldown. Vasa interacts with Buc, Buc(1–361) and Buc(1–601).

Previous reports in chicken showed that Vasa overexpression reprograms embryonic stem cells to a germline fate [77]. Furthermore, *Drosophila* Osk enhances Vasa activity suggesting that Vasa performs a key activity during germline specification [13]. We therefore analyzed the role of Vasa in the zebrafish germ cell induction assay. Surprisingly, Vasa induced ectopic germ cells, whereas another Buc binding protein Hermes [19–21, 23] showed no activity (Fig 7A and 7B). This result suggests that Vasa performs a critical activity during germ cell specification.

As Osk activates *Drosophila* Vasa and Vasa triggers germ cell formation in zebrafish, we investigated, whether *in vitro* translated Osk-GFP binds to zebrafish Vasa. Indeed, Osk pulled down zebrafish Vasa whereas controls did not interact (Fig 7D) supporting the hypothesis that Osk and Buc share conserved interactions. The Buc(1–361) and Buc(1–601) mutants do not induce PGCs and we therefore analyzed its interaction with Vasa. To our surprise both mutant alleles bound Vasa like wt Buc, (Fig 7E), whereas a control protein (non-muscle myosin II) was not bound by Buc (S5 Fig). Although these results show that the interaction with Vasa is conserved among germ plasm organizer proteins, the data also indicate that the mutant Buc proteins lack another critical interaction.

### Buc interacts with *nanos3* RNA

**Fig 8:**
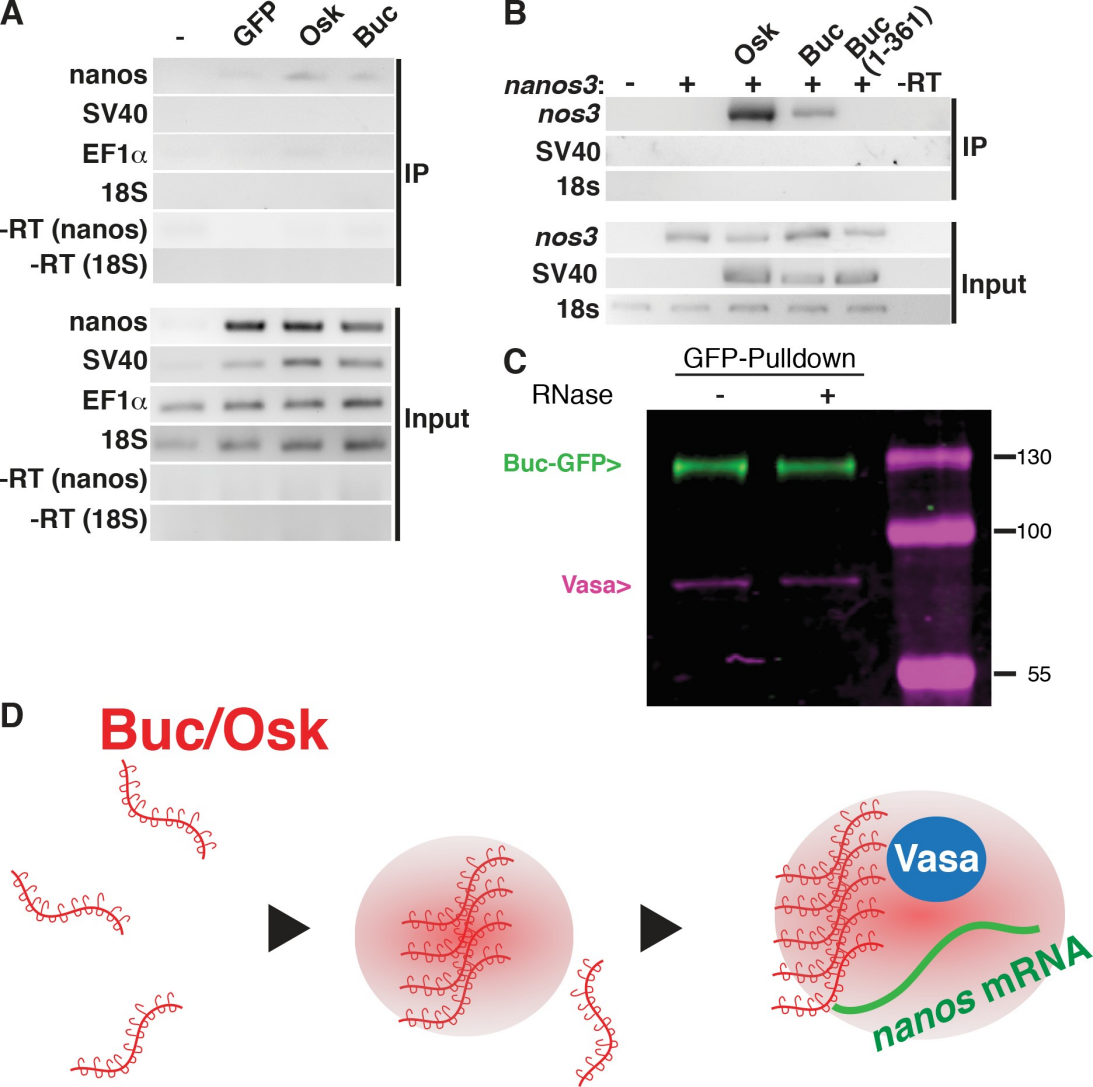
Buc interacts with RNA. (A) RT-PCR of zebrafish *nanos3*, SV40, human EF1α and human 18S rRNA after GFP-pulldown (IP) of HEK293 cells either untransfected (-) or transfected with GFP, Osk-GFP or Buc-GFP together with Cherry-nos-3'UTR and Cherry-SV40-3'UTR. RNA levels before GFP-pulldown (Input) show endogenous 18S rRNA and EF1α mRNA, and transfected Cherry-nos-3'UTR and Cherry-SV40-3'UTR. nos3-3'UTR is detected in Osk and Buc samples after GFP-pulldown (IP).–RT: RNA without reverse transcriptase control. (B) Buc(1–361)-GFP does not pulldown *nos3-3'UTR*. Cherry-nos-3’UTR (+), alone or cotransfected with Osk-GFP, Buc-GFP or Buc(1–361)-GFP including SV40-3'-UTR. (C) RNase treatment (+) prior to GFP pulldown of *in vitro* translated protein does not disrupt Buc-Vasa interaction. (D) Model for germ plasm formation. Single Buc or Osk molecules (red) aggregate through weak interactions of their intrinsically disordered regions (hooks and loops), until a threshold concentration is reached. This leads to a liquid-liquid phase separation (red haze) to form hydrogel-like germ plasm. The aggregate then recruits Vasa protein (blue) and *nanos* mRNA (green).

Mutant Buc(1–361) binds to Vasa, but does not induce germ cells suggesting that full-length Buc performs additional interactions. Osk was recently shown to bind RNA *e*.*g*. *nanos* [12, 14] and many IDPs are RNA-binding proteins [reviewed in 78]. To address whether Buc interacts with zebrafish *nanos3* mRNA [33], we coexpressed GFP-tagged versions in HEK293 cells. After immunoprecipitation of Buc, we detected zebrafish *nanos3* by RT-PCR, but not a cotransfected competitor 3'UTR (SV40) or an abundant, endogenous control (18S rRNA) (Fig 8A). Similarly, Osk-GFP bound to zebrafish *nanos3* mRNA. We then tested whether Buc(1–361) pulls-down *nanos3*-3'-UTR RNA. Indeed, this mutant Buc could not pull-down the RNA suggesting that it lacks a motif, necessary for RNA interaction (Fig 8B).

As Buc and Vasa interact with RNA, their interaction might be mediated indirectly *via* RNA. However, RNase treatment did not inhibit Buc-Vasa binding showing that the complex was held together by protein–protein interactions or was protected by RNA-bridging from nuclease activity (Fig 8C). These results discover two novel biochemical interactors of Buc *i*.*e*. Vasa protein and *nanos* mRNA, which are conserved with *Drosophila* Osk (Fig 8D).

## Discussion

Here we discover a conserved core complex, which is required for germ cell specification. This complex includes the conserved germline components Vasa protein [reviewed in 79, 80, 81] and *nanos* mRNA as well as a germ plasm organizer like Osk or Buc. These molecules are probably not the only components of the complex and might contain additional proteins or RNAs, since numerous, canonical germ plasm components are conserved in metazoan genomes [reviewed in 35, 82, 83]. For instance, while this manuscript was under revision, the Tudor protein Tdrd 6 was shown to interact with Buc in zebrafish [84]. This interesting study suggests that the Tdrd6 interaction controls the aggregation of Buc. Remarkably, Tudor as the founding member of this protein family was first discovered as a germ plasm component in *Drosophila* [85–87] thereby supporting the hypothesis of a conserved core of germ plasm components in metazoans.

In addition to a conserved interactome, Osk and Buc also share intrinsically disordered regions (IDRs), which probably form weak interactions to oligomerize (Fig 8D). Multimerization of intrinsically disordered proteins causes phase-transitions or biological condensates [reviewed in 88]. The hydrogel-disruptor hexanediol dissolves germ plasm in *C*. *elegans*, whereas we observed fragmentation in zebrafish suggesting that the liquid character of germ plasm varies in different species [50, 59, 89]. In *Xenopus* eggs however, the Buc homolog Velo forms amyloid aggregates, which are resistant to hexanediol [25, 58, 90]. The less liquid character of amyloids is also consistent with the initial description of the germ plasm harboring Balbiani body in spider oocytes, which shows a more solid state [91]. At the end of frog oogenesis however, it was reported that Velo does not form amyloid-like aggregates anymore, which is in line with a more liquid behavior of the germ plasm in the embryo [25, 63].

Our hexanediol experiments are similar to Mip6p aggregates in yeast [90]. A 30-min pulse of hexanediol treatment leads to the fragmentation of Mip6p aggregates and to reaggregation after wash-out of the drug. Interestingly, the reassembled Mip6p aggregates were inherited symmetrically during cell division, whereas Mip6p granules are inherited asymmetrically in untreated controls leading to more Mip6p positive cells. This fragmentation of Mip6p aggregates seem similar to the behavior of germ plasm in zebrafish, which eventually results in the increased number of Buc aggregates 2 hrs after hexanediol treatment.

Our data are consistent with a model, in which germ plasm organizers like Buc provide a scaffold, which nucleate a phase transition at a specific location in the embryo. This aggregation drives the recruitment of other germ plasm components and eventually germ cell specification (Fig 8D). Interestingly, RNAs might not only contribute to the specificity of different granules, but also seem to nucleate phase transitions by recruiting IDPs as shown in the fungus *Ashbya gossypii* [92]. This might explain why the IDRs of Buc are not sufficient for germline formation. Our results however suggest that phase-transition of germ plasm seems to have a rather permissive than an instructive role for germline formation. Although the liquid nature of germ plasm was described in different organisms, the purpose of forming these aggregates for germ cell development is still a matter of debate.

Vasa seems to be a central component for germline specification. It was already reported that Vasa overexpression in chicken embryonic stem cells induces germ cells [77]. In addition, a zebrafish *vasa* mutant does not maintain *nanos3* mRNA expression and thereby loses its germline stem cells [93]. Interestingly, Osk activates Vasa helicase activity demonstrating that in *Drosophila* the germ plasm organizer has an instructive role in germline specification [13]. Buc might also regulate Vasa activity in zebrafish and not only act as a scaffold recruiting Vasa to the germ plasm. Our results showing germ cell induction after Vasa overexpression would be consistent with this model. However, Vasa is already expressed in somatic cells in the early zebrafish embryo, which raises the question, why its overexpression reprograms a corner blastomere to the germline. We speculate that overexpression of Vasa bypasses the requirement for an activator. Such an effect was also observed for intracellular signaling components. For instance, Smad proteins in the BMP pathway are active after overexpression, but their endogenous activation requires phosphorylation [94]. Similarly, overexpression of Vasa might therefore have sufficient activity to start germline specification. In this model, it is not the localization of Vasa protein, which marks the germline of a species, but its activity. We therefore speculate that the activity of Vasa would be a more reliable marker for the germline. It would be more precise to visualize the early germline by the localization of a germ plasm organizer as an activator like Oskar or by the downstream products of Vasa's helicase activity such as piRNA maturation [95–97]. This is especially interesting in species similar to zebrafish such as the sea urchin, where Vasa is ubiquitously present in the early embryo [98].

A fascinating finding of our study is that Osk and Buc share some biochemical interactions despite the absence of recognizable sequence homologies. These similarities are remarkable considering that vertebrates and dipterans split more than 500 million years ago [99]. Two alternative scenarios could explain this functional equivalence. Both proteins are analogous designs, which converged at recruiting a similar interactome during evolution. We cannot rule out this model, but it seems most plausible for somatic tissues, where the loss of an organ might not lead to an evolutionary dead-end. By contrast, tinkering with a germ plasm organizer during evolution would result in reduced or missing fertility, and eventually the extinction of the entire species. As the invention of novel proteins with identical functions is very unlikely [reviewed in 37, 100, 101], the convergence model for germ plasm organizer evolution becomes increasingly complex to explain.

We therefore favor the second scenario, in which Osk and Buc are homologs, which diverged from a common ancestor. They probably have unrelated sequences, because their role as intrinsically disordered scaffolds releases the constraints to maintain a defined protein structure as described for other IDPs [reviewed in 102]. This model is supported by the recent finding that the LOTUS domain is not sufficient to bind Vasa, but requires an intrinsically disordered extension (aa 226–234) of low evolutionary conservation [13]. In addition, germ cell-specification is a very early event during the evolution of multicellularity and hence, germ plasm organizer proteins have a long history of diverging during evolution [103]. The fast evolution of IDPs probably hides conserved motifs, which bind to a similar interactome such as Vasa and *nanos* mRNA [51, reviewed in 53, 54]. Indeed, a similar situation was previously described for the intrinsically disordered domains CID and NCBD [32].

This hypothesis would also predict that Osk and Buc have a similar structure, which would explain their conserved interactions. There are already known examples of protein pairs with structural similarity, which do not display a related amino acid sequence. For instance, Sumo and Ubiquitin show a sequence identity of 18%, but form almost identical structures [104]. Despite their similar structure, both have different biological roles [reviewed in 105]. Moreover, Hsc70 and Actin provide another example for structural similarity without sequence conservation [106]. Furthermore, biochemistry has isolated numerous analogous enzymes *e*.*g*. carbonic anhydrases from different organisms, which show identical biochemical activities without related sequences [reviewed in 107]. However, in none of these examples, the conservation of their biological role was investigated, *i*.*e*. whether the function of a protein is conserved in the other species like Osk in zebrafish. It will therefore be fascinating to learn how similar the structure of Buc is, compared to the known structure of Osk [12–14].

The functional equivalence of germ plasm organizers in the absence of sequence similarity might be more widespread including other species. For instance, *C*. *elegans* germ plasm or P-granules have a similar composition, since they also contain Vasa protein and *nanos* RNA [108, reviewed in 109, 110–112]. Although the identity of a germ plasm organizer protein in *C*. *elegans* is currently not clear, it has been speculated that MEG-3 or PGL proteins might act as P-granule nucleators similar to Osk [113–115]. MEG-3 binds RNA [113] and could therefore interact with the *C*. *elegans* homologs of *nanos* mRNA and Vasa protein similar to Osk and Buc. Furthermore, recent studies on PGL-3 show that it binds *nanos 3* RNA albeit weakly [116]. Moreover, PGL proteins are not found in vertebrate or insect genomes and nucleate the formation of hydrogels [116]. PGL proteins genetically interact with Vasa homologs [112, 117], but the direct biochemical binding was not tested. These examples provide some candidates in *C*. *elegans*, whose conservation as germ plasm nucleators will not be revealed by sequence comparisons but need to be analyzed with functional and biochemical experiments.

However, Osk, Buc or germ plasm organizers in other species could only be termed true homologs, if the identity of a common ancestor is known. Without this information, the functional similarity of two proteins without sequence homology remains a fascinating, but unique case. Contrarily, Osk and Buc could also represent a widespread phenomenon. In a more global perspective, more protein-pairs could exist, which are currently termed "novel" or "species-specific" but show similar functions across distant species.

## Materials and methods

### Ethics statement

Fish were maintained as described [118] in accordance with regulations of the Georg-August University Goettingen, Germany. Zebrafish experiments were conducted according to EU directive 2010/63/EU and maintained according to the EuFishBioMed/Felasa recommendations (https://www.eufishbiomed.kit.edu/59.php). Experiments were approved by the Lower Saxony State Office for Consumer Protection and Food Safety (AZ14/1681).

### Zebrafish

Injections were performed into wild-type embryos (hybrid of *ABxTLF). 16-cell embryos were injected as previously described [17]. At least 20 embryos were sorted per injection and for biological replicates independent clutches of eggs were used. One blastomere was injected with 0.5 nl RNA-solution containing 100 pg/nl of PGC-reporter (*GFP-nos-3´UTR*) plus 100 pg/nl mRNA encoding a germ plasm component. *Buc* and short *osk* mRNAs contained their 5´ and 3´UTR sequences, respectively. *Buc*^*p43*^ and *buc*^*p106*^ were identical to wt mRNA except for a premature stop codon in 362 aa and 602 aa, respectively [17]. Short *osk* and *osk*^*084*^ mRNA were identical except for the premature stop codon in *osk*^*084*^ [9].

BiFC assays were performed with modifications as previously described [72]. Briefly, wild-type embryos (hybrid of *ABxTLF) were injected at the one-cell stage with the mRNAs encoding the VN- and VC-fusions (200 pg each). Embryos were imaged for fluorescence at the 3hpf stage with a LSM780 confocal microscope (Carl Zeiss Microscopy, Jena).

Dechorionated Buc-GFP transgenic embryos at the one cell stage were treated with 1,6-hexanediol or 1,2,3-hexanetriol (5% w/v in E3-medium) for 30 min, whereas control treated embryos were exposed to E3-medium. Embryos were incubated at 28.5°C in glass dishes for 30min and then transferred into fresh E3-medium in agarose coated dishes at 28.5°C until 3 hpf.

### Protein biochemistry

#### Embryonic extracts

Embryos were enzymatically dechorionated for 3–5 min in Pronase solution (3 mg/ml in E3-buffer) and washed three times with E3. Then, embryos were deyolked in 50% Ginzburg Fish Ringer with Calcium (55 mM NaCl, 2.7 mM CaCl_2_, 1.8 mM KCl, 1.25 mM NaHCO_3_) as described [119] and the cell pellet washed with wash buffer (10 mM Tris pH 8.5, 110 mM NaCl, 3.5 mM KCl, 2.7 mM CaCl_2_). Cells were homogenized on ice in lysis buffer (10 mM Tris pH 7.5, 150 mM NaCl, 0.5 mM EDTA, 0.5% NP-40, 1x complete protease inhibitor cocktail (Roche Mannheim)) and cell debris removed by centrifugation. The supernatant was incubated with GFP-Trap magnetic beads (ChromoTek, Planegg-Martinsried) according to instructions by the manufacturer. Beads were either directly resuspended in SDS-loading buffer for gel electrophoresis or processed by the Proteome Analysis Core Facility of the University Medical Center, Goettingen.

#### In vitro translation

The Promega TnT SP6 Quick Coupled Transcription/Translation System was used to synthesize proteins. Products were diluted in YSS buffer and for pull-downs GFP Trap beads were used as described above.

#### Western blot

Proteins were separated on denaturing 10% SDS-PAGE and transferred to nitrocellulose by semi-dry blotting for 70 min at 25V. Blots were blocked in 5% TBS/milk for 1 hr, incubated overnight with primary antibody, washed, incubated with fluorescent secondary antibody and detected with the Li-Cor Odyssey CLx Infrared Imaging system (Li-Cor, Lincoln, USA). Antibodies: rabbit anti-Vasa (1:500; Genetex GTX128306 and gift from G. Vorbrueggen, MPI-BPC Goettingen), mouse anti-GFP (1:1,000; Roche 11814460001), mouse anti-Myc (1:200; Sigma), goat anti-rabbit (1:20,000; IRDye, Li-Cor, P/N 925–68071), goat anti-guinea pig 800CW (1:20,000; IRDye, Li-Cor, P/N 925–32210).

#### Immunohistochemistry

Embryos were fixed and stained as previously described [63]. Microtubule and f-Actin labeling was performed as published [120]. Antibody dilutions: guinea pig anti-Buc (1:5,000) [63], rabbit anti-Vasa (1:500). Secondary antibodies goat-anti-guinea pig Alexa Fluor 488 (1:500, Life Technologies, Carlsbad, USA, **#**A-11073), goat anti-rabbit Alexa Fluor 594 (ab150080) and imaged with a LSM780 confocal microscope (Carl Zeiss Microscopy, Jena).

#### Protein aggregation assay

HEK cells (10^4^/per well) in an eight-chambered slide (Sarstedt) were transfected using ScreenFectA reagent with 100 ng of the indicated plasmids (S4 Table). Cells were imaged after 48 hrs using 10X objective with a 10X digital zoom with an LSM780 confocal microscope and cell profiles were analyzed with ZEN2011 software (Carl Zeiss Microscopy, Goettingen).

### Proteomics

Sample preparation: Proteins were separated on denaturing 4–12% gradient SDS-PAGE (Invitrogen, Carlsbad/CA, U.S.A.). After Coomassie staining for visualization, each lane was cut into 23 equidistant slices irrespective of staining. For in-gel digestion, gel slices were washed with water, reduced with dithiothreitol (10 mM in 100 mM NH_4_HCO_3_, 50 min, 56°C) and alkylated with iodoacetamide (55 mM in 100 mM NH_4_HCO_3_, 20 min, RT, dark). In between, the gel slices were washed with acetonitrile for 15 min and dried in a Speedvac at 35°C. Gel slices were digested overnight at 37°C with porcine trypsin (12.5 ng/μl in 50 mM NH_4_HCO_3_, 5 mM CaCl_2_). Peptide extraction from the gel slices was performed with aqueous acetonitrile.

#### Mass spectrometry

For LC-MS analysis, the peptides derived from each SDS-PAGE slice were dissolved in 20 μl of 2% (v/v) acetonitrile and 0.1% (v/v) formic acid aqueous solution. 5 μl each was injected for LC-MSMS analysis. LC-MSMS was carried out using the following conditions: Peptides were separated on an Easy nLC-1000 nanoflow chromatography system (Thermo Fisher Scientific, Bremen, Germany) operated in a vented column setup. Concentration and desalting was achieved on an in-house packed C18 trap column (2 cm, 150 μm I.D., Reprosil-Pur C18-AQ, 120 Å, 5 μm, Dr. Maisch, Ammerbuch-Entringen, Germany) with 20 μl of Buffer A (0.1% formic acid). Peptides were separated on an in-house-packed C18 column (20 cm, 75 μm I.D., Reprosil-Pur C18-AQ, 120 Å, 3 μm, Dr. Maisch) at a flow rate of 300 nl/min with a gradient from 5–35% of Buffer B (95% acetonitrile, 0.1% formic acid) for 37 min. Eluting peptides were analyzed on-line on a Q Exactive hybrid quadrupole/orbitrap mass spectrometer (Thermo Electron) operated in Data Dependent acquisition mode where the 10 most intense ions in the MS scan (m/z 350–1400, resolution 70.000 at m/z 200, target value 1•10E6) were selected for fragmentation by Higher Collision Energy Dissociation (2 m/z isolation window, 25% Normalized CE, Start Mass 100, resolution 17.500 at m/z 200, target value 2•10E5). Sequenced precursors were put on a dynamic exclusion list for 15 sec. The lock mass option (polysiloxane at m/z 445.120025) was used for internal calibration.

#### Mass spectrometry data analysis

Peaklists were extracted from tandem mass spectra using Raw2MSM v1.7, selecting the top seven peaks for 100 Da. All MS/MS samples were analyzed using Mascot v2.4.1 (Matrix Science, London, UK). Mascot was set up to search the NCBInr_20130816 database (selected for *Danio rerio*, v20130405, 51384 entries) assuming the digestion enzyme trypsin. Mascot was searched with a fragment ion mass tolerance of 0.020 Da and a parent ion tolerance of 5.0 ppm. Carbamidomethylation of cysteine was specified as a fixed modification, deamidation of asparagine and glutamine, and oxidation of methionine as variable modifications, respectively.

Scaffold (version Scaffold_4.4.1.1, Proteome Software Inc., Portland, OR) was used to validate MS/MS based peptide and protein identifications. Peptide identifications were accepted if they could be established at greater than 95.0% probability by the Scaffold Local FDR algorithm. Protein identifications were accepted, if they could be established at greater than 99.0% probability and contained at least 2 identified peptides. Protein probabilities were assigned by the Protein Prophet algorithm [121]. Proteins that contained similar peptides and could not be differentiated based on MS/MS analysis alone were grouped to satisfy the principles of parsimony. Proteins sharing significant peptide evidence were grouped into clusters.

### RNA-Immunoprecipitation

HEK-293 cells (0.2 x10^6^/ well) were co-transfected with the indicated combinations of plasmids for protein and RNA expression (S4 Table). Cells were incubated for 48 hrs and screened for expression of GFP and Cherry fluorescence. Cells were then lysed in (0.5 ml) YSS buffer (50 mM Tris pH 8, 75 mM NaCl, 1 mM MgCl_2_, 100 mM sucrose, 1 mM DTT, 0.5% NP- 40, 1x complete protease inhibitor cocktail (Roche Mannheim)) and centrifuged for 10 min. (13,000 rpm, 4 ºC). 50 μl of the supernatant were kept aside as the input fraction and the rest was incubated with pre-blocked GFP nanotrap beads (Chromotek) for 3 hrs at 4°C. Beads were washed (YSS buffer) and the bound fraction was released from the beads in 5% SDS. RNA was isolated using phenol/chloroform/isoamylalcohol and precipitated in 0.3 M ammonium acetate/ 50% EtOH, washed with 70% EtOH and used for cDNA synthesis.

### RT-PCR

RNA was reverse transcribed for first strand synthesis using random hexamers and SuperScript II RTase (Thermo Fisher Scientific). cDNA was amplified using the primers described in S4 Table.

### Plasmids

Plasmids used in this study are listed in S4 Table.

### Bioinformatics

Protein sequences for *Danio rerio* Bucky ball, *Drosophila melanogaster* Oskar, and the respective orthologs were retrieved from the NCBI protein database. The vertebrate Buc sequences used for multiple alignments were: *Danio rerio* (gi|292610748), *Oryzias latipes* (gi|432930267), *Tetraodon nigroviridis* (gi|47225100), *Takifugu rubripes* (gi|410909482), *Oncorhynchus mykiss* (gi|642119256), *Pimephales promelas* (gi|73433600), *Ictalurus punctatus* (gi|311721748), *Xenopus laevis* (gi|148230857), *Xenopus tropicalis* (gi|301615136), *Anolis carolinensis* (gi|327275069), *Gallus gallus* (gi|118086206 / gi|513169732), *Canis familiaris* (gi|545522949 / gi|73976581). The Oskar insect sequences: *Drosophila melanogaster* (gi|45553317 / gi|24645205 / gi|317183309), *D*. *sechellia* (gi|195330556), *D*. *simulans* (gi|195572425), *D*. *yakuba* (gi|195499262), *D*. *erecta* (gi|194903569), *D*. *ananassae* (gi|194741640), *D*. *pseudoobscura* (gi|198454187), *D*. *persimilis* (gi|195152922), *D*. *mojavensis* (gi|195111098), *D*. *virilis* (gi|2498716 / gi|195389208), *D*. *immigrans* (gi|111663086 / gi|111663088), *D*. *grimshawi* (gi|195054868), *D*. *willistoni* (gi|195445335 / gi|195445337), *Aedes aegypti* (gi|83701126 / gi|157134733), *Culex quinquefasciatus* (gi|170041806), *Anopheles gambiae* (gi|118783859 / gi|333468779), *Anopheles darlingi* (gi|312371899), *Acromyrmex echinatior* (gi|332023144), *Nasonia vitripennis* (gi|302138022). Global/local pairwise alignments of Buc and Osk were performed using the EMBOSS tools *Needle/Water* (http://www.ebi.ac.uk/Tools/psa/) with default parameters. Multiple alignments of Buc/Osk and their respective orthologs were constructed with the T-COFFEE software version 8.69 using standard parameters [122]. Hidden Markov models (HMM) were built from the multiple alignments using the HMMER3 software in default configuration [123]. The HMMs were used to search the complete genomic protein sequence complement of *Danio rerio* and *Drosophila melanogaster* as obtained from the NCBI protein database. To detect potential distant relationship between the models, the HMMs were uploaded to the HHpred server [124]. The intrinsic disorder of proteins was predicted with PONDR-VSL2 in default configuration [125].

### Statistics

Error bars indicate the standard deviation of the average (at least three independent experiments). The statistical significance (P-value) of two groups of values was calculated using a two-tailed, two-sample unequal variance t-test with MS-Excel.

## Supporting information

S1 Fig Expression control of injected mRNAs. (A) Microinjection of 200 pg of the indicated mRNAs encoding GFP fusions leads to fluorescent embryos compared to uninjected controls (Co), lateral views, animal to the top. Scale bar: 200 μm. (B) Quantification of fluorescent germ cells per embryo at the 18-somite stage after injection of PGC reporter (GFP-nos3’-UTR) alone (Co; 4.0±1.9; n = 5) or together with Buc (10.3±1.2; n = 4; p = 0.0008) or sOsk mRNA (13.4±3.2 PGCs/embryo; n = 5; p = 0.0005) in a corner blastomere at the 16-cell stage. Error bars represent standard deviation.
(TIF)

S2 Fig *Drosophila* Oskar induces Vasa mRNA positive cells in zebrafish. *In situ* hybridization for *vasa* mRNA (blue) in 3 hpf embryos in animal view after injection of control (GFP; A) or *oskar* mRNA (B). Note the additional Vasa-positive germ cells (blue) after Oskar overexpression and the overall higher background after staining for the same period. Scale bar: 200 μm.
(TIF)

S3 Fig Buc and Osk aggregation in HEK cells. Protein aggregates upon transfection of HEK cells with enhanced GFP (eGFP) fused to (A) Buc (99.3 ±1.15%; n = 111 percentage of transfected cells showing aggregated GFP signal) (B) sOsk (83.17± 8.18%; n = 90) or (C) unfused (0%; n = 81). The profiles below the pictures show levels of fluorescent intensity along the line indicated by white dashes. Scale bar (A-C): 10μm.
(TIF)

S4 Fig Hexanediol treatment of oocytes and embryos. Buc-GFP (green) in the Balbiani body of stage Ib oocytes before hexanediol treatment (A, C; 0 min) or after 30 min treatment with double conc. (10%; B, D). Stippled squares indicate the magnified area shown in panel C and D. Note the BucGFP fragments draining off the Blabiani body after HD treatment (D). Scale bar (A, B): 20 μm; (C, D): 1 μm. Cytoskeleton after Hexanediol treatment. Oocytes (E-L) or embryos (M-T) were treated for 30 min with hexanediol and stained for microtubules (β-tubulin) or microfilaments (filamentous Actin). Stippled boxes (E-H, M-P) indicate magnified area (I-L, Q-T). 2-cell embryos (M-T)are shown in animal view. Scale bars (E-H, Q-T): 20 μm. (I-L): 1μm. (M-P):100 μm.
(TIF)

S5 Fig Buc does not interact with Non-muscle MyosinII. Western blot of Buc-GFP (green) and Myc-Non-muscle Myosin II (green; NMII; 20 kD) after *in vitro* translation (input = 40% of pulldown) and after GFP pulldown. Buc does not interact with NMII.
(TIF)

S1 Table Bucky ball and Oskar do not share sequence homology. Graph summarizing scores of global (white bar; Needleman-Wunsch) and local alignments (black bar; Smith-Waterman). Note that Buc-Osk alignments are equally low as the negative control (Buc-Dm Vasa), whereas ZfVasa and DmVasa show a characteristic score of two homologous sequences. Analysis of protein sequences with global pairwise alignments using the Needleman-Wunsch algorithm (A; http://www.ebi.ac.uk/Tools/psa/emboss_needle/; standard settings) or with local pairwise alignments using the Smith-Waterman algorithm (B; http://www.ebi.ac.uk/Tools/psa/emboss_water/; standard settings). Depicted are the percentages of similar and identical amino acids of two aligned protein sequences (sequences and raw data of sequence alignments in Supplementary Data 1).
(PDF)

S2 Table Comparison of Buc and Osk with Hidden-Markov-Models. Homology search with conserved domains using Hidden-Markov-Models (www.HMMer.org) of the respective proteins did not reveal any conserved domains between Oskar and Bucky ball. Hits of the used HMM in the NCBI databases are shown with their corresponding E-value.
(PDF)

S3 Table Comparative Analysis of GFP and Buc-GFP Samples by Mass Spectrometric Analysis. The number of successfully assigned MS/MS spectra per protein (Total Spectrum Counts, TSC) was normalized to 100% for each sample. Entries labeled 'Clusters' designate the identification of more than one protein sequence entry with largely redundant MS/MS evidence (>50% total sequence, >95% evidenced sequence). Following the principle of parsimony, only the best evidenced ('primary') protein in the cluster is listed.
(XLSX)

S4 Table List of plasmids and primers used. (DOCX)

S1 Movie Time-lapse confocal microscopy of Balbiani body (Co). Balbiani body (green) in stage Ib oocyte from Buc-GFP transgenic females. Only the first 5 minutes are shown (see S3 Movie for full time-lapse). Scale bar: 20 μm.
(WMV)

S2 Movie Time-lapse of hexanediol treated Balbiani body. The first 5 min after adding hexanediol to the medium are shown (see S4 Movie for full 30 min time-lapse). Scale bar: 20 μm.
(WMV)

S3 Movie Time-lapse of Balbiani body (Co). Full movie of S1 with untreated stage Ib oocyte. Scale bar: 20 μm.
(WMV)

S4 Movie Time-lapse of Balbiani-body treated with hexanediol. Full movie of S2 showing stage Ib oocyte treated with hexanediol for 3 hrs. Note the reduction of fluorescence within the first 5 minutes. Scale bar: 20μm.
(WMV)
