# Supplementary figures and images for: Functional equivalence of germ plasm organizers
Source: PLoS Genet. 2018 Nov 6;14(11):e1007696. doi: 10.1371/journal.pgen.1007696 (PMC6219760; doi:10.1371/journal.pgen.1007696)

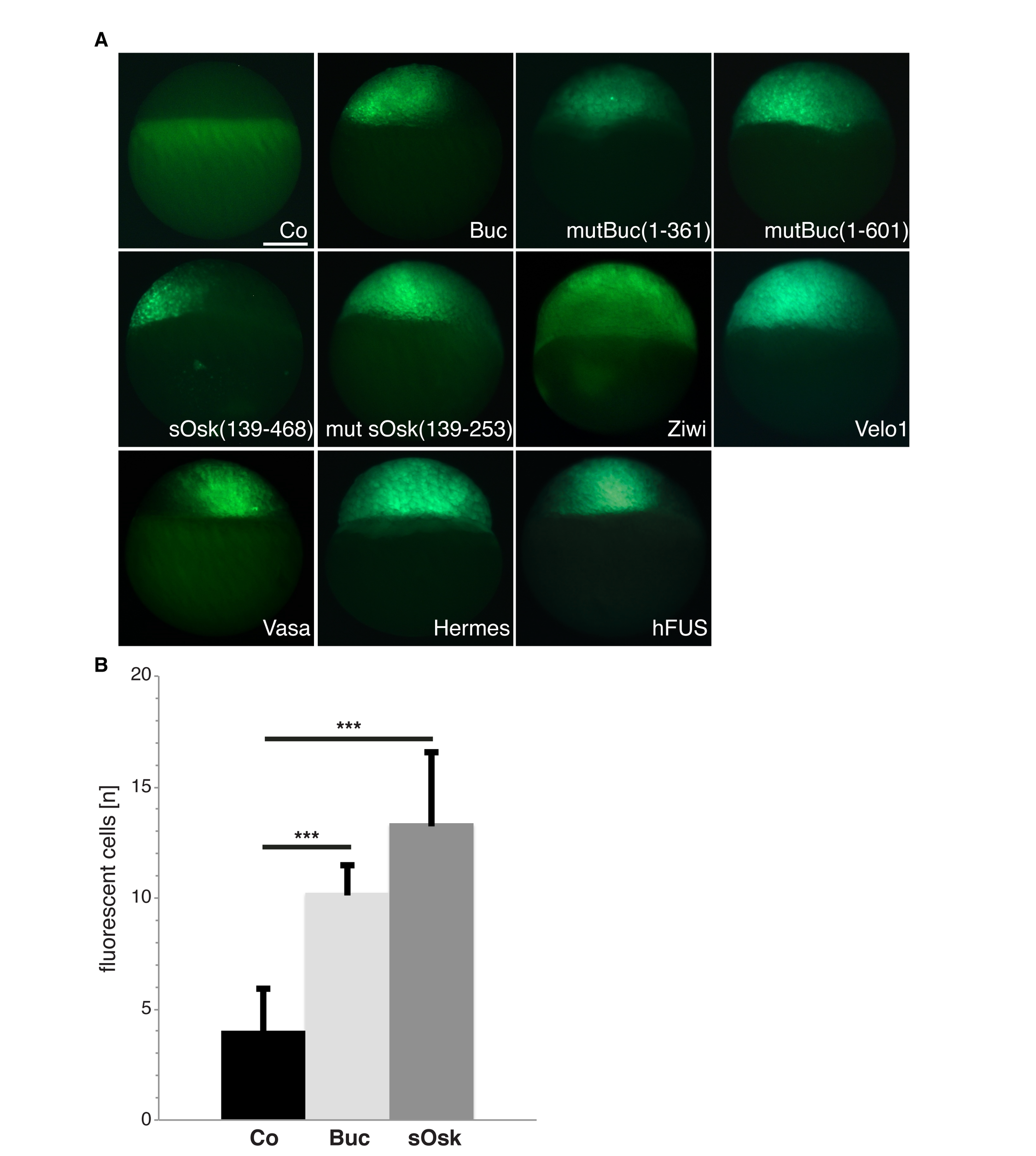

Supplement: S1 Fig — (A) Microinjection of 200 pg of the indicated mRNAs encoding GFP fusions leads to fluorescent embryos compared to uninjected controls (Co), lateral views, animal to the top. Scale bar: 200 μm. (B) Quantification of fluorescent germ cells per embryo at the 18-somite stage after injection of PGC reporter (GFP-nos3’-UTR) alone (Co; 4.0±1.9; n = 5) or together with Buc (10.3±1.2; n = 4; p = 0.0008) or sOsk mRNA (13.4±3.2 PGCs/embryo; n = 5; p = 0.0005) in a corner blastomere at the 16-cell stage. Error bars represent standard deviation. (TIF) [file pgen.1007696.s001.tif]

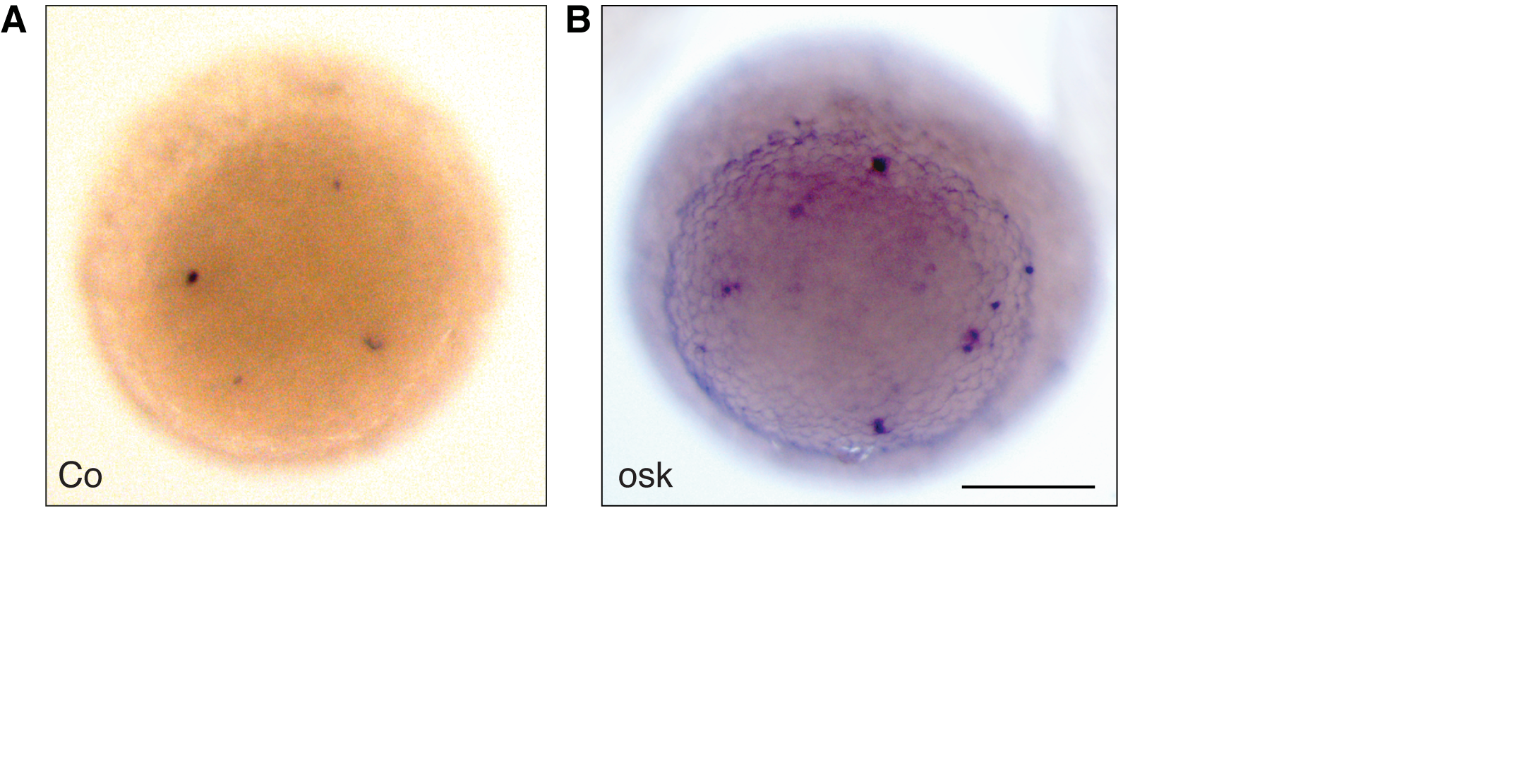

Supplement: S2 Fig — In situ hybridization for vasa mRNA (blue) in 3 hpf embryos in animal view after injection of control (GFP; A) or oskar mRNA (B). Note the additional Vasa-positive germ cells (blue) after Oskar overexpression and the overall higher background after staining for the same period. Scale bar: 200 μm. (TIF) [file pgen.1007696.s002.tif]

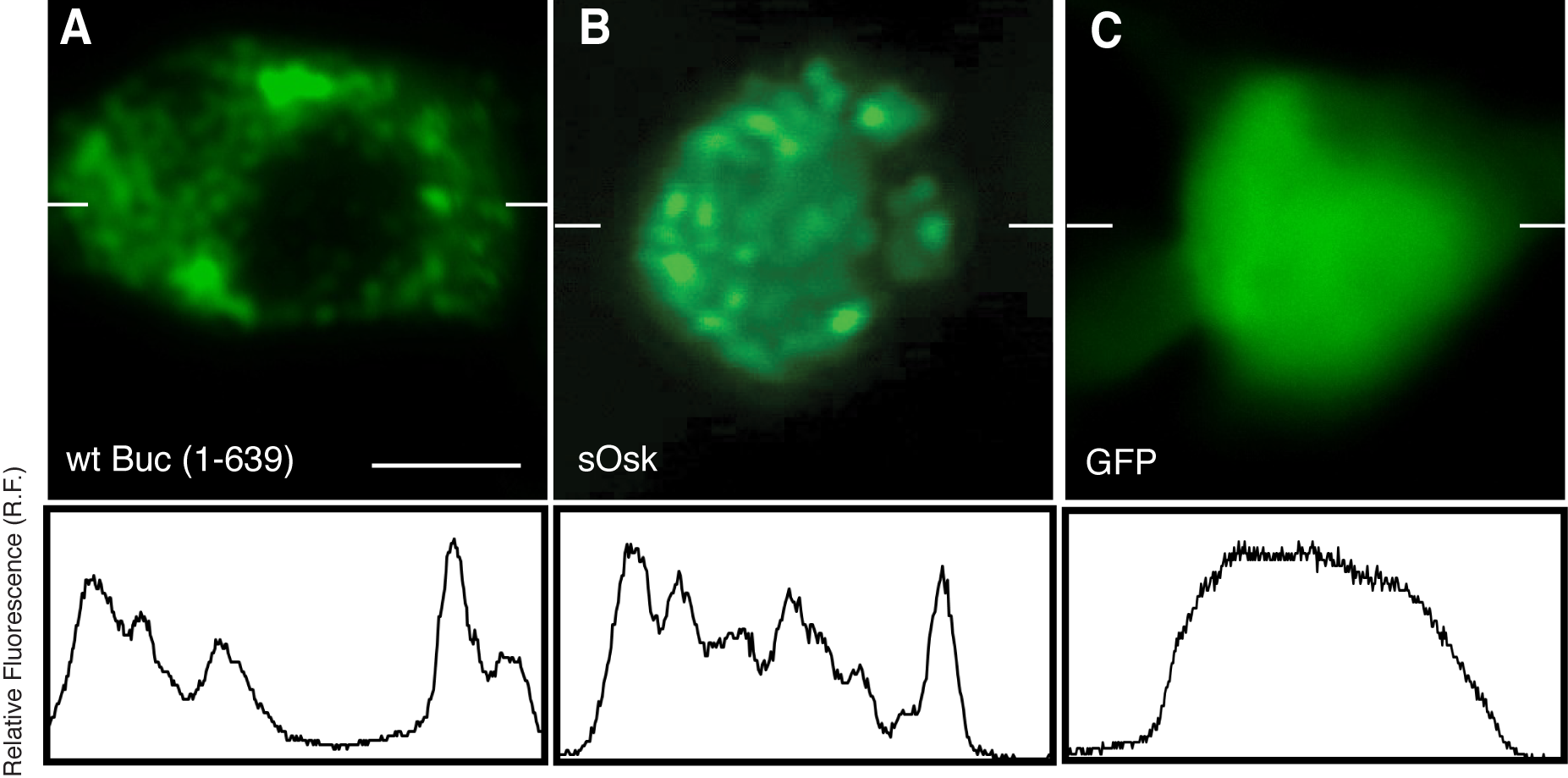

Supplement: S3 Fig — Protein aggregates upon transfection of HEK cells with enhanced GFP (eGFP) fused to (A) Buc (99.3 ±1.15%; n = 111 percentage of transfected cells showing aggregated GFP signal) (B) sOsk (83.17± 8.18%; n = 90) or (C) unfused (0%; n = 81). The profiles below the pictures show levels of fluorescent intensity along the line indicated by white dashes. Scale bar (A-C): 10μm. (TIF) [file pgen.1007696.s003.tif]

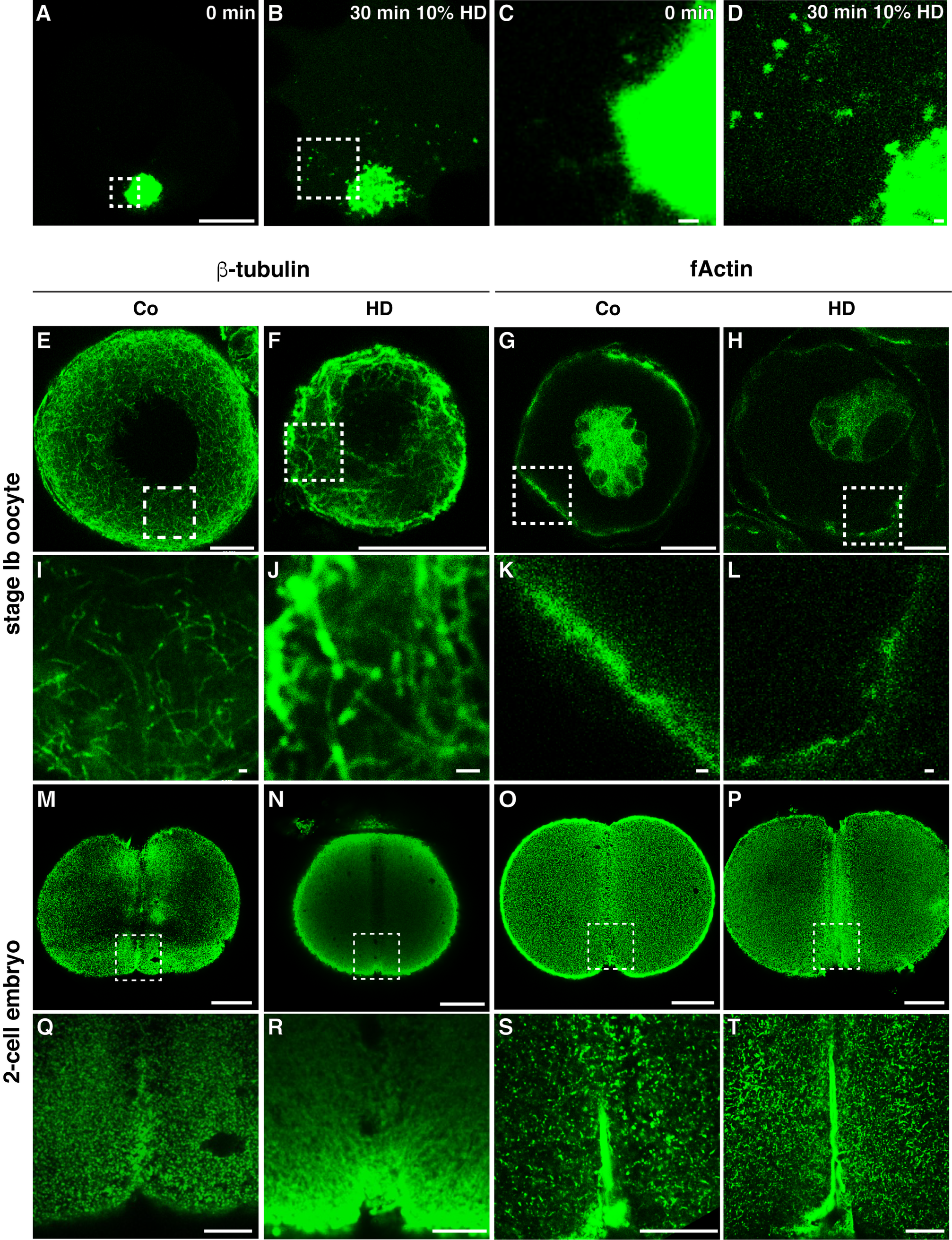

Supplement: S4 Fig — Buc-GFP (green) in the Balbiani body of stage Ib oocytes before hexanediol treatment (A, C; 0 min) or after 30 min treatment with double conc. (10%; B, D). Stippled squares indicate the magnified area shown in panel C and D. Note the BucGFP fragments draining off the Blabiani body after HD treatment (D). Scale bar (A, B): 20 μm; (C, D): 1 μm. Cytoskeleton after Hexanediol treatment. Oocytes (E-L) or embryos (M-T) were treated for 30 min with hexanediol and stained for microtubules (β-tubulin) or microfilaments (filamentous Actin). Stippled boxes (E-H, M-P) indicate magnified area (I-L, Q-T). 2-cell embryos (M-T)are shown in animal view. Scale bars (E-H, Q-T): 20 μm. (I-L): 1μm. (M-P):100 μm. (TIF) [file pgen.1007696.s004.tif]

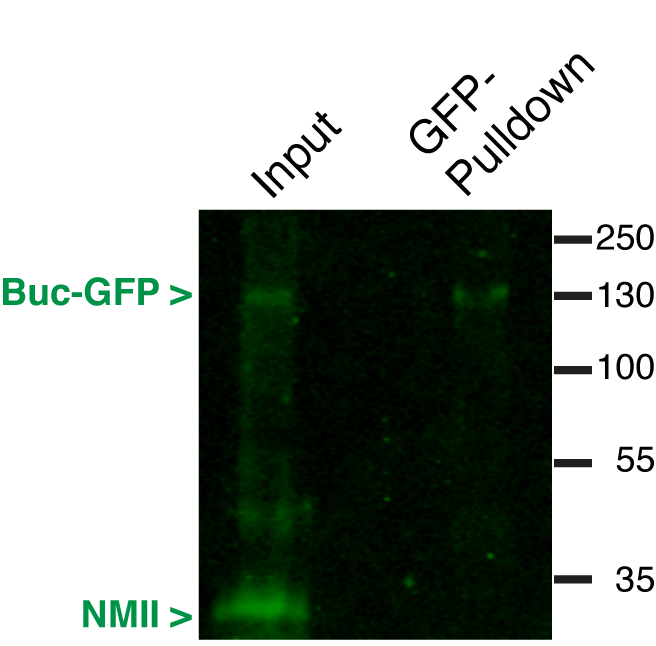

Supplement: S5 Fig — Western blot of Buc-GFP (green) and Myc-Non-muscle Myosin II (green; NMII; 20 kD) after in vitro translation (input = 40% of pulldown) and after GFP pulldown. Buc does not interact with NMII. (TIF) [file pgen.1007696.s005.tif]
