# Supplementary material for: Functional equivalence of germ plasm organizers
Source: PLoS Genet. 2018 Nov 6;14(11):e1007696. doi: 10.1371/journal.pgen.1007696 (PMC6219760; doi:10.1371/journal.pgen.1007696)
Supplement: S1 Table — Graph summarizing scores of global (white bar; Needleman-Wunsch) and local alignments (black bar; Smith-Waterman). Note that Buc-Osk alignments are equally low as the negative control (Buc-Dm Vasa), whereas ZfVasa and DmVasa show a characteristic score of two homologous sequences. Analysis of protein sequences with global pairwise alignments using the Needleman-Wunsch algorithm (A; http://www.ebi.ac.uk/Tools/psa/emboss_needle/; standard settings) or with local pairwise alignments using the Smith-Waterman algorithm (B; http://www.ebi.ac.uk/Tools/psa/emboss_water/; standard settings). Depicted are the percentages of similar and identical amino acids of two aligned protein sequences (sequences and raw data of sequence alignments in Supplementary Data 1). (PDF) [file pgen.1007696.s006.pdf]

Table S1: Bucky ball and Oskar do not share sequence homology.

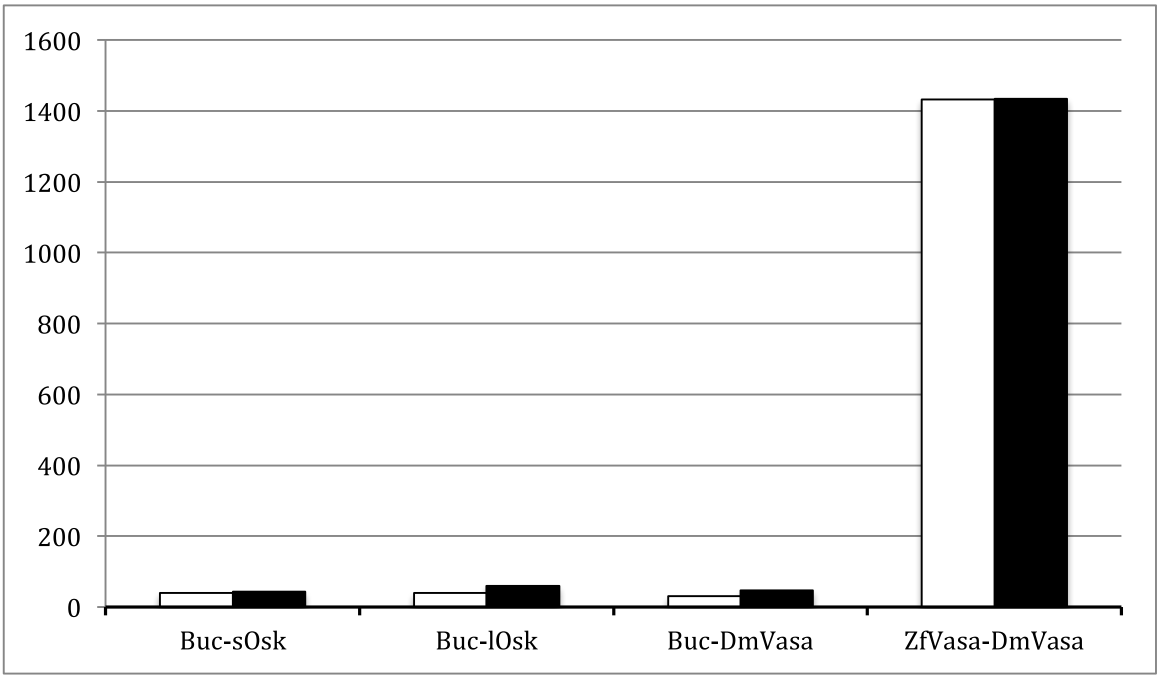

A) Global alignment with Needleman-Wunsch

Alignment	Bucky ball short Oskar (Isoform C)	Bucky ball long Oskar (Isoform A)	Zf Bucky ball Dm Vasa	Zf Vasa Dm Vasa
Seq. similarity [%]	11.5%	10.0%	18.5%	59.4%
Seq. identity [%]	6.3%	5.5%	10.8%	45.2%
score	39.0	39.0	30.5	1433

B) Local alignment with Smith-Waterman

Alignment	Bucky ball short Oskar (Isoform C)	Bucky ball long Oskar (Isoform A)	Zf Bucky ball Dm Vasa	Zf Vasa Dm Vasa
Seq. similarity [%]	30.6	35.9	29.9	59.8
Seq. identity [%]	17.1	17.7	18.4	45.7
score	43.0	59.5	46.5	1434
