# Supplementary material for: Functional equivalence of germ plasm organizers
Source: PLoS Genet. 2018 Nov 6;14(11):e1007696. doi: 10.1371/journal.pgen.1007696 (PMC6219760; doi:10.1371/journal.pgen.1007696)
Supplement: S2 Table — Homology search with conserved domains using Hidden-Markov-Models (www.HMMer.org) of the respective proteins did not reveal any conserved domains between Oskar and Bucky ball. Hits of the used HMM in the NCBI databases are shown with their corresponding E-value. (PDF) [file pgen.1007696.s007.pdf]

Table S2: Comparison of Buc and Osk with Hidden-Markov-Models.

Used Hidden-Markov-Model	Searched NCBI organism database	Significant hits	E-Value
Buc-HMM	*Danio rerio*	Bucky ball	9.4e-223
		zDazl	0.022
	Drosophila melanogaster	-	-
Osk-HMM	*Danio rerio*	Tdrd7A	1.4e-11
		Tdrd7A, isoform 1	3.2e-11
		Tdrd5	2.4e-07
		Tdrd7B	1e-06
		Her13	0.017
	Drosophila melanogaster	Oskar; isoform A	2.8e-223
		Oskar; isoform C	1.1e-205
		RE24380p	8.8e-198
		CG8920, isoform D /E	1.9e-07
		CG8920, isoform B / C	2.8e-07
		CG34007	0.00015
		Tejas / FI02030p	0.0048
		IP20666p	0.016

HOME | Login | PDBalert | Personal Databases | Contact | Tool Versions | Imprint | Disclaimer | Help

MAX-PLANCK-GESELLSCHAFT

Bioinformatics Toolkit
Max-Planck Institute for Developmental Biology

Quickfinder

Search Alignment Sequence Analysis 2ary Structure 3ary Structure Classification Utils

Show results of job:

Show results

Recent jobs:

7515734 HHPR

Select all Deselect all

Clear sel. Jobs

Delete sel. Jobs

queued
running
done
error

CS-BLAST HHblits HHpred HHsenser HMMER3 PatternSearch ProtBLAST PSI-BLAST SimShiftDB
New job Resubmit Resubmit_HMM Resubmit/HHsenser Realign Log Input-params Delete

HHpred - Results

Job-ID: 7515734 Date: 14:13 on Jan 27 2015

Help

Results | Histogram | Reduced alignment | Representative alignment | Full alignment
Create model Merge Q/T alignments Forward to PCoils Forward HMM Save Export

Color alignments
color only SS ◉ color alignments ○ color alignments

View alignment Activate Jalview

Note: click PDBalert to upload your query sequence and get alerted by email as soon as better PDB templates get available.

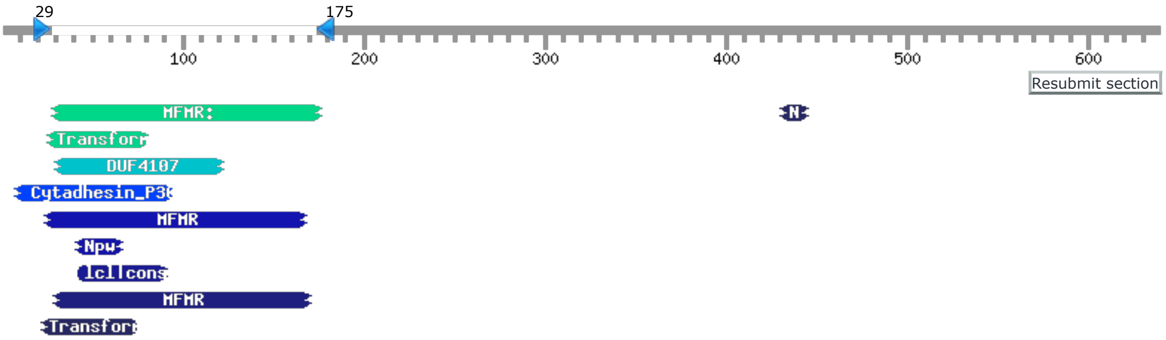

Query gi|292610748|ref|XP_002660872.1| (seq=MEGINNNSQP...GSMKKRGSRY Len=639 Neff=2.4 Nseqs=7)
Parameters score SS:yes search:local realign with MAP:no

No	Hit	Prob	E-value	P-value	Score	SS	Cols	Query HMM	Template HMM
1	PF07777 MFMR: G-box binding p	62.0	50	0.0005	35.6	6.5	127	29-175	19-185 (189)
2	pfam06495 Transformer Fruit fl	61.6	23	0.00022	37.6	4.0	50	27-79	117-180 (182)
3	pfam13389 DUF4107 Protein of u	58.1	17	0.00017	37.5	2.4	47	31-121	102-149 (158)
4	pfam07271 Cytadhesin_P30 Cytad	45.6	1.5E+02	0.0015	33.6	6.9	83	9-93	170-262 (279)
5	pfam07777 MFMR G-box binding p	34.0	2.9E+02	0.0029	30.6	6.7	124	25-167	45-179 (189)
6	pfam12622 NpwBP mRNA biogenesi	33.6	65	0.00064	28.7	1.9	23	42-65	6-29 (54)
7	lcl|consensus	31.3	81	0.00081	35.1	2.6	41	42-90	1-45 (228)
8	pfam07777 MFMR G-box binding p	28.8	4E+02	0.004	29.6	6.8	132	30-169	19-175 (189)
9	pfam05810 NinF NinF protein. T	24.9	54	0.00053	29.8	0.0	14	431-444	14-27 (58)
10	PF06495 Transformer: Fruit fl	24.5	1.7E+02	0.0017	32.0	3.3	47	23-74	135-182 (182)

No 1 InterPro PubMed

>PF07777 MFMR: G-box binding protein MFMR; InterPro: This region is found to the N terminus of IPR011616 from INTERPRO, which is a transcription factor domain. It is between 150 and 200 amino acids in length. The N-terminal half is rather rich in proline residues and has been termed the PRD (proline rich domain) [], whereas the C-terminal half is more polar and has been called the MFMR (multifunctional mosaic region). It has been suggested that this family is composed of three sub-families called A, B and C [], classified according to motif composition. It has been suggested that some of these motifs may be involved in mediating protein-protein interactions []. The MFMR region contains a nuclear localisation signal in bZIP opaque and GBF-2 []. The MFMR also contains a transregulatory activity in TAF-1. The MFMR in CPRF-2 contains cytoplasmic retention signals []. ;
GO: 0003677 DNA binding, 0006351 transcription, DNA-dependent, 0006355 regulation of transcription, DNA-dependent, 0005634 nucleus
Probab=61.96 E-value=50 Score=35.65 Aligned_cols=127 Identities=24% Similarity=0.234 Sum_probs=0.0

```
Q ss_pred              ecCCCCCCcccc-ccc-cccCCCccCCccc--------cCCCCCCCccccccCCccccccCCCCChhhhcccCCCCCCccc
Q gi|292610748|r    29 VQPPSQPYFMYQ-WPM-NPYGHYGFPGPAL--------HFGRPYMAPYQFMQYPGYVIPHAPMQPIDYRRINPHYPSVAS   98 (639)
Q Consensus         29 aQP~aQppf~~p-Wyl-npY~pYciPgpGF--------r~G~Py~p~Ysl~eYPGffvP~~Pv~~r~~RRFnp~~psP~f   98 (639)
                          +|........|| |-- ++|..=+ +-|+|         +..+|||             .-|++||.|-    |-...|-|++
T Consensus         19 ~~~~~~~~~~~ypDWs~mQAYyg~~-~~p~~f~s~va~sp~phPYM-----------WG~~q~mmPP----YGtP~pY~A~   82 (189)
T PF07777_consen    19 PPEDQPTPHVYPDWSAMQAYYGPG-APPPYFNSAVASSPQPHPYM-----------WGPQQPMMPP----YGTPVPYPAM   82 (189)
T ss_pred              CcCCCCCCccCCccHhhhccCCC-CCCcccCcccCCCCCCCCCcc-----------cCCCccccCC----CCCCCCCccc


Q ss_pred              c-cccccccCCC----------------------ccccccc-ccccccCcchHHHHhhhhh----hhccccccCCcccc-
Q gi|292610748|r    99 Y-DLRVRHHFQNA----------------------GMHRETA-CSEVQTDPSDSVNKLIDKI----ESLKACELGSDKG-  149 (639)
Q Consensus         99 y-ha~rf~Hy~~P----------------------gr~reTe-tkEtQTDP~q~enK~k~~~----d~~kgC~~G~~~g-  149 (639)
                     | |-..+.|=+-|                       +.  ||+ .|-...-=+-+..|+|+.+    -.++.++.|...+
T Consensus         83 YphGgvYAHP~mp~~s~p~~~~~~~~~s~~~~~~~tp~s~--E~~p~Kss~~kd~~~~KksKg~~g~~a~s~~n~~~gk~~~~~   160 (189)
T PF07777_consen    83 YPHGGVYAHPSMPPGSHPFSPYAMPSPETPAATPLST--ETDPGKSSGNKDKGSMKKSKGFDGGLAMSIKNGESGKTSGS   160 (189)
T ss_pred              cCCCccccCCCCCccccCCCCcccccccCCCCccc--ccccccCcCcccccccccccccccccccccccceeeccCCccCccccC


Q ss_pred              cCceeeecCC-CCcccccchhccccCC
Q gi|292610748|r   150 PNNVVSSTPD-VVQGEKLTRLNEDSNL  175 (639)
Q Consensus        150 ~~t~Vas~~~-~~~g~e~~~e~~d~s~  175 (639)
                     -.+...|.++ ++.  |-.++.-|.+.
T Consensus        161 s~n~~~Sqs~eSgs--egSSdgSD~Nt  185 (189)
T PF07777_consen   161 SANDGSSQSSESGS--EGSSDGSDGNT  185 (189)
T ss_pred              CCCCccCccccccc--cccccCcCcccc
```

No 2

>pfam06495 Transformer Fruit fly transformer protein. This family consists of transformer proteins from several Drosophila species and also from Ceratitis capitata (Mediterranean fruit fly). The transformer locus (tra) produces an RNA processing protein that alternatively splices the doublesex pre-mRNA in the sex determination hierarchy of Drosophila melanogaster.
Probab=61.64 E-value=23 Score=37.59 Aligned_cols=50 Identities=30% Similarity=0.667 Sum_probs=0.0

```
Q ss_pred                 EEecCCCCCC-cccccc-----ccc-cCCCccCCccccCCCCCCCCcc-------cccCCcccccCC
Q gi|292610748|r    27 FYVQPPSQPY-FMYQWP-----MNP-YGHYGFPGPALHFGRPYMAPYQ-------FMQYPGYVIPHA   79 (639)
Q Consensus         27 FYaQP~aQpp-f~~pWy-----lnp-Y~pYciPgpGFr~G~Py~p~Ys-------l~eYPGffvP~~   79 (639)
                         +|||=||+-| |.|-|.     ||+ |+++++.-+- |-=+|||+||.       |-.=|  |.||+
T Consensus        117 vpVpVPa~~y~yaYgwp~p~p~f~~M~~~~~y~~~p-RP~~Pyfapy~rppa~f~YR~pP--f~p~p  180 (182)
T pfam06495       117 VPVPVPAADYPYAYGWPPPAPQFSGMQGAFPYGMLP-RPVPPYFAPYPRPPAPFRYRAPP--FRPHP  180 (182)
T ss_pred                 eeccccCcccccccccCCCCccccccccccCCCCCC-CCCCccccCCCCCCCCcccccCCC--CCCCC
```

No 3

>pfam13389 DUF4107 Protein of unknown function (DUF4107). This family of putative proteins are found in Trichomonas vaginalis in large numbers. The function of this protein is unknown.
Probab=58.13 E-value=17 Score=37.53 Aligned_cols=47 Identities=34% Similarity=0.533 Sum_probs=0.0

```
Q ss_pred                 CCCCCCccccccccccCCCccCCccccCCCCCCCCccccCCccccCCCCChhhhcc-cCCCCCCcccccccccccCC
Q gi|292610748|r    31 PPSQPYFMYQWPMNPYGHYGFPGPALHFGRPYMAPYQFMQYPGYVIPHAPMQPIDYRR-INPHYPSVASYDLRVRHHFQN  109 (639)
Q Consensus         31 P~aQppf~~pWylnpY~pYciPgpGFr~G~Py~p~Ysl~eYPGffvP~~Pv~~r~~RR-Fnp~~psP~fyha~rf~Hy~~  109 (639)
                         +++||+      .|-||.|||+=|                                +-|-|  |+|------|||-
T Consensus        102 ~v~~P~------av~PY~pyGmy~-------------------------------~pyerPFDPktGGSNWyHY--------  138 (158)
T pfam13389       102 NVSSPP------AVLPYGPYGMYG-------------------------------NPYERPFDPLTGGSNWYHY--------  138 (158)
T ss_pred                 CCCCCC------CCCCcCCccccC-------------------------------CCcCCCCCCCcCCccceee--------


Q ss_pred                 Cccccccccccc
Q gi|292610748|r   110 AGMHRETACSEV  121 (639)
Q Consensus        110 Pgr~reTetkEt  121 (639)
                          ||+|++.-+|+
T Consensus        139 -Grrrk~vk~e~  149 (158)
T pfam13389       139 -GRRRKTVKLET  149 (158)
T ss_pred                 -ccchhhhHHHH
```

No 4

>pfam07271 Cytadhesin_P30 Cytadhesin P30/P32. This family consists of several Mycoplasma species specific Cytadhesin P32 and P30 proteins. P30 has been found to be membrane associated and localized on the tip organelle. It is thought that it is important in cytadherence and virulence.
Probab=45.63 E-value=1.5e+02 Score=33.63 Aligned_cols=83 Identities=33% Similarity=0.474 Sum_probs=0.0

```
Q ss_pred                 CCCCCCCCCccccCCCCceEEecCCCCCCcccccccccc-cCC--CccCCccccCCCCCCCCcc----cccCCc-ccccCCC
Q gi|292610748|r     9 QPMGVGQPHHPVNHTRPFFYVQPPSQPYFMYQWPMNP-YGH--YGFPGPALHFGRPYMAPYQ----FMQYPG-YVIPHAP   80 (639)
Q Consensus          9 p~~g~G~~~~~~~Nh~RPFFYaQP~aQppf~~pWylnp-Y~p--YciPgpGFr~G~Py~p~Ys----l~eYPG-ffvP~~P   80 (639)
                         .+-+-|.+-.+.-..||=|- |=+.|-+=+.|=.|-| |++  ==.|-|||+- +|+|.|-+    |..-|| |--|-+|
T Consensus        170 ~p~rf~~p~~PnMg~rPGfn-Qmpphm~gmpPn~mRPgf~~mpgm~PRPGf~~-~P~m~PnM~rpGF~P~Pg~~~~Pg~p  247 (279)
T pfam07271       170 NPQRFGFPMQPNMGMRPGFN-QMPPHMPGMPPNQMRPGFNPMPGMPPRPGFNQ-NPNMMPNMNRPGFRPQPGGFNHPGTP  247 (279)
T ss_pred                 CCCCCCCCCCCCcCCCCCcC-CCCCcCCCCCcccCCCCCCCCCCCCCCCCCCC-CCCCCCCCCCCCCCCCCCCCCCCCCC


Q ss_pred                 CChhhhcc--cCCCC
Q gi|292610748|r    81 MQPIDYRR--INPHY   93 (639)
Q Consensus         81 v~~r~~RR--Fnp~~   93 (639)
                         ++|-+--|  |||+.
T Consensus        248 ~~Pnm~~RpgF~P~~  262 (279)
T pfam07271       248 MGPNMQQRPGFNPNQ  262 (279)
T ss_pred                 CCCCccccCCCCCCCc
```

No 5

>pfam07777 MFMR G-box binding protein MFMR. This region is found to the N-terminus of the pfam00170 transcription factor domain. It is between 150 and 200 amino acids in length. The N-terminal half is rather rich in proline residues and has been termed the PRD (proline rich domain), whereas the C-terminal half is more polar and has been called the MFMR (multifunctional mosaic region). It has been suggested that this family is composed of three sub-families called A, B and C, classified according to motif composition. It has been suggested that some of these motifs may be involved in mediating protein-protein interactions. The MFMR region contains a nuclear localisation signal in bZIP opaque and GBF-2. The MFMR also contains a transregulatory activity in TAF-1. The MFMR in CPRF-2 contains cytoplasmic retention signals.
Probab=33.98 E-value=2.9e+02 Score=30.57 Aligned_cols=124 Identities=27% Similarity=0.319 Sum_probs=0.0

```
Q ss_pred                 ceEEecC-CCCCCccccccc-cc-cCCCccCCccccCCCCCCCCCcccccCCccccCCCCChhhhcccCCCCCCCcccccc
Q gi|292610748|r    25 PFFYVQP-PSQPYFMYQWPM-NP-YGHYGFPGPALHFGRPYMAPYQFMQYPGYVIPHAPMQPIDYRRINPHYPSVASYDL  101 (639)
Q Consensus         25 PFFYaQP-~aQppf~~pWyl-np-Y~pYciPgpGFr~G~Py~p~Ysl~eYPGffvP~~Pv~~r~~RRFnp~~psP~fyha  101 (639)
                          |||+.=- .+.+|-+|=|-- ++ -.|||.|=        ||.+.|.     ||=|.-|+.|.+..+     |+-|.+|-.
T Consensus         45 ~~f~s~va~sp~phPYMWG~~qpmmpPYGtP~-------pY~A~Yp----~GgvYAHP~mp~gs~----p~~~~~~~s--  107 (189)
T pfam07777        45 PYFNSSVASSPQPHPYMWGPQQPMMPPYGTPP-------PYAAMYP----PGGVYAHPSMPPGSH----PFSPYAMPS--  107 (189)
T ss_pred                 cccCccccCCCCCCccccCCCCCCCCCCCCCC-------CccccC----CCcccCCCCCccc----cCCcccccc--


Q ss_pred                 ccccccCCCccccccccccccccCcchHHHHhhhhh------hhccccccCCccc-cCceeeecCC-CCcccccc
Q gi|292610748|r   102 RVRHHFQNAGMHRETACSEVQTDPSDSVNKLIDKI------ESLKACELGSDKG-PNNVVSSTPD-VVQGEKLT  167 (639)
Q Consensus        102 ~rf~Hy~~Pgr~reTetkEtQTDP~q~enK~k~~~------d~~kgC~~G~~~g-~~t~Vas~~~-~~~g~e~~  167 (639)
                         +.----..-..  ||..|-.-.-=+-+..|+|+.+       -.++.++.|...+ -.+...|+++ ++.+-.+.
T Consensus        108 ~~~~~~tp~s~--E~~~Kss~~kd~~~Kk~Kg~~g~~gla~s~~n~~~gk~~~~s~n~~~SqS~ESgsegSSd  179 (189)
T pfam07777       108 AEVPGSTPLSM--ETDAKSSDNKDKGSIKKSKGSDGSLGLAMSGKNGESGKASGSSANGGSSQSSESGSDGSSE  179 (189)
T ss_pred                 cccCCCCcccc--ccCCCCcCcccccchhhhccccccccceeeccCCcCCcccCCCCCCCCCCCcccccccccc
```

No 6

>pfam12622 NpwBP mRNA biogenesis factor. The full-length Wbp11 proteins carry several copies of a PPGPPP motif throughout their length. This motif is thought to be necessary for folding of the molecule as it helps to bind

the WW domain, Wbp11, pfam09429. This domain together with Wbp11 may function as components of an mRNA factory in the nucleus.
Probab=33.58 E-value=65 Score=28.73 Aligned_cols=23 Identities=26% Similarity=0.417 Sum_probs=0.0

```
Q ss_pred              cccc-cCCCccCCccccCCCCCCCC
Q gi|292610748|r   42 PMNP-YGHYGFPGPALHFGRPYMAP   65 (639)
Q Consensus        42 ylnp-Y~pYciPgpGFr~G~Py~p~   65 (639)
                      |.|| +||||.+=+|||+ -||+|.
T Consensus         6 yydP~lNP~G~~P~g~~~-~~~~p~   29 (54)
T pfam12622         6 YYDPELNPLGKPPPGYKN-IPYNPY   29 (54)
T ss_pred              ecCCccCCCCCCCCcccc-cccCCc
```

No 7

>lcl|consensus
Probab=31.29 E-value=81 Score=35.15 Aligned_cols=41 Identities=29% Similarity=0.435 Sum_probs=0.0

```
Q ss_pred              ccccccCCCccCCccccCCCCCCCCccccCCccccCCCCChhhhcc----cC
Q gi|292610748|r   42 PMNPYGHYGFPGPALHFGRPYMAPYQFMQYPGYVIPHAPMQPIDYRR----IN   90 (639)
Q Consensus        42 ylnpY~pYciPgpGFr~G~Py~p~Ysl~eYPGffvP~~Pv~~r~~RR----Fn   90 (639)
                      |||. .||.++|-||..  |||.  .+|..||.-++   +-+|-+||     |+
T Consensus         1 ~~~~-~~~~~~~~~~~fa~--p~~d--~~h~~~~vP~~---~~pRkqRRERTtFt   45 (228)
T lcl|consensus     1 YLNQ-PPYAVNGLAFAA--PYMD--LLHPKPGVPYS---SGPRKQRRERTTFT   45 (228)
T ss_pred              CCCC-CCCCcCcccccc--cccc--ccccCCCCCcC---ccchhcccccceec
```

No 8

>pfam07777 MFMR G-box binding protein MFMR. This region is found to the N-terminus of the pfam00170 transcription factor domain. It is between 150 and 200 amino acids in length. The N-terminal half is rather rich in proline residues and has been termed the PRD (proline rich domain), whereas the C-terminal half is more polar and has been called the MFMR (multifunctional mosaic region). It has been suggested that this family is composed of three sub-families called A, B and C, classified according to motif composition. It has been suggested that some of these motifs may be involved in mediating protein-protein interactions. The MFMR region contains a nuclear localisation signal in bZIP opaque and GBF-2. The MFMR also contains a transregulatory activity in TAF-1. The MFMR in CPRF-2 contains cytoplasmic retention signals.
Probab=28.82 E-value=4e+02 Score=29.61 Aligned_cols=132 Identities=20% Similarity=0.222 Sum_probs=0.0

```
Q ss_pred              cCCCCCCcccc-ccc-cccCCCcc-CCccc----cCCCCCCCCcc----cccCCccccCCCCChhhhcc--cCCCCCCc
Q gi|292610748|r   30 QPPSQPYFMYQ-WPM-NPYGHYGF-PGPAL----HFGRPYMAPYQ----FMQYPGYVIPHAPMQPIDYRR--INPHYPSV   96 (639)
Q Consensus        30 QP~aQppf~~p-Wyl-npY~pYci-PgpGF----r~G~Py~p~Ys----l~eYPGffvP~~Pv~~r~~RR--Fnp~~psP   96 (639)
                      |+.......|| |-- ++|  ||. +-|+|     -...|-=+||+    -+--|-|-.|++  |+-+|=-  .-+||.-|
T Consensus        19 ~~~~~~~~~~yPDWs~mQAY--yg~~~~p~~f~s~va~sp~phPYMWG~~qpmmPYGtP~p--Y~A~Yp~GgvYAHP~mp   94 (189)
T pfam07777        19 QEDTPTPTVYPDWSAMQAY--YGPRPPPPYFNSSVASSPQPHPYMWGPQQPMMPPYGTPPP--YAAMYPPGGVYAHPSMP   94 (189)
T ss_pred              CCCCCCCccCcchHHhhcc--cCCCCCCccccCccccCCCCCCCcccCCCCCCCCCCCCCC--cccccCCCccccCCCCC


Q ss_pred              cccccccccCCCcccccc---ccccccCcchHHHHhhhhhhccccc--------cCCccccCceeeecCC-CCccc
Q gi|292610748|r   97 ASYDLRVRHHFQNAGMHRET---ACSEVQTDPSDSVNKLIDKIESLKACE--------LGSDKGPNNVVSSTPD-VVQGE  164 (639)
Q Consensus        97 ~fyha~rf~Hy~~Pgr~reT---etkEtQTDP~q~enK~k~~~d~~kgC~--------~G~~~g~~t~Vas~~~-~~~g~  164 (639)
                      +=.|.  |-||..+..  ++   .---+-||-+-.+||-+..+.++||.+         .||...-.+.-+|.-+ .++--
T Consensus        95 ~gs~p--~~~~~~~~s~~~--~~~~tp~s~E~~~Kss~~kd~~~~Kk~Kg~~g~~gla~s~~n~~~gk~~~~s~n~~~SqS~  170 (189)
T pfam07777        95 PGSHP--FSPYAMPSA--EVPGSTPLSMETDAKSSDNKDKGSIKKSKGSDGSLGLAMSGKNGESGKASGSSANGGSSQSS  170 (189)
T ss_pred              Ccccc--CCcccccc--ccCCCCccccCCCCCcccchhhhcccccccccceeeccCCcCCccccCCCCCCcccc


Q ss_pred              cccch
Q gi|292610748|r  165 KLTRL  169 (639)
Q Consensus       165 e~~~e  169 (639)
                      |+-+|
T Consensus       171 ESgse  175 (189)
T pfam07777       171 ESGSD  175 (189)
T ss_pred              ccccc
```

No 9

>pfam05810 NinF NinF protein. This family consists of several bacteriophage NinF proteins as well as related sequences from Escherichia coli.
Probab=24.93 E-value=54 Score=29.83 Aligned_cols=14 Identities=43% Similarity=0.945 Sum_probs=0.0

```
Q ss_pred              cchhhhhhhhhhhh
Q gi|292610748|r  431 SVCQKTCACCGSTL  444 (639)
Q Consensus       431 ~~~~~~cacCgk~l  444 (639)
                      ||.|-+||.|||-|
T Consensus        14 Sv~RaLCA~C~kqL   27 (58)
T pfam05810        14 SIARALCAGCGKQL   27 (58)
T ss_pred              HHHHHHHhCccccc
```

No 10

>PF06495 Transformer: Fruit fly transformer protein; InterPro: This family consists of transformer proteins from several Drosophila species and also from Ceratitis capitata (Mediterranean fruit fly). The transformer locus (tra) produces an RNA processing protein that alternatively splices the doublesex pre-mRNA in the sex determination hierarchy of Drosophila melanogaster [].; GO: 0006397 mRNA processing, 0046660 female sex differentiation, 0005634 nucleus
Probab=24.50 E-value=1.7e+02 Score=32.00 Aligned_cols=47 Identities=30% Similarity=0.576 Sum_probs=0.0

```
Q ss_pred              CCceEEecCCCCCCcccccccccc-CCCccCCccccCCCCCCCCccccCCcc
Q gi|292610748|r   23 TRPFFYVQPPSQPYFMYQWPMNPY-GHYGFPGPALHFGRPYMAPYQFMQYPGY   74 (639)
Q Consensus        23 ~RPFFYaQP~aQppf~~pWylnpY-~pYciPgpGFr~G~Py~p~Ysl~eYPGf   74 (639)
                      +.+-|=++=.+-+|-|-|-.|+|| +||+.|-+-|+.+.   ||  |+.-|.|
T Consensus       135 ~~~~~~~~m~~~~~y~~~~~~P~~p~~apy~~~p~p~ryr~----~p--f~~~pr~  182 (182)
T PF06495_consen  135 PAPQFNPMQGAYPYQMPPRPMPPYFAPYPRPPAPFRYRA---PP--FRPHPRF  182 (182)
T ss_pred              ccccccccccccccCCCCCCcccCccCCCCCCCCCccccC---CC--CCCCCCC
```

Done!

Please cite as appropriate:

***HHpred*: Söding, J. (2005) Protein homology detection by HMM-HMM comparison. Bioinformatics 21: 951-960.**

PSIPRED: Jones, D.T. (1999) Protein secondary structure prediction based on position-specific scoring matrices. JMB 292: 195-202.

Interpro: Mulder NJ, Apweiler R, Attwood TK, Bairoch A, Bateman A, Binns D, Bradley P, Bork P, Bucher P, Cerutti L, Copley R, Courcelle E, Das U, Durbin R, Fleischmann W, Gough J, Haft D, Harte N, Hulo N, Kahn D, Kanapin A, Krestyaninova M, Lonsdale D, Lopez R, Letunic I, Madera M, Maslen J, McDowall J, Mitchell A, Nikolskaya AN, Orchard S, Pagni M, Ponting CP, Quevillon E, Selengut J, Sigrist CJ, Silventoinen V, Studholme DJ, Vaughan R, Wu CH. (2005) InterPro, progress and status in 2005. NAR 33: D201-205.

Pfam: Bateman A, Coin L, Durbin R, Finn RD, Hollich V, Griffiths-Jones S, Khanna A, Marshall M, Moxon S, Sonnhammer EL, Studholme DJ, Yeats C, Eddy SR. (2004) The Pfam protein families database. NAR 32: D138-141.

© 2008-2014, **Dept. of Protein Evolution at the Max Planck Institute for Developmental Biology**, Tübingen Release-2.18.1

HOME

MAX-PLANCK-GESELLSCHAFT

| Login | PDBalert | Personal Databases | Contact | Tool Versions | Imprint | Disclaimer | Help

Quickfinder

Bioinformatics Toolkit
Max-Planck Institute for Developmental Biology

Search Alignment Sequence Analysis 2ary Structure 3ary Structure Classification Utils

CS-BLAST HHblits HHpred HHsenser HMMER3 PatternSearch ProtBLAST PSI-BLAST SimShiftDB

New job Resubmit Resubmit_HMM Resubmit/HHsenser Realign Log Input-params Delete

Show results of job:

Show results

Recent jobs:

	6856982	HHPR
	7515734	HHPR

Select all Deselect all

Clear sel. Jobs

Delete sel. Jobs

queued
running
done
error

HHpred - Results Job-ID: 6856982 Date: 12:02 on Jan 28 2015

Help

Results | Histogram | Reduced alignment | Representative alignment | Full alignment

Create model Merge Q/T alignments Forward to PCoils Forward HMM Save Export

Color alignments

color only SS color alignments color alignments

View alignment Activate Jalview

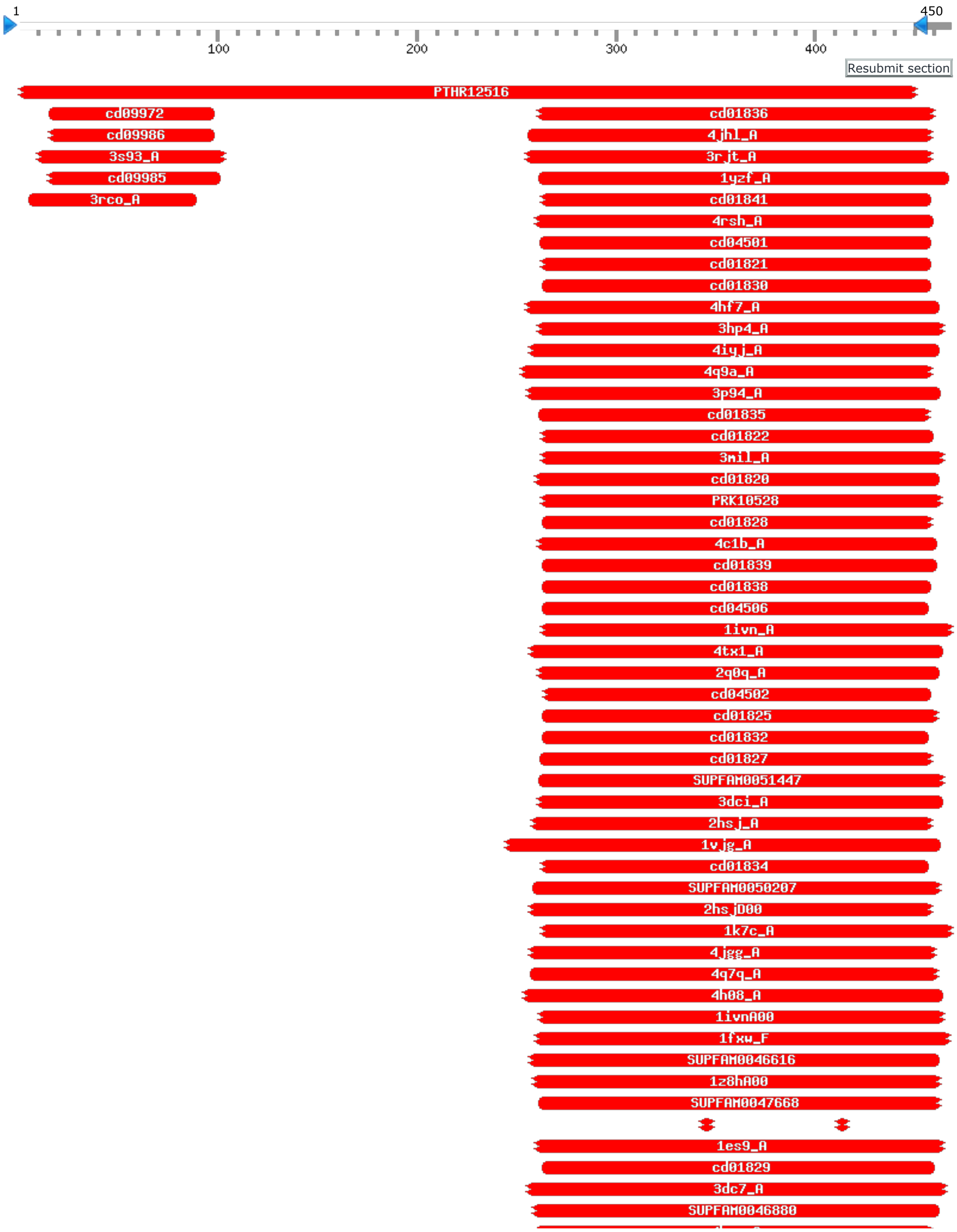

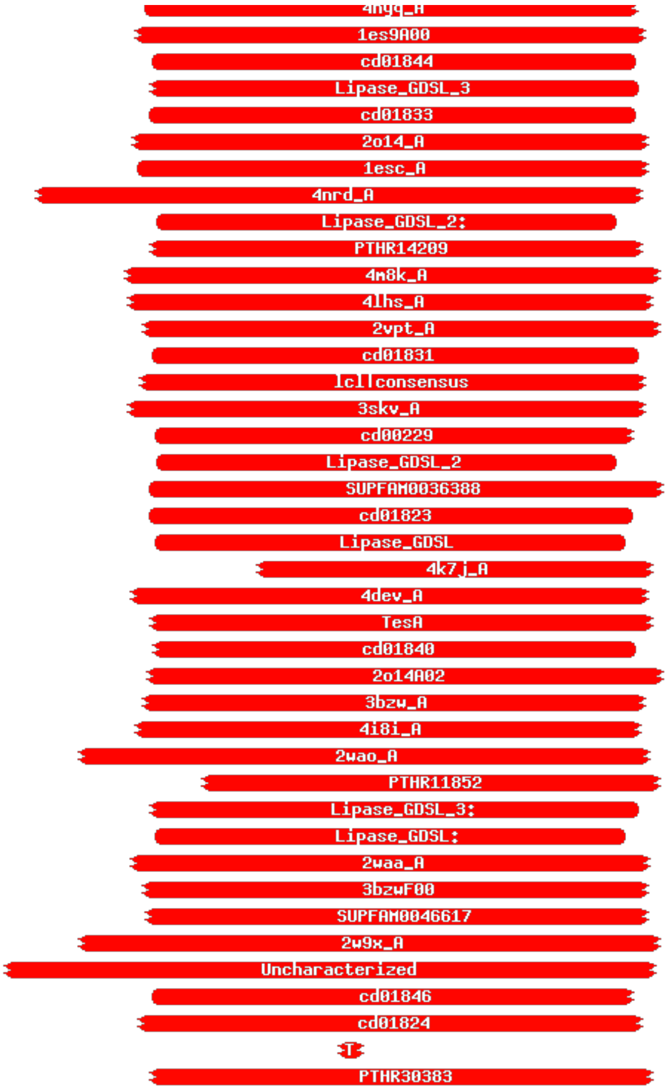

Query gi|45553317|ref|NP_996186.1| (seq=MTIIESNYIS...LQVIETSLEY Len=468 Neff=7.0 Nseqs=280)
Parameters score SS:yes search:local realign with MAP:no

No	Hit	Prob	E-value	P-value	Score	SS	Cols	Query HMM	Template HMM
1	PTHR12516 .	100.0	3.3E-33	2.3E-38	282.7	-8.2	446	1-450	119-581 (582)
2	cd09972 LOTUS_TDRD_OSKAR The f	99.9	3E-26	2.1E-31	191.3	9.9	83	15-98	1-87 (87)
3	cd01836 FeeA_FeeB_like SGNH_hy	99.9	6E-25	4.2E-30	209.0	18.9	179	261-459	2-190 (191)
4	cd09986 LOTUS_1_TDRD7 The firs	99.9	4.9E-26	3.5E-31	188.2	9.9	82	16-98	2-88 (88)
5	4jhl_A Acetyl xylan esterase;	99.9	8.9E-25	6.3E-30	208.4	19.9	198	256-458	1-211 (219)
6	3s93_A Tudor domain-containing	99.9	2E-26	1.4E-31	197.6	7.6	93	10-103	4-101 (102)
7	cd09985 LOTUS_1_TDRD5 The firs	99.9	1.1E-25	8E-31	189.3	10.7	86	15-101	5-95 (95)
8	3rjt_A Lipolytic protein G-D-S	99.9	1.1E-24	8.1E-30	206.9	18.9	199	255-458	2-213 (216)
9	1yzf_A Lipase/acylhydrolase; s	99.9	6.8E-25	4.8E-30	205.5	16.6	190	261-467	1-195 (195)
10	cd01841 NnaC_like NnaC (CMP-Ne	99.9	1.6E-24	1.1E-29	202.6	17.8	167	263-458	2-174 (174)
11	4rsh_A Lipolytic protein G-D-S	99.9	1.1E-24	7.6E-30	203.3	16.6	174	260-459	3-179 (179)
12	cd04501 SGNH_hydrolase_like_4	99.9	3.9E-24	2.8E-29	200.5	19.9	177	262-458	1-183 (183)
13	cd01821 Rhamnogalacturan_acety	99.9	3.3E-24	2.3E-29	205.7	18.8	179	263-458	2-198 (198)
14	cd01830 XynE_like SGNH_hydrola	99.9	3.8E-24	2.7E-29	206.2	17.8	185	263-458	1-204 (204)
15	4hf7_A Putative acylhydrolase;	99.9	1.7E-24	1.2E-29	206.1	14.3	181	255-462	20-209 (209)
16	3hp4_A GDSL-esterase; psychrot	99.9	1E-23	7.2E-29	197.9	19.2	178	261-464	2-184 (185)
17	4iyj_A GDSL-like protein; PF13	99.9	1.5E-24	1.1E-29	207.0	12.6	179	257-462	25-212 (212)
18	4q9a_A TAT pathway signal sequ	99.9	6.7E-24	4.8E-29	205.8	16.9	202	253-458	11-226 (229)
19	3p94_A GDSL-like lipase; serin	99.9	2.8E-24	2E-29	204.0	13.8	180	256-463	17-204 (204)
20	cd01835 SGNH_hydrolase_like_3	99.9	1.4E-23	1E-28	200.1	18.0	179	261-457	1-192 (193)
21	cd01822 Lysophospholipase_L1_l	99.9	2.7E-23	1.9E-28	193.9	19.5	173	263-459	2-177 (177)
22	3mil_A Isoamyl acetate-hydroly	99.9	1.9E-23	1.3E-28	203.1	18.8	188	263-464	5-213 (240)
23	cd01820 PAF_acetylesterase_lik	99.9	1.4E-23	9.6E-29	204.4	17.8	177	260-462	31-214 (214)
24	PRK10528 multifunctional acyl-	99.9	2.2E-23	1.5E-28	199.8	18.6	173	263-463	12-188 (191)
25	cd01828 sialate_O-acetylestera	99.9	3.3E-23	2.3E-28	192.6	18.8	164	263-458	1-168 (169)
26	4c1b_A ZFL2-1 ORF1P, ORF1-enco	99.9	1.7E-23	1.2E-28	193.9	16.7	163	261-461	3-171 (171)
27	cd01839 SGNH_arylesterase_like	99.9	3E-23	2.1E-28	200.5	18.1	181	263-461	1-208 (208)
28	cd01838 Isoamyl_acetate_hydrol	99.9	3E-23	2.2E-28	196.7	17.8	174	263-458	1-199 (199)
29	cd04506 SGNH_hydrolase_YpmR_li	99.9	1.8E-23	1.3E-28	200.6	16.3	179	263-457	1-204 (204)
30	1ivn_A Thioesterase I; hydrola	99.9	3.7E-23	2.6E-28	195.4	17.7	179	263-468	3-184 (190)
31	4tx1_A Esterase; SGNH-hydrolas	99.9	1.5E-23	1E-28	202.4	14.7	187	257-464	9-225 (225)
32	2q0q_A ARYL esterase; SGNH hyd	99.9	2.4E-23	1.7E-28	199.4	15.5	181	261-462	2-216 (216)
33	cd04502 SGNH_hydrolase_like_7	99.9	8.4E-23	6E-28	191.4	17.9	164	264-458	2-171 (171)
34	cd01825 SGNH_hydrolase_peri1 S	99.9	4.1E-23	2.9E-28	195.4	15.5	173	263-461	1-188 (189)
35	cd01832 SGNH_hydrolase_like_1	99.9	1.4E-22	1E-27	190.4	18.3	180	263-457	1-185 (185)
36	cd01827 sialate_O-acetylestera	99.9	1.6E-22	1.1E-27	192.3	18.5	173	262-458	1-187 (188)
37	SUPFAM0051447 c.23.10 SGNH hyd	99.9	6.9E-22	4.9E-27	174.0	20.6	187	261-464	1-192 (195)
38	3dci_A Arylesterase; SGNH_hydr	99.9	9.8E-23	6.9E-28	200.7	15.7	183	261-464	23-232 (232)
39	3rco_A Tudor domain-containing	99.9	1.4E-23	9.8E-29	174.9	7.9	84	5-89	1-89 (89)
40	2hsj_A Putative platelet activ	99.9	8.9E-23	6.3E-28	195.5	14.6	172	258-458	31-213 (214)

41	1vjg_A	Putative lipase from th	99.9	9.4E-23	6.7E-28	196.6	13.4	201	245-463	4-218 (218)
42	cd01834	SGNH_hydrolase_like_2	99.9	6.7E-22	4.8E-27	184.9	18.3	176	263-457	3-191 (191)
43	SUPFAM0050207	c.23.10 SGNH hyd	99.9	2.9E-21	2.1E-26	170.8	20.5	187	258-462	1-199 (201)
44	2hsjD00		99.9	9.7E-22	6.9E-27	191.1	17.8	173	257-458	30-213 (214)
45	1k7c_A	Rhamnogalacturonan acet	99.9	1.4E-22	9.8E-28	195.7	11.2	186	263-468	2-222 (233)
46	4jgg_A	Esterase TESA; alpha/be	99.9	1.1E-21	8E-27	186.8	16.7	180	257-460	17-199 (207)
47	4q7q_A	Lipolytic protein G-D-S	99.9	8.3E-23	5.9E-28	201.4	7.2	192	257-461	1-258 (266)
48	4h08_A	Putative hydrolase; GDS	99.9	1.3E-21	9.3E-27	186.1	14.9	179	254-464	13-200 (200)
49	1ivnA00		99.9	1.2E-20	8.7E-26	180.9	21.5	176	262-464	2-180 (190)
50	1fxw_F	Alpha2, platelet-activa	99.9	1.2E-21	8.4E-27	189.3	13.9	178	260-467	38-222 (229)
51	SUPFAM0046616	c.23.10 SGNH hyd	99.9	1.1E-20	7.9E-26	168.5	18.5	176	257-462	30-212 (212)
52	1z8hA00		99.9	1.5E-20	1E-25	182.1	20.4	186	259-462	2-199 (202)
53	SUPFAM0047668	c.23.10 SGNH hyd	99.9	4.1E-20	2.9E-25	160.6	19.6	175	261-462	1-178 (179)
54	TIGR03304	TIGR03304 outer memb	99.8	3.2E-23	2.2E-28	7.0	0.0	6	343-348	2-7 (10)
55	1es9_A	PAF-AH, platelet-activa	99.9	1.3E-21	8.9E-27	189.5	10.9	175	260-464	37-218 (232)
56	cd01829	SGNH_hydrolase_peri2 S	99.9	6.7E-21	4.8E-26	182.7	15.6	170	263-460	1-200 (200)
57	3dc7_A	Putative uncharacterize	99.9	3.3E-22	2.3E-27	194.9	5.4	189	256-465	16-227 (232)
58	SUPFAM0046880	c.23.10 SGNH hyd	99.9	4.2E-20	3E-25	164.6	18.1	174	259-462	32-212 (212)
59	4hyq_A	Phospholipase A1; glyce	99.9	7E-22	4.9E-27	190.3	6.6	189	260-458	1-235 (236)
60	1es9A00		99.9	4.5E-20	3.2E-25	180.6	18.8	175	257-461	34-215 (216)
61	cd01844	SGNH_hydrolase_like_6	99.9	1.6E-20	1.2E-25	176.8	15.0	167	263-458	1-177 (177)
62	pfam14606	Lipase_GDSL_3 GDSL-l	99.9	3.5E-20	2.5E-25	175.3	16.7	166	263-459	3-178 (178)
63	cd01833	XynB_like SGNH_hydrola	99.9	1.6E-20	1.2E-25	172.8	13.7	147	262-458	1-157 (157)
64	2o14_A	Hypothetical protein YX	99.8	2.8E-20	2E-25	196.2	14.9	192	256-462	157-363 (375)
65	1esc_A	Esterase; 2.10A {Strept	99.8	1.9E-20	1.4E-25	189.8	10.1	197	257-462	1-304 (306)
66	4nrd_A	Uncharacterized protein	99.8	1.4E-20	9.8E-26	183.8	6.9	175	217-460	9-225 (227)
67	PF13472	Lipase_GDSL_2: GDSL-l	99.8	2.2E-19	1.6E-24	165.8	14.4	166	265-450	1-179 (179)
68	PTHR14209	ISOAMYL ACETATE-HYDR	99.8	2.7E-20	1.9E-25	188.5	7.1	176	263-460	26-232 (277)
69	4m8k_A	Hypothetical protein, G	99.8	6.8E-21	4.8E-26	186.9	1.8	177	253-467	12-231 (236)
70	4lhs_A	Uncharacterized protein	99.8	3.8E-19	2.7E-24	185.3	14.0	179	254-464	149-338 (343)
71	2vpt_A	Lipolytic enzyme; ester	99.8	3.5E-19	2.5E-24	172.8	12.6	179	260-467	4-204 (215)
72	cd01831	Endoglucanase_E_like E	99.8	1.7E-18	1.2E-23	162.7	15.6	160	263-459	1-169 (169)
73	lcl|consensus		99.8	2.1E-18	1.5E-23	163.7	13.7	181	259-461	4-211 (245)
74	3skv_A	SSFX3; jelly roll, GDSL	99.8	1.5E-18	1.1E-23	183.7	14.5	188	254-461	179-378 (385)
75	cd00229	SGNH_hydrolase SGNH_hy	99.8	5.9E-18	4.2E-23	154.9	16.3	173	264-456	1-186 (187)
76	pfam13472	Lipase_GDSL_2 GDSL-l	99.8	5.8E-18	4.1E-23	154.2	15.4	166	265-450	1-173 (173)
77	SUPFAM0036388	c.23.10 SGNH hyd	99.8	4E-17	2.8E-22	147.4	20.0	187	262-468	1-222 (233)
78	cd01823	SEST_like SEST_like. A	99.8	2.8E-18	2E-23	171.7	13.5	188	262-457	1-259 (259)
79	TIGR04294	TIGR04294 prepilin-t	99.6	1.2E-19	8.3E-25	9.1	0.0	6	411-416	7-12 (16)
80	pfam00657	Lipase_GDSL GDSL-lik	99.7	4.4E-17	3.1E-22	154.8	13.0	177	264-454	1-219 (219)
81	4k7j_A	GDSL-like lipase/acylhy	99.7	4.7E-17	3.4E-22	172.3	10.0	155	306-464	206-374 (379)
82	4dev_A	Acetyl-xylan esterase E	99.7	7.2E-17	5.1E-22	172.6	11.0	181	255-462	160-404 (408)
83	COG2755	TesA Lysophospholipase	99.7	6.8E-16	4.8E-21	150.4	16.4	189	263-464	10-214 (216)
84	cd01840	SGNH_hydrolase_yrhL_li	99.7	5.8E-16	4.1E-21	143.6	14.3	144	264-458	2-150 (150)
85	2o14A02		99.7	6.3E-15	4.5E-20	144.4	20.5	192	262-468	2-208 (214)
86	3bzw_A	Putative lipase; protei	99.7	2.2E-16	1.6E-21	156.5	9.3	183	260-461	25-262 (274)
87	4i8i_A	Hypothetical protein; 5	99.7	2.9E-15	2.1E-20	151.8	15.3	178	257-459	6-224 (271)
88	2wao_A	Endoglucanase E; plant	99.7	2E-15	1.4E-20	157.3	12.6	211	234-463	95-332 (341)
89	PTHR11852	PLATELET-ACTIVATING	99.6	4.9E-16	3.5E-21	194.8	9.0	175	284-467	1714-1895(1956)
90	PF14606	Lipase_GDSL_3: GDSL-l	99.6	4E-15	2.8E-20	140.8	11.0	166	263-459	3-178 (178)
91	PF00657	Lipase_GDSL: GDSL-lik	99.6	4.2E-15	3E-20	144.5	11.3	182	264-454	1-234 (234)
92	2waa_A	Acetyl esterase, xylan	99.6	3.4E-15	2.4E-20	156.4	10.7	182	255-463	126-338 (347)
93	3bzwF00		99.6	9.6E-14	6.8E-19	139.3	17.8	184	260-462	6-244 (255)
94	SUPFAM0046617	c.23.10 SGNH hyd	99.6	2E-13	1.4E-18	129.2	15.4	197	261-462	2-301 (302)
95	2w9x_A	AXE2A, CJCE2B, putative	99.6	2.4E-14	1.7E-19	151.6	8.9	207	234-467	115-362 (366)
96	COG2845	Uncharacterized protei	99.5	1.6E-12	1.1E-17	131.3	15.4	211	204-465	82-324 (354)
97	cd01846	fatty_acyltransferase_	99.5	2E-12	1.4E-17	131.2	15.5	187	263-456	1-269 (270)
98	cd01824	Phospholipase_B_like P	99.4	1.5E-11	1.1E-16	126.5	16.4	198	258-460	6-285 (288)
99	TIGR01167	TIGR01167 LPXTG cell	99.1	1.4E-13	9.6E-19	6.8	0.0	9	339-347	10-18 (34)
100	PTHR30383	PERIPLASMIC BROAD-SP	99.4	3.4E-12	2.4E-17	118.3	7.8	178	263-464	38-218 (224)

No 1

>template .

```
Probab=99.97  E-value=3.3e-33  Score=282.69  Aligned_cols=446  Identities=98%  Similarity=1.499  Sum_probs=0.0

Q ss_pred              Cccccchhh-hhhhHHHHHHHHHHHHeecCCCCCcCHHHHHHHHHHhCCcCCCcccHHHHHhcCCCeEEEEeCCCCEEE
Q gi|45553317|re    1 MTIIESNYIS-VREEYPDIDSEVRAILLSHAQNGITISSIKSEYRKLTGNPFPLHDNVTDFLLTIPNVTAECSESGKRIF   79 (468)
Q Consensus         1 ~~~~~~~~~~-~~~~~~~~~~~~irs~l~s~~k~g~~~~~l~~dy~~~~g~~~p~~~~~~~~~l~~~~~~~~~~~~~g~~~~   79 (468)
                       |||++++|+. +++|+++++++++|++++++.++|++++.++.+|+.++|+++|++.++.+|++++|.++..|.+.|..++
T Consensus       119 ~~~~~~~~~~~~~~~~~~~~~~~~~~~~~~~~~~~~~~~~~~~~~~~~~~~~~~~~~~~~~~~~~~~~~~~~~~~~~~~~~~  198 (582)
T PTHR12516       119 MTIIESNYISVVREEYPDIDSEVRAILLSHAQNGITISSIKSEYRKLTGNPFPLHDNVTDFLLTIPNVTAECSESGKRIF  198 (582)
T ss_pred              HHHHHHhHHHHHHHhhhhhHHHHHHHHHHHhhhhhhhhhHHHHHHHhhccccchhhHHHHHHHHhhhHHHHHHhhhhHHHHH


Q ss_pred              EEecCccchHHHHHHHHHhccCCccccCCccccccccCCCCccccCCccccCC--------CCcccccccccccccccc
Q gi|45553317|re   80 NLKASLKNGHLLDMVLNQKERTSDYSSGAPSLENIPRAPPRYWKNPFKRRALS--------QLNTSPRTVPKITDEKTKD  151 (468)
Q Consensus        80 ~~~~~~~~t~hi~~mv~~Qk~~~~~~~~~~~~~~~~~~~~~~~~~r~~~~~~~~~r~~~~--------~~~~~~~~~~~~~~~~~~~~~~~  151 (468)
                       ..+..-+..|+.+||..||...+.+.++.++....+..+|+.|..+.+|+...        .+.......+.++++..+.
T Consensus       199 ~~~~~~~~~~~~~~~~~~~~~~~~~~~~~~~~~~~~~~~~~~~~~~~~~~~~~~~~~~~~~~~~~~~~~~~~~~~~~~~~~~  278 (582)
T PTHR12516       199 NLKASLKNGHLLDMVLNQKERTSDYSSGAPSLENIPRAPPRYWKNPFKRRALSQLLLLLLLALNTSPRTVPKITDEKTKD  278 (582)
T ss_pred              HHHHHhhhhHHHHHHHHHhhhhhhhhhhccchhhhhhhhhhhhhhhhhhHHHHHHHHHHHHHHHHHhhhccchhhhhhhhhhhh


Q ss_pred              ccccccccchhhcccccccccccchhhhhhhhh---hhccccccccccccccCCCCCCcCCCCCCccccCCCCCCccccc
Q gi|45553317|re  152 IATRPVSLHQMANEAAESNWCYQDNWKHLNNFYQ---QASVNAPKMPVPINIYSPDAPEEPINLAPPGHQPSCRTQSQKT  228 (468)
Q Consensus       152 ~~~~~~~~~~~~~~~~~~~~~~~~~~~~~~~~~~---~~~~~~~~~~~~~~~~~~~~~~~~~~~~~~~~~~~~~~~~~~p~~~~~~~~~~~~~  228 (468)
                       .+.....+.++++.+++++|||+|||+|++++||   +...++.+..+..++|+|+.|++|..+..|.+.|..+.+.+..
T Consensus       279 ~~~~~~~~~~~~~~~~~~~~~~~~~~~~~~~~~~~~~~~~~~~~~~~~~~~~~~~~~~~~~~~~~~~~~~~~~~~~~~~~~~~  358 (582)
T PTHR12516       279 IATRPVSLHQMANEAAESNWCYQDNWKHLNNFYQAAAQASVNAPKMPVPINIYSPDAPEEPINLAPPGHQPSCRTQSQKT  358 (582)
T ss_pred              hhhhhhHHHHHHHHHHHhhhHHHHHHHHHHHHHHHHHHHHHHhhhhhcccccchhhhcccccchhhhhhhhcccccccchhhhhhhhhh


Q ss_pred              ccccchhhcccccccccccccccCCcCCCCCCccEEEEEcchhccCCCCCCCccccccccccchHHHHHHHHHHHHhhcCCC
Q gi|45553317|re  229 EPTENRHLGIFVHPFNGMNIMKRRHEMTPTPTILTSGTYNDSLLTINSDYDAYLLDFPLMGDDFMLYLARMELKCRFRRH  308 (468)
Q Consensus       229 ~~~~~~~~~~~~~~~~~~~~~~g~~~~~~~~~~e~~~~~P~~~~Iv~iGDSIT~G~~~~~~~~~~~~~~~~~~liGds~~~~La~~~l~~~~~~~~~  308 (468)
                       .+..+..++++.+++.|....+++++.++.|+.+....+.||+.....++.....    .|+++..+++...+.+.+...
T Consensus       359 ~~~~~~~~~~~~~~~~~~~~~~~~~~~~~~~~~~~~~~~~~~~~~~~~~~~~~~~~~~~~~~~~----~~~~~~~~~~~~~~~~~~~~~~  434 (582)
T PTHR12516       359 EPTENRHLGIFVHPFNGMNIMKRRHEMTPTPTILTSGTYNDSLLTINSDYDDFPL----MGDDFMLYLARMELKCRFRRH  434 (582)
T ss_pred              cchhhhhhhhhhhhhhhhhHHHHHHHHhhhccccchhhhccccchhhhhhhhhhhhhhhhh----hhHHHHHHHHHHHHHHHHHHH
```

```
Q ss_pred              ceEEEeC--CCcCCccHHHHHHHHHHHHHhcCCCeEEEEeeeccCCCCCCHHHHHHHHHHHHHHHHHhC---CCEEEEEcCC
Q gi|45553317|re   309 ERVLQSG--LCVSGLTINGARNRLKRVQLPEGTQIIVNIGSVDIMRGKPLVQIEHDFRLLIKEMHNM---RLVPILTNLA  383 (468)
Q Consensus        309 ~~V~N~G--~~isG~ts~~~~~rl~~~l~~~PDlVvI~lGtND~~~g~s~e~f~~~l~~LI~~lr~~---ga~VIL~t~~  383 (468)
                       ..+....  +|++|.+......++..+.......+++.+|.-|+..+.....+...+.-++..+...   ....++..+.
T Consensus        435 ~~~~~~~~~~~~~~~~~~~~~~~~~~~~~~~~~~~~~~~~~~~~~~~~~~~~~~~~~~~~~~~~~~~~~~~~~~~~~~~~  514 (582)
T PTHR12516        435 ERVLQSAAGLCVSGLTINGARNRLKRVQLPEGTQIIVNIGSVDIMRGKPLVQIEHDFRLLIKEMHNMRLVPLLAILTNLA  514 (582)
T ss_pred              HHHHHhhhhhhhhhhhHHhhHHHHHHHhhhhccchhhhhhhhhhHhhhhhhHHHHHHHHHHHHHHHHHHHHHHHHHHHHHH


Q ss_pred              CCCccccchhhHHHHHHHHHHHHHHHHhCCCEEEECHHHHhhhcccccHhhcCCCCcCCCCcHHHHHHH
Q gi|45553317|re   384 PLGNYCHDKVLCDKIYRFNKFIRSECCHLKVIDIHSCLINERGVVRFDCFQASPRQVTGSKEPYLFW  450 (468)
Q Consensus        384 P~~~~~~~~~~~~~~i~~~N~~ir~~A~gv~fIDl~~~~~~~~g~~~~~~~~dDGvHPn~~~~Gy~~l  450 (468)
                       |...+......+..+..++..++..+-++.++|++.++..+.+....+++.......+.++..|..|
T Consensus        515 ~~~~~~~~~~~~~~~~~~~~~~~~~~~~~~~~~~~~~~~~~~~~~~~~~~~~~~~~~~~~~~~~~~~  581 (582)
T PTHR12516        515 PLGNYCHDKVLCDKIYRFNKFIRSECCHLKVIDIHSCLINERGVVRFDCFQASPRQVTGSKEPYLFW  581 (582)
T ss_pred              hhhhhhHHHHHHHHHHHHHHHHHHHHHHHHHHHHHHHHHHHHHHHhhhhhhhHHHhhhhhhhhhcchhhhhcc
```

No 2 Conserved Domains PubMed

>**cd09972** LOTUS_TDRD_OSKAR The first LOTUS domain in Oskar and Tudor-containing proteins 5 and 7. The first LOTUS domain in Oskar and Tudor-containing proteins 5 and 7: The LOTUS containing proteins are germline-specific and are found in the nuage/polar granules of germ cells. Tudor-containing protein 5 and 7 belong to the evolutionary conserved Tudor domain-containing protein (TDRD) family involved in germ cell development. In mice, TDRD5 and TDRD7 are components of the intermitochondrial cements (IMCs) and the chromatoid bodies (CBs), which are cytoplasmic ribonucleoprotein granules involved in RNA processing for spermatogenesis. Oskar protein is a critical component of the pole plasm in the Drosophila oocyte, which is required for germ cell formation.The exact molecular function of LOTUS domain remains to be identified. Its occurrence in proteins associated with RNA metabolism suggests that it might be involved in RNA binding function. The presence of several basic residues and RNA fo

Probab=99.94 E-value=3e-26 Score=191.34 Aligned_cols=83 Identities=31% Similarity=0.621 Sum_probs=0.0

```
Q ss_pred              HHHHHHHHHHHeecCCCCCcCHHHHHHHHHHHhCCcCCC----cccHHHHHhcCCCeEEEEeCCCCEEEEEecCccchHHH
Q gi|45553317|re    15 YPDIDSEVRAILLSHAQNGITISSIKSEYRKLTGNPFPL----HDNVTDFLLTIPNVTAECSESGKRIFNLKASLKNGHL   90 (468)
Q Consensus         15 ~~~~~~~irs~l~s~~k~g~~~~~l~~dy~~~~g~~~p~----~~~~~~~~l~~~~~~~~~~~~~~g~~~~~~~~~~~t~hi   90 (468)
                       +++++++|||||+|  +|+|+|+++|++|||+++|++|||    |+|++|||++||||+..++.+|+++++++++++++|+||
T Consensus          1 ~~~vk~~lRslL~S-~k~gvt~~~l~~dY~~~~G~~iP~r~lG~~sl~~fL~s~pdvv~~~~~~g~~~v~~~~~~~~t~HI   79 (87)
T cd09972          1 LEEVKKVLRSLLIS-SKGGLTLSELERDYRELEGEPIPYRKLGYSSLEDFLRSIPDVVTVRSSGGEVLVKAVADEKTAHI   79 (87)
T ss_pred              ChHHHHHHHHHHhhh-CCCCcCHHHHHHHHHHHcCCcCCchhcCcccHHHHHhcCCCEEEEEcCCCcEEEEEecChhhHHH


Q ss_pred              HHHHHHhc
Q gi|45553317|re    91 LDMVLNQK   98 (468)
Q Consensus         91 ~~mv~~Qk   98 (468)
                       ++||++||
T Consensus         80 ~~lV~~QK   87 (87)
T cd09972         80 ASLVAKQK   87 (87)
T ss_pred              HHHHHhcC
```

No 3 Conserved Domains PubMed

>**cd01836** FeeA_FeeB_like SGNH_hydrolase subfamily, FeeA, FeeB and similar esterases/lipases. FeeA and FeeB are part of a biosynthetic gene cluster and may participate in the biosynthesis of long-chain N-acyltyrosines by providing saturated and unsaturated fatty acids, which it turn are loaded onto the acyl carrier protein FeeL. SGNH hydrolases are a diverse family of lipases and esterases. The tertiary fold of the enzyme is substantially different from that of the alpha/beta hydrolase family and unique among all known hydrolases; its active site closely resembles the Ser-His-Asp(Glu) triad found in other serine hydrolases.

Probab=99.94 E-value=6e-25 Score=209.04 Aligned_cols=179 Identities=15% Similarity=0.113 Sum_probs=0.0

```
Q ss_pred              ccEEEEEcchhccCCCCCCCcccccccccchHHHHHHHHHHHHhhcCCCceEEEeCCCcCCccHHHHHHHHHHHHhcCCCe
Q gi|45553317|re   261 ILTSGTYNDSLLTINSDYDAYLLDFPLMGDDFMLYLARMELKCRFRRHERVLQSGLCVSGLTINGARNRLKRVQLPEGTQ  340 (468)
Q Consensus        261 ~~~Iv~iGDSIT~G~~~~~~~~~~~~~~liGds~~~~La~~~l~~~~~~~~~~~V~N~G~~isG~ts~~~~~rl~~~l~~~PDl  340 (468)
                       .++|+|+|+|||||.|.....         .+.+|...+++ .+...+...+.+.|.|  ++|.|+.+++.++......+||+
T Consensus          2 ~~~i~~~GDSit~G~g~~~--------~~~~~~~~~~~~~-~l~~~~~~~~~~v~n~g--~~G~tt~~~~~~l~~~~~~~~pd~   70 (191)
T cd01836          2 PLRLLVLGDSTAAGVGVET--------QDQALAGQLAR-GLAAITGRGVRWRLFA--KTGATSADLLRQLAPLPETRFDV   70 (191)
T ss_pred              CeEEEEEecchhceeccCc--------hhhhHHHHHHH-HHHHhcCCCceEEEEEe--CCCCCHHHHHHHHhhcccCCCCE


Q ss_pred              EEEEeeeccCCCCCCHHHHHHHHHHHHHHHHHh--CCCEEEEEcCCCCCc-----ccchhhHHHHHHHHHHHHHHHHhC---
Q gi|45553317|re   341 IIVNIGSVDIMRGKPLVQIEHDFRLLIKEMHN--MRLVPILTNLAPLGN-----YCHDKVLCDKIYRFNKFIRSECC---  410 (468)
Q Consensus        341 VvI~lGtND~~~g~s~e~f~~~l~~LI~~lr~--~ga~VIL~t~~P~~~-----~~~~~~~~~~~i~~~N~~ir~~A~---  410 (468)
                       |+|++|+||+..+.+.++|.+++++++++.+++  .+++|++++++|+..      .........++.++|++++++|+
T Consensus         71 v~i~~G~ND~~~~~~~~~~~~~~~l~~li~~i~~~~~~~~~~v~l~~~~~~~~~~~~~~~~~~~~~~~~~~~~~~~~~~n~~~~~~a~~~~  150 (191)
T cd01836         71 AVISIGVNDVTHLTSIARWRKQLAELVDALRAKFPGARVVVTAVPPLGRFPALPQPLRWLLGRRARLLNRALERLASEAP  150 (191)
T ss_pred              EEEEccCccccCcCHHHHHHHHHHHHHHHHHHCCCCEEEEecCCCccccCCCcHHHHHHHHHHHHHHHHHHHHHHHhhcC


Q ss_pred              CCEEEECHHHHhhhcccccHhhcCCCCcCCCCcHHHHHHHHHHHHHHHHH
Q gi|45553317|re   411 HLKVIDIHSCLINERGVVRFDCFQASPRQVTGSKEPYLFWNKIGRQRVL  459 (468)
Q Consensus        411 gv~fIDl~~~~~~~~g~~~~~~~~dDGvHPn~~~~Gy~~lA~~i~~~I~  459 (468)
                       ++.|+|++..+.        .+++..||+|||.  +||+.||+.+.+.|.
T Consensus        151 ~~~~vd~~~~~~-------~~~~~~~DglHpn~--~Gy~~~a~~l~~~i~  190 (191)
T cd01836        151 RVTLLPATGPLF-------PALFASDGFHPSA--AGYAVWAEALAPAIA  190 (191)
T ss_pred              CeEEEecCCccc-------cccccCCCCCCCH--HHHHHHHHHHHHHHh
```

No 4 Conserved Domains PubMed

>**cd09986** LOTUS_1_TDRD7 The first LOTUS domain on Tudor-containing protein 7 (TDRD7). The first LOTUS domain on Tudor-containing protein 7 (TDRD7): TDRD7 contains three N-terminal LOTUS domains and three Tudor domain repeats at the C-terminus. It belongs to the evolutionary conserved Tudor domain-containing protein (TDRD) family involved in germ cell development. In mice, TDRD7 together with TDRD1/MTR-1, TDRD5 and TDRD6 forms a ribonucleoprotein complex in the intermitochondrial cements (IMCs) and the chromatoid bodies (CBs) involving in RNA processing for spermatogenesis. TDRD7 is functionally essential for the differentiation of germ cells. The exact molecular function of LOTUS domain on TDRD7 remains to be characterized. Its occurrence in proteins associated with RNA metabolism suggests that it might be involved in RNA binding function. The presence of several basic residues and RNA fold recognition motifs support this hypothesis. The RNA binding function might be the first step of

Probab=99.94 E-value=4.9e-26 Score=188.20 Aligned_cols=82 Identities=24% Similarity=0.464 Sum_probs=0.0

```
Q ss_pred              HHHHHHHHHHeecCCCCCcCHHHHHHHHHHHhCCcCCC----cccHHHHHhcCCCeEEEE-eCCCCEEEEEecCccchHHH
Q gi|45553317|re    16 PDIDSEVRAILLSHAQNGITISSIKSEYRKLTGNPFPL----HDNVTDFLLTIPNVTAEC-SESGKRIFNLKASLKNGHL   90 (468)
Q Consensus         16 ~~~~~~irs~l~s~~k~g~~~~~l~~dy~~~~g~~~p~----~~~~~~~~l~~~~~~~~~~-~~~g~~~~~~~~~~~~t~hi   90 (468)
                       |+++++|||||+|  +|+|+|+++|++||++|+|++|||     |+|+++||++||||+..+ +.+|+++++++++++++|+||
T Consensus          2 ~~Vkk~lrsll~S-~k~gv~~~~L~~dY~~l~Ge~iP~r~lGy~~l~~fl~siPdvV~i~~~~~Ge~~v~av~~~~tahI   80 (88)
T cd09986           2 ELVKKMLRAVLQS-SKGGVSLPRLQGEYKELTGEQIPFKQLGYHTLDALLRSMPSVVRLERSRSGEVMCFASLCNETAHI   80 (88)
T ss_pred              hHHHHHHHHHhee-CCCCcCHHHHHHHHHHHcCCcCChhHhCchhHHHHHhcccCeEEEEecCCCCEEEEEecCHHHHHH


Q ss_pred              HHHHHHhc
Q gi|45553317|re    91 LDMVLNQK   98 (468)
Q Consensus         91 ~~mv~~Qk   98 (468)
                       ++||++||
T Consensus         81 ~~LVa~Qk   88 (88)
T cd09986          81 AKLVARQR   88 (88)
T ss_pred              HHHHHccC
```

No 5

>**4jhl_A** Acetyl xylan esterase; SGNH hydrolase fold, hydrolase; HET: MSE; 1.70A {Geobacillus stearothermophilus}
SCOP: **c.23.10.0** PDB: **3w7v _A*** **4jko _A** **4jj4 _A** **4jj6 _A**
Probab=99.94 E-value=8.9e-25 Score=208.45 Aligned_cols=198 Identities=13% Similarity=0.095 Sum_probs=0.0

```
Q ss_pred              CCCCCccEEEEEcchhccCCCCCCCcccccccccchHHHHHHHHHHHHhhcCCCCeEEEeCCCcCCccHHHHHHHHHH-HH
Q gi|45553317|re   256 TPTPTILTSGTYNDSLLTINSDYDAYLLDFPLMGDDFMLYLARMELKCRFRRHERVLQSGLCVSGLTINGARNRLKR-VQ  334 (468)
Q Consensus        256 ~~~P~~~~Iv~iGDSIT~G~~~~~~~~~~~~~~liGds~~~~La~~~l~~~~~~~~~~~V~N~G~~isG~ts~~~~~rl~~-~l  334 (468)
                       .+.|...+|+++|||||.|........+.+...+.+|...++..+-...-..++.+.|.|  ++|.++.+++.++.. +.
T Consensus          1 ~~~~~~~~~~i~~~GDSit~g~~~~~~~~~~~~~~~~~~~~~~~~~~l~~~l~~~~~~~~~~~~~~n~g--~~G~~~~~~~~~~~~~~~~~~   78 (219)
T 4jhl_A           1 MKIGSGEKLLFIGDSITDCGRARPEGEGSFGALGTGYVAYVVGLLQAVYPELGIRVVNKG--ISGNTVRDLKARWEEDVI   78 (219)
T ss_dssp              CCSCTTCEEEEEESHHHHTTCCTTTCBCSSSTTCSSHHHHHHHHHHHHHCGGGCCEEEECC--CTTCCHHHHHHHHHHHTG
T ss_pred              CcCCCCCEEEEEccchhcCcccCCCCCccccccccCCCHHHHHHHHHHhhCCCCCeEEEecC--cCcccHHHHHHHhhhhhh


Q ss_pred              hcCCCeEEEEeeeccCCC----------CCCHHHHHHHHHHHHHHHHhCCCEEEEEcCCCCCccchhhHHHHHHHHHHH
Q gi|45553317|re   335 LPEGTQIIVNIGSVDIMR----------GKPLVQIEHDFRLLIKEMHNMRLVPILTNLAPLGNYCHDKVLCDKIYRFNKF  404 (468)
Q Consensus        335 ~~~PDlVvI~lGtND~~~----------g~s~e~f~~~l~~LI~~lr~~ga~VIL~t~~P~~~~~~~~~~~~~~i~~~N~~  404 (468)
                       ..+||+|+|++|+|+..           ..+.++|.++++++|+.+++.+++||++++++........ ....+..+|++
T Consensus         79 ~~~pd~vvi~~G~ND~~~~~~~~~~~~~~~~~~~~~~~~~~~l~~~i~~~~~~~~~~vil~~p~~~~~~~~~~~~~~~n~~  157 (219)
T 4jhl_A          79 AQKPDWVSIMIGINDVWRQYDLPFMKEKHVYLDEYEATLRSLVLETKPLVKGIILMTPFYIEGNEQDP-MRRTMDQYGRV  157 (219)
T ss_dssp              GGCCSEEEEECCHHHHHHHHHSTTCGGGCCCHHHHHHHHHHHHHHHHHGGGSSEEEECCCCCCCCTTSH-HHHHHHHHHHH
T ss_pred              cCCCCEEEEEeecchhhhccccccccCCCHHHHHHHHHHHHHHHHHHHCCCCEEEECCCCCCcchhhH-HHHHHHHHHHH


Q ss_pred              HHHHhC--CCEEEEChHHHhhhcccccHhhcCCCCcCCCCcHHHHHHHHHHHHHHH
Q gi|45553317|re   405 IRSECC--HLKVIDIHSCLINERGVVRFDCFQASPRQVTGSKEPYLFWNKIGRQRV  458 (468)
Q Consensus        405 ir~~A~--gv~fIDl~~~~~~~~~~g~~~~~~~~~~dDGvHPn~~~~Gy~~lA~~i~~~I  458 (468)
                       ++++|+  ++.|||++..+.+.......+++..||+|||.  +||++||+.+.+.|
T Consensus        158 ~~~~a~~~~~~~~~~~vD~~~~~~~~~~~~~~~~~~~~~~~~~~~~~Dg~Hpn~--~G~~~~a~~l~~~~l  211 (219)
T 4jhl_A         158 VKQIAEETNSLFVDTQAAFNEVLKTLYPAALAWDRVHPSV--AGHMILARAFLREI  211 (219)
T ss_dssp              HHHHHHHHTCEEECHHHHHHHHHHTTSCGGGTCTTSSSCCH--HHHHHHHHHHHHHHT
T ss_pred              HHHHHHHcCCEEEEhHHHHHHHHHhccCcccccCCCCcCCCCCH--HHHHHHHHHHHHHh
```

No 6

>**3s93_A** Tudor domain-containing protein 5; structural genomics consortium, SGC, transcription; 2.28A {Homo sapiens}
Probab=99.94 E-value=2e-26 Score=197.60 Aligned_cols=93 Identities=20% Similarity=0.415 Sum_probs=0.0

```
Q ss_pred              hhhhhHHHHHHHHHHHHeecCCCCCcCHHHHHHHHHHHhCCcCCC----cccHHHHHhcCCCeEEEE-eCCCCEEEEEecC
Q gi|45553317|re    10 SVREEYPDIDSEVRAILLSHAQNGITISSIKSEYRKLTGNPFPL----HDNVTDFLLTIPNVTAEC-SESGKRIFNLKAS   84 (468)
Q Consensus         10 ~~~~~~~~~~~~~~irs~l~s~~k~g~~~~~l~~dy~~~~g~~~p~----~~~~~~~~l~~~~~~~~~~-~~~g~~~~~~~~~   84 (468)
                       ..++.+++++++|||||+| +|+|+|+++|++|+|||+++|     |+|++|||++||||+.++ +.+|+++++++++++
T Consensus          4 ~~~~~l~~v~~~lRslL~S~~k~gitl~~l~~dY~~~~G~~iP~r~lGy~sl~~fL~sipdvv~~~~~~~~~G~~~v~a~~~   82 (102)
T 3s93_A           4 EQERIQECLRKEIRSLLIS-TKDGLSPQELEKEYLLMVGNHLPLRILGYRSTMELVLDMPDVVRVCPGAGGTVILKAIPD   82 (102)
T ss_dssp              HHHHHHHHHHHHHHHHHHHS-SSSCBCHHHHHHHHHHHHSSCCCTGGGTCSSHHHHHHTCTTTEEEECCGGGCCEEEEC--
T ss_pred              cHHHHHHHHHHHHHhhheeee-cCCCCcHHHHHHHHHHHhcCCcCCchhcCcccHHHHHHcCCCEEEEEecCCCCEEEEEEeC


Q ss_pred              ccHHHHHHHHHHhccCCcc
Q gi|45553317|re    85 LKNGHLLDMVLNQKERTSD  103 (468)
Q Consensus         85 ~~t~hi~~mv~~Qk~~~~~  103 (468)
                       ++|+||++||||++||++++.
T Consensus         83 ~~t~HI~~lV~~Qkss~k~  101 (102)
T 3s93_A          83 ESTKGIASLVAKQRSSHKL  101 (102)
T ss_dssp              -------------------
T ss_pred              HHHHHHHHHHHhhcCCccC
```

No 7

>**cd09985** LOTUS_1_TDRD5 The first LOTUS domain on Tudor-containing protein 5 (TDRD5). The first LOTUS domain on Tudor-containing protein 5 (TDRD5): TDRD5 contains three N-terminal LOTUS domains and a C-terminal Tudor domain. It belongs to the evolutionary conserved Tudor domain-containing protein (TDRD) family involved in germ cell development. In mice, TDRD5 is a component of the intermitochondrial cements (IMCs) and the chromatoid bodies (CBs), which are cytoplasmic ribonucleoprotein granules involved in RNA processing for spermatogenesis. The exact molecular function of LOTUS domain on TDRD5 remains to be identified. Its occurrence in proteins associated with RNA metabolism suggests that it might be involved in RNA binding function. The presence of several basic residues and RNA fold recognition motifs support this hypothesis. The RNA binding function might be the first step of regulating mRNA translation or localization.
Probab=99.93 E-value=1.1e-25 Score=189.29 Aligned_cols=86 Identities=23% Similarity=0.499 Sum_probs=0.0

```
Q ss_pred              HHHHHHHHHHHeecCCCCCcCHHHHHHHHHHHhCCcCCC----cccHHHHHhcCCCeEEEE-eCCCCEEEEEecCccchHH
Q gi|45553317|re    15 YPDIDSEVRAILLSHAQNGITISSIKSEYRKLTGNPFPL----HDNVTDFLLTIPNVTAEC-SESGKRIFNLKASLKNGH   89 (468)
Q Consensus         15 ~~~~~~~~irs~l~s~~k~g~~~~~l~~dy~~~~g~~~p~----~~~~~~~~l~~~~~~~~~~-~~~g~~~~~~~~~~~~t~h   89 (468)
                       +++++++|||||+|  +|+|+|+++|++||++|+|++|||     |.|+++||++||||+.++ +.+|+++++++++++++|+|
T Consensus          5 l~~Vkk~vrsll~S-~k~gv~l~~L~~dY~~~~Ge~iP~r~LGy~sl~~~L~s~pdvv~~~~~~~~G~~~v~av~~~~tah   83 (95)
T cd09985           5 MERLKKDVRSLLIS-SKNGLTPEQLEQDYLAMVGSPLPLRSLGFRSTMELVLDMPDVVRVHPQLDGTVILKAVGDETTKG   83 (95)
T ss_pred              HHHHHHHHHHHhhc-CCCCcCHHHHHHHHHHHhCCCCChhhcCcccHHHHHHhccCeEEEEecCCCcEEEEEecCHHHHH
```

```
Q ss_pred                  HHHHHHHhccCC
Q gi|45553317|re   90 LLDMVLNQKERT  101 (468)
Q Consensus        90 i~~mv~~Qk~~~  101 (468)
                      |++||++||+++
T Consensus        84 I~~lV~~QK~sk   95 (95)
T cd09985          84 IEDLVSKQRDSK   95 (95)
T ss_pred             HHHHHHhccCCC
```

No 8

>3rjt_A Lipolytic protein G-D-S-L family; PSI-biology, midwest center for structural genomics, MCSG, H; 1.50A {Alicyclobacillus acidocaldarius subsp} SCOP: c.23.10.0
Probab=99.93 E-value=1.1e-24 Score=206.94 Aligned_cols=199 Identities=13% Similarity=0.034 Sum_probs=0.0

```
Q ss_pred                  CCCCCCccEEEEEcchhccCCCCCCCcccccccccchHHHHHHHHHHHHhhcCCCCceEEEeCCCCcCCccHHHHHHHHHH-H
Q gi|45553317|re  255 MTPTPTILTSGTYNDSLLTINSDYDAYLLDFPLMGDDFMLYLARMELKCRFRRHERVLQSGLCVSGLTINGARNRLKR-V  333 (468)
Q Consensus       255 ~~~~P~~~~Iv~iGDSIT~G~~~~~~~~~~~~~~liGds~~~~La~~~l~~~~~~~~~~~V~N~G~~isG~ts~~~~~rl~~-~  333 (468)
                      ..+.+...+|+++|||||.|............-.+.+|...++..+....-..++.+.|.|  ++|.++.++..++.. +
T Consensus        2 ~~~~~~~~~~~i~~~GDSit~g~~~~~~~g~~~~~~~~~~~~~~~~~~~~l~~~l~~~~~~~~~~~~~~~n~g-~~~G~~~~~~~~~~~~~~~~~~~~   79 (216)
T 3rjt_A            2 NAMIEPGSKLVMVGDSITDCGRAHPVGEAPRGGLGNGYVALVDAHLQVLHPDWRIRVVNVG--TSGNTVADVARRWEDDV   79 (216)
T ss_dssp             -CCCCTTCEEEEEESHHHHTTCCSSCEESSTTTTCSSHHHHHHHHHHHHCGGGCCEEEECC--CTTCCHHHHHHHHHHHT
T ss_pred             CCCCCCCCEEEEEecChhhccccccccCcccccCccHHHHHHHHHHhhCCCCCceEEEECC--cCchhHHHHHHHHHhhH
```

```
Q ss_pred                  HhcCCCeEEEEeeeccCCC----------CCCHHHHHHHHHHHHHHHHhCCCEEEEEcCCCCCccccchhhHHHHHHHHHHH
Q gi|45553317|re  334 QLPEGTQIIVNIGSVDIMR----------GKPLVQIEHDFRLLIKEMHNMRLVPILTNLAPLGNYCHDKVLCDKIYRFNK  403 (468)
Q Consensus       334 l~~~PDlVvI~lGtND~~~----------g~s~e~f~~~l~~LI~~lr~~ga~VIL~t~~P~~~~~~~~~~~~~~~i~~~N~  403 (468)
                      ...+||+|+|++|+||+..          ..+.++|.++++++|+.+++.+++||++++++++........    ....+..+|+
T Consensus       80 ~~~~pd~vvi~~G~ND~~~~~~~~~~~~~~~~~~~~~~~~~~l~~~i~~~~~~~~~~~~vil~~p~~~~~~~~~~~~~~~~-~~~~~~~~~~~~n~  158 (216)
T 3rjt_A           80 MALQPDYVSLMIGVNDVWRQFDMPLVVERHVGIDEYRDTLRHLVATTKPRVREMFLLSPFYLEPNRSDP-MRKTVDAYIE  158 (216)
T ss_dssp             GGGCCSEEEEECCHHHHHHHHHSTTCGGGCCCHHHHHHHHHHHHHHHHGGGSSEEEECCCCCCCCTTSH-HHHHHHHHHH
T ss_pred             hhcCCCEEEEEcccchhhhhccCCcccccCCCHHHHHHHHHHHHHHHHHHcCCCcEEEECCcccCCccchhH-hHHHHHHHHH
```

```
Q ss_pred                  HHHHHhC--CCEEEEChHHHhhhcccccHhhcCCCCcCCCCcHHHHHHHHHHHHHHH
Q gi|45553317|re  404 FIRSECC--HLKVIDIHSCLINERGVVRFDCFQASPRQVTGSKEPYLFWNKIGRQRV  458 (468)
Q Consensus       404 ~ir~~A~--gv~fIDl~~~~~~~~g~~~~~~~~dDGvHPn~~~~Gy~~lA~~i~~~I  458 (468)
                      +++++|+  ++.|||++..|.+.......+++..||+|||.  +||++||+.+.+.|
T Consensus      159 ~~~~~a~~~~~~~~vD~~~~~~~~~~~~~~~~~~~~~DgiHpn~--~G~~~~a~~i~~~l  213 (216)
T 3rjt_A          159 AMRDVAASEHVPFVDVQAEFDRLLAHLNTWVLAPDRVHPYL--NGHLVIARAFLTAV  213 (216)
T ss_dssp             HHHHHHHHHTCCEECHHHHHHHHHTTSCHHHHCSSSSSCCH--HHHHHHHHHHHHHHT
T ss_pred             HHHHHHHHcCCEEEehHHHHHHHHHhcCCCcccccCCCcCCCh--HHHHHHHHHHHHHh
```

No 9

>1yzf_A Lipase/acylhydrolase; structural GENO PSI, protein structure initiative, midwest center for struc genomics, MCSG; 1.90A {Enterococcus faecalis} SCOP: c.23.10.5
Probab=99.93 E-value=6.8e-25 Score=205.49 Aligned_cols=190 Identities=15% Similarity=0.164 Sum_probs=0.0

```
Q ss_pred                  ccEEEEEcchhccCCCCCCCcccccccccchHHHHHHHHHHHHhhcCCCCceEEEeCCCCcCCccHHHHHHHHH-HHHHhcCCC
Q gi|45553317|re  261 ILTSGTYNDSLLTINSDYDAYLLDFPLMGDDFMLYLARMELKCRFRRHERVLQSGLCVSGLTINGARNRLK-RVQLPEGT  339 (468)
Q Consensus       261 ~~~Iv~iGDSIT~G~~~~~~~~~~~~~~liGds~~~~La~~~l~~~~~~~~~~~V~N~G~~isG~ts~~~~~rl~-~~~l~~~PD  339 (468)
                      ..+|+++|||||.|.....          .+++|...|+.  .+.......++.+.|.|  ++|.++.++..+++ .++..+||
T Consensus        1 m~~i~~~~GDSit~g~~~~~~~~~-~~~~~~~~~~~~~~~~~~~~~~~~l~~-~~~~~~~~~~~~~~~v~n~g--~~G~~~~~~~~~~~~~~~~~~~pd   69 (195)
T 1yzf_A            1 MRKIVLFGDSITAGYLDEA--------VSPVLVDLVKR-DIAAMGLEEVAVINAG--MPGDTTEDGLKRLNKEVLIEKPD   69 (195)
T ss_dssp             CEEEEEEESHHHHCBTTBS--------SCSHHHHHHHH-HHHHTTBCCEEEEEEE--CTTCCHHHHHHHHHHHTGGGCCS
T ss_pred             CceEEEecccchhhcccCCC--------ccchHHHHHHH-HHhhccCCCeEEEeCC--CCCCcHHHHHHHHHHHHhhhCCC
```

```
Q ss_pred                  eEEEEeeeccCC--CCCCHHHHHHHHHHHHHHHHHhCCCEEEEEcCCCCCccccchhhHHHHHHHHHHHHHHHhC--CCEEE
Q gi|45553317|re  340 QIIVNIGSVDIM--RGKPLVQIEHDFRLLIKEMHNMRLVPILTNLAPLGNYCHDKVLCDKIYRFNKFIRSECC--HLKVI  415 (468)
Q Consensus       340 lVvI~lGtND~~--~g~s~e~f~~~l~~LI~~lr~~ga~VIL~t~~P~~~~~~~~~~~~~i~~~N~~ir~~A~--gv~fI  415 (468)
                      +|+|++|+|+.  .+.+.++|.++++++|+.++.  +|||++++|+...........+..+|++++++|+  ++.||
T Consensus       70 ~vvi~~G~ND~~~~~~~~~~~~~~~~~l~~~i~~~~~~~~~~~~~vv~~p~~~~~~~~~~~~~~~~~~~~~~~~n~~~~~~~~a~~~~~~~~~~~~i  147 (195)
T 1yzf_A           70 EVVIFFGANDASLDRNITVATFRENLETMIHEIGSE--KVILITPPYADSGRRPERPQTRIKELVKVAQEVGAAHNLPVI  147 (195)
T ss_dssp             EEEEECCTTTTCTTSCCCHHHHHHHHHHHHHHHCGG--GEEEECCCCCCTTCTTSCHHHHHHHHHHHHHHHHHHHTTCCEE
T ss_pred             EEEEecccccccccCCCHHHHHHHHHHHHHHHhCCC--CEEEEcCCCcccccccccchHHHHHHHHHHHHHHHHHcCCcEE
```

```
Q ss_pred                  EChHHHhhhcccccHhhcCCCCcCCCCcHHHHHHHHHHHHHHHHHHHHHHHHHhc
Q gi|45553317|re  416 DIHSCLINERGVVRFDCFQASPRQVTGSKEPYLFWNKIGRQRVLQVIETSLE  467 (468)
Q Consensus       416 Dl~~~~~~~~g~~~~~~~~dDGvHPn~~~~Gy~~lA~~i~~~I~~~L~~~~a  467 (468)
                      |++..+.+..+  ..+++..||+|||.  +||++||+.+.+.|.+.+++..+
T Consensus      148 D~~~~~~~~~~~~~~~~~~~~~~~~~~~~~Dg~Hpn~~~~G~~~~a~~l~~~l~~~l~~~~~~  195 (195)
T 1yzf_A          148 DLYKAMTVYPG--TDEFLQADGLHFSQ--VGYELLGALIVREIKGRLKPKQA  195 (195)
T ss_dssp             CHHHHHHHSTT--GGGGBCTTSSSBCH--HHHHHHHHHHHHHHHHGGGCCBCCC
T ss_pred             eHHHHHhhccC--cccccccCCCCcCH--HHHHHHHHHHHHHHHHHHHhhccCC
```

No 10

>cd01841 NnaC_like NnaC (CMP-NeuNAc synthetase) _like subfamily of SGNH_hydrolases, a diverse family of lipases and esterases. The tertiary fold of the enzyme is substantially different from that of the alpha/beta hydrolase family and unique among all known hydrolases; its active site closely resembles two of the three components of typical Ser-His-Asp(Glu) triad from other serine hydrolases. E. coli NnaC appears to be involved in polysaccharide synthesis.
Probab=99.93 E-value=1.6e-24 Score=202.63 Aligned_cols=167 Identities=16% Similarity=0.174 Sum_probs=0.0

```
Q ss_pred                  EEEEEcchhccCCCCCCCcccccccccchHHHHHHHHHHHHhhcCCCCceEEEeCCCCcCCccHHHHHHHH-HHHHHhcCCCeE
Q gi|45553317|re  263 TSGTYNDSLLTINSDYDAYLLDFPLMGDDFMLYLARMELKCRFRRHERVLQSGLCVSGLTINGARNRL-KRVQLPEGTQI  341 (468)
Q Consensus       263 ~Iv~iGDSIT~G~~~~~~~~~~~~~~liGds~~~~La~~~l~~~~~~~~~~~V~N~G~~isG~ts~~~~~rl-~~~l~~~PDlV  341 (468)
                      +|+|+|||+|+|.......                          .....|.|.|  ++|.|+.++..++ ..+...+|||+|
T Consensus        2 ~iv~~GDS~t~g~~~~~~~~~~~~~~~~~~~~~~~~~~~~~~~~~~~~~v~N~G--~~~G~t~~~~~~~~~~~~~~~~~~~~~~~pd~v   55 (174)
T cd01841          2 NIVFIGDSLFEGWPLYEAE-------------------------GKGKTVNNLG--IAGISSRQYLEHIEPQLIQKNPSKV   55 (174)
T ss_pred             CEEEEechhhcCCChhhhc-------------------------CCCCceEEec--cccCCHHHHHHHHHhHhhcCCCEE
```

```
Q ss_pred                EEEeeeccCCCCCCHHHHHHHHHHHHHHHHHhC--CCEEEEEcCCCCCccc-chhhHHHHHHHHHHHHHHHhC--CCEEEE
Q gi|45553317|re    342 IVNIGSVDIMRGKPLVQIEHDFRLLIKEMHNM--RLVPILTNLAPLGNYC-HDKVLCDKIYRFNKFIRSECC--HLKVID  416 (468)
Q Consensus         342 vI~lGtND~~~g~s~e~f~~~l~~LI~~lr~~--ga~VIL~t~~P~~~~~~-~~~~~~~~~~i~~~N~~ir~~A~--gv~fID  416 (468)
                         +|++|+||+..+.+.++|.+++++||+.+++.  +++|++++++|+....  ......+.+.+||++++++|+  ++.|||
T Consensus          56 ii~~G~ND~~~~~~~~~~~~~~~~~li~~i~~~~p~~~i~~~~~~~p~~~~~~~~~~~~~~~~~~~~~n~~l~~~a~~~~~~~~iD  135 (174)
T cd01841           56 FLFLGTNDIGKEVSSNQFIKWYRDIIEQIREEFPNTKIYLLSVLPVLEEDEIKTRSNTRIQRLNDAIKELAPELGVTFID  135 (174)
T ss_pred                EEEeeeccCCCCCCHHHHHHHHHHHHHHHHHHHCCCCeEEEEeCCCcCcccccccCHHHHHHHHHHHHHHHHcCCEEEE
```

```
Q ss_pred                ChHHHhhhcccccHhhcCCCCcCCCCcHHHHHHHHHHHHHHH
Q gi|45553317|re    417 IHSCLINERGVVRFDCFQASPRQVTGSKEPYLFWNKIGRQRV  458 (468)
Q Consensus         417 l~~~~~~~~g~~~~~~~~~dDGvHPn~~~~Gy~~lA~~i~~~I  458 (468)
                         ++..|.+..+.....+..|+ +|||+  +||++||+.|.+.|
T Consensus         136 ~~~~~~~~~~~~~~~~~~~DG-lH~n~--~Gy~~~a~~l~~~~  174 (174)
T cd01841          136 LNDVLVDEFGNLKKEYTTDG-LHFNP--KGYQKLLEILEEYL  174 (174)
T ss_pred                CcHHhcCCCCCCcchhhcCCC-cCcCH--HHHHHHHHHHHhhC
```

No 11

>**4rsh_A** Lipolytic protein G-D-S-L family; structural genomics, PSI-biology, midwest center for structu genomics, MCSG; 2.19A {Desulfitobacterium hafniense dcb-2}
Probab=99.93 E-value=1.1e-24 Score=203.33 Aligned_cols=174 Identities=15% Similarity=0.223 Sum_probs=0.0

```
Q ss_pred                CccEEEEEcchhccCCCCCCCcccccccccchHHHHHHHHHHHhhcCCCceEEEeCCCcCCccHHHHHHHHH-HHHhcCC
Q gi|45553317|re    260 TILTSGTYNDSLLTINSDYDAYLLDFPLMGDDFMLYLARMELKCRFRRHERVLQSGLCVSGLTINGARNRLK-RVQLPEG  338 (468)
Q Consensus         260 ~~~~Iv~iGDSIT~G~~~~~~~~~~~~~~liGds~~~~La~~~l~~~~~~~~~~V~N~G~~isG~ts~~~~~rl~~~~l~~~P  338 (468)
                         ..++|+|+|||||.|....          .+.+|+..++..+        ++.+.|.|  ++|.++.++..+++ .+...+|
T Consensus           3 ~~~~i~~~GDSit~g~~~~-----------------~~l~~~l----------n~g~~~G~~~~~~~~~~~~~~~~~~~p   64 (179)
T 4rsh_A             3 ANTKVVAIGDSFTFGYPGN---------TENSWPAVLGQTS-------QIEVVNKG--LKSQTAQDLYSRFDADVLAEKP   64 (179)
T ss_dssp               -CCCEEEEESHHHHTTTSC---------GGGSHHHHHHHHT-------TSCEEEEE--CTTCCHHHHHHHHHHHTGGGCC
T ss_pred               CCcEEEEEeccccccccCCC---------CCCcHHHHHHHhc-------CceEEecC--cCcccHHHHHHHHHHHHHhcCC
```

```
Q ss_pred                CeEEEEeeeccCCCCCCHHHHHHHHHHHHHHHHHhCCCEEEEEcCCCCCccccchhhHHHHHHHHHHHHHHHhC--CCEEEE
Q gi|45553317|re    339 TQIIVNIGSVDIMRGKPLVQIEHDFRLLIKEMHNMRLVPILTNLAPLGNYCHDKVLCDKIYRFNKFIRSECC--HLKVID  416 (468)
Q Consensus         339 DlVvI~lGtND~~~g~s~e~f~~~l~~LI~~lr~~ga~VIL~t~~P~~~~~~~~~~~~~~~~~i~~~N~~ir~~A~--gv~fID  416 (468)
                         |+|+|++|+||+..+.+.++|.++++++++|+.+++.+.|+|++++|..      .....+..+|++++++|+  ++.|||
T Consensus          65 d~vvi~~G~ND~~~~~~~~~~~~~~l~~i~~~~~~~~~~vil~~~~p~~--------------n~~~~~~a~~~~~~~vD  138 (179)
T 4rsh_A            65 GRVIIFVGNGDAIKEVPLETFQQHIKAMVEKAESNHIIPILALPLPYT------GVQNTIKEFREWESSYAKEKNILVLD  138 (179)
T ss_dssp               SEEEEECSHHHHTTCCHHHHHHHHHHHHHHHHHHTTCEEEEECCCCCT------TCHHHHHHHHHHHHHHHHTTCEEEC
T ss_pred               CEEEEEeecccccccCCHHHHHHHHHHHHHHHHHHHcCCeEEEEeCCCCc------hHHHHHHHHHHHHHHHHHHcCCeEEe
```

```
Q ss_pred                ChHHHhhhcccccHhhcCCCCcCCCCcHHHHHHHHHHHHHHHH
Q gi|45553317|re    417 IHSCLINERGVVRFDCFQASPRQVTGSKEPYLFWNKIGRQRVL  459 (468)
Q Consensus         417 l~~~~~~~~g~~~~~~~~~dDGvHPn~~~~Gy~~lA~~i~~~I~  459 (468)
                         ++..+.+..+.....++..||+|||.  +||++||+.+.+.|+
T Consensus         139 ~~~~~~~~~~~~~~~d~~~~~Dg~Hpn~--~G~~~~~a~~l~~~l~  179 (179)
T 4rsh_A           139 FATVLMDADNVYLEGLLSKEANYPSK--EGYKLMGEYASRVLD  179 (179)
T ss_dssp               THHHHBCTTSCBCTTTBCSSTTSBCH--HHHHHHHHHHHHHC-
T ss_pred               hHHHhccccccccCCccCCCCCCCCCH--HHHHHHHHHHHHHhC
```

No 12

>**cd04501** SGNH_hydrolase_like_4 Members of the SGNH-hydrolase superfamily, a diverse family of lipases and esterases. The tertiary fold of the enzyme is substantially different from that of the alpha/beta hydrolase family and unique among all known hydrolases; its active site closely resembles the Ser-His-Asp(Glu) triad from other serine hydrolases, but may lack the carboxlic acid.
Probab=99.93 E-value=3.9e-24 Score=200.53 Aligned_cols=177 Identities=18% Similarity=0.198 Sum_probs=0.0

```
Q ss_pred                cEEEEEcchhccCCCCCCCcccccccccchHHHHHHHHHHHhhcCCCceEEEeCCCcCCccHHHHHHHHHH-HHhcCCCCe
Q gi|45553317|re    262 LTSGTYNDSLLTINSDYDAYLLDFPLMGDDFMLYLARMELKCRFRRHERVLQSGLCVSGLTINGARNRLKR-VQLPEGTQ  340 (468)
Q Consensus         262 ~~Iv~iGDSIT~G~~~~~~~~~~~~~~liGds~~~~La~~~l~~~~~~~~~~V~N~G~~isG~ts~~~~~rl~~~~l~~~PDl  340 (468)
                         +||+|+|||+|.|....          .+.+|+..+.         ..+.++|.|  ++|.++.++..++.. +...+||+
T Consensus           1 ~ri~~~GDS~t~g~~~~---------------w~~~l~~~------~~~n~g~~~G~~~~~~~~~~~~~~~~~pd~  62 (183)
T cd04501           1 MRVVCLGDSITYGYPVG---------PEASWVNLLAE-------FLGKEVINRG--INGDTTSQMLVRFYEDVIALKPAV  62 (183)
T ss_pred               CeEEEEecccccCCC---------CCCcHHHHHH-------hcCCcEEECC--cCcccHHHHHHHHHHHHHhcCCCE
```

```
Q ss_pred                EEEEeeeccCCCCCCHHHHHHHHHHHHHHHHHhCCCEEEEEcCCCCCccch---hhHHHHHHHHHHHHHHHhC--CCEEE
Q gi|45553317|re    341 IIVNIGSVDIMRGKPLVQIEHDFRLLIKEMHNMRLVPILTNLAPLGNYCHD---KVLCDKIYRFNKFIRSECC--HLKVI  415 (468)
Q Consensus         341 VvI~lGtND~~~g~s~e~f~~~l~~LI~~lr~~ga~VIL~t~~P~~~~~~~~~~~~~~~~~~~i~~~N~~ir~~A~--gv~fI  415 (468)
                         |+|++|+||+...+.+.++|.+++.++++|+.++++|++++++|...      ...+..+.+|++++++|+  ++.||
T Consensus          63 vv~~~G~ND~~~~~~~~~~~~~~~~~li~~~~~~~vv~~~~p~~~~~~~~~~~~~~~~~~~~~~~~~n~~l~~~a~~~~~~i  142 (183)
T cd04501          63 VIIMGGTNDIIVNTSLEMIKDNIRSMVELAEANGIKVILASPLPVDDYPWKPQWLRPANKLKSLNRWLKDYARENGLLFL  142 (183)
T ss_pred               EEEEeeecccccCCHHHHHHHHHHHHHHHHHHhCCCeEEEEeCCCcCcccchhhHHHHHHHHHHHHHHHHHHHHHHHcCCcEE
```

```
Q ss_pred                EChHHHhhhcccccHhhcCCCCcCCCCcHHHHHHHHHHHHHHH
Q gi|45553317|re    416 DIHSCLINERGVVRFDCFQASPRQVTGSKEPYLFWNKIGRQRV  458 (468)
Q Consensus         416 Dl~~~~~~~~g~~~~~~~~~dDGvHPn~~~~Gy~~lA~~i~~~I  458 (468)
                         |++..+.+..+......+..||+|||+  +||++||+.+.+.|
T Consensus         143 d~~~~~~~~~~~~~~~~~~~~~DgiHpn~--~G~~~~a~~i~~~i  183 (183)
T cd04501          143 DFYSPLLDERNVGLKPGLLTDGLHPSR--EGYRVMAPLAEKAL  183 (183)
T ss_pred               EchHHHhcccccccchhhcCCCCcCH--HHHHHHHHHHHHhC
```

No 13

>**cd01821** Rhamnogalacturan_acetylesterase_like Rhamnogalacturan_acetylesterase_like subgroup of SGNH-hydrolases. Rhamnogalacturan acetylesterase removes acetyl esters from rhamnogalacturonan substrates, and renders them susceptible to degradation by rhamnogalacturonases. Rhamnogalacturonans are highly branched regions in pectic polysaccharides, consisting of repeating -(1,2)-L-Rha-(1,4)-D-GalUA disaccharide units, with many rhamnose residues substituted by neutral oligosaccharides such as arabinans, galactans and arabinogalactans. Extracellular enzymes participating in the degradation of plant cell wall polymers, such as Rhamnogalacturonan acetylesterase, would typically be found in saprophytic and plant pathogenic fungi and bacteria.
Probab=99.93 E-value=3.3e-24 Score=205.72 Aligned_cols=179 Identities=12% Similarity=0.095 Sum_probs=0.0

```
Q ss_pred             EEEEEcchhccCCCCCCCcccccccccchHHHHHHHHHHHHhcCCCceEEEeCCCcCCccHHHHHH--HHHHHHc--CC
Q gi|45553317|re  263 TSGTYNDSLLTINSDYDAYLLDFPLMGDDFMLYLARMELKCRFRRHERVLQSGLCVSGLTINGARN--RLKRVQLP--EG  338 (468)
Q Consensus       263 ~Iv~iGDSIT~G~~~~~~~~~~~~~liGds~~~~La~~~l~~~~~~~~~~V~N~G~~isG~ts~~~~~~--rl~~~l~~--~P  338 (468)
                      +|+|+|||||.|........          +|...|++      .+..++.|+|.|  ++|+|+.++..  +++.++..  +|
T Consensus         2 ~i~~~GDS~t~g~~~~~~~~--------gw~~~l~~------~l~~~v~v~N~g--i~G~t~~~~~~~~~~~~~~~~~~~~p   66 (198)
T cd01821         2 TIFLAGDSTVADYDPGAPQA--------GWGQALPQ-----YLDTGITVVNHA--KGGRSSRSFRDEGRWDAILKLIKPG   66 (198)
T ss_pred             EEEEEeeccccCCCCCCCCC--------CHHHHHH-----HhCCCCEEEEec--cCCccHHHHHhCCcHHHHHhcCCCC


Q ss_pred             CeEEEEeeeccCCCCC-----CHHHHHHHHHHHHHHHHHhCCCEEEEEcCCCCCccccchhhHHHHHHHHHHHHHHHhC--C
Q gi|45553317|re  339 TQIIVNIGSVDIMRGK-----PLVQIEHDFRLLIKEMHNMRLVPILTNLAPLGNYCHDKVLCDKIYRFNKFIRSECC--H  411 (468)
Q Consensus       339 DlVvI~lGtND~~~g~-----s~e~f~~~l~~LI~~lr~~ga~VIL~t~~P~~~~~~~~~~~~~~~~~i~~~N~~ir~~A~~-g  411 (468)
                      |+|||++|+||...+.     +.++|.++|++||+.+++.+++|||++++|..........+.+.+|+++++++|+  +
T Consensus        67 d~vii~~G~ND~~~~~~~~~~~~~~~~nl~~ii~~~~~~~~~~vil~tp~~~~~~~~~~~~~~~~~~~~~~y~~~~~~vA~e~~  146 (198)
T cd01821        67 DYVLIQFGHNDQKPKDPEYTEPYTTYKEYLRRYIAEARAKGATPILVTPVTRRTFDEGGKVEDTLGDYPAAMRELAAEEG  146 (198)
T ss_pred             CEEEEEeCCCCCCCCCCCCCCCCCHHHHHHHHHHHHHHHHHHHhCCCEEEEecCcccccCCCCCceecchHHHHHHHHHHhC


Q ss_pred             CEEEECHHHhhhcccccHh-------hcCCCCcCCCCcHHHHHHHHHHHHHH
Q gi|45553317|re  412 LKVIDIHSCLINERGVVRFD-------CFQASPRQVTGSKEPYLFWNKIGRQRV  458 (468)
Q Consensus       412 v~fIDl~~~~~~~~~g~~~~~~~-------~~~dDGvHPn~~~~Gy~~lA~~i~~~I  458 (468)
                      ++|||+++.+.+.+.......+       ++..||+||.  +||++||+.+.++|
T Consensus       147 ~~~iD~~~~~~~~~~~~~~~~~~~~~~~~~~~~~~DgvHpn~--~G~~~~a~~i~~~~  198 (198)
T cd01821       147 VPLIDLNAASRALYEAIGPEKSKKYFPEGPGDNTHFSE--KGADVVARLVAEEL  198 (198)
T ss_pred             CcEEecHHHHHHHHHHCHHHHHHhccCCCCCCCcCH--HHHHHHHHHHHhC
```

No 14

>**cd01830** XynE_like SGNH_hydrolase subfamily, similar to the putative arylesterase/acylhydrolase from the rumen anaerobe Prevotella bryantii XynE. The P. bryantii XynE gene is located in a xylanase gene cluster. SGNH hydrolases are a diverse family of lipases and esterases. The tertiary fold of the enzyme is substantially different from that of the alpha/beta hydrolase family and unique among all known hydrolases; its active site closely resembles the Ser-His-Asp(Glu) triad found in other serine hydrolases.
Probab=99.93 E-value=3.8e-24 Score=206.24 Aligned_cols=185 Identities=17% Similarity=0.121 Sum_probs=0.0

```
Q ss_pred             EEEEEcchhccCCCCCCCcccccccccchHHHHHHHHHHHHhcCCCceEEEeCCCcCCccH------HHHHHHHHHHHc
Q gi|45553317|re  263 TSGTYNDSLLTINSDYDAYLLDFPLMGDDFMLYLARMELKCRFRRHERVLQSGLCVSGLTI------NGARNRLKRVQLP  336 (468)
Q Consensus       263 ~Iv~iGDSIT~G~~~~~~~~~~~~~liGds~~~~La~~~l~~~~~~~~~~V~N~G~~isG~ts------~~~~~rl~~~l~~  336 (468)
                      +|+|||||||.|.........          +|+..++..+.......++.++|.|  ++|.++      .+++.|++..+..
T Consensus         1 ~iv~~GDSiT~G~~~~~~~~-------~~~~~~l~~~l~~~~~~~~~~~~v~N~G--i~G~t~~~~~~~~~~Rl~~~v~~   71 (204)
T cd01830         1 SVVALGDSITDGRGSTPDANN-------RWPDLLAARLAARAGTRGIAVLNAG--IGGNRLLADGLGPSALARFDRDVLS   71 (204)
T ss_pred             CEEEEECccccCcCCCCCCC-------cccHHHHHHHHhcCCCCceEEecC--CCCccccCCChHHHHHHHHHhh


Q ss_pred             C--CCeEEEEeeeccCCCCC--------CHHHHHHHHHHHHHHHHHhCCCEEEEEcCCCCCCccccchhhHHHHH-HHHHHHH
Q gi|45553317|re  337 E--GTQIIVNIGSVDIMRGK--------PLVQIEHDFRLLIKEMHNMRLVPILTNLAPLGNYCHDKVLCDKI-YRFNKFI  405 (468)
Q Consensus       337 ~--PDlVvI~lGtND~~~g~--------s~e~f~~~l~~LI~~lr~~ga~VIL~t~~P~~~~~~~~~~~~~~~i~~~~N~~i  405 (468)
                      .  ||+|+|++|+||+|+..+.         +.++|.++|++|++.+++.+++|++++++++|+.........+.+ ..+|+++
T Consensus        72 ~~~~~V~i~~G~ND~~~~~~~~~~~~~~~~~~~ii~~~~~~~~~vil~ti~P~~~~~~~~~~~~~~~~~~~~~~~~n~~i  151 (204)
T cd01830        72 QPGVRTVIILEGVNDIGASGTDFAAAPVTAEELIAGYRQLIRRAHARGIKVIGATITPFEGSGYYTPAREATRQAVNEWI  151 (204)
T ss_pred             CCCCCEEEEEecccccccccccccCCCHHHHHHHHHHHHHHHHHHhCCCEEEEEccCCCCCccCccCcCCChhHHHHHHHHHHHH


Q ss_pred             HHHhCCCEEEECHHHhhhc--ccccHhhcCCCCcCCCCcHHHHHHHHHHHHHH
Q gi|45553317|re  406 RSECCHLKVIDIHSCLINER--GVVRFDCFQASPRQVTGSKEPYLFWNKIGRQRV  458 (468)
Q Consensus       406 r~~A~gv~fIDl~~~~~~~~~~--g~~~~~~~~~~dDGvHPn~~~~Gy~~lA~~i~~~I  458 (468)
                      ++.+....+||++..|.+..  +...+.+++.||+||.  +||++||+.+..++
T Consensus       152 ~~~~~~~~~~~vD~~~~~~~~~~~~~~~~~~~~~~DglHpn~~~~Gy~~mA~~v~~~~  204 (204)
T cd01830       152 RTSGAFDAVVDFDAALRDPADPSRLRPAYDSGDHLHPND--AGYQAMADAVDLDL  204 (204)
T ss_pred             HhcCCCCEEEEcHHHHcCCCCcccccccchhhcCCCCCCCCH--HHHHHHHHhcCCCC
```

No 15

>**4hf7_A** Putative acylhydrolase; PF13472 family, structural genomics, joint center for struct genomics, JCSG, protein structure initiative; HET: OSE; 1.77A {Bacteroides thetaiotaomicron} SCOP: **c.23.10.0**
Probab=99.92 E-value=1.7e-24 Score=206.15 Aligned_cols=181 Identities=13% Similarity=0.111 Sum_probs=0.0

```
Q ss_pred             CCCCCCccEEEEEcchhccCCCCCCCcccccccccchHHHHHHHHHHHHhcCCCceEEEeCCCcCCccHHHHHHHHHH-H
Q gi|45553317|re  255 MTPTPTILTSGTYNDSLLTINSDYDAYLLDFPLMGDDFMLYLARMELKCRFRRHERVLQSGLCVSGLTINGARNRLKR-V  333 (468)
Q Consensus       255 ~~~~P~~~~Iv~iGDSIT~G~~~~~~~~~~~~~liGds~~~~La~~~l~~~~~~~~~~V~N~G~~isG~ts~~~~~rl~~-~  333 (468)
                      ..+.+...+|+|+|+|||||.|              ++..+...|...           .+.|.|  ++|.++.++..++.. +
T Consensus        20 ~~~~~~~~~~~i~~~GDSit~G-------------~~~~~~~~~~~l~~~-----------~v~n~g--~~G~~~~~~~~~~~~   74 (209)
T 4hf7_A         20 AQPVKKEKRVVFMGNXITEG-------------WVRTHPDFFKTN----------GYIGRG--ISGQTSYQFLLRFREDV   74 (209)
T ss_dssp             CCCCGGGCCEEEEESHHHHH-------------HHHHCHHHHHHH----------TEEEEE--CTTCCHHHHHHHHHHHT
T ss_pred             ccCCCCCCeEEEECChhhh-------------hhhhHHHhcCcC----------ceEEcc--cchhhHHHHHHHhcch


Q ss_pred             HhcCCCeEEEEeeeccCCCC---CCHHHHHHHHHHHHHHHHHhCCCEEEEEcCCCCCccccchhhH---HHHHHHHHHHHHHH
Q gi|45553317|re  334 QLPEGTQIIVNIGSVDIMRG---KPLVQIEHDFRLLIKEMHNMRLVPILTNLAPLGNYCHDKVL---CDKIYRFNKFIRS  407 (468)
Q Consensus       334 l~~~PDlVvI~lGtND~~~g~---s~e~f~~~l~~LI~~lr~~ga~VIL~t~~P~~~~~~~~~~~~~~---~~~i~~~N~~ir~  407 (468)
                      ...+||+|+|++|+||+|+..+   .+.++|.+++++||+.+++.+++|||++++|.........   ...+.++|+++++++
T Consensus        75 ~~~~pd~vvi~~G~ND~~~~~~~~~~~~~~~~~~~l~~li~~~~~~~~~vil~~~~p~~~~~~~~~~~~~~~~n~~~~~~  154 (209)
T 4hf7_A         75 INLSPALVVINAGTNDVAENTGAYNEDYTFGNIASMAELAKANKIKVILTSVLPAAEFPWRREIKDAPQKIQSLNARIEA  154 (209)
T ss_dssp             GGGCCSEEEECCCHHHHTTSSSCCHHHHHHHHHHHHHHHHHHHHTTCEEEEECCCCCSCCTTCTTCCCHHHHHHHHHHHH
T ss_pred             hhcCCCEEEEEEeCCCchhhcCCCCHHHHHHHHHHHHHHHHhCCCEEEEEeCCCcCCCCCCCcccccHHHHHHHHHHHHH


Q ss_pred             HhC--CCEEEECHHHhhhcccccHhhcCCCCcCCCCcHHHHHHHHHHHHHHHHHH
Q gi|45553317|re  408 ECC--HLKVIDIHSCLINERGVVRFDCFQASPRQVTGSKEPYLFWNKIGRQRVLQVI  462 (468)
Q Consensus       408 ~A~--gv~fIDl~~~~~~~g~~~~~~~~~~dDGvHPn~~~~Gy~~lA~~i~~~I~~~L  462 (468)
                      +|+  ++.|||++..+.+.........+..||+|||.  +||++||+.+.+.|.++|
T Consensus       155 ~a~~~~v~~~iD~~~~~~~~~~~~~~~~~~~~~~~Dg~Hpn~~~~G~~~~a~~l~~~l~~~L  209 (209)
T 4hf7_A       155 YAKANKIPFVNYYQPMVVGENKALNPQYTKDGVHPTG--EGYDIMEALIKQAIEKAL  209 (209)
T ss_dssp             HHHHTTCCEECSHHHHEETTTTEECGGGBSSSSBCH--HHHHHHHHHHHHHHHHHC
T ss_pred             HHHHCCCeEEECchhhccccccccCCCCCCCCCCCcCH--HHHHHHHHHHHHHHHhC
```

No 16

>3hp4_A GDSL-esterase; psychrotrophic, monoethylphosphonate, hydrolase; HET: MIR; 1.35A {Pseudoalteromonas SP}
SCOP: c.23.10.0
Probab=99.92 E-value=1e-23 Score=197.90 Aligned_cols=178 Identities=15% Similarity=0.134 Sum_probs=0.0

```
Q ss_pred              ccEEEEEcchhccCCCCCCCccccccccccchHHHHHHHHHHHHhhCCCCceEEEeCCCcCCccHHHHHHHHHHHHhc-CCC
Q gi|45553317|re   261 ILTSGTYNDSLLTINSDYDAYLLDFPLMGDDFMLYLARMELKCRFRRHERVLQSGLCVSGLTINGARNRLKRVQLP-EGT  339 (468)
Q Consensus       261 ~~~Iv~iGDSIT~G~~~~~~~~~~~~~~liGds~~~~La~~~l~~~~~~~~~~V~N~G~~isG~ts~~~~~rl~~~l~~~PD  339 (468)
                       ..+|+++|||||.|....          .+.+|...++..+-..  ..++.++|.|  ++|.++.++..+++.+.. +||
T Consensus         2 ~~~i~~~GDSit~G~g~~~~~~~~~~~~~~~~~~~~~~~~l~~~l~~~~~~~~~~~v~n~g~~~~G~~~~~~~~~~~~~~~~~pd   68 (185)
T 3hp4_A           2 DNTILILGDXLSAAYGLQ---------QEEGWVKLLQDKYDAE--QSDIVLINAS--ISGETSGGALRRLDALLEQYEPT   68 (185)
T ss_dssp             CEEEEEEECTTTTTTSC---------GGGSHHHHHHHHHHHT--TCCEEEEECC--CTTCCHHHHHHHHHHHHHHHHCCS
T ss_pred             CceEEEECCccccccCCC---------CCCCHHHHHHHHHHhc--CCCEEEEECC--cCCccHHHHHHHHHHHHHHHhCCC


Q ss_pred              eEEEEeeeccCCCCCCHHHHHHHHHHHHHHHHHhCCCEEEEEcC--CCCCccccchhhHHHHHHHHHHHHHHHhC--CCEEE
Q gi|45553317|re   340 QIIVNIGSVDIMRGKPLVQIEHDFRLLIKEMHNMRLVPILTNL--APLGNYCHDKVLCDKIYRFNKFIRSECC--HLKVI  415 (468)
Q Consensus       340 lVvI~lGtND~~~g~s~e~f~~~l~~LI~~lr~~ga~VIL~t~~~P~~~~~~~~~~~~~~~~~~~i~~~N~~ir~~A~~~gv~fI  415 (468)
                       +|+|++|+||+..+.+.++|.++++++++++.+++|+++++  ||..       .......+|++++++|+  ++.|+
T Consensus        69 ~vvi~~G~ND~~~~~~~~~~~~~~l~~~i~~~~~~~~~~vil~~~~~p~~~~--------~~~~~~~~~~~~~~a~~~~~~~v  141 (185)
T 3hp4_A          69 HVLIELGANDGLRGFPVKKMQTNLTALVKKSQAANAMTALMEIYIPPNY-------GPRYSKMFTSSFTQISEDTNAHLM  141 (185)
T ss_dssp             EEEEECCHHHHHTTCCHHHHHHHHHHHHHHHHHHTTCEEEEECCCCSTT-------CHHHHHHHHHHHHHHHHHHCCEEE
T ss_pred             EEEEeeccccccccCCHHHHHHHHHHHHHHHHHHHHcCCcEEEEeCCCCCcc-------cHHHHHHHHHHHHHHHHHHcCEEE


Q ss_pred              EChHHHhhhccccHhhcCCCCcCCCCcHHHHHHHHHHHHHHHHHHHHH
Q gi|45553317|re   416 DIHSCLINERGVVRFDCFQASPRQVTGSKEPYLFWNKIGRQRVLQVIET  464 (468)
Q Consensus       416 Dl~~~~~~~~~~g~~~~~~~~~~~dDGvHPn~~~~Gy~~lA~~i~~~I~~~L~~  464 (468)
                       |++.......    .+++..||+|||.  +||+.||+.+.+.|.++|++
T Consensus       142 d~~~~~~~~~~~~~~~~~~~~~~~~Dg~Hpn~~~G~~~~a~~l~~~l~~~l~~  184 (185)
T 3hp4_A         142 NFFMLDIAGK----SDLMQNDSLHPNK--KAQPLIRDEMYDSIKKWLNN  184 (185)
T ss_dssp             CCTTTTTTC----GGGBCTTSSBCT--THHHHHHHHHHHHHHHHHHC
T ss_pred             ehhhhccccC----chhhcCCCCCCCH--HHHHHHHHHHHHHHHHHHHHhC
```

No 17

>4iyj_A GDSL-like protein; PF13472 family, GDSL-like lipase, structural genomics, joint for structural genomics, JCSG; HET: MSE; 1.37A {Bacteroides uniformis} SCOP: c.23.10.0 PDB: 4ppy _A
Probab=99.92 E-value=1.5e-24 Score=206.98 Aligned_cols=179 Identities=14% Similarity=0.128 Sum_probs=0.0

```
Q ss_pred              CCCCccEEEEEcchhccCCCCCCCccccccccccchHHHHHHHHHHHHhhcCCCceEEEeCCCcCCccHHHHHHHHHH-HHh
Q gi|45553317|re   257 PTPTILTSGTYNDSLLTINSDYDAYLLDFPLMGDDFMLYLARMELKCRFRRHERVLQSGLCVSGLTINGARNRLKR-VQL  335 (468)
Q Consensus       257 ~~P~~~~Iv~iGDSIT~G~~~~~~~~~~~~~~~liGds~~~~La~~~l~~~~~~~~~~V~N~G~~isG~ts~~~~~rl~~-l~  335 (468)
                       +.+...+|+|+|||||.|             ++..+...+.          +..+.|.|  ++|.++.++..++. +..
T Consensus        25 ~~~~~~~~i~~~GDSit~G~~~~~~~~~~~~~~~~~~~~~~~~~~~~~~~~~~~~~~~~~~~v~n~g~~~~G~~~~~~~~~~~~~~~~~~~   79 (212)
T 4iyj_A          25 KGKQKDRVVFMGNSITEG-------------WVANDAAFFE----------DNGYVGRG--IGGQTSSHFLLRFREDVIK   79 (212)
T ss_dssp             TCCCTTCEEEEESHHHH-------------HHHHCHHHHH----------HHTEEEEE--CTTCCHHHHHHHHHHHHTGG
T ss_pred             CCCCCCeEEEEcCCHHHh-------------hhhhhHHhcc----------cCcEEEec--ccCCCHHHHHHHHhHhHhh


Q ss_pred              cCCCeEEEEeeeccCCCC---CCHHHHHHHHHHHHHHHHHhCCCEEEEEcCCCCCccccchhhH---HHHHHHHHHHHHHHh
Q gi|45553317|re   336 PEGTQIIVNIGSVDIMRG---KPLVQIEHDFRLLIKEMHNMRLVPILTNLAPLGNYCHDKVL---CDKIYRFNKFIRSEC  409 (468)
Q Consensus       336 ~~PDlVvI~lGtND~~~g~~~s~e~f~~~l~~LI~~lr~~ga~VIL~t~~~P~~~~~~~~~~~~~~~~~~~i~~~N~~ir~~A  409 (468)
                       .+||+|+|++||+.+   .+.++|.++++.+|+.+++.+++.+++||||++|..........   ...+.++|+++++|
T Consensus        80 ~~pd~vvi~~G~ND~~~~~~~~~~~~~~~l~~li~~~~~~~~~~vil~~~p~~~~~~~~~~~~~~~~~~~~~~~n~~l~~~a  159 (212)
T 4iyj_A          80 LAPALVVINAGTNDIAENAGAYNEEYTFGNIVSMVELARANKIKVILTSVLPAAAFGWNPSVKDAPQKIMQLNARIRKYA  159 (212)
T ss_dssp             GCCSEEEECCCHHHHTTTTSSCCHHHHHHHHHHHHHHHHHHTTCEEEEECCCCSCTTCTTCCCHHHHHHHHHHHHHHHHHH
T ss_pred             cCCCEEEEEeeeCCCCCCCCCCCCHHHHHHHHHHHHHHHHHhcCCCeEEEEeCCCCCCCCCCCCCHHHHHHHHHHHHHHHH


Q ss_pred              C--CCEEEEChHHHhhhccccHhhcCCCCcCCCCcHHHHHHHHHHHHHHHHHHHHHHH
Q gi|45553317|re   410 C--HLKVIDIHSCLINERGVVRFDCFQASPRQVTGSKEPYLFWNKIGRQRVLQVI  462 (468)
Q Consensus       410 ~~gv~fIDl~~~~~~~~~~g~~~~~~~~~~~dDGvHPn~~~~Gy~~lA~~i~~~I~~~L  462 (468)
                       +  ++.|||++..+.+.........+..|||||.  +||++||+.+.+.|++|
T Consensus       160 ~~~~v~~iD~~~~~~~~~~~~~~~~~~~~~~~Dg~Hpn~~~G~~~~a~~l~~~l~~~L  212 (212)
T 4iyj_A         160 QENKIPYVDYYSEMVEGDNKALNSSYTRDGVHPTL--EGYKVMEALIKKAIDKVL  212 (212)
T ss_dssp             HHHTCCEECHHHHHEETTTTEECGGGBSSSSSBCH--HHHHHHHHHHHHHHHHHC
T ss_pred             HHCCCeEEEChHhhhhccccccccccccCCCCCCCH--HHHHHHHHHHHHHHHHhC
```

No 18

>4q9a_A TAT pathway signal sequence domain protein; acylhydrolase family, PF13472, structural genomics, joint CE structural genomics, JCSG; HET: SEP; 2.86A {Parabacteroides merdae}
Probab=99.92 E-value=6.7e-24 Score=205.77 Aligned_cols=202 Identities=13% Similarity=0.084 Sum_probs=0.0

```
Q ss_pred              CcCCCCCCccEEEEEcchhccCCCCCCCCcccc-cccccchHHHHHHHHHHHHhhcCCCceEEEeCCCcCCccHHHHHHHH
Q gi|45553317|re   253 HEMTPTPTILTSGTYNDSLLTINSDYDAYLLD-FPLMGDDFMLYLARMELKCRFRRHERVLQSGLCVSGLTINGARNRLK  331 (468)
Q Consensus       253 ~e~~~~P~~~~Iv~iGDSIT~G~~~~~~~~~~~-~~liGds~~~~La~~~l~~~~~~~~~~V~N~G~~isG~ts~~~~~rl~  331 (468)
                       ....+.....+|+|+|||||.|+++.......    ....|.+|+..++.++.+.......+.+.|.|  ++|.++.++..++.
T Consensus        11 ~p~~~~~~~~~Il~iGDSIT~g~~~~~~~~~~~~~gy~~~~~~~L~~~~~~~~~~~~~~~n~g~~~~G~~~~~~~~~~r~~  88 (229)
T 4q9a_A          11 APKINLKKDCVILFQGDSITDCGRDRNSNRCNTMEQFGSGYVLFTATQLLEGKAALQPKIYNRG--ISGNKVYQLRERWE  88 (229)
T ss_dssp             CCCCCCTTCEEEEEESHHHHTTCCTTCSSSSHHHHCSSHHHHHHHHHHHHTGGGCCEEEECC--CTTCCHHHHHHTTTT
T ss_pred             CCCccCCCCCEEEEEcCChhcCCCCCCCCCccccccCCCHHHHHHHHHHHhCCCCCCeEEEE--ECcchHHHHHHHHHHH


Q ss_pred              H-HHhcCCCeEEEEeeeccC------CCCCCHHHHHHHHHHHHHHHHHhC--CCEEEEEcCCCCCC--cccchhhHHHHHHH
Q gi|45553317|re   332 R-VQLPEGTQIIVNIGSVDI------MRGKPLVQIEHDFRLLIKEMHNM--RLVPILTNLAPLGN--YCHDKVLCDKIYR  400 (468)
Q Consensus       332 ~-~l~~~PDlVvI~lGtND~~~~~~~~~g~s~e~f~~~l~~LI~~lr~~~ga~VIL~t~~~P~~~~~~~~~~~~~~~~~i~~  400 (468)
                       . +...+||+|+|++||+      ....+.+.|.+++++++++++.+  ++.+++++|||...   .......+.+.+.
T Consensus        89 ~~~~~~~pd~vii~~G~ND~~~~~~~~~~~~~~~~~~~~i~~~~~~~~~~~~~~~~~~~~~~~~~~~~~~~~~~~~~~~~~~~~~~~  168 (229)
T 4q9a_A          89 IDCLAFQPDVLSILIGVNDYWHTLTHGYKGTVETYENDLRALLKYTKEKLPNTQIVLCEPFTLRDGAAIEDSKWYPMFDE  168 (229)
T ss_dssp             TTTGGGCCSEEEEEECCSHHHHHHHHHHSCCCCHHHHHHHHHHHHHHHHHHHHHHHCTTCEEEEECCCCCTTSTTCCHHHHTTTTHH
T ss_pred             HHHHhCCCCEEEEeccccCCCCCcCCcccCCcccHHHHHHHHHHHHHHhCCCCeEEEEcccccCCCCCCCcchhHHHHHH


Q ss_pred              HHHHHHHHhC--CCEEEEChHHHhhhccccHhhcCCCCcCCCCcHHHHHHHHHHHHHHHHH
Q gi|45553317|re   401 FNKFIRSECC--HLKVIDIHSCLINERGVVRFDCFQASPRQVTGSKEPYLFWNKIGRQRV  458 (468)
```

```
Q Consensus       401 ~N~~ir~~A~--gv~fIDl~~~~~~~~g~~~~~~~~dDGvHPn~~~~Gy~~lA~~i~~~I  458 (468)
                      ||++++++|.  ++.|||+++.+.+..+.....++..|++|||+  +||++||+.+.++|
T Consensus       169 ~~~~~~~~a~~~~~~~vd~~~~~~~~~~~~~~~~~~~~~D~vHpn~--~G~~~mA~~~l~al  226 (229)
T 4q9a_A          169 FRKSARKLSEEFNTIFVPFQSGFDAAVKLAPARYWSNDGVHPDL--PGRQLMANMWMEAT  226 (229)
T ss_dssp           HHHHHHHHHHHTTCEEECHHHHHHHHTTTSCHHHHCSSSSSCCH--HHHHHHHHHHHHHH
T ss_pred           HHHHHHHHHhhcCCEEEecHHHHHHHhhcccccccCCCCCCCCH--HHHHHHHHHHHHHh
```

No 19

>3p94_A GDSL-like lipase; serine hydrolase, catalytic triad, flavodo structural genomics, joint center for structural genomics; HET: MSE PG4; 1.93A {Parabacteroides distasonis} SCOP: c.23.10.0
Probab=99.92 E-value=2.8e-24 Score=203.96 Aligned_cols=180 Identities=17% Similarity=0.178 Sum_probs=0.0

```
Q ss_pred             CCCCCccEEEEEcchhccCCCCCCCcccccccccchHHHHHHHHHHHHhhcCCCCceEEEeCCCcCCccHHHHHHHHHH-HH
Q gi|45553317|re  256 TPTPTILTSGTYNDSLLTINSDYDAYLLDFPLMGDDFMLYLARMELKCRFRRHERVLQSGLCVSGLTINGARNRLKR-VQ  334 (468)
Q Consensus       256 ~~~P~~~~Iv~iGDSIT~G~~~~~~~~~~~~~~liGds~~~~La~~~l~~~~~~~~~~~~V~N~G~~isG~ts~~~~~rl~~-~l  334 (468)
                      ...|...+|+|+|||||.|              ++..+...|...            .++|.|  ++|.++.++..++.. +.
T Consensus        17 ~~~~~~~~~i~~~GDSit~g-------------~~~~~~~~~~~l~~~-----------~v~n~g--~~G~~~~~~~~~~~~~~~   71 (204)
T 3p94_A         17 KTVKVPSNVVFMGNSITDG-------------WWPADSTFFIRN----------NFVDRG--ISGQTTSEMLVRFRQDVI   71 (204)
T ss_dssp           TTSCSSEEEEEEESHHHHT-------------HHHHCTTHHHHH----------TEEEEE--CTTCCHHHHHHHHHHHTG
T ss_pred           cccCCCCeEEEEecchhcc-------------cchhHHHhcccC----------ceEecc--ccchhHHHHHHHHHHhHHh
```

```
Q ss_pred             hcCCCeEEEEeeeccCCCC---CCHHHHHHHHHHHHHHHHHhCCCEEEEEcCCCCCccccchh--hHHHHHHHHHHHHHHHh
Q gi|45553317|re  335 LPEGTQIIVNIGSVDIMRG---KPLVQIEHDFRLLIKEMHNMRLVPILTNLAPLGNYCHDK--VLCDKIYRFNKFIRSEC  409 (468)
Q Consensus       335 ~~~PDlVvI~lGtND~~~g~~~s~e~f~~~l~~LI~~lr~~ga~VIL~t~~P~~~~~~~~~~--~~~~~~i~~~N~~ir~~A  409 (468)
                      ..+||+|+|++|+|+..+   .+.++|.++++++|+.+++.+++|+++++++|........  .....+.++|++++++|
T Consensus        72 ~~~pd~vvi~~G~ND~~~~~~~~~~~~~~~~~~~l~~~i~~~~~~~~~~~vil~~~~p~~~~~~~~~~~~~~~~~~~~~n~~l~~~a  151 (204)
T 3p94_A         72 NLKPKAVVILAGINDIAHNNGVIALENVFGNLVSMAELAKANHIKVIFCSVLPAYDFPWRPGMQPADKVIQLNKWIKEYA  151 (204)
T ss_dssp           GGCEEEEEEECCHHHHTTTTSCCCHHHHHHHHHHHHHHHHHHHHTTCEEEEECCCCCSCBTTBTTCCCHHHHHHHHHHHHHHH
T ss_pred           hcCcCEEEEEEEecCcccccCCCCHHHHHHHHHHHHHHHHHcCCCeEEEEeCCCcCccCCCccccHHHHHHHHHHHHHHHHH
```

```
Q ss_pred             C--CCEEEEChHHHhhhcccccHhhcCCCCcCCCCcHHHHHHHHHHHHHHHHHHHH
Q gi|45553317|re  410 C--HLKVIDIHSCLINERGVVRFDCFQASPRQVTGSKEPYLFWNKIGRQRVLQVIE  463 (468)
Q Consensus       410 ~--gv~fIDl~~~~~~~~g~~~~~~~~dDGvHPn~~~~Gy~~lA~~i~~~I~~~L~  463 (468)
                      +  ++.|||++..+.+..+.....++.|+ +|||.  +||++||+.+.+.|.++|+
T Consensus       152 ~~~~v~~iD~~~~~~~~~~~~~~~~~~~Dg-~Hpn~--~G~~~~a~~l~~~l~~~l~  204 (204)
T 3p94_A         152 DKNGLTYVDYHSAMKDERNGLPANLSKDG-VHPTL--EGYKIMEKIVLEAIHKTVK  204 (204)
T ss_dssp           HHTTCEEECHHHHHCCTTSSCCTTTBSSS-SSBCH--HHHHHHHHHHHHHHHHHHCC
T ss_pred           HHcCCeEEeccchhhhcccccccccCCCC-CCcCH--HHHHHHHHHHHHHHHHHHhC
```

No 20

>cd01835 SGNH_hydrolase_like_3 SGNH_hydrolase subfamily. SGNH hydrolases are a diverse family of lipases and esterases. The tertiary fold of the enzyme is substantially different from that of the alpha/beta hydrolase family and unique among all known hydrolases; its active site closely resembles the Ser-His-Asp(Glu) triad found in other serine hydrolases.
Probab=99.92 E-value=1.4e-23 Score=200.06 Aligned_cols=179 Identities=14% Similarity=0.104 Sum_probs=0.0

```
Q ss_pred             ccEEEEEcchhccCCCCCCCcccccccccchHHHHHHHHHHHHhhcCCCCceEEEeCCCcCCccHHHHHHHHHHHHh-----
Q gi|45553317|re  261 ILTSGTYNDSLLTINSDYDAYLLDFPLMGDDFMLYLARMELKCRFRRHERVLQSGLCVSGLTINGARNRLKRVQL-----  335 (468)
Q Consensus       261 ~~~Iv~iGDSIT~G~~~~~~~~~~~~~~liGds~~~~La~~~l~~~~~~~~~~~~V~N~G~~isG~ts~~~~~rl~~~l~-----  335 (468)
                      .++|+++|||||.|.+..          .+.+|..++...+...  ...+.+.|.|  ++|.|+.+++.++++...+.
T Consensus         1 ~~~iv~lGDSit~G~g~~~~~~~~~~~~~~w~~~l~~~l~~~~~~~~~~v~N~G~~~G~tt~~~~~~r~~~~~~~~~~~~~  67 (193)
T cd01835          1 PKRLIVVGDSLVYGWGDP---------EGGGWVGRLRARWMNL--GDDPVLYNLG--VRGDGSEDVAARWRAEWSRRGEL  67 (193)
T ss_pred           CeEEEEEccccccCCC---------CCCccHHHHHHHHhcc--CCceEEEEcc--CCCCchHHHHHHHHHHHHHhccC
```

```
Q ss_pred             cCCCeEEEEeeeccCCCC------CCHHHHHHHHHHHHHHHHHhCCCEEEEEcCCCCCccccchhhHHHHHHHHHHHHHHHHHh
Q gi|45553317|re  336 PEGTQIIVNIGSVDIMRG------KPLVQIEHDFRLLIKEMHNMRLVPILTNLAPLGNYCHDKVLCDKIYRFNKFIRSEC  409 (468)
Q Consensus       336 ~~PDlVvI~lGtND~~~g------~s~e~f~~~l~~LI~~lr~~ga~VIL~t~~P~~~~~~~~~~~~~~~~i~~~N~~ir~~A  409 (468)
                      .+||+|+|++|+|+..+      .+.+.|.++++.++  .+.+|+++++++|+......  ..++.+..||+++++|
T Consensus        68 ~~pd~V~i~~G~ND~~~~~~~~~~~~~~~~~~~~~~li~~~~~~v~vi~~~p~~~~~~~~~~~~~~~~~~~~~~~~~n~~~~~~~a  145 (193)
T cd01835         68 NVPNRLVLSVGLNDTARGGRKRPQLSARAFLFGLNQLLEEAK-RLVPVLVVGPTPVDEAKMP-YSNRRIARLETAFAEVC  145 (193)
T ss_pred           CCCCEEEEEecccchhccccCCcccCHHHHHHHHHHHHHHHH-hcCCEEEEcCCCcccccch-HHHHHHHHHHHHHHHHHH
```

```
Q ss_pred             C--CCEEEEChHHHhhhcccccHhhcCCCCcCCCCcHHHHHHHHHHHHHHH
Q gi|45553317|re  410 C--HLKVIDIHSCLINERGVVRFDCFQASPRQVTGSKEPYLFWNKIGRQR  457 (468)
Q Consensus       410 ~--gv~fIDl~~~~~~~~g~~~~~~~~dDGvHPn~~~~Gy~~lA~~i~~~  457 (468)
                      +  +++|||++..|.+.......++..||+|||.  +||++||+.|++.
T Consensus       146 ~~~~~~~~~iD~~~~~~~~~~~~~~~~~~~~~~DgvHpn~--~Gy~~~a~~i~~~  192 (193)
T cd01835        146 LRRDVPFLDTFTPLLNH-PQWRRELAATDGIHPNA--AGYGWLAWLVLHW  192 (193)
T ss_pred           HHcCCcEEcccchhhhc-hhHHHhcccCCCCCCCH--HHHHHHHHHHhcC
```

No 21

>cd01822 Lysophospholipase_L1_like Lysophospholipase L1-like subgroup of SGNH-hydrolases. The best characterized member in this family is TesA, an E. coli periplasmic protein with thioesterase, esterase, arylesterase, protease and lysophospholipase activity.
Probab=99.92 E-value=2.7e-23 Score=193.89 Aligned_cols=173 Identities=17% Similarity=0.100 Sum_probs=0.0

```
Q ss_pred             EEEEEcchhccCCCCCCCcccccccccchHHHHHHHHHHHHhhcCCCCceEEEeCCCcCCccHHHHHHHHHHHHhc-CCCeE
Q gi|45553317|re  263 TSGTYNDSLLTINSDYDAYLLDFPLMGDDFMLYLARMELKCRFRRHERVLQSGLCVSGLTINGARNRLKRVQLP-EGTQI  341 (468)
Q Consensus       263 ~Iv~iGDSIT~G~~~~~~~~~~~~~~liGds~~~~La~~~l~~~~~~~~~~~~V~N~G~~isG~ts~~~~~rl~~~l~~-~PDlV  341 (468)
                      +|+++|||||.|....          .+.+|+..|+.++.  .....+.++|.|  ++|.++.++..+++..+.. +|||+|
T Consensus         2 ~i~~~GDSit~G~g~~~~~~~~~~~~~~~~~~l~~~l~~~~~~~~v~N~g~~~G~~~~~~~~~~~~~~~~~~~~~pd~v   68 (177)
T cd01822          2 TILALGDSLTAGYGLP---------PEEGWPALLQKRLD--ARGIDVTVINAG--VSGDTTAGGLARLPALLAQHKPDLV   68 (177)
T ss_pred           eEEEEccccCCCCC---------CCCchHHHHHHHHH--hcCCCeEEEecc--ccCChHHHHHHHHHHHhhcCCCEE
```

```
Q ss_pred             EEEeeeccCCCCCCHHHHHHHHHHHHHHHHHhCCCEEEEEcCCCCCccccchhhHHHHHHHHHHHHHHHHHhC--CCEEEEChH
Q gi|45553317|re  342 IVNIGSVDIMRGKPLVQIEHDFRLLIKEMHNMRLVPILTNLAPLGNYCHDKVLCDKIYRFNKFIRSECC--HLKVIDIHS  419 (468)
Q Consensus       342 vI~lGtND~~~g~s~e~f~~~l~~LI~~lr~~ga~VIL~t~~P~~~~~~~~~~~~~~~~i~~~N~~ir~~A~--gv~fIDl~~  419 (468)
                      +|++|+|+..+.+.+.+++..++++|+.+++.+++|+++++++|......      +....+|+++++|+  ++.|+|.+.
T Consensus        69 i~~~G~ND~~~~~~~~~~~~~~~~li~~~~~~~~~~~~~vil~~~~~~~~~~~~~~~~~~~~~~~~~~n~~~~~~a~~~~~~~~id~~~~  143 (177)
```

```
T cd01822          69 ILELGGNDGLRGIPPDQTRANLRQMIETAQARGAPVLLVGMQAPPNYGP-----RYTRRFAAIYPELAEEYGVPLVPFFL  143 (177)
T ss_pred             EEeccccccccCCHHHHHHHHHHHHHHHHHCCCeEEEEecCCCCccch-----HHHHHHHHHHHHHHHHcCCeeehhhh


Q ss_pred             HHhhhcccccHhhcCCCCcCCCCcHHHHHHHHHHHHHHH
Q gi|45553317|re  420 CLINERGVVRFDCFQASPRQVTGSKEPYLFWNKIGRQRVL  459 (468)
Q Consensus      420 ~~~~~~g~~~~~~~~dDGvHPn~~~~Gy~~lA~~i~~~I~  459 (468)
                    ..    .....+++..||+|||+  +||++||+.+.+.|+
T Consensus      144 ~~--------------DgiHpn~--~G~~~~a~~i~~~l~  177 (177)
T cd01822      144 EG----VAGDPELMQSDGIHPNA--EGQPIIAENVWPALE  177 (177)
T ss_pred             hh----hcccchhccCCCCCCCH--HHHHHHHHHHHHhhC
```

No 22

>**3mil_A** Isoamyl acetate-hydrolyzing esterase; SGNH-hydrolase, hydrolase; 1.60A {Saccharomyces cerevisiae}
Probab=99.92 E-value=1.9e-23 Score=203.13 Aligned_cols=188 Identities=13% Similarity=0.144 Sum_probs=0.0

```
Q ss_pred             EEEEEcchhccCCCCCCCcccccccc--chHHHHHHHHHHHhhcCCCceEEEeCCCcCCccHHHHHHHHHHHh--cCC
Q gi|45553317|re  263 TSGTYNDSLLTINSDYDAYLLDFPLMG--DDFMLYLARMELKCRFRRHERVLQSGLCVSGLTINGARNRLKRVQL--PEG  338 (468)
Q Consensus      263 ~Iv~iGDSIT~G~~~~~~~~~~~~~~liG--ds~~~~La~~~l~~~~~~~~~~~V~N~G~~isG~ts~~~~~~rl~~~l~----P  338 (468)
                    +|+++|||||.++........    .|  .+|...|+.     .+..++.+.|.|  ++|.++.++..+++..+.  .+|
T Consensus        5 ~i~~~GDSit~~g~~~~~~~~~----~g~~~~~~~~~l~~------~~~~~~~~v~n~g--~~~G~t~~~~~~~l~~~l~~~~~~p   73 (240)
T 3mil_A          5 KFLLFGDSITEFAFNTRPIED----GKDQYALGAALVN-----EYTRKMDILQRG--FKGYTSRWALKILPEILKHESNI   73 (240)
T ss_dssp             EEEEEESHHHHTTTCSCCSTT----CCCCCCHHHHHHH-----HTTTTEEEEEEE--CTTCCHHHHHHHHHHHHHHHCCCE
T ss_pred             eEEEEeccccccccccCccc----cchHhHHHHHHHH-----HcCCCeEEEecC--CCcccHHHHHHHHHHHhcccCCC


Q ss_pred             CeEEEEeeeccC----CCCCCHHHHHHHHHHHHHHHHhCCCEEEEEcCCCCCccccchhh-----------HHHHHHHHHH
Q gi|45553317|re  339 TQIIVNIGSVDI----MRGKPLVQIEHDFRLLIKEMHNMRLVPILTNLAPLGNYCHDKV-----------LCDKIYRFNK  403 (468)
Q Consensus      339 DlVvI~lGtND~----~~g~s~e~f~~~l~~LI~~lr~~ga~VIL~t~~P~~~~~~~~~~~~~~~~~----------~~~~i~~~N~  403 (468)
                    |+|+|++|+|+|+    ..+.+.++|.++++++|+.+++.+++||++++|+........               .++.+..||+
T Consensus       74 d~vvi~~G~ND~~~~~~~~~~~~~~~~~~~~~~~l~~~~i~~~~~~~~~~~vil~~~~~~~~~~~~~~~~~~~~~~~~~~~~~~~n~  153 (240)
T 3mil_A         74 VMATIFLGANDACSAGPQSVPLPEFIDNIRQMVSLMKSYHIRPIIIGPGLVDREKWEKEKSEEIALGYFRTNENFAIYSD  153 (240)
T ss_dssp             EEEEEECCTTTTSSSSTTCCCHHHHHHHHHHHHHHHHHHHTTCEEEEECCCCCHHHHHHHCHHHHHTTCCCCHHHHHHHHH
T ss_pred             CEEEEEeecccCCccCCCCCCCHHHHHHHHHHHHHHHHhcCCCcEEEECCCCCCCcccchhhhcccccccchhhHHHHHHHH


Q ss_pred             HHHHHhC--CCEEEECHhHHHhhhcccccHhhcCCCCcCCCCcHHHHHHHHHHHHHHHHHHHHH
Q gi|45553317|re  404 FIRSECC--HLKVIDIHSCLINERGVVRFDCFQASPRQVTGSKEPYLFWNKIGRQRVLQVIET  464 (468)
Q Consensus      404 ~ir~~A~~--gv~fIDl~~~~~~~~~g~~~~~~~~~dDGvHPn~~~~Gy~~lA~~i~~~I~~~L~~  464 (468)
                    +++++|+  ++.|||++..|.+..+.....++.|+  +|||.  +||++||+.+.+|.+.+.+
T Consensus      154 ~~~~~a~~~~v~~vD~~~~~~~~~~~~~~~~~~~~~Dg~Hpn~--~G~~~~a~~l~~~l~~~~p~  213 (240)
T 3mil_A         154 ALAKLANEEKVPFVALNKAFQQEGGDAWQQLLTDG-LHFSG--KGYKIFHDELLKVIETFYPQ  213 (240)
T ss_dssp             HHHHHHHHTTCCEECHHHHHHHHHGGGGGGGBSSS-SSBCH--HHHHHHHHHHHHHHHHHHCGG
T ss_pred             HHHHHHHHcCCeEEehHHHHHhhcccccHHHHhccC-CCcCH--HHHHHHHHHHHHHHHHhccc
```

No 23

>**cd01820** PAF_acetylesterase_like PAF_acetylhydrolase (PAF-AH)_like subfamily of SGNH-hydrolases.
Platelet-activating factor (PAF) and PAF-AH are key players in inflammation and in atherosclerosis. PAF-AH is a calcium independent phospholipase A2 which exhibits strong substrate specificity towards PAF, hydrolyzing an acetyl ester at the sn-2 position. PAF-AH also degrades a family of oxidized PAF-like phospholipids with short sn-2 residues.
In addition, PAF and PAF-AH are associated with neural migration and mammalian reproduction.
Probab=99.92 E-value=1.4e-23 Score=204.41 Aligned_cols=177 Identities=14% Similarity=0.175 Sum_probs=0.0

```
Q ss_pred             CccEEEEEcchhccCCCCCCCcccccccccchHHHHHHHHHHHhhcCCCceEEEeCCCcCCccHHHHHHHHH--HHHhcC
Q gi|45553317|re  260 TILTSGTYNDSLLTINSDYDAYLLDFPLMGDDFMLYLARMELKCRFRRHERVLQSGLCVSGLTINGARNRLK--RVQLPE  337 (468)
Q Consensus      260 ~~~~Iv~iGDSIT~G~~~~~~~~~~~~~~liGds~~~~La~~~l~~~~~~~~~~~V~N~G~~isG~ts~~~~~~rl~~--~~l~~~  337 (468)
                    ...+|+|+|||||+|.....           ...|...+.           ++.++|.|  ++|.++.+++.|++   .+...+
T Consensus       31 ~~~~iv~~GDSit~g~~~~~---------------~~~~~~~~~~~~~---------~~~~~v~N~G~~i~G~tt~~~l~rl~~~~~~~~~~~   89 (214)
T cd01820       31 KEPDVVFIGDSITQNWEFTG---------LEVWRELYA----------PLHALNFG--IGGDRTQNVLWRLENGELDGVN   89 (214)
T ss_pred             CCCCEEEEccHHhhhcccc---------hhHHHHHcC----------CCCeEEee--eCcccHHHHHHHhcCCcccCC


Q ss_pred             CCeEEEEeeeccCCCCCCHHHHHHHHHHHHHHHHhC--CCEEEEEcCCCCCccccchhHHHHHHHHHHHHHHHHHhC---CC
Q gi|45553317|re  338 GTQIIVNIGSVDIMRGKPLVQIEHDFRLLIKEMHNM--RLVPILTNLAPLGNYCHDKVLCDKIYRFNKFIRSECC---HL  412 (468)
Q Consensus      338 PDlVvI~lGtND~~~g~s~e~f~~~l~~LI~~lr~~--~ga~VIL~t~~P~~~~~~~~~~~~~~~~~~i~~~N~~ir~~A~~---gv  412 (468)
                    ||+|+|++|+|||+..+.+.+.+++.+++++|+.+++.  +++|++++|++|+....    .....+..++|+.+++.++   ++
T Consensus       90 Pd~VvI~~GtND~~~~~~~~~~~~li~~ir~~~p~~~Ivl~~~~~~~~~~~~~~~~~~~~~~~~~~~~n~~l~~~v  167 (214)
T cd01820       90 PKVVVLLIGTNNIGHTTTAEEIAEGILAIVEEIREKLPNAKILLLGLLPRGQN--PNPLRERNAQVNRLLAVRYDGLPNV  167 (214)
T ss_pred             CCEEEEEeccccCCCCCHHHHHHHHHHHHHHHHHHHCCCCEEEEEeCCCCCCC--cchHHHHHHHHHHHHHHHHhcCCCe


Q ss_pred             EEEECHhHHHhhhcccccHhhcCCCCcCCCCcHHHHHHHHHHHHHHHHHHH
Q gi|45553317|re  413 KVIDIHSCLINERGVVRFDCFQASPRQVTGSKEPYLFWNKIGRQRVLQVI  462 (468)
Q Consensus      413 ~fIDl~~~~~~~~~g~~~~~~~~~dDGvHPn~~~~Gy~~lA~~i~~~I~~~L  462 (468)
                    .|||++..|.+..+.....++.+|+  +||||+|||++||+.++++
T Consensus      168 ~~iD~~~~~~~~~~~~~~~~~~~~~~~~~~~DG-lHPn~~~~Gy~~~~A~~~l~~~l~~~~  214 (214)
T cd01820      168 TFLDIDKGFVQSDGTISHHDMPDY-LHLTA--AGYRKWADALHPTLARLL  214 (214)
T ss_pred             EEEeCchhhcCCCCCcChhhcCCC-CCCCH--HHHHHHHHHHHHHHHHhC
```

No 24

>PRK10528 multifunctional acyl-CoA thioesterase I and protease I and lysophospholipase L1; Provisional
Probab=99.92 E-value=2.2e-23 Score=199.82 Aligned_cols=173 Identities=16% Similarity=0.127 Sum_probs=0.0

```
Q ss_pred             EEEEEcchhccCCCCCCCcccccccccchHHHHHHHHHHHhhcCCCceEEEeCCCcCCccHHHHHHHHHHHhc-CCCeE
Q gi|45553317|re  263 TSGTYNDSLLTINSDYDAYLLDFPLMGDDFMLYLARMELKCRFRRHERVLQSGLCVSGLTINGARNRLKRVQLP-EGTQI  341 (468)
Q Consensus      263 ~Iv~iGDSIT~G~~~~~~~~~~~~~~liGds~~~~La~~~l~~~~~~~~~~~V~N~G~~isG~ts~~~~~~rl~~~l~~-~PDlV  341 (468)
                    +|+|+|||+|.|.+..          -+.+|+..|+.++    ...+.|+|.|  ++|.|++.+.++.+.+..+..|  +|+
T Consensus       12 ~i~~~GDSlT~G~~~~~~~~~~~~~~w~~~L~~~l~------~~~~~v~N~G--i~G~tt~~~~rl~~~~~~~~~pd~V   75 (191)
T PRK10528       12 TLLILGDSLSAGYRMP---------ASAAWPALLNDKW-----QSKTSVVNAS--ISGDTSQQGLARLPALLKQHQPRWV   75 (191)
T ss_pred             eEEEEECccccCcccC---------ccCchHHHHHHH-----hhCCCeEeCC--cchhHHHHHHHHHHHHhcCCCEE


Q ss_pred             EEEeeeccCCCCCCHHHHHHHHHHHHHHHHhCCCEEEEE-cCCCCCccccchhHHHHHHHHHHHHHHHHHHhC--CCEEEECh
Q gi|45553317|re  342 IVNIGSVDIMRGKPLVQIEHDFRLLIKEMHNMRLVPILT-NLAPLGNYCHDKVLCDKIYRFNKFIRSECC--HLKVIDIH  418 (468)
```

```
Q Consensus       342 vI~lGtND~~~g~s~e~f~~~l~~LI~~lr~~ga~VIL~-t~~P~~~~~~~~~~~~~~~i~~~N~~ir~~A~--gv~fIDl~  418 (468)
                      ||++|+||+.++.+.++|.+++++|++.+++.+..++++ ...|...      .......|+++++++|+  ++.|||++
T Consensus        76 ii~~G~ND~~~~~~~~~~~~nl~~li~~~~~~~~~~~il~~~~~p~~~------~~~~~~~~~~~~~~~~A~~~~~~~id~~  149 (191)
T PRK10528         76 LVELGGNDGLRGFPPQQTEQTLRQIIQDVKAANAQPLLMQIRLPANY------GRRYNEAFSAIYPKLAKEFDIPLLPFF  149 (191)
T ss_pred              EEEccccccccCCCHHHHHHHHHHHHHHHHHhcCCcEEEEeccCCCcc------chHHHHHHHHHHHHHHHHHhCCccccchh


Q ss_pred              HHHhhhcccccHhhcCCCCcCCCCcHHHHHHHHHHHHHHHHHHHHH
Q gi|45553317|re  419 SCLINERGVVRFDCFQASPRQVTGSKEPYLFWNKIGRQRVLQVIE  463 (468)
Q Consensus       419 ~~~~~~~g~~~~~~~~~dDGvHPn~~~~Gy~~lA~~i~~~I~~~L~  463 (468)
                      ...    .....+++..||+|||.  .||+.||+.+++.|.+++.
T Consensus       150 ~~~----~~~~~~~~~~~DGiHpn~-~~G~~~~A~~i~~~i~~~~~  188 (191)
T PRK10528        150 MEE----VYLKPQWMQDDGIHPNR--DAQPFIADWMAKQLQPLVN  188 (191)
T ss_pred              hhh----cccCcccCCCCCCCcCH--HHHHHHHHHHHHHHHHHHc
```

No 25

>**cd01828** sialate_O-acetylesterase_like2 sialate_O-acetylesterase_like subfamily of the SGNH-hydrolases, a diverse family of lipases and esterases. The tertiary fold of the enzyme is substantially different from that of the alpha/beta hydrolase family and unique among all known hydrolases; its active site closely resembles the Ser-His-Asp(Glu) triad found in other serine hydrolases.
Probab=99.92 E-value=3.3e-23 Score=192.57 Aligned_cols=164 Identities=17% Similarity=0.258 Sum_probs=0.0

```
Q ss_pred              EEEEEcchhccCCCCCCCccccccccchHHHHHHHHHHHhhcCCCceEEEeCCCcCCccHHHHHHHHHHHHhcCCCeEE
Q gi|45553317|re  263 TSGTYNDSLLTINSDYDAYLLDFPLMGDDFMLYLARMELKCRFRRHERVLQSGLCVSGLTINGARNRLKRVQLPEGTQII  342 (468)
Q Consensus       263 ~Iv~iGDSIT~G~~~~~~~~~~~~~~liGds~~~~La~~~l~~~~~~~~~~~V~N~G~~isG~ts~~~~~~rl~~~l~~~PDlVv  342 (468)
                      +|+|+|||||.|..                          ......++.+.|.|  ++|.++.++..+++..+..+||+|+
T Consensus          1 ~i~~~GDSi~~g~~------------------------~~~~~~~~~~~v~n~g--~~G~~~~~~~~~~l~~~~~~~~~d~vv   53 (169)
T cd01828          1 ALVFLGDSLTEGGP-------------------------WALLFPDVKVANRG--ISGDTTRGLLARLDEDVALQPKAIF   53 (169)
T ss_pred              CEEEEechHhccCc-------------------------hHHhCCCCceEEcc--cCCccHHHHHHHhhhcCCCCEEE


Q ss_pred              EEeeeccCCCCCCHHHHHHHHHHHHHHHHHh--CCCEEEEEcCCCCCcccchhhHHHHHHHHHHHHHHHHhC--CCEEEECh
Q gi|45553317|re  343 VNIGSVDIMRGKPLVQIEHDFRLLIKEMHN--MRLVPILTNLAPLGNYCHDKVLCDKIYRFNKFIRSECC--HLKVIDIH  418 (468)
Q Consensus       343 I~lGtND~~~g~s~e~f~~~l~~LI~~lr~~-ga~VIL~t~~P~~~~~~~~~~~~~~~~~i~~~N~~ir~~A~--gv~fIDl~  418 (468)
                      |++|+||+..+.+.+.+.|.++++++|+.+++  .+.+|++++++|..  .........+..+|+.++++|+  ++.|||++
T Consensus        54 ~~G~ND~~~~~~~~~~~~~~~l~~li~~i~~~~~~~~~vi~~~~~~~~-~~~~~~~~~~~~~~~~~n~~~~~~a~~~~~~~~id~~  131 (169)
T cd01828         54 IMIGINDLAQGTSDEDIVANYRTILEKLRKHFPNIKIVVQSILPVG--ELKSIPNEQIEELNRQLAQLAQQEGVTFLDLW  131 (169)
T ss_pred              EEEEecCCCCCCCCHHHHHHHHHHHHHHHHHHHCCCCEEEEEecCCcC--ccccccHHHHHHHHHHHHHHHhCCCEEEEeCc


Q ss_pred              HHHhhhcccccHhhcCCCCcCCCCcHHHHHHHHHHHHHH
Q gi|45553317|re  419 SCLINERGVVRFDCFQASPRQVTGSKEPYLFWNKIGRQRV  458 (468)
Q Consensus       419 ~~~~~~~g~~~~~~~~~dDGvHPn~~~~Gy~~lA~~i~~~I  458 (468)
                      ..+.+..+ ....++..||+|||+  +||+.||+.+.+.|
T Consensus       132 ~~~~~~~~~-~~~~~~~~~DgiHp~~--~~Gy~~~a~~i~~~i  168 (169)
T cd01828        132 AVFTNADG-DLKNEFTTDGLHLNA--KGYAVWAAALQPYL  168 (169)
T ss_pred              HHHhccCC-CCchhcCCCCCCcCH--HHHHHHHHHHHHh
```

No 26

>**4c1b_A** ZFL2-1 ORF1P, ORF1-encoded protein; hydrolase, retrotransposition, RNA-binding, membrane-binding lipid-binding, SELF-association; 2.50A {Danio rerio}
Probab=99.92 E-value=1.7e-23 Score=193.91 Aligned_cols=163 Identities=11% Similarity=0.149 Sum_probs=0.0

```
Q ss_pred              ccEEEEEcchhccCCCCCCCccccccccchHHHHHHHHHHHhhcCCCceEEEeCCCcCCccHHHHHHHHHHH--hcCC
Q gi|45553317|re  261 ILTSGTYNDSLLTINSDYDAYLLDFPLMGDDFMLYLARMELKCRFRRHERVLQSGLCVSGLTINGARNRLKRVQ--LPEG  338 (468)
Q Consensus       261 ~~~Iv~iGDSIT~G~~~~~~~~~~~~~~liGds~~~~La~~~l~~~~~~~~~~~V~N~G~~isG~ts~~~~~~rl~~~l-~~~~P  338 (468)
                      ..+|+|+|||||.|.....                           ..++.+.|.|  ++|.++.++..+++..+  ..+|
T Consensus          3 ~~~i~~~GDSit~g~~~~~~~--------------------------~~~~~~v~n~g~~~G~~~~~~~~~~~~~~~~~~~~~p   54 (171)
T 4c1b_A          3 AMDVAIIGDSIVRHVRAAS--------------------------SKGNKVRTFC--FPGARVKNISTQIPTILGAAESP   54 (171)
T ss_dssp              SSCEEEEESGGGGCCCC--------------------------CTTCCEEEEE--CTTCCHHHHHHHHHHHSSTTCC
T ss_pred              CceEEEEcchhhhcCCCCh--------------------------hcCCEEEEee--cCChhHHHHHHHHhhccccCCCC


Q ss_pred              CeEEEEeeeccCCCCCCHHHHHHHHHHHHHHHHHHhCC--CEEEEEcCCCCCcccchhhHHHHHHHHHHHHHHHHhC--CCEE
Q gi|45553317|re  339 TQIIVNIGSVDIMRGKPLVQIEHDFRLLIKEMHNMR--LVPILTNLAPLGNYCHDKVLCDKIYRFNKFIRSECC--HLKV  414 (468)
Q Consensus       339 DlVvI~lGtND~~~g~s~e~f~~~l~~LI~~lr~~g--a~VIL~t~~P~~~~~~~~~~~~~~~~~i~~~N~~ir~~A~--gv~f  414 (468)
                      |+|+|++|+||+..+.+ ++|.++++++|+.+++.+  ++|++++++|.....  .....++.++|+++++|+  ++.|
T Consensus        55 d~vvi~~G~ND~~~~~~~~~~~~~~i~~~~~~~~~P~~~~~vi~~~~~~p~~~~~~~~~~~~~~~~~~~n~~~~~~a~~~~v~~  131 (171)
T 4c1b_A         55 GAVVLHVGTNDTGLRQS-EILKKDFRSLIETVRRTSPATQIIVSGPLPTYRRG--NERFSRLLALNEWLITWCKEQKLLF  131 (171)
T ss_dssp              SEEEEEECCTTGGGCCHH-HHHHHHHHHHHHHHHHHCTTSEEEEECCCCCSSSC--HHHHHHHHHHHHHHHHHHHHHHHTTCEE
T ss_pred              CEEEEEecCCCCccccch-HHHHHHHHHHHHHHHHhCCCCeEEEEeCCCcCCCCc--hhhHHHHHHHHHHHHHHHhCCCEE


Q ss_pred              EEChHHHhhhcccccHhhcCCCCcCCCCcHHHHHHHHHHHHHHHHHHHH
Q gi|45553317|re  415 IDIHSCLINERGVVRFDCFQASPRQVTGSKEPYLFWNKIGRQRVLQV  461 (468)
Q Consensus       415 IDl~~~~~~~~~~g~~~~~~~~~dDGvHPn~~~~Gy~~lA~~i~~~I~~~  461 (468)
                      +|++..+.+      ..+++..||+|||.  +||++||+.+.+.|.++
T Consensus       132 id~~~~~~~~-----~~~~~~~~~DgiHpn~-~~G~~~~a~~i~~~l~~i  171 (171)
T 4c1b_A        132 ANNWNLFWE-----RPRLFRPDGLHPSR--AGAELLSDNISRLLRTI  171 (171)
T ss_dssp              ECCHHHHTT-----CGGGBCTTSSSBCH--HHHHHHHHHHHHHHTTC
T ss_pred              EccHHHHhc-----CccccCCCCcCcCH--HHHHHHHHHHHHHhcC
```

No 27

>**cd01839** SGNH_arylesterase_like SGNH_hydrolase subfamily, similar to arylesterase (7-aminocephalosporanic acid-deacetylating enzyme) of A. tumefaciens. SGNH hydrolases are a diverse family of lipases and esterases. The tertiary fold of the enzyme is substantially different from that of the alpha/beta hydrolase family and unique among all known hydrolases; its active site closely resembles the Ser-His-Asp(Glu) triad found in other serine hydrolases.
Probab=99.91 E-value=3e-23 Score=200.54 Aligned_cols=181 Identities=12% Similarity=0.017 Sum_probs=0.0

```
Q ss_pred              EEEEEcchhccCCCCCCCccccccccchHHHHHHHHHHHhhcCCCceEEEeCCCcCCccH---------HHHHHHHHHH
Q gi|45553317|re  263 TSGTYNDSLLTINSDYDAYLLDFPLMGDDFMLYLARMELKCRFRRHERVLQSGLCVSGLTI---------NGARNRLKRV  333 (468)
Q Consensus       263 ~Iv~iGDSIT~G~~~~~~~~~~~~~~liGds~~~~La~~~l~~~~~~~~~~~V~N~G~~isG~ts---------~~~~~~rl~~~  333 (468)
                      +|+|||||||.|   +.....+..-.+++|+..|+. .+...+ .++.|+|.|  ++|.|+          .+...+++..
```

```
T Consensus         1 ~I~~~GDSiT~G---~~~~~~~~~~~~~~~w~~~L~~-~l~~~~-~~~~vin~G--~~G~tt~~~~~~~~~~~~~~l~~~   73 (208)
T cd01839           1 TILCFGDSNTWG---IIPDTGGRYPFEDRWPGVLEK-ALGANG-ENVRVIEDG--LPGRTTVLDDPFFPGRNGLTYLPQA   73 (208)
T ss_pred              CEEEEECCccCC---CCCCCCCCCcCCCcHHHHHH-HHhcCC-CceEEEecC--CCCccccCCCcccCccHHHHHHHH


Q ss_pred              Hh--cCCCCeEEEEeeeccCCCC--CCHHHHHHHHHHHHHHHHhCC-------CEEEEEcCCCC-----CccccchhhHHHH
Q gi|45553317|re  334 QL--PEGTQIIVNIGSVDIMRG--KPLVQIEHDFRLLIKEMHNMR-------LVPILTNLAPL-----GNYCHDKVLCDK  397 (468)
Q Consensus       334 l~--~~PDlVvI~lGtND~~~g--~s~e~f~~~l~~LI~~lr~~g-------a~VIL~t~~P~-----~~~~~~~~~~~~  397 (468)
                      +.  ..||+|+|++|+||+...  .+.+++.+++++||+.+++..       ++|+|++++|+     ..........+.
T Consensus        74 l~~~~~~d~vii~lGtND~~~~~~~~~~~~~~~~li~~i~~~~~~~~~~~~~~iil~~ppp~~~~~~~~~~~~~~~~~~~  153 (208)
T cd01839          74 LESHSPLDLVIIMLGTNDLKSYFNLSAAEIAQGLGALVDIIRTAPIEPGMPAPKILIVAPPPIRTPKGSLAGKFAGAEEK  153 (208)
T ss_pred              HHhCCCCCEEEEEeccccccccCCCCHHHHHHHHHHHHHHHHHhhhcccCCCCeEEEEcCCCcccccchhhhccHHHH


Q ss_pred              HHHHHHHHHHHHhC--CCEEEECHHHhhhcccccHhhcCCCCcCCCCcHHHHHHHHHHHHHHHH
Q gi|45553317|re  398 IYRFNKFIRSECC--HLKVIDIHSCLINERGVVRFDCFQASPRQVTGSKEPYLFWNKIGRQRVLQV  461 (468)
Q Consensus       398 i~~~N~~ir~~A~--gv~fIDl~~~~~~~~~g~~~~~~~~~~dDGvHPn~~~~Gy~~lA~~i~~~I~~~  461 (468)
                      ...++++++++++|+  ++.|||++..+..          +..||+|||+  +||++||+.+.+.|.++
T Consensus       154 ~~~~~~~~~~~~a~~~~~~~id~~~~~~~-------------~~DGvHp~~--~G~~~~a~~l~~~i~~~  208 (208)
T cd01839         154 SKGLADAYRALAEELGCHFFDAGSVGST---------SPVDGVHLDA--DQHAALGQALASVIRAL  208 (208)
T ss_pred              HHHHHHHHHHHHHHcCCcEechhhhcCC---------CCCCCCCCCH--HHHHHHHHHHHHHHHhC
```

No 28

>**cd01838** Isoamyl_acetate_hydrolase_like Isoamyl-acetate hydrolyzing esterase-like proteins. SGNH_hydrolase subfamily similar to the Saccharomyces cerevisiae IAH1. IAH1 may be the major esterase that hydrolyses isoamyl acetate in sake mash. The SGNH-family of hydrolases is a diverse family of lipases and esterases. The tertiary fold of the enzyme is substantially different from that of the alpha/beta hydrolase family and unique among all known hydrolases; its active site closely resembles the Ser-His-Asp(Glu) triad found in other serine hydrolases
Probab=99.91 E-value=3e-23 Score=196.72 Aligned_cols=174 Identities=15% Similarity=0.200 Sum_probs=0.0

```
Q ss_pred              EEEEEcchhccCCCCCC--CcccccccccchHHHHHHHHHHHHhhcCCCceEEEeCCCcCCccHHHHHHHHHHHHHhcC---
Q gi|45553317|re  263 TSGTYNDSLLTINSDYD--AYLLDFPLMGDDFMLYLARMELKCRFRRHERVLQSGLCVSGLTINGARNRLKRVQLPE---  337 (468)
Q Consensus       263 ~Iv~iGDSIT~G~~~~~--~~~~~~~~~liGds~~~~La~~~l~~~~~~~~~~~~V~N~G~~isG~ts~~~~~rl~~~l~~~---  337 (468)
                      +|+++|||+|.|.....   .+..                    .+...+...+.|.|.|  ++|.++.++..+++..+...
T Consensus         1 ri~~lGDSit~g~~~~~~~~~~~~~~~----------------~~l~~~~~~~~~~~~v~n~g--~~G~~~~~~~~~~~~~~~~~~~~~~   62 (199)
T cd01838           1 KIVLFGDSITQFSFDQGEFGFGA----------------ALADVYSRKLDVINRG--FSGYNTRWALKVLPKIFLEEKLA   62 (199)
T ss_pred              CEEEEecCcccccCCCCCcHHH----------------HHHHHhcchhcEEecC--CCcccHHHHHHHHHHHHHhhhccC


Q ss_pred              -CCeEEEEeeeccCCCCCC-----HHHHHHHHHHHHHHHHHh--CCCEEEEEcCCCCCccc----------chhhHHHHHH
Q gi|45553317|re  338 -GTQIIVNIGSVDIMRGKP-----LVQIEHDFRLLIKEMHN--MRLVPILTNLAPLGNYC----------HDKVLCDKIY  399 (468)
Q Consensus       338 -PDlVvI~lGtND~~~g~s-----~e~f~~~l~~LI~~lr~~~~~ga~VIL~t~~P~~~~~~~~~----------~~~~~~~~~i~  399 (468)
                       ||+|||++|+||+|+.....     .+.|.++++++++.+++   .+++|||++++|+.....          .....+..+.
T Consensus        63 ~~d~vvi~~G~ND~~~~~~~~~~~~~~~~~~~~l~~lv~~~~~~~~~~~~~~vvl~~~~~~~~~~~~~~~~~~~~~~~~~~  142 (199)
T cd01838          63 QPDLVTIFFGANDAALPGQPQHVPLDEYKENLRKIVSHLKSLSPKTKVILITPPPVDEEAWEKSLEDGGSQPGRTNELLK  142 (199)
T ss_pred              CCCEEEEEeEcCcCCCCCCCCCcCCCHHHHHHHHHHHHHHHHHHhCCCCCeEEEECCCCCcCccccccccccccccHHHHH


Q ss_pred              HHHHHHHHHHhC--CCEEEECHHHhhhcccccHhhcCCCCcCCCCcHHHHHHHHHHHH
Q gi|45553317|re  400 RFNKFIRSECC--HLKVIDIHSCLINERGVVRFDCFQASPRQVTGSKEPYLFWNKIGRQRV  458 (468)
Q Consensus       400 ~~N~~ir~~A~--gv~fIDl~~~~~~~~~g~~~~~~~~~~dDGvHPn~~~~Gy~~lA~~i~~~I  458 (468)
                      .+|+.++++|+  +++|+|++..+..+..  ..+.+.+..||+|||.  +||+.||+.|+.+|+.+|
T Consensus       143 ~~~~~~~~~a~~~~~~v~~~iD~~~~~~~~~~~~~~~~~~DgiHp~~~~~G~~~~a~~i~~~i  199 (199)
T cd01838         143 QYAEACVEVAEELGVPVIDLWTAMQEEAG--WLESLLTDGLHFSS--KGYELLFEEIVKVI  199 (199)
T ss_pred              HHHHHHHHHHHHhCCCcEEehHHHhhccc--chhcCCCCCCCh--hHHHHHHHHHHHhhC
```

No 29

>**cd04506** SGNH_hydrolase_YpmR_like Members of the SGNH-hydrolase superfamily, a diverse family of lipases and esterases. The tertiary fold of the enzyme is substantially different from that of the alpha/beta hydrolase family and unique among all known hydrolases; its active site closely resembles the Ser-His-Asp(Glu) triad from other serine hydrolases, but may lack the carboxlic acid. This subfamily contains sequences similar to Bacillus YpmR.
Probab=99.91 E-value=1.8e-23 Score=200.56 Aligned_cols=179 Identities=15% Similarity=0.125 Sum_probs=0.0

```
Q ss_pred              EEEEEcchhccCCCCCCCcccccccccchHHHHHHHHHHHHhhcCCCceEEEeCCCcCCccHHHHHHHHHHHHHh----cCC
Q gi|45553317|re  263 TSGTYNDSLLTINSDYDAYLLDFPLMGDDFMLYLARMELKCRFRRHERVLQSGLCVSGLTINGARNRLKRVQL----PEG  338 (468)
Q Consensus       263 ~Iv~iGDSIT~G~~~~~~~~~~~~~~~liGds~~~~La~~~l~~~~~~~~~~~~V~N~G~~isG~ts~~~~~rl~~~l~----~~P  338 (468)
                      +|+++|||||.|.+..           .+++++..+++...+.....+.+.|  ++|.+.+++.+++.....    .+|
T Consensus         1 ~iv~~GDSit~G~g~~----------~~~~~~~~~~~~~~~~~~~~N~g~~i~G~t~~~~~~~~r~~~~~~~~~~~~~~  69 (204)
T cd04506           1 KIVALGDSLTEGVGDE---------TGKGGYVGRLDKLIETKTVKKVTVQNFG--VSGDRSDQLLKRLKTKKVQKELKKA  69 (204)
T ss_pred              CEEEEecccccCCCCC---------CCCCCHHHHHHHHHHhcCCCceEEEEee--cCCCCcCHHHHHHHHHccCCCcccCC


Q ss_pred              CeEEEEeeeccCCCC--------------CCHHHHHHHHHHHHHHHHHhC--CCEEEEEcC--CCCCccccchhhHHHHHHH
Q gi|45553317|re  339 TQIIVNIGSVDIMRG--------------KPLVQIEHDFRLLIKEMHNM--RLVPILTNL--APLGNYCHDKVLCDKIYR  400 (468)
Q Consensus       339 DlVvI~lGtND~~~g--------------~s~e~f~~~l~~LI~~lr~~~~~ga~VIL~t~~~~P~~~~~~~~~~~~~~~~i~~  400 (468)
                      |+|+|++|+||+|+.+             .+.+.+.|..+|+++|+.++++.|+++++.|+++++....+.....+.+.+
T Consensus        70 d~v~i~~G~ND~~~~~~~~~~~~~~~~~~~l~~~i~~~r~~~p~~~I~v~~~~~~~~~~~~~~~~~~p~~~~~~~~~~~~~  149 (204)
T cd04506          70 DVITITIGGNDLMQVLEKNFLSLDVEDFKKAEETYQNNLKKIFKEIRKLNPDAPIFLVGLYNPFYVYFPNITEINDIVND  149 (204)
T ss_pred              CEEEEEecchhhHHHHHHhccccCHHHHHHHHHHHHHHHHHHHHHHHCCCCeEEEEeCCCccccccchHHHHHHHH


Q ss_pred              HHHHHHHhC--C-CEEEECHHHhhhcccccHhhcCCCCcCCCCcHHHHHHHHHHHH
Q gi|45553317|re  401 FNKFIRSECC--H-LKVIDIHSCLINERGVVRFDCFQASPRQVTGSKEPYLFWNKIGRQR  457 (468)
Q Consensus       401 ~N~~ir~~A~--g-v~fIDl~~~~~~~~~g~~~~~~~~~~dDGvHPn~~~~Gy~~lA~~i~~~  457 (468)
                      +|+++++|+  + +.|||+++.|.+.   ...++.||+|||+  +||+++||+++++.
T Consensus       150 ~n~~~~~~a~~~~~~vd~~~~~~~~~~~~~~~~~~~~~~~DglHPn~~~Gy~~~A~~l~~~  204 (204)
T cd04506         150 WNEASQKLASQYKNAYFVPIFDLFSDGQ---NKYLLTSDHFHPND--KGYQLIADRVFKA  204 (204)
T ss_pred              HHHHHHHHHhCCCCeEEEehHHHHhhHHHhcCCCC---CcccccccCCCCCCH--HHHHHHHHHHHHhC
```

No 30

>**1ivn_A** Thioesterase I; hydrolase, protease; 1.90A {Escherichia coli} SCOP: **c.23.10.5** PDB: **1u8u _A*** **1j00 _A*** **1jrl _A** **1v2g _A***
Probab=99.91 E-value=3.7e-23 Score=195.39 Aligned_cols=179 Identities=15% Similarity=0.140 Sum_probs=0.0

```
Q ss_pred             EEEEEcchhccCCCCCCCcccccccccchHHHHHHHHHHHhhcCCCceEEEeCCCcCCccHHHHHHHHHHHHhc-CCCeE
Q gi|45553317|re  263 TSGTYNDSLLTINSDYDAYLLDFPLMGDDFMLYLARMELKCRFRRHERVLQSGLCVSGLTINGARNRLKRVQLP-EGTQI  341 (468)
Q Consensus       263 ~Iv~iGDSIT~G~~~~~~~~~~~~~~liGds~~~~La~~~l~~~~~~~~~V~N~G~~isG~ts~~~~~rl~~~l~~-~PDlV  341 (468)
                      +|+|+|||||.|....          .+.+|...++.     .+...+.+.|.|  ++|.++.++..+++..+.. +||+|
T Consensus         3 ~i~~~GDSit~g~~~~---------~~~~~~~~l~~-----~l~~~~~v~n~g--~~G~~~~~~~~~~~~~~~~~~~pd~v   66 (190)
T 1ivn_A           3 TLLILGDSLSAGYRMS---------ASAAWPALLND-----KWQSKTSVVNAS--ISGDTSQQGLARLPALLKQHQPRWV   66 (190)
T ss_dssp            EEEEEECHHHHCSSSC---------GGGSHHHHHHH-----HC-CCEEEEECC--CTTCCHHHHHHHHHHHHHHHCCSEE
T ss_pred            eEEEEecccccccCCC---------CCCcHHHHHHH-----HhccCCeEEecC--CCCccHHHHHHHHHHHHHhcCCCEE


Q ss_pred             EEEeeeccCCCCCCHHHHHHHHHHHHHHHHHHhCCCEEEEEcCCCCCccccchhhHHHHHHHHHHHHHHHhC--CCEEEEChH
Q gi|45553317|re  342 IVNIGSVDIMRGKPLVQIEHDFRLLIKEMHNMRLVPILTNLAPLGNYCHDKVLCDKIYRFNKFIRSECC--HLKVIDIHS  419 (468)
Q Consensus       342 vI~lGtND~~~g~s~e~f~~~l~~LI~~lr~~ga~VIL~t~~P~~~~~~~~~~~~~~~i~~~N~~ir~~A~~-gv~fIDl~~  419 (468)
                      +|++|+||+..+.+.++|.++++++|+.+++.+.+++|+++.....+.      ......+|++++++|+  ++.|||++.
T Consensus        67 vi~~G~ND~~~~~~~~~~~~~l~~li~~~~~~~~~~~il~~~~~p~~~~~-----~~~~~~~~~~~~a~~~~~~~vd~~~  141 (190)
T 1ivn_A          67 LVELGGNDGLRGFQPQQTEQTLRQILQDVKAANAEPLLMQIRLPANYG-----RRYNEAFSAIYPKLAKEFDVPLLPFFM  141 (190)
T ss_dssp            EEECCTTTTSSSCCHHHHHHHHHHHHHHHHHHHHTTCEEEEECCCCCGGGC-----HHHHHHHHHHHHHHHHHTTCCEECCTH
T ss_pred            EEEecCcccccccCCHHHHHHHHHHHHHHHHHHHHhcCCcEEEEeccCCcchh-----HHHHHHHHHHHHHHHHHHhCCcEEccHH


Q ss_pred             HHhhhcccccHhhcCCCCcCCCCcHHHHHHHHHHHHHHHHHHHHHHHHhcC
Q gi|45553317|re  420 CLINERGVVRFDCFQASPRQVTGSKEPYLFWNKIGRQRVLQVIETSLEY  468 (468)
Q Consensus       420 ~~~~~~g~~~~~~~~~~~dDGvHPn~~~~Gy~~lA~~i~~~~I~~~L~~~~a~  468 (468)
                      ..     .....+++..||+|||.  +||++||+.+.+.|.+++.+...+
T Consensus       142 ~~----~~~~~~~~~~~~DgiHpn~--~G~~~~~a~~i~~~l~~~~~~~~~  184 (190)
T 1ivn_A         142 EE----VYLKPQWMQDDGIHPNR--DAQPFIADWMAKQLQPLVNHDSLE  184 (190)
T ss_dssp            HH----HHTCGGGBCTTSSSBCG--GGHHHHHHHHHHHHHTTTTC-----
T ss_pred            hh----cccCccccCCCCCcCH--HHHHHHHHHHHHHHHHHHhhcccchhh
```

No 31

>4tx1_A Esterase; SGNH-hydrolase, SGNH-family, hydrolase; 1.75A {Sinorhizobium meliloti 1021}
Probab=99.91 E-value=1.5e-23 Score=202.41 Aligned_cols=187 Identities=15% Similarity=0.044 Sum_probs=0.0

```
Q ss_pred             CCCCccEEEEEcchhccCCCCCCCccccccccccchHHHHHHHHHHHhhcCCCceEEEeCCCcCCccHH----------HH
Q gi|45553317|re  257 PTPTILTSGTYNDSLLTINSDYDAYLLDFPLMGDDFMLYLARMELKCRFRRHERVLQSGLCVSGLTIN----------GA  326 (468)
Q Consensus       257 ~~P~~~~Iv~iGDSIT~G~~~~~~~~~~~~~~~liGds~~~~La~~~l~~~~~~~~~V~N~G~~isG~ts~-----------~~  326 (468)
                      +.|...+|+|+|||||.|   +.....+-.-.+.+|...|+..+     ...+.++|.|  ++|.++.             +.
T Consensus         9 ~~~~~~~~i~~~GDSit~G---~~~~~~~~~~~~~~~~~~~~~~l~~~l----------v~n~g--~~G~t~~~~~~~~~~~~~~~~~~  78 (225)
T 4tx1_A           9 HHGSMKTVLCYGDSLTWG---YDATGSGRHALEDRWPSVLQKAL-----GSDAHVIAEG--LNGRTTAYDDHLADCDRNG   78 (225)
T ss_dssp            ----CEEEEEEESHHHHT---EETTTTEECCGGGSHHHHHHHHH-----GGGEEEEEEE--CTTCBSSCCCCSSSSCCBH
T ss_pred            ccCccceEEEEECccccc---cCCCCCcccccccchHHHHHHHh-----CCCcceeecc--cCCCcccCCCcccccccccch


Q ss_pred             HHHHHHHH-hcCC-CeEEEEeeeccCCCCC--CHHHHHHHHHHHHHHHHHHhC---------CCEEEEEcCCCCCcc------
Q gi|45553317|re  327 RNRLKRVQ-LPEG-TQIIVNIGSVDIMRGK--PLVQIEHDFRLLIKEMHNM---------RLVPILTNLAPLGNY------  388 (468)
Q Consensus       327 ~~rl~~~l-~~~P-DlVvI~lGtND~~~g~--s~e~f~~~l~~LI~~lr~---------ga~VIL~t~~P~~~~------  388 (468)
                      ..++...+ ..+|  |+|+|++|+||+..+.  +.++|.++++++|+.+++.          +++|+|++++|+...
T Consensus        79 ~~~~~~~~l~~~~p~d~vvi~~G~ND~~~~~~~~~~~~~~l~~li~~~l~~~~~~~~~~~P~~~~ivl~~~~~~~~~~~~~~~  158 (225)
T 4tx1_A          79 ARVLPTVLHTHAPLDLIVFMLGSNDMKPIIHGTAFGAVKGIERLVNLVRRHDWPTETEEGPEILIVSPPPLCETANSAFA  158 (225)
T ss_dssp            HHHHHHHHHHHCSCSEEEEECCTGGGSTTTTCCHHHHHHHHHHHHHHHHHHHHTCCCSSCCCSCCEEEEEECCCCCCCSCHHHH
T ss_pred            HHHHHHHHhhCCCCcEEEEEeCCCcccccccCCCHHHHHHHHHHHHHHHHHHHhccccccCCCCCcEEEEeCCCcCcCCCcchh


Q ss_pred             cchhhHHHHHHHHHHHHHHHhC--CCEEEEChHHHhhhcccccHhhcCCCCcCCCCcHHHHHHHHHHHHHHHHHHHHHHH
Q gi|45553317|re  389 CHDKVLCDKIYRFNKFIRSECC--HLKVIDIHSCLINERGVVRFDCFQASPRQVTGSKEPYLFWNKIGRQRVLQVIET  464 (468)
Q Consensus       389 ~~~~~~~~~~~i~~~N~~ir~~A~~-gv~fIDl~~~~~~~~~~~~g~~~~~~~~~~~dDGvHPn~~~~Gy~~lA~~i~~~~I~~~L~~  464 (468)
                      .......+.+..+|++++++|+  ++.|||++..+          ..+..||+|||.  +||++||+.+.+.|.++|++
T Consensus       159 ~~~~~~~~~~~~~~~~n~~~~~~~~a~~~~v~~vD~~~~----------------------DgvHpn~--~G~~~iA~~l~~~l~~~l~~  225 (225)
T 4tx1_A         159 AMFAGGVEQSAMLAPLYRDLADELDCGFFDGGSVA---------RTTPIDGVHLDA--ENTRAVGRGLEPVVRMMLGL  225 (225)
T ss_dssp            HHTTTHHHHHTTHHHHHHHHHHHHTCEEEEGGGTC---------CCCTTTSSCCCH--HHHHHHHHHHHHHHHHHHTC
T ss_pred            hhccchHHHHHHHHHHHHHHHHHHHcCCeEEehhHhc---------ccccCCCcCCH--HHHHHHHHHHHHHHHHHHhcC
```

No 32

>2q0q_A ARYL esterase; SGNH hydrolase, oligomeric enzyme, acyl transfer, ARYL ester hydrolase; 1.50A {Mycobacterium smegmatis} PDB: 2q0s _A*
Probab=99.91 E-value=2.4e-23 Score=199.41 Aligned_cols=181 Identities=13% Similarity=0.070 Sum_probs=0.0

```
Q ss_pred             ccEEEEEcchhccCCCCCCC----ccccccccccchHHHHHHHHHHHhhcCCCceEEEeCCCcCCccHH---------HHH
Q gi|45553317|re  261 ILTSGTYNDSLLTINSDYDA----YLLDFPLMGDDFMLYLARMELKCRFRRHERVLQSGLCVSGLTIN---------GAR  327 (468)
Q Consensus       261 ~~~Iv~iGDSIT~G~~~~~~~~~~~~~~~~liGds~~~~La~~~l~~~~~~~~~V~N~G~~isG~ts~------------~~~  327 (468)
                      +.+|+|+|||||.|    +.+     ...+..-.+.+|...|+..+     ...+.++|.|  ++|.++.           +..
T Consensus         2 ~~~i~~~GDSit~G~~~~~~~~~~~~~~~~~~~~~~~~l~~~l-----------v~n~g--~~G~~~~~~~~~~~~~~~~~~~  71 (216)
T 2q0q_A           2 AKRILCFGDSLTWG---WVPVEDGAPTERFAPDVRWTGVLAQQL-----GADFEVIEEG--LSARTTNIDDPTDPRLNGA   71 (216)
T ss_dssp            CEEEEEEESHHHHT---BCCCTTCCCBCBCCTTTSHHHHHHHHH-----CTTEEEEEEE--CTTCBSSCCBTTBTTCBHH
T ss_pred            CceEEEEecCcccc---cCCcccccccccCCccccHHHHHHHHh-----CCCCCeeecc--cCCccccccCCCccccccccchH


Q ss_pred             HHHHHHHhcC-C-CeEEEEeeeccCCCC--CCCHHHHHHHHHHHHHHHHHHhCC---------CEEEEEcCCCCCccccchhhH
Q gi|45553317|re  328 NRLKRVQLPE-G-TQIIVNIGSVDIMR--GKPLVQIEHDFRLLIKEMHNMR---------LVPILTNLAPLGNYCHDKVL  394 (468)
Q Consensus       328 ~rl~~~l~~~-P-DlVvI~lGtND~~~g~s~e~f~~~l~~LI~~lr~~g---------a~VIL~t~~P~~~~~~~~~~~~  394 (468)
                      .+++..+... | |+|+|++|+||+..+  +.+.++|.++++++|+.+++.+         ++|+++++|+.......-.
T Consensus        72 ~~~~~~~l~~~~p~d~vvi~~G~ND~~~~~~~~~~~~~~~~l~~li~~~~~~~~~~~~~~~p~~~~iil~~~~~~~~~~~~~~~~  151 (216)
T 2q0q_A          72 SYLPSCLATHLPLDLVIIMLGTNDTKAYFRRTPLDIALGMSVLVTQVLTSAGGVGTTYPAPKVLVVSPPPLAPMPHPWFQ  151 (216)
T ss_dssp            HHHHHHHHHHCSCSEEEEECCTGGGSGGGCCCHHHHHHHHHHHHHHHHHTCTTTTBCCCCCEEEEEECCCCCCCCSHHHH
T ss_pred            HHHHHHHhhCCCCcEEEEEeCCCcccccccCCCHHHHHHHHHHHHHHHHHHhcccccccCCCCCCeEEEEeCCCcCcccCccch


Q ss_pred             ------HHHHHHHHHHHHHHHhC--CCEEEEChHHHhhhcccccHhhcCCCCcCCCCcHHHHHHHHHHHHHHHHHHHHHH
Q gi|45553317|re  395 ------CDKIYRFNKFIRSECC--HLKVIDIHSCLINERGVVRFDCFQASPRQVTGSKEPYLFWNKIGRQRVLQVI  462 (468)
Q Consensus       395 ---------i~~~N~~ir~~A~~-gv~fIDl~~~~~~~~~~~~g~~~~~~~~~~~dDGvHPn~~~~Gy~~lA~~i~~~~I~~~L  462 (468)
                            .+.+..+|++++++|+  ++.|||++..           ..+..||+|||.  +||++||+.+.+.|.++|
T Consensus       152 ~~~~~~~~~~~~~~~~n~~~~~~~~a~~~~v~~vD~~~~----------------------DgvHpn~--~G~~~~a~~i~~~l~~~L  216 (216)
T 2q0q_A         152 LIFEGGEQKTTELARVYSALASFMKVPFFDAGSVI---------STDGVDGIHFTE--ANNRDLGVALAEQVRSLL  216 (216)
T ss_dssp            HHTTTHHHHHTTHHHHHHHHHHHHTCCEEEGGGTC---------CCCSTTSSSCCH--HHHHHHHHHHHHHHHHHC
T ss_pred            hccccHHHHHHHHHHHHHHHHHHHcCCcEEechhcc---------ccccCCccCcCH--HHHHHHHHHHHHHHHHhC
```

No 33

>**cd04502** SGNH_hydrolase_like_7 Members of the SGNH-hydrolase superfamily, a diverse family of lipases and esterases. The tertiary fold of the enzyme is substantially different from that of the alpha/beta hydrolase family and unique among all known hydrolases; its active site closely resembles the Ser-His-Asp(Glu) triad from other serine hydrolases, but may lack the carboxlic acid.
Probab=99.91 E-value=8.4e-23 Score=191.39 Aligned_cols=164 Identities=21% Similarity=0.331 Sum_probs=0.0

```
Q ss_pred              EEEEcchhccCCCCCCCccccccccchHHHHHHHHHHHHhhcCCCceEEEEeCCCcCCccHHHHHHHHHHHHHhc-CCCeEEE
Q gi|45553317|re  264 SGTYNDSLLTINSDYDAYLLDFPLMGDDFMLYLARMELKCRFRRHERVLQSGLCVSGLTINGARNRLKRVQLP-EGTQII  342 (468)
Q Consensus       264 Iv~iGDSIT~G~~~~~~~~~~~~~~liGds~~~~La~~~l~~~~~~~~~~V~N~G~~isG~ts~~~~~~rl~~~l~~-~PDlVv  342 (468)
                     |+|+|||++                       ..|..-.....++.++|.|  ++|.++.+++.+++..+.. +||+|+
T Consensus         2 i~~~G~s~~-----------------------~~w~~~~~~~~~~~~~viN~G--i~G~~~~~~~~~~~r~~~~~~~~~p~~vi   55 (171)
T cd04502          2 ILFYGSSSI-----------------------RLWDTLADDLAPLPVVNRG--FGGSTLADCLHYFDRLVLPYQPRRVV   55 (171)
T ss_pred              EEEEcCchh-----------------------hchhhHHHhcCCCCeEecC--cChhhHHHHHHHHHhHhhcCCCCEEE
```

```
Q ss_pred              EEeeeccCCCCCCHHHHHHHHHHHHHHHHHhC--CCEEEEEcCCCCCccccchhhHHHHHHHHHHHHHHHHhC---CCEEEEC
Q gi|45553317|re  343 VNIGSVDIMRGKPLVQIEHDFRLLIKEMHNM--RLVPILTNLAPLGNYCHDKVLCDKIYRFNKFIRSECC---HLKVIDI  417 (468)
Q Consensus       343 I~lGtND~~~g~s~e~f~~~l~~LI~~lr~~--ga~VIL~t~~P~~~~~~~~~~~~~~~~~i~~~N~~ir~~A~---gv~fIDl  417 (468)
                     |++|+||+..+.+++++.++++++++.+++.  +++|++++++|   ..........+.++|+.++++|+   ++.|+|+
T Consensus        56 i~~G~ND~~~~~~~~~~~~~~~~~~li~~~~~~~~~~~~vil~~~~p---~~~~~~~~~~~~~~~~n~~l~~~a~~~~~~~~~~vD~  132 (171)
T cd04502         56 LYAGDNDLASGRTPEEVLRDFRELVNRIRAKLPDTPIAIISIKP---SPARWALRPKIRRFNALLKELAETRPNLTYIDV  132 (171)
T ss_pred              EEEeecccCCCCCHHHHHHHHHHHHHHHHHHCCCCcEEEEeecC---CcchhhhhHHHHHHHHHHHHHHHHhhCCCeEEEEC
```

```
Q ss_pred              hHHHhhhcccccHhhcCCCCcCCCCcHHHHHHHHHHHHHHH
Q gi|45553317|re  418 HSCLINERGVVRFDCFQASPRQVTGSKEPYLFWNKIGRQRV  458 (468)
Q Consensus       418 ~~~~~~~~~g~~~~~~~~~dDGvHPn~~~~Gy~~lA~~i~~~I  458 (468)
                     +..|.+..+....+++..||+|||.  +||++||+.+.++|
T Consensus       133 ~~~~~~~~~~~~~~~~~~~~~~~~DGvHpn~--~Gy~~~a~~l~~~~  171 (171)
T cd04502        133 ASPMLDADGKPRAELFQEDGLHLND--AGYALWRKVIKPAL  171 (171)
T ss_pred              hHHHhCCCCCCChhhcCCCCCcCH--HHHHHHHHHHhhC
```

No 34

>**cd01825** SGNH_hydrolase_peri1 SGNH_peri1; putative periplasmic member of the SGNH-family of hydrolases, a diverse family of lipases and esterases. The tertiary fold of the enzyme is substantially different from that of the alpha/beta hydrolase family and unique among all known hydrolases; its active site closely resembles the Ser-His-Asp(Glu) triad found in other serine hydrolases.
Probab=99.91 E-value=4.1e-23 Score=195.41 Aligned_cols=173 Identities=13% Similarity=0.044 Sum_probs=0.0

```
Q ss_pred              EEEEEcchhccCCCCCCCccccccccchHHHHHHHHHHHHhhcCCCceEEEEeCCCcCCccHHHHHHHHHHHH-----hcC
Q gi|45553317|re  263 TSGTYNDSLLTINSDYDAYLLDFPLMGDDFMLYLARMELKCRFRRHERVLQSGLCVSGLTINGARNRLKRVQ-----LPE  337 (468)
Q Consensus       263 ~Iv~iGDSIT~G~~~~~~~~~~~~~~liGds~~~~La~~~l~~~~~~~~~~V~N~G~~isG~ts~~~~~~rl~~~l-----~~~  337 (468)
                     +|+|+|||||               -|..|+..|...+       ++.|.|.|  ++|.++.++.......+     ..+
T Consensus         1 ~iv~~GDSit---------------~g~~~~~l~~~l--------~v~N~g--~~G~t~~~~~~~~~~~~~~~~~~~~~~~~~  56 (189)
T cd01825          1 RIAQLGDSHI---------------AGDFFTDVLRGLL-------GVIYDNLG--VNGASASLLLKWDAEFLQAQLAALP   56 (189)
T ss_pred              CeEEEecccc---------------cccchHHHHhhc-------ceEEEecc--cCccchhHhhcCCHHHHHHHHhcC
```

```
Q ss_pred              CCeEEEEeeeccCCCCC-CHHHHHHHHHHHHHHHHHhC--CCEEEEEcCCCCCccc--chhhHHHHHHHHHHHHHHHHhC--
Q gi|45553317|re  338 GTQIIVNIGSVDIMRGK-PLVQIEHDFRLLIKEMHNM--RLVPILTNLAPLGNYC--HDKVLCDKIYRFNKFIRSECC--  410 (468)
Q Consensus       338 PDlVvI~lGtND~~~g~-s~e~f~~~l~~LI~~lr~~--ga~VIL~t~~P~~~~~~~~~~~~~~~~~i~~~N~~ir~~A~~--  410 (468)
                     ||+|+|++|+||+..+. +.++|.+++.++|+.+++.  +++|++++++|.....  .........+..+|++++++|+
T Consensus        57 pd~v~i~~G~ND~~~~~~~~~~~~l~~li~~ir~~~~~ii~~~~~~~~~~~~~~~~~~~~~~~~~~~~~~~~~~~~~a~~~~  136 (189)
T cd01825         57 PDLVILSYGTNEAFNKQLNASEYRQQLREFIKRLRQILPNASILLVGPPDSLQKTGAGRWRTPPGLDAVIAAQRRVAKEE  136 (189)
T ss_pred              CCEEEEEEccccCCCCCCHHHHHHHHHHHHHHHHHHHHCCCCeEEEEcCCcHhhcCCCCcccChhHHHHHHHHHHHHHHc
```

```
Q ss_pred              CCEEEECHHHHhhhcc---cccHhhcCCCCcCCCCcHHHHHHHHHHHHHHHHHH
Q gi|45553317|re  411 HLKVIDIHSCLINERG---VVRFDCFQASPRQVTGSKEPYLFWNKIGRQRVLQV  461 (468)
Q Consensus       411 gv~fIDl~~~~~~~~g---~~~~~~~~~dDGvHPn~~~~Gy~~lA~~i~~~I~~~~  461 (468)
                     ++.|||+++.+.+..+   ....+++..||+|||.  +||+.||+.+.+.|.+.
T Consensus       137 ~~~~id~~~~~~~~~~~~~~~~~~~~~~~~~DgiHp~~~~Gy~~la~~~~~~~~l~~~  188 (189)
T cd01825        137 GIAFWDLYAAMGGEGGIWQWAEPGLARKDYVHLTP--RGYERLANLLYEALLKA  188 (189)
T ss_pred              CCEEEEhHHhhCCCcchhhHhhccccccCCCCcccCc--HHHHHHHHHHHHHHhh
```

No 35

>**cd01832** SGNH_hydrolase_like_1 Members of the SGNH-hydrolase superfamily, a diverse family of lipases and esterases. The tertiary fold of the enzyme is substantially different from that of the alpha/beta hydrolase family and unique among all known hydrolases; its active site closely resembles the Ser-His-Asp(Glu) triad from other serine hydrolases, but may lack the carboxlic acid. Myxobacterial members of this subfamily have been reported to be involved in adventurous gliding motility.
Probab=99.91 E-value=1.4e-22 Score=190.40 Aligned_cols=180 Identities=9% Similarity=-0.036 Sum_probs=0.0

```
Q ss_pred              EEEEEcchhccCCCCCCCccccccccchHHHHHHHHHHHHhhcCCCceEEEEeCCCcCCccHHHHHHHHHH-HHHhcCCCeE
Q gi|45553317|re  263 TSGTYNDSLLTINSDYDAYLLDFPLMGDDFMLYLARMELKCRFRRHERVLQSGLCVSGLTINGARNRLKR-VQLPEGTQI  341 (468)
Q Consensus       263 ~Iv~iGDSIT~G~~~~~~~~~~~~~~liGds~~~~La~~~l~~~~~~~~~~V~N~G~~isG~ts~~~~~~rl~~-~l~~~PDlV  341 (468)
                     +|+|+|||||.|........      ...+|...++..+-.  ...++.+.|.|  ++|.++.++..+... .+..+||+|
T Consensus         1 ~i~~~GDSit~G~~~~~~~~-----~~~~~~~~~~~~~~~~~~~~~l~~~~~~~~~n~g~~~~G~~~~~~~~~~~~~~pd~v   71 (185)
T cd01832          1 RYVALGDSITEGVGDPVPDG-----GYRGWADRLAAALAA--ADPGIEYANLA--VRGRRTAQILAEQLPAALALRPDLV   71 (185)
T ss_pred              CeEEeecccccccCCCCCCC-----CcccHHHHHHHHhcc--cCCCceEEEecC--cCcccHHHHHHHhHHHHhcCCCEE
```

```
Q ss_pred              EEEeeeccCCC-CCCHHHHHHHHHHHHHHHHHhCCCEEEEEcCCCC-CccccchhhHHHHHHHHHHHHHHHHhC--CCEEEEC
Q gi|45553317|re  342 IVNIGSVDIMR-GKPLVQIEHDFRLLIKEMHNMRLVPILTNLAPL-GNYCHDKVLCDKIYRFNKFIRSECC--HLKVIDI  417 (468)
Q Consensus       342 vI~lGtND~~~-g~s~e~f~~~l~~LI~~lr~~ga~VIL~t~~P~-~~~~~~~~~~~~~~~i~~~N~~ir~~A~--gv~fIDl  417 (468)
                     ||++|+||...  ..+.++|.+++.++|+.++..+++|++++++|. ...+..........+|++++++|+  ++.++|+
T Consensus        72 i~~G~ND~~~~~~~~~~~~~~i~~i~~~~ii~v~~~~~~~~~~~~~~~~n~~~~~~~~v~~vd~  151 (185)
T cd01832         72 TLLAGGNDILRPGTDPDTYRADLEEAVRRLRAAGARVVVFTIPDPAVLEPFRRRVRARLAAYNAVIRAVAARYGAVHVDL  151 (185)
T ss_pred              EEeccccccCCCCCHHHHHHHHHHHHHHHHhcCCCeEEEeccCCCCcchhhHHHHHHHHHHHHHHHHHHHHcCcEEEec
```

```
Q ss_pred              hHHHhhhcccccHhhcCCCCcCCCCcHHHHHHHHHHHHHH
Q gi|45553317|re  418 HSCLINERGVVRFDCFQASPRQVTGSKEPYLFWNKIGRQR  457 (468)
Q Consensus       418 ~~~~~~~~~g~~~~~~~~~dDGvHPn~~~~Gy~~lA~~i~~~  457 (468)
```

```
                         +..+.    ....+++..||+|||.  +||+.||+.|+++
T Consensus        152 ~~~~~~---------~~~~~~DgiHpn~--~G~~~~a~~i~~a  185 (185)
T cd01832          152 WEHPE----FADPRLWASDRLHPSA--AGHARLAALVLAA  185 (185)
T ss_pred              ccccc----ccCccccCCCCCCCH--HHHHHHHHHHhhC
```

No 36

>**cd01827** sialate_O-acetylesterase_like1 sialate O-acetylesterase_like family of the SGNH hydrolases, a diverse family of lipases and esterases. The tertiary fold of the enzyme is substantially different from that of the alpha/beta hydrolase family and unique among all known hydrolases; its active site closely resembles the Ser-His-Asp(Glu) triad found in other serine hydrolases.
Probab=99.91 E-value=1.6e-22 Score=192.31 Aligned_cols=173 Identities=15% Similarity=0.066 Sum_probs=0.0

```
Q ss_pred              cEEEEEcchhccCCCCCCCcccccccccchHHHHHHHHHHHHhhcCCCCceEEEeCCCcCCccHHHH-------HHHHHHHH
Q gi|45553317|re  262 LTSGTYNDSLLTINSDYDAYLLDFPLMGDDFMLYLARMELKCRFRRHERVLQSGLCVSGLTINGA-------RNRLKRVQ  334 (468)
Q Consensus       262 ~~Iv~iGDSIT~G~~~~~~~~~~~~~~liGds~~~~La~~~l~~~~~~~~~~~~V~N~G~~isG~ts~~~~--------~~rl~~~l  334 (468)
                         +||+|+||||||+|....          .+.+|+..+++.+     ...+.|.|.|  ++|.++.+.       ..++..++
T Consensus          1 ~~I~~lGDSiT~G~~~~-----------------~~~~~l~~~l----------~~~v~N~G--~~G~~~~~~~~~~~~~~~~~~~~~~  64 (188)
T cd01827            1 IKVACVGNSITEGAGLR---------AYDSYPSPLAQML-----GDGYEVGNFG--KSARTVLNKGDHPYMNEERYKNAL   64 (188)
T ss_pred              CEEEEeccccCCCCCC---------CCCChHHHHHHHh-----cCCCEEEecC--cccccchhccCCcchhhHHHHHhh


Q ss_pred              hcCCCeEEEEeeeccCC--CCCCHHHHHHHHHHHHHHHHhC--CCEEEEEcCCCCCcccch-hhHHHHHHHHHHHHHHHh
Q gi|45553317|re  335 LPEGTQIIVNIGSVDIM--RGKPLVQIEHDFRLLIKEMHNM--RLVPILTNLAPLGNYCHD-KVLCDKIYRFNKFIRSEC  409 (468)
Q Consensus       335 ~~~PDlVvI~lGtND~~--~~g~s~e~f~~~~l~~LI~~lr~~--~ga~VIL~t~~P~~~~~~~~~~-~~~~~~~i~~~N~~ir~~A  409 (468)
                   ..+|||+|+|++|+|||+.  .....+.|.+++++||||+.+++.  +++|+|++++|.......  .........+|++++++|
T Consensus         65 ~~~pd~V~i~~G~ND~~~~~~~~~~~~~~~~~~~~~li~~l~~~~~~~~~Iil~tp~p~~~~~~~~~~~~~~~~~~~~~~~i~~~a  144 (188)
T cd01827           65 AFNPNIVIIKLGTNDAKPQNWKYKDDFKKDYETMIDSFQALPSKPKIYICYPIPAYYGDGGFINDNIIKKEIQPMIDKIA  144 (188)
T ss_pred              ccCCCEEEEEcCcCCCCccccccHHHHHHHHHHHHHHHhcCCCCeEEEEeCCccccCCCCcccHHHHHHHHHHHHH


Q ss_pred              C--CCEEEECHHHHhhcccccHhhcCCCCcCCCCcHHHHHHHHHHHHHH
Q gi|45553317|re  410 C--HLKVIDIHSCLINERGVVRFDCFQASPRQVTGSKEPYLFWNKIGRQRV  458 (468)
Q Consensus       410 ~--gv~fIDl~~~~~~~~~g~~~~~~~~~~~~~dDGvHPn~~~~Gy~~lA~~i~~~I  458 (468)
                         +  ++.|||++..+....       .+..||+|||.  +||++||+.++++|
T Consensus        145 ~~~~~~~~~iD~~~~~~~~~~~~~-------~~~~~~~Dg~HPn~~~~G~~~~~a~~i~~~i  187 (188)
T cd01827          145 KKLNLKLIDLHTPLKGKP------ELVPDWVHPNE--KGAYILAKVVYKAI  187 (188)
T ss_pred              HHcCCeEEECcccccCCCc------CccCCCCCCCCH--HHHHHHHHHHHHHh
```

No 37

>**SUPFAM template** c.23.10 SGNH hydrolase (**52266**) SCOP seed sequence: d1yzfa1.
Probab=99.90 E-value=6.9e-22 Score=174.04 Aligned_cols=187 Identities=20% Similarity=0.290 Sum_probs=0.0

```
Q ss_pred              ccEEEEEcchhccCCCCCCCcccccccccchHHHHHHHHHHHHhhcCCCCceEEEeCCCcCCccHHHHHHHHHHHHhcC-CC
Q gi|45553317|re  261 ILTSGTYNDSLLTINSDYDAYLLDFPLMGDDFMLYLARMELKCRFRRHERVLQSGLCVSGLTINGARNRLKRVQLPE-GT  339 (468)
Q Consensus       261 ~~~Iv~iGDSIT~G~~~~~~~~~~~~~~liGds~~~~La~~~l~~~~~~~~~~~~V~N~G~~isG~ts~~~~~~rl~~~l~~~~PD  339 (468)
                   .++|+++|||+|.|.....          .+..|...+.. .+...++.++.+.|.|  ++|.++.++..++...+...  ||
T Consensus          1 ~~~~~~~~~~~GDS~~~g~~~~~~~~---------~~~~~~~~~~~-~l~~~~~~~~~~~~~n~g--~~G~~~~~~~~~~~~~~~p~  69 (195)
T SUPFAM0051447     1 PLRILLLGDSITAGYGASG--------PGGGWPALLAE-LLAARFGRGVTVINLG--ISGDTTADLLARLDRLLLALKPD  69 (195)
T ss_pred              CccEEEeCCChhhcccccc--------ccccHHHHHHH-HHHHhcCCCceEEEec--cCcccHHHHHHHHHHHhhcCCC


Q ss_pred              eEEEEeeeccC--CCCCCHHHHHHHHHHHHHHHHhCCCEEEEEcCCCCCccccchhhHHHHHHHHHHHHHHHHHhC--CCEEE
Q gi|45553317|re  340 QIIVNIGSVDI--MRGKPLVQIEHDFRLLIKEMHNMRLVPILTNLAPLGNYCHDKVLCDKIYRFNKFIRSECC--HLKVI  415 (468)
Q Consensus       340 lVvI~lGtND~~~~~g~s~e~f~~~~l~~LI~~lr~~ga~VIL~t~~P~~~~~~~~~~~~~~~~~~~i~~~N~~ir~~A~~~gv~fI  415 (468)
                   +|+|++|+||.  ..+.+.+.+.++++.+++.+++.++++++++++++++.....  ...+....+|++++++|.  ++.++
T Consensus         70 ~v~~~~~G~ND~~~~~~~~~~~~~~~~~~~~~~~~~~~~~~~~~~~~~~~--~~~~~~~~~~~~~~~~~~~~~~~~~~~~~~~~~~~~~~~~~~~~~~~~~  147 (195)
T SUPFAM0051447    70 LVVILLGTNDLLLLRGVSLEEFAANLRALIDRLRAPGAKVVLVTPPPVGELPN--RLNERLAAYNAALRELAAELGVPFV  147 (195)
T ss_pred              EEEEEeecchhhhhcCCCHHHHHHHHHHHHHHHhccCCeEEEEecccchhcchh--hhHHHHHHHHHHHHHHHHHHHhcCCeEe


Q ss_pred              EChHHHhhcccccHhhcCCCCcCCCCcHHHHHHHHHHHHHHHHHHHHH
Q gi|45553317|re  416 DIHSCLINERGVVRFDCFQASPRQVTGSKEPYLFWNKIGRQRVLQVIET  464 (468)
Q Consensus       416 Dl~~~~~~~~~g~~~~~~~~~~~~~dDGvHPn~~~~Gy~~lA~~i~~~I~~~L~~  464 (468)
                   |++..+.+.+...  ..+++..||+|||.  .||+.+|+.+.+.|.+.+++
T Consensus        148 d~~~~~~~~~~~~~~~~~~~~~~~~D~~H~~~--~~G~~~~~~~~~~~~~l~~~  192 (195)
T SUPFAM0051447   148 DLYSALLDLAG--LPELLLPDGLHPNA--AGYRLLAEALAEALKKLLPE  192 (195)
T ss_pred              ehHHHHhhcc--chhhhccCcCCcCH--HHHHHHHHHHHHHHHHHhhcc
```

No 38

>**3dci_A** Arylesterase; SGNH_hydrolase SUBF structural genomics, PSI-2, protein structure initiative, M center for structural genomics, MCSG; HET: MSE; 2.00A {Agrobacterium tumefaciens str}
Probab=99.90 E-value=9.8e-23 Score=200.73 Aligned_cols=183 Identities=13% Similarity=0.044 Sum_probs=0.0

```
Q ss_pred              ccEEEEEcchhccCCCCCCCcccccccccchHHHHHHHHHHHHhhcCCCCceEEEeCCCcCCccH----------HHHHHHH
Q gi|45553317|re  261 ILTSGTYNDSLLTINSDYDAYLLDFPLMGDDFMLYLARMELKCRFRRHERVLQSGLCVSGLTI----------NGARNRL  330 (468)
Q Consensus       261 ~~~Iv~iGDSIT~G~~~~~~~~~~~~~~liGds~~~~La~~~l~~~~~~~~~~~~V~N~G~~isG~ts----------~~~~~rl  330 (468)
                   +.+|+|||||||.|   +.+.......-....|+..|+..+     ..++.|+|.|  ++|.|+            .+...++
T Consensus         23 mK~Il~~GDSiT~G~~~~~~~~g~r~~~~~~rwp~~L~~~l-----g~~~~~ViN~G--~~G~Tt~~~~~~~~~~~~~g~~~l   92 (232)
T 3dci_A           23 MKTVLAFGDSLTWG---ADPATGLRHPVEHRWPDVLEAEL-----AGKAKVHPEG--LGGRTTCYDDHAGPACRNGARAL   92 (232)
T ss_dssp              CEEEEEEESHHHHT---BCTTTCCBCCGGGSHHHHHHHHH-----TTSEEEEEEE--CTTCBSSCCCCSSSSCCBHHHHH
T ss_pred              CCEEEEEEcccccC---ccCCCCCcCCccccHHHHHHHc-----CCCCEEEECc--cccccccCCCChhhhhHHHHH


Q ss_pred              HHHHhcC-C-CeEEEEeeeccCCC--CCCHHHHHHHHHHHHHHHHhC-------CCEEEEEcCCCCCc----ccchhhHH
Q gi|45553317|re  331 KRVQLPE-G-TQIIVNIGSVDIMR--GKPLVQIEHDFRLLIKEMHNM-------RLVPILTNLAPLGN----YCHDKVLC  395 (468)
Q Consensus       331 ~~~l~~~P-DlVvI~lGtND~~~~~g~s~e~f~~~~l~~LI~~lr~~--------ga~VIL~t~~P~~~~~~~~~~~~~~~  395 (468)
                   +..+...  |  |+|||++|+||+..  +.+++++.++++.+|++.+++.        +++|++++++|...    ........
T Consensus         93 ~~~~~~p~D~ViI~lGtND~~~~~~~~~~~~~~~~~~l~~li~~i~~~~~~~~~~~~~~~ill~~~~p~~~~~~~~~~~~~~~~
T 3dci_A           93 EVALSCHMPLDLVIIMLGTNDIKPVHGGRAEAAVSGMRRLAQIVETFIYKPREAVPKLLIVAPPPCVAGPGGEPAGGRDI  172 (232)
T ss_dssp              HHHHHHCSCSEEEEECCTTTSGGGTSSHHHHHHHHHHHHHHHHHHHCCCSSTTCCCEEEEEECCCCCCCTTSSCGGGCCH
T ss_pred              HHHHhCCCCCEEEEccCccccCCCHHHHHHHHHHHHHHHhcccccCCCCeEEEeeccccCcccccchhH


Q ss_pred              HHHHHHHHHHHHHhC--CCEEEEChHHHhhcccccHhhcCCCCcCCCCcHHHHHHHHHHHHHHHH
```

```
Q gi|45553317|re  396 DKIYRFNKFIRSECC--HLKVIDIHSCLINERGVVRFDCFQASPRQVTGSKEPYLFWNKIGRQRVLQVIET  464 (468)
Q Consensus       396 ~~i~~~N~~ir~~A~--gv~fIDl~~~~~~~~g~~~~~~~~dDGvHPn~~~~Gy~~lA~~i~~~I~~~L~~  464 (468)
                      +...+++++++++|.  ++.|||+...+         +....||+|||+  +||++||+.|++.|+++|..
T Consensus       173 ~~~~~~~~~~~~vA~~~~v~~iD~~~~~-----------~~~~~DgiHpn~~~G~~~lA~~ia~~I~~iLg~  232 (232)
T 3dci_A          173 EQSMRLAPLYRKLAAELGHHFFDAGSVA---------SASPVDGVHLDA--SATAAIGRALAAPVRDILGS  232 (232)
T ss_dssp            HHHTTHHHHHHHHHHHHTCEEEEGGGTC---------CCCTTTSSSCCH--HHHHHHHHHHHHHHHHHC--
T ss_pred            HHHHHHHHHHHHHHHHHcCCeEEeCcccc---------CCCCCCCCCcCH--HHHHHHHHHHHHHHHHHHhCc
```

No 39

>**3rco_A** Tudor domain-containing protein 7; structural genomics, structural genomics consortium, SGC, HL DNA binding protein; 1.80A {Homo sapiens} PDB: **2lh9 _A**

Probab=99.90 E-value=1.4e-23 Score=174.95 Aligned_cols=84 Identities=27% Similarity=0.403 Sum_probs=0.0

```
Q ss_pred             ccchhhhhhHHHHHHHHHHHHeecCCCCCcCHHHHHHHHHHHhCCcCCC----cccHHHHHhcCCCeEEEE-eCCCCEEE
Q gi|45553317|re    5 ESNYISVREEYPDIDSEVRAILLSHAQNGITISSIKSEYRKLTGNPFPL----HDNVTDFLLTIPNVTAEC-SESGKRIF   79 (468)
Q Consensus         5 ~~~~~~~~~~~~~~~~~~irs~l~s~~k~g~~~~~l~~dy~~~~g~~~p~----~~~~~~~~l~~~~~~~~~~-~~~g~~~~   79 (468)
                      |..|+..+.|.++++++|||||+| +|+|+|+++|++|||+++|++|||     |+|++|||++||+|+|+..+ +.+|++++
T Consensus       1 ~~~~~~~~~~~~e~Vkk~iRslL~S~~k~gvtl~~L~~~DYr~l~G~~iP~r~lGy~sl~~fL~~sipdvv~~~~~~~~Gev~v   79 (89)
T 3rco_A          1 ENLYFQGMLEGDLVSKMLRAVLQS-HKNGVALPRLQGEYRSLTGDWIPFKQLGFPTLEAYLRSVPAVVRIETSRSGEITC   79 (89)
T ss_dssp            -------CCHHHHHHHHHHHHHHHT-CTTCEEHHHHHHHHHHHHHSSCCCTTTTTCSSHHHHHHTCTTTEEEEECTTSCEEE
T ss_pred             CcchhhhhccHHHHHHHHHHHHhee-CCCCCCHHHHHHHHHHHcCCcCChhhhCCcCHHHHHhcCcCeEEEEecCCCcEEE
```

```
Q ss_pred             EEecCcchHH
Q gi|45553317|re   80 NLKASLKNGH    89 (468)
Q Consensus        80 ~~~~~~~t~h    89 (468)
                      +++++++|+|
T Consensus       80 ~av~~~~taH    89 (89)
T 3rco_A         80 YAMACTETAR    89 (89)
T ss_dssp            EECC------
T ss_pred             EEEeCccccC
```

No 40

>**2hsj_A** Putative platelet activating factor; structr genomics, structural genomics, PSI-2; HET: MSE; 1.50A {Streptococcus pneumoniae} SCOP: **c.23.10.3**

Probab=99.90 E-value=8.9e-23 Score=195.52 Aligned_cols=172 Identities=14% Similarity=0.187 Sum_probs=0.0

```
Q ss_pred             CCCccEEEEEcchhccCCCCCCCcccccccccchHHHHHHHHHHHHhhcCCCCceEEEeCCCcCCccHHHHHHHHHHHHhcC
Q gi|45553317|re  258 TPTILTSGTYNDSLLTINSDYDAYLLDFPLMGDDFMLYLARMELKCRFRRHERVLQSGLCVSGLTINGARNRLKRVQLPE  337 (468)
Q Consensus       258 ~P~~~~Iv~iGDSIT~G~~~~~~~~~~~~~~~~liGds~~~~La~~~l~~~~~~~~~~~V~N~G~~isG~ts~~~~~~rl~~~l~~~   337 (468)
                      .+...+|+|+|||||.|..                        +...+..++.+.|.|  ++|.++.++..+++..+...
T Consensus        31 ~~~~~~i~~~GDSit~g~~------------------------~~~~~~~~~~~~~~n~g~~~G~~~~~~~~~~~~~~~~~~~   84 (214)
T 2hsj_A         31 SVVEPNILFIGDSIVEYYP------------------------LQELFGTSKTIVNRG--IRGYQTGLLLENLDAHLYGG   84 (214)
T ss_dssp            CSSCCSEEEEESHHHHTCC------------------------HHHHHCSSSCEEEEE--CTTCCHHHHHHTGGGGCCCS
T ss_pred             cCcccCcEEEEeccccccCh------------------------HHHHhCCCCcEEEeC--CccccHHHHHHHHHHHHHhhc
```

```
Q ss_pred             -CCeEEEEeeeccCCCCCCHHHHHHHHHHHHHHHHhC--CCEEEEEcCCCCCccccchhhH-----HHHHHHHHHHHHHHh
Q gi|45553317|re  338 -GTQIIVNIGSVDIMRGKPLVQIEHDFRLLIKEMHNM--RLVPILTNLAPLGNYCHDKVL-----CDKIYRFNKFIRSEC  409 (468)
Q Consensus       338 -PDlVvI~lGtND~~~g~s~e~f~~~l~~LI~~lr~~~ga~VIL~t~~P~~~~~~~~~~~~~-----~~~i~~~N~~ir~~A  409 (468)
                       ||+|+|++|+||+..+.+.++|.++++++|+.+++.  +++|++++++|.........     .+.+..+|++++++|
T Consensus        85 ~pd~vvi~~G~ND~~~~~~~~~~~~~~~~~~~~~~~~i~~~~~~~p~~~ii~~~~~~p~~~~~~~~~~~~~~~~~~~~~~n~~l~~~a  164 (214)
T 2hsj_A         85 AVDKIFLLIGTNDIGKDVPVNEALNNLEAIIQSVARDYPLTEIKLLSILPVNEREEYQQAVYIRSNEKIQNWNQAYQELA  164 (214)
T ss_dssp            CCCEEEEECCHHHHHTTCCHHHHHHHHHHHHHHHHHHHCTTCEEEEECCCCCCSGGGHHHHTTCCHHHHHHHHHHHHHHHH
T ss_pred             CCCEEEEEeecccCCCCCCHHHHHHHHHHHHHHHHHHHHCCCCeEEEEecCCCCCcccccccccccccHHHHHHHHHHHHHHH
```

```
Q ss_pred             C---CCEEEECHHHHhhhcccccHhhcCCCCcCCCCcHHHHHHHHHHHHHH
Q gi|45553317|re  410 C---HLKVIDIHSCLINERGVVRFDCFQASPRQVTGSKEPYLFWNKIGRQRV  458 (468)
Q Consensus      410 ~---gv~fIDl~~~~~~~~~~~g~~~~~~~~~~dDGvHPn~~~~Gy~~lA~~i~~~~I  458 (468)
                      +   ++.|||++..+.+.+..+.....++.|+ +|||.  +||++||+.+.+.|
T Consensus      165 ~~~~v~~iD~~~~~~~~~~~~~~~~~~~~~~~Dg~Hpn~~~G~~~~a~~l~~~~l  213 (214)
T 2hsj_A        165 SAYMQVEFVPVFDCLTDQAGQLKKEYTTDG-LHLSI--AGYQALSKSLKDYL  213 (214)
T ss_dssp            TTCTTEEEECCGGGSBCTTSSBCGGGBSSS-SSBCH--HHHHHHHHHHHHHH
T ss_pred             HHcCCcEEEEcHHHHhhccCCcccccCCCCC-cCcCH--HHHHHHHHHHHHHHh
```

No 41

>**1vjg_A** Putative lipase from the G-D-S-L family; structural genomics center for structural genomics, JCSG, protein structure INI PSI, hydrolase; 2.01A {Nostoc SP} SCOP: **c.23.10.6** PDB: **1z8h _A**

Probab=99.90 E-value=9.4e-23 Score=196.62 Aligned_cols=201 Identities=10% Similarity=0.025 Sum_probs=0.0

```
Q ss_pred             cccccccCCcCCCCCCCccEEEEEcchhccCCCCCCCcccccccccchHHHHHHHHHHHHhhcCCCCceEEEeCCCCcCCccHH
Q gi|45553317|re  245 GMNIMKRRHEMTPTPTILTSGTYNDSLLTINSDYDAYLLDFPLMGDDFMLYLARMELKCRFRRHERVLQSGLCVSGLTIN  324 (468)
Q Consensus       245 g~~~~~~~~~e~~~~~P~~~~Iv~iGDSIT~G~~~~~~~~~~~~~~~~liGds~~~~La~~~l~~~~~~~~~~~V~N~G~~isG~ts~  324 (468)
                      .........+....+...+|+|+|||||.|.....           +.+|...|+..+.. ...++.+.|.|  ++|.++.
T Consensus         4 ~~~~~~~~~~~~~~~~~~~~~~~i~~lGDSit~g~~~~~~---------------~~~~~~l~~~l~~~-----~~~~~~~~~n~g~~~G~~~~   70 (218)
T 1vjg_A          4 DKIHHHHHHMTKQSKTQIRICFVGDSFVNGTGDPE---------CLGWTGRVCVNANK--KGYDVTYYNLG--IRRDTSS   70 (218)
T ss_dssp            -------------CCEEEEEEEESHHHHTTCTT---------SCHHHHHHHHHHHH--TTEEEEEEEE--CTTCCHH
T ss_pred             cchhhhhhhhcccCCCeEEEEEcccccccCCCC---------CCChHHHHHHHHHhc--cCCCeEEEecC--CCCCcHH
```

```
Q ss_pred             HHHHHHHHHHhc-----CCCeEEEEeeeccCCC-----CCCHHHHHHHHHHHHHHHHhCCCEEEEEcCCCCCccccchhhH
Q gi|45553317|re  325 GARNRLKRVQLP-----EGTQIIVNIGSVDIMR-----GKPLVQIEHDFRLLIKEMHNMRLVPILTNLAPLGNYCHDKVL  394 (468)
Q Consensus       325 ~~~~rl~~~l~~------~PDlVvI~lGtND~~~-----g~s~e~f~~~l~~LI~~lr~~ga~VIL~t~~P~~~~~~~~~~~~~  394 (468)
                      ++..++...+..      +||+|+|++|+||+..     +.+.++|.++++++|+.++ .+++|||++++|.......  .
T Consensus        71 ~~~~~~~~~~~~~~~~~~~~pd~Vvi~~G~ND~~~~~~~~~~~~~~~~~~~~~~~~~l~~~i~~~~~~~~~~vi~~~~~p~~~~~~~~~~~   148 (218)
T 1vjg_A         71 DIAKRWLQEVSLRLHKEYNSLVVFSFGLNDTTLENGKPRVSIAETIKNTREILTQAK-KLYPVLMISPAPYIEQQDPG-R  148 (218)
T ss_dssp            HHHHHHHHHHHTTCCTTSEEEEEEECCHHHHCEETTEESSCHHHHHHHHHHHHHHHH-HHSCEEEECCCCCCTTCTT-H
T ss_pred             HHHHHHHHHHhccccCCCCEEEEEecccccccCCCCCcCHHHHHHHHHHHHHHHHHh-cCCCEEEEcCCCcccccHH-H
```

```
Q ss_pred             HHHHHHHHHHHHHHhC--CCEEEECHHHHhhhcccccHhhcCCCCcCCCCcHHHHHHHHHHHHHH--HHHHHHH
Q gi|45553317|re  395 CDKIYRFNKFIRSECC--HLKVIDIHSCLINERGVVRFDCFQASPRQVTGSKEPYLFWNKIGRQ--RVLQVIE  463 (468)
Q Consensus      395 ~~~i~~~N~~ir~~A~--gv~fIDl~~~~~~~~~~~g~~~~~~~~~~dDGvHPn~~~~Gy~~lA~~i~~--~I~~~L~  463 (468)
```

```
                          ...+..+|++++++|+  ++.|||++..+.+..+.....++.|+ +|||.  +||++||+.|++  .+.+.|+
T Consensus        149 ~~~~~~~n~~l~~~a~~~~v~~vD~~~~~~~~~~~~~~~~~~~~Dg-vHpn~--~G~~~~A~~i~~~~~~~~w~~  218 (218)
T 1vjg_A           149 RRRTIDLSQQLALVCQDLDVPYLDVFPLLEKPSVWLHEAKANDG-VHPQA--GGYTEFARIVENWDAWLNWFR  218 (218)
T ss_dssp              HHHHHHHHHHHHHHHHHHTCCEECCTGGGSTTSSHHHHHHHTTS-SCCCH--HHHHHHHHHHHTCHHHHTTC-
T ss_pred              HHHHHHHHHHHHHHHHHHhCCeEEehHHHHhhccchhhhcccCCC-CCCCH--HHHHHHHHHHHhchHHHHHhC
```

No 42

>**cd01834** SGNH_hydrolase_like_2 SGNH_hydrolase subfamily. SGNH hydrolases are a diverse family of lipases and esterases. The tertiary fold of the enzyme is substantially different from that of the alpha/beta hydrolase family and unique among all known hydrolases; its active site closely resembles the Ser-His-Asp(Glu) triad found in other serine hydrolases.
Probab=99.90 E-value=6.7e-22 Score=184.85 Aligned_cols=176 Identities=12% Similarity=0.072 Sum_probs=0.0

```
Q ss_pred              EEEEEcchhccCCCCCCccccccccchHHHHHHHHHHHhhcCCCceEEEeCCCcCCccHHHHHHHHHHHHhcC-CCeE
Q gi|45553317|re  263 TSGTYNDSLLTINSDYDAYLLDFPLMGDDFMLYLARMELKCRFRRHERVLQSGLCVSGLTINGARNRLKRVQLPE-GTQI  341 (468)
Q Consensus       263 ~Iv~iGDSIT~G~~~~~~~~~~~~~~liGds~~~~La~~~l~~~~~~~~~~~V~N~G~~isG~ts~~~~~~rl~~~l~~~-PDlV  341 (468)
                       +|+++|||||.|.                .|...+..++.......++.+.|.|  ++|.++.++..++...+... ||+|
T Consensus          3 ~i~~~GDSit~g~---------------~~~~~~~~~~l~~~~~~~~~~~~~~~n~~-~~G~~~~~~~~~~~~~~~~~~~~d~v   65 (191)
T cd01834          3 RIVFIGNSITDRG---------------GYVGYVETYLAARYPELKLTFRNLG--WSGDTVSDLAARRDRDVLPAKPDVV   65 (191)
T ss_pred              EEEEECCCccccCc---------------eeHHHHHHHHHHhCCCCCeEEEECc--cCCCcHhHHHHHhHHhhhhCCCCEE


Q ss_pred              EEEeeeccCCC----CCCHHHHHHHHHHHHHHHHhC--CCEEEEEcCCCCCcccc----hhhHHHHHHHHHHHHHHHhC-
Q gi|45553317|re  342 IVNIGSVDIMR----GKPLVQIEHDFRLLIKEMHNM--RLVPILTNLAPLGNYCH----DKVLCDKIYRFNKFIRSECC-  410 (468)
Q Consensus       342 vI~lGtND~~~----g~s~e~f~~~l~~LI~~lr~~--ga~VIL~t~~P~~~~~~~~~~----~~~~~i~~~N~~ir~~A~-  410 (468)
                       +|++|+||...    ..+.++|..+|+.+|+.+++.  +++|+++++++......    ....+..+..+|++++++|.
T Consensus         66 i~~G~ND~~~~~~~~~~~~~~~~~~~i~~~~~~~~~~vv~~~~~~~~~~~~~~~~~~~~~~~~~~~~~~~~~~~a~~  145 (191)
T cd01834         66 SIMFGINDSFRGFDDPVGLEKFKTNLRRLIDRLKNKESAPRIVLVSPIAYEANEDPLPDGAEYNANLAAYADAVRELAAE  145 (191)
T ss_pred              EEEccccchhhcccCcccHHHHHHHHHHHHHHHHHhcCCCceEEEECCccccccccCCcchhhHHHHHHHHHHHHHHHHH


Q ss_pred              -CCEEEECHHHHhhcccccHhhcCCCCcCCCCCcHHHHHHHHHHHHHH
Q gi|45553317|re  411 -HLKVIDIHSCLINERGVVRFDCFQASPRQVTGSKEPYLFWNKIGRQR  457 (468)
Q Consensus       411 -gv~fIDl~~~~~~~~~~g~~~~~~~~~~dDGvHPn~~~~Gy~~lA~~i~~~~  457 (468)
                        ++.|+|++..+.+........++..|++|||.  +||++||+.+++.
T Consensus        146 ~~~~~id~~~~~~~~~~~~~~~~~~~~~D~~Hp~~--~G~~~~a~~l~~~~  191 (191)
T cd01834        146 NGVAFVDLFTPMKEAFQKAGEAVLTVDGVHPNE--AGHRALARLWLEA  191 (191)
T ss_pred              cCCEEEecHHHHHHHHHhCCccceecCCCCCCCH--HHHHHHHHHHHhC
```

No 43

>**SUPFAM template** c.23.10 SGNH hydrolase (**52266**) SCOP seed sequence: d1vjga_.
Probab=99.89 E-value=2.9e-21 Score=170.84 Aligned_cols=187 Identities=18% Similarity=0.214 Sum_probs=0.0

```
Q ss_pred              CCCccEEEEEcchhccCCCCCCccccccccchHHHHHHHHHHHhhcCCCceEEEeCCCcCCccHHHHHHHHHHHH---
Q gi|45553317|re  258 TPTILTSGTYNDSLLTINSDYDAYLLDFPLMGDDFMLYLARMELKCRFRRHERVLQSGLCVSGLTINGARNRLKRVQ---  334 (468)
Q Consensus       258 ~P~~~~Iv~iGDSIT~G~~~~~~~~~~~~~~liGds~~~~La~~~l~~~~~~~~~~~V~N~G~~isG~ts~~~~~~rl~~~l---  334 (468)
                       .+...+|+++|||+|.|.+..          .+.+|...+.. .+...+. ++.+.|.|  ++|.++.++.++....
T Consensus          1 ~~~~~~~~~~~GDS~~~g~~~~~~---------~~~~~~~~~~~~~~~~~~~~~~~~~~~~~~~~n~g--~~g~~~~~~~~~~~~~~~~~~~   67 (201)
T SUPFAM0050207    1 SKKPLRIVALGDSITAGYGDP---------PGGGWVGLLAA-LLAALGP-GVTVINLG--VSGDTTAGLLARLEELLLLR   67 (201)
T ss_pred              CCCCceEEEECCchhcccCCc---------ccccHHHHHHH-HHHhhcC-CceEEEee--cccccHHHHHHHHHHHHHHH


Q ss_pred              --hcCCCeEEEEeeeccCCC-----CCCHHHHHHHHHHHHHHHHhCCCEEEEEcCCCCCcccchhhHHHHHHHHHHHHHHH
Q gi|45553317|re  335 --LPEGTQIIVNIGSVDIMR-----GKPLVQIEHDFRLLIKEMHNMRLVPILTNLAPLGNYCHDKVLCDKIYRFNKFIRS  407 (468)
Q Consensus       335 --~~~PDlVvI~lGtND~~~~~~-----g~s~e~f~~~l~~LI~~lr~~ga~VIL~t~~P~~~~~~~~~~~~~~~~~i~~~N~~ir~  407 (468)
                         ..+||+|+|++|+||...     +.+.+.+..++..+++.++ .++++++++++|....... .....+..+|+++++
T Consensus         68 ~~~~~~~~~~~~~G~ND~~~~~~~~~~~~~~~~~~~~~~~~~~~~~~~~~~~~~~~~~~~~~~~~~~~~~~~~~~~~~~~  145 (201)
T SUPFAM0050207   68 LEALKPDLVVLLLGTNDLLRVLGRPGVSLEEYRENLRAILELLR-PGAKVLLVGPPPVPELLLP-RLNERVAELNAALRE  145 (201)
T ss_pred              hhccCCCEEEEEeecccchhhcccccCCCHHHHHHHHHHHHHHHHh-cCCeEEEEecccchhhhh-hHHHHHHHHHHHHH


Q ss_pred              HhC--CCEEEECHHHHhhcccccHhhcCCCCcCCCCCcHHHHHHHHHHHHHHHHHHHH
Q gi|45553317|re  408 ECC--HLKVIDIHSCLINERGVVRFDCFQASPRQVTGSKEPYLFWNKIGRQRVLQVI  462 (468)
Q Consensus       408 ~A~--gv~fIDl~~~~~~~~~~g~~~~~~~~~~dDGvHPn~~~~Gy~~lA~~i~~~I~~~L  462 (468)
                       +|+  ++.++|++..+.+ ......+++||+|||.  +||..+|+.+.+.|.+.
T Consensus        146 ~~~~~~~~~~~~~~~~d~~~~~~~~~~~~~~~~~~~~~~~D~~H~~~~~G~~~~~~~~~~~l~~~~  199 (201)
T SUPFAM0050207  146 LAEELGVPFVDLFSLLLD-EEGLLPELLAADGLHPNA--AGYELIAEALLEALKELL  199 (201)
T ss_pred              HHHHcCCeeechHHHHhh-hhcccccccCCcCCcCCH--HHHHHHHHHHHHHHHHhh
```

No 44

>2hsjD00
Probab=99.89 E-value=9.7e-22 Score=191.10 Aligned_cols=173 Identities=18% Similarity=0.270 Sum_probs=0.0

```
Q ss_pred              CCCCccEEEEEcchhccCCCCCCccccccccchHHHHHHHHHHHhhcCCCceEEEeCCCcCCccHHHHHHHHHHHhc
Q gi|45553317|re  257 PTPTILTSGTYNDSLLTINSDYDAYLLDFPLMGDDFMLYLARMELKCRFRRHERVLQSGLCVSGLTINGARNRLKRVQLP  336 (468)
Q Consensus       257 ~~P~~~~Iv~iGDSIT~G~~~~~~~~~~~~~~liGds~~~~La~~~l~~~~~~~~~~~V~N~G~~isG~ts~~~~~~rl~~~l~~  336 (468)
                       ......+|+|+|||||.|.........                       .+.++|.|  ++|.++.+++.+++..+..
T Consensus         30 ~~~~~~~~~~~~~GDS~t~~~~~~~~~~~~~~~~~~~~~~~~~~~~~~~~~~~~~~n~G--~~g~~~~~~~~~~~~~~~~~   83 (214)
T 2hsjD00         30 AKEKEPKIVFLGDSITEGWGLWEELFA------------------------GLDVINRG--ISGDTTAGLLARLDEDLLA   83 (214)
T ss_pred              ccCCCCcEEEEccChhcCcCCChhhCC------------------------CCCEEEcc--CcHHHHHHHHHHHHhhhhh


Q ss_pred              C-CCeEEEEeeeccCCCCCCHHHHHHHHHHHHHHHHhC--CCEEEEEcCCCCCcccchh-----hHHHHHHHHHHHHHHHH
Q gi|45553317|re  337 E-GTQIIVNIGSVDIMRGKPLVQIEHDFRLLIKEMHNM--RLVPILTNLAPLGNYCHDK-----VLCDKIYRFNKFIRSE  408 (468)
Q Consensus       337 ~-PDlVvI~lGtND~~~g~s~e~f~~~l~~LI~~lr~~~~ga~VIL~t~~P~~~~~~~~~~~~~~~~~~i~~~N~~ir~~  408 (468)
                      . |++|+|++|+||+..+.+.+++.++++.+++.+++.  +++|++|++|++++.......   ...+.+..+|+|++++
T Consensus         84 ~~p~~~~~~~~~G~ND~~~~~~~~~~~~~~~~~~~~~~~~~~~~~~~~~~~~~~~~~~~~~~~p~~~~~~~~~~~~~~~~~  163 (214)
T 2hsjD00         84 LKPDLVVIMLGTNDLLRGVPLEEIAENLEAIIEKIRAKLPGAKILLLSILPVGEEAEEKEAPPNRTNEKIKELNEALKEL  163 (214)
T ss_pred              CCcCeEEEEcccccccCCCCHHHHHHHHHHHHHHHHHHCcCCeEEEEeCCcccCCChhhhcCchhhHHHHHHHHHHHHHH


Q ss_pred              hC---CCEEEECHHHHhhcccccHhhcCCCCcCCCCCcHHHHHHHHHHHHHH
Q gi|45553317|re  409 CC---HLKVIDIHSCLINERGVVRFDCFQASPRQVTGSKEPYLFWNKIGRQRV  458 (468)
Q Consensus       409 A~---gv~fIDl~~~~~~~~~~g~~~~~~~~~~dDGvHPn~~~~Gy~~lA~~i~~~I  458 (468)
```

```
                         |+   ++.|+|++..|.+..+.....++.|+ +||+.  +||++|++.+.+.|
T Consensus          164 a~~~~~~~~~~~~~~~~~~~~~~~~~~~~~~~Dg-~H~~~--~Gy~~~~~~~~~~~~l  213 (214)
T 2hsjD00            164 AEEEPGVTFVDLYSALLDEDGKLKEELTSDG-LHPNA--AGYELLAEALLPAL  213 (214)
T ss_pred                 HHHCCCeEEEEccHHHhCccCCCChhhCCCC-CCcCH--HHHHHHHHHHHHHh
```

No 45

>**1k7c_A** Rhamnogalacturonan acetylesterase; N-linked glycosylation, SGNH-hydrolase, hydrolase; HET: NAG MAN; 1.12A {Aspergillus aculeatus} SCOP: **c.23.10.4** PDB: **1dex _A*** **1deo _A*** **1pp4 _A*** **3c1u _A***

Probab=99.89 E-value=1.4e-22 Score=195.66 Aligned_cols=186 Identities=12% Similarity=0.030 Sum_probs=0.0

```
Q ss_pred                EEEEEcchhccCCCCCCCcccccccccchHHHHHHHHHHHHhhcCCCCceEEEeCCCcCCccHHHHHH--HHHHHHh-cCC-
Q gi|45553317|re      263 TSGTYNDSLLTINSDYDAYLLDFPLMGDDFMLYLARMELKCRFRRHERVLQSGLCVSGLTINGARN--RLKRVQL-PEG-   338 (468)
Q Consensus          263 ~Iv~iGDSIT~G~~~~~~~~~~~~~~~~liGds~~~~La~~~l~~~~~~~~~~~~V~N~G~~isG~ts~~~~~~~~--rl~~~~l~-~~~P-   338 (468)
                         +|+++|||||.|.........-...+.+.+                ++.+.|.|   ++|.++.++..  +++.++. .+|
T Consensus            2 ~i~~~GDSit~g~~~~~~~~~~~~~~~~~l~~~l----------------~~~v~n~g--~~G~~~~~~~~~~~~~~~~~~~~~~~~~~~~~~   64 (233)
T 1k7c_A              2 TVYLAGDSTMAKNGGGSGTNGWGEYLASYL---------------SATVVNDA--VAGRSARSYTREGRFENIADVVTAG   64 (233)
T ss_dssp                 EEEEECCTTTSTTTTSTTCCCGGGGSGGGB---------------SSEEEECC--CTTCCHHHHHHTTHHHHHHHHCCTT
T ss_pred                 eEEEEecccccCCCCCCCCCCHHHHHHHHh---------------CCceEecc--cCCcchhhhhccccHHHHHhcCCCC
```

```
Q ss_pred                CeEEEEeeeccCCCC-------------------------CCHHHHHHHHHHHHHHHHhCCCEEEEEcCCCCCccchhh
Q gi|45553317|re      339 TQIIVNIGSVDIMRG-------------------------KPLVQIEHDFRLLIKEMHNMRLVPILTNLAPLGNYCHDKV   393 (468)
Q Consensus          339 DlVvI~lGtND~~~g-------------------------~s~e~f~~~l~~LI~~lr~~ga~VIL~t~~P~~~~~~~~~~~~~   393 (468)
                         |+|+|++|+||+...                         .+.++|.++++++|+.+++.+++|||++++|...+....
T Consensus           65 d~vvi~~G~ND~~~~~~~~~~~r~~~~~~~~~~~~~~~~~~~~~~~~~~~~~~~~~l~~~i~~~~~~~~~~~~~vil~~~~p~~~~~~~~~~~-   143 (233)
T 1k7c_A             65 DYVIVEFGHNDGGSLSTDNGRTDCSGTGAEVCYSVYDGVNETILTFPAYLENAAKLFTAKGAKVILSSQTPNNPWETGT-   143 (233)
T ss_dssp                 CEEEECCCTTSCSCGGGCCSCCCBSSSSSCEEEEEETTEEEEEEBHHHHHHHHHHHHHHHTTCEEEEECCCCCCTTTTSS-
T ss_pred                 CEEEEEecCCCcccccccccccccccchhhhhcccccccccHHHHHHHHHHHHHHHHHHCCCEEEEECCCCCccCCCcc-
```

```
Q ss_pred                HHHHHHHHHHHHHHHHhC--CCEEEECHHHHhhh----cccccHhhcCCCCcCCCCcHHHHHHHHHHHHHHHHHHHHHHHhc
Q gi|45553317|re      394 LCDKIYRFNKFIRSECC--HLKVIDIHSCLINE----RGVVRFDCFQASPRQVTGSKEPYLFWNKIGRQRVLQVIETSLE   467 (468)
Q Consensus          394 ~~~~i~~~N~~ir~~A~--gv~fIDl~~~~~~~~~~~----g~~~~~~~~~~~dDGvHPn~~~~Gy~~lA~~i~~~I~~~L~~~~~a   467 (468)
                         ....+..||++++++|+  ++.|||++..+.+.    ......+++..||+|||.  +||++||+.+.+.|.+...+...
T Consensus          144 ~~~~~~~~~~n~~~~~~~a~~~~~v~~iD~~~~~~~~~~~~~~~~~~~~~~~~~~~~~~~~~Dg~Hpn~~-~G~~~~A~~i~~~l~~~~~~~~~~~~   221 (233)
T 1k7c_A            144 FVNSPTRFVEYAELAAEVAGVEYVDHWSYVDSIYETLGNATVNSYFPIDHTHTSP--AGAEVVAEAFLKAVVCTGTSLKS   221 (233)
T ss_dssp                 CCCCCCHHHHHHHHHHHHHHTCEEECHHHHHHHHHHHCHHHHHHTCSSSSSCCCH--HHHHHHHHHHHHHHHHHHTCGGGG
T ss_pred                 hhhhHHHHHHHHHHHHHHHHcCCeEEEhHHhHHHHHHHhChhhccccccCCCCCcCH--HHHHHHHHHHHHHHHhcCCChhh
```

```
Q ss_pred                C
Q gi|45553317|re      468 Y  468 (468)
Q Consensus          468 ~  468 (468)
                         |
T Consensus          222 ~  222 (233)
T 1k7c_A            222 V  222 (233)
T ss_dssp                 G
T ss_pred                 h
```

No 46

>**4jgg_A** Esterase TESA; alpha/beta/alpha, lysophospholipase, hydrolase; 1.90A {Pseudomonas aeruginosa} SCOP: **c.23.10.0**

Probab=99.89 E-value=1.1e-21 Score=186.76 Aligned_cols=180 Identities=13% Similarity=0.080 Sum_probs=0.0

```
Q ss_pred                CCCCccEEEEEcchhccCCCCCCCcccccccccchHHHHHHHHHHHHhhcCCCCceEEEeCCCcCCccHHHHHHHHHHHHH-h
Q gi|45553317|re      257 PTPTILTSGTYNDSLLTINSDYDAYLLDFPLMGDDFMLYLARMELKCRFRRHERVLQSGLCVSGLTINGARNRLKRVQ-L   335 (468)
Q Consensus          257 ~~P~~~~~Iv~iGDSIT~G~~~~~~~~~~~~~~~~liGds~~~~La~~~l~~~~~~~~~~~~V~N~G~~isG~ts~~~~~~rl~~~~l-~   335 (468)
                         ..+...+|+++|||+|.|....           .+.+|...++..+-..  ..++.+.|.|  ++|.++.++..+++..+ .
T Consensus           17 ~~~~~~~~~~i~~~GDSit~g~~~~~---------~~~~~~~~~~~~~~~~~~l~~~l~~~~---~~~~~~~~~~~~n~g--~~~G~~~~~~~~~~~~~~~~~~~~~~~~~~~   83 (207)
T 4jgg_A             17 QQAAAQTLLVVGDSISAALGLD---------TSQGWVALLQKRLADE--GYDYRVVNAS--ISGDTSAGGLARLPALLAE   83 (207)
T ss_dssp                 -----CEEEEEECHHHHTTTSC---------GGGSHHHHHHHHHHHT--TCCCEEEECC--CTTCCHHHHHHHHHHHHHH
T ss_pred                 CCCCCcEEEEECcccccCCCCC---------CCCchHHHHHHHHHhc--CCCeEEEecc--ccCccHHHHHHHHHHHhhh
```

```
Q ss_pred                cCCCeEEEEeeeccCCCCCCHHHHHHHHHHHHHHHHhCCCEEEEEcCCCCCccchhhHHHHHHHHHHHHHHHHHhC--CCE
Q gi|45553317|re      336 PEGTQIIVNIGSVDIMRGKPLVQIEHDFRLLIKEMHNMRLVPILTNLAPLGNYCHDKVLCDKIYRFNKFIRSECC--HLK   413 (468)
Q Consensus          336 ~~PDlVvI~lGtND~~~g~s~e~f~~~l~~LI~~lr~~ga~VIL~t~~P~~~~~~~~~~~~~~~~~~~~i~~~N~~ir~~A~--gv~   413 (468)
                         .+||+|+|++|+|+..+.+.++|.++++++++++.+++++.++++++......        .....++|++++++|+  ++.
T Consensus           84 ~~pd~vvi~~G~ND~~~~~~~~~~~~~~~~l~~~i~~~~~~~~~~~~~~~~~~~vvlv~~~~~p~~~~~~--------~~~~~~~~~n~~~~~~~a~~~~~   158 (207)
T 4jgg_A             84 EKPALVVIELGGNDGLRGMAPAQLQQNLASMAQKARAEGAKVLLLGIQLPPNYG-----PRYIEAFSRVYGAVAAQEKTA   158 (207)
T ss_dssp                 HCCSEEEEECCHHHHHTTCCHHHHHHHHHHHHHHHHHHHHHTCEEEEECCCCCGGGC-----HHHHHHHHHHHHHHHHHTCE
T ss_pred                 cCCCEEEEecccccccCCCHHHHHHHHHHHHHHHHHHHcCCCEEEEecCCCCCcC-----HHHHHHHHHHHHHHHHHHcCCe
```

```
Q ss_pred                EEECHHHHhhhcccccHhhcCCCCcCCCCcHHHHHHHHHHHHHHHHHH
Q gi|45553317|re      414 VIDIHSCLINERGVVRFDCFQASPRQVTGSKEPYLFWNKIGRQRVLQ   460 (468)
Q Consensus          414 fIDl~~~~~~~~~~~~g~~~~~~~~~~~dDGvHPn~~~~Gy~~lA~~i~~~I~~   460 (468)
                         |||++.......    .+++..||+|||.  +||+.||+.+++.|.+
T Consensus          159 ~id~~~~~~~~~~~~~~~----~~~~~~~~Dg~Hpn~~-~G~~~~a~~i~~~~~~   199 (207)
T 4jgg_A            159 LVPFFLEGVGGV----QGMMQADGIHPAL--AAQPRLLENVWPTLKP   199 (207)
T ss_dssp                 EETTTTTTTTTC----TTTBCTTSSSBCG--GGHHHHHHHHHHHHGG
T ss_pred                 EecchhhhccCC----cccccCCCCCCCH--HHHHHHHHHHHHHHHH
```

No 47

>**4q7q_A** Lipolytic protein G-D-S-L family; structural genomics, PSI-biology, protein structure initiati midwest center for structural genomics; HET: PG4; 1.45A {Chitinophaga pinensis}

Probab=99.88 E-value=8.3e-23 Score=201.42 Aligned_cols=192 Identities=13% Similarity=0.150 Sum_probs=0.0

```
Q ss_pred                CCCCccEEEEEcchhccCCCCCCCcccccccccchHHHHHHHHHHHHhhcCCCCceEEEeCCCcCCccHHHHHHHHHHHHHhc
Q gi|45553317|re      257 PTPTILTSGTYNDSLLTINSDYDAYLLDFPLMGDDFMLYLARMELKCRFRRHERVLQSGLCVSGLTINGARNRLKRVQLP   336 (468)
Q Consensus          257 ~~P~~~~~Iv~iGDSIT~G~~~~~~~~~~~~~~~~liGds~~~~La~~~l~~~~~~~~~~~~V~N~G~~isG~ts~~~~~~rl~~~~l~~   336 (468)
                         ..|..++|+++|||||.|+.........   .+.+|...|+..+      +++.+.|.|  ++|.++.++..+++.....
T Consensus            1 s~~~~~~~~i~~~GDSit~g~~~~~~~~~~~~~~~~~~~~~~~~~~l~~~l~~~~~~~~~~~~~~~v~N~g--~~~G~~~~~~~~~~~~~~~~~~~~~~~~~~~~   69 (266)
T 4q7q_A              1 SNAKELTWVAIGDSITYLNDHLDETGNR---VSKGYLTRLNEIL------PNLKYINQG--HNGWTSGGIAGNIDSLGLI   69 (266)
```

```
T ss_dssp            --CCCEEEEEEEESHHHHGGGCGGGGTTC---CSCCHHHHHHHHS------TTEEEEEEE--CTTCCHHHHHHTGGGSCCC
T ss_pred            CCCccceEEEEecccccCCccccCCC---cCCcHHHHHHHhc------CCCeeEeec--cCcccHhHHHHHHHhhccC


Q ss_pred             CCCeEEEEeeeccCCCCC------------CHHHHHHHHHHHHHHHhC--CCEEEEEcCCCCCccccchhhH--------
Q gi|45553317|re  337 EGTQIIVNIGSVDIMRGK------------PLVQIEHDFRLLIKEMHNM--RLVPILTNLAPLGNYCHDKVL--------  394 (468)
Q Consensus       337 ~PDlVvI~lGtND~~~g~------------s~e~f~~~l~~LI~~lr~~--ga~VIL~t~~P~~~~~~~~~~~~~~~~--------  394 (468)
                     +||+|+|++|+||+..+.            +.++|..+++++|+.+++.  +++||+++++|.........
T Consensus        70 ~~d~v~i~~G~ND~~~~~~~~~~~~~~~~~~~~~~~l~~~i~~l~~~~p~~~ivl~~p~~~~~~~~~~~~~~~~~~~~~~  149 (266)
T 4q7q_A          70 KADVYSVFLGTNDWWQGRPVGKLDDYQHDNGNTTVYGSFRIIISKIRQLNPEAKIVLITPMQRNDFVYIADAKNNAFGSY  149 (266)
T ss_dssp            CCSEEEEECCHHHHHHTCCCCCHHHHHSCCCSCSHHHHHHHHHHHHHHHHCTTCEEEEECCCCCCCEECTTCTTSEECCTT
T ss_pred            CCCEEEEEeecccchhhcCCCCchhhccccchhHHHHHHHHHHHHHHHHHhCCCCeEEEecCcccCCcccccccccccchhh


Q ss_pred             ----HHHHHHHHHHHHHHHhC--CCEEEEChHH-------HhhhcccccHh------------------------------
Q gi|45553317|re  395 ----CDKIYRFNKFIRSECC--HLKVIDIHSC-------LINERGVVRFD------------------------------  431 (468)
Q Consensus       395 ----~~~i~~~N~~ir~~A~--gv~fIDl~~--------------g~~~~~-----------------------------  431 (468)
                         ...+..||++++++|+  ++.|||++..       +..-.....+.
T Consensus       150 ~~~~~~~~~~~~n~~i~~~a~~~~v~~vD~~~~~~~~~~~~~~~~~~~~~~~~~~~~~~~p~~~~~~~~~~~~~~~~~~~~~  229 (266)
T 4q7q_A         150 QKKNGQTLEEFANAVLTIGRYEQIPVVDLYHHPLLTLRNMVKFKHLKNPKNGKYVNYKYPAFVNIPFNPENNEYPYPPAA  229 (266)
T ss_dssp            SCBTTBCHHHHHHHHHHHHHHHHHTCCEECCTTCTTCCGGGTBSCEEEECTTTSCEEEECTTGGGGSCCCTTSSCCSCCGGG
T ss_pred            hhhhhHHHHHHHHHHHHHHHHHHCCceEchhhhcccccccccchhhhhccccccccccccCcccccccccccccccccccccc


Q ss_pred             -hcCCCCcCCCCcHHHHHHHHHHHHHHHHHH
Q gi|45553317|re  432 -CFQASPRQVTGSKEPYLFWNKIGRQRVLQV  461 (468)
Q Consensus       432 -~~~dDGvHPn~~~~Gy~~lA~~i~~~I~~~  461 (468)
                      .+..||+|||.  +||++||+.|.+.|.++
T Consensus       230 ~~~~~~DgvHpn~--~~G~~~~~a~~l~~~~l~~~  258 (266)
T 4q7q_A         230 VNLTYDGLHPSD--KGNAIIASALADVFRQL  258 (266)
T ss_dssp            BSSBSSSSSBCH--HHHHHHHHHHHHHHHHT
T ss_pred            ccccccCCCcCH--HHHHHHHHHHHHHHHHhc
```

No 48

>**4h08_A** Putative hydrolase; GDSL-like lipase/acylhydrolase family protein, structural GE joint center for structural genomics, JCSG; HET: GOL; 1.80A {Bacteroides thetaiotaomicron}
Probab=99.88 E-value=1.3e-21 Score=186.06 Aligned_cols=179 Identities=13% Similarity=0.039 Sum_probs=0.0

```
Q ss_pred             cCCCCCCccEEEEEcchhccCCCCCCCCccccccccccchHHHHHHHHHHHHhhcCCCCceEEEEeCCCcCCccHHHHHHHHHHH
Q gi|45553317|re  254 EMTPTPTILTSGTYNDSLLTINSDYDAYLLDFPLMGDDFMLYLARMELKCRFRRHERVLQSGLCVSGLTINGARNRLKRV  333 (468)
Q Consensus       254 e~~~~P~~~~Iv~iGDSIT~G~~~~~~~~~~~~~~~~~~~liGds~~~~La~~~l~~~~~~~~~~~~V~N~G~~isG~ts~~~~~~rl~~~  333 (468)
                     .....|...+|+|+|||||.|                      |...+....-..  .....+.+.|  ++|.+..  ..+++..
T Consensus        13 ~~~~~~~~~~~~~~i~~~~GDSit~G-----------------~~~~~~~~~~~~~~~~~~~~~~~-~~~~~~~~g--~~~g~~~~~~~~~~~~~~l~~~~  69 (200)
T 4h08_A          13 PGANKTDLPHVLLIGNSITRG-----------------YYGKVEAALKEK--AYVGRLSNSK--SVGDPAL--IEELAVV  69 (200)
T ss_dssp            TTTTCCSSCEEEEEESHHHHH-----------------HHHHHHHHTTTT--CEEEEEEESC--CTTCHHH--HHHHHHH
T ss_pred            cCCCCCCCCcEEEEecchhhh-----------------hHHHHHHHcccc--ceecccccccc--CCCChhH--HHHHHHH


Q ss_pred             HhcC-CCeEEEEeeeccCCCCCCHHHHHHHHHHHHHHHhC--CCEEEEEcCCCCCccccchhhHH---HHHHHHHHHHH
Q gi|45553317|re  334 QLPE-GTQIIVNIGSVDIMRGKPLVQIEHDFRLLIKEMHNM--RLVPILTNLAPLGNYCHDKVLC---DKIYRFNKFIRS  407 (468)
Q Consensus       334 l~~~~-PDlVvI~lGtND~~~g~s~e~f~~~l~~LI~~lr~~--ga~VIL~t~~P~~~~~~~~~~~~~~~~~~~---~~i~~~N~~ir~  407 (468)
                     +...  ||+|+|++|+||+.  .+.++|.++++++++|+.++++  +++||+++++|.........   ..+..||+++++
T Consensus        70 ~~~~~pd~Vvi~~G~ND~~~--~~~~~~~~~~~~~l~~li~~~r~~~~~~ivl~~~p~~~~~~~~~~~~~~~~~~~~~~~~~~n~~~~  147 (200)
T 4h08_A          70 LKNTKFDVIHFNNGLHGFD--YTEEEYDKSFPKLIKIIRKYAPKAKLIWANTTPVRTGEGMKEFAPITERLNVRNQIALK  147 (200)
T ss_dssp            HHHSCCSEEEECCCSSCTT--SCHHHHHHHHHHHHHHHHCTTCEEEEECCCCCEESGGGCEECTHHHHHHHHHHHHHH
T ss_pred            hhccCCCEEEEEccccccC--CCHHHHHHHHHHHHHHHHHHHCCCCEEEEEeCCCCCCcccccccchhhhHHHHHHHHHHHH


Q ss_pred             HhC--CCEEEEChHHHhhhccccHhhcC-CCCcCCCCcHHHHHHHHHHHHHHHHHHHHHHH
Q gi|45553317|re  408 ECC--HLKVIDIHSCLINERGVVRFDCFQ-ASPRQVTGSKEPYLFWNKIGRQRVLQVIET  464 (468)
Q Consensus       408 ~A~--gv~fIDl~~~~~~~~~~~g~~~~~~~~~~-dDGvHPn~~~~Gy~~lA~~i~~~I~~~~L~~  464 (468)
                     +|+  ++.|+|++..+.+      ..+++. .||+|||.  +||++||+.+.+.|.++|++
T Consensus       148 ~a~~~~v~~vD~~~~~~-------~~~~~~~~~~~~~~Dg~Hpn~--~~G~~~~~a~~i~~~~l~~l~k  200 (200)
T 4h08_A         148 HINRASIEVNDLWKVVID-----HPEYYAGGDGTHPID--AGYSALANQVIKVIKNVLVH  200 (200)
T ss_dssp            HHHHTTCEEECHHHHHTT-----CGGGTTTSCSSSCCH--HHHHHHHHHHHHHHHHHHHSCC
T ss_pred            HHHHcCCEEEEHHHhhcc-----CcceeecCCCCCcCH--HHHHHHHHHHHHHHHHHHHccC
```

No 49

>1ivnA00
Probab=99.88 E-value=1.2e-20 Score=180.94 Aligned_cols=176 Identities=21% Similarity=0.278 Sum_probs=0.0

```
Q ss_pred             cEEEEEcchhccCCCCCCCCcccccccccchHHHHHHHHHHHHhhcCCCCceEEEEeCCCcCCccHHHHHHHHHHHHhcC-CCe
Q gi|45553317|re  262 LTSGTYNDSLLTINSDYDAYLLDFPLMGDDFMLYLARMELKCRFRRHERVLQSGLCVSGLTINGARNRLKRVQLPE-GTQ  340 (468)
Q Consensus       262 ~~Iv~iGDSIT~G~~~~~~~~~~~~~~~~~~~liGds~~~~La~~~l~~~~~~~~~~~~V~N~G~~isG~ts~~~~~~rl~~~l~~~~-PDl  340 (468)
                     .+|+++|||+|.|....            -+++|...+...+      ...+.|+|.|  ++|.++.+++.+++..+...  ||+
T Consensus         2 ~~~~~~~~GDS~~~~~~~~~~~~~~~~~~~~~~~~~~~~l~~~~l~~~~~~~~~~~~~n~g~~~G~~~~~~~~~~r~~~~~~~~~~~~~p~~  65 (190)
T 1ivnA00           2 VTILALGDSLTAGYGLP---------PEEGWVALLQERL-----GADVTVINAG--VSGDTTAGGLARLEALLLAHKPDL  65 (190)
T ss_pred            CEEEEEchhhccCCCCC---------CCCChHHHHHHHh-----CCCCEEEEcC--CChhhHHHHHHHHHHHHHHhcCCCE


Q ss_pred             EEEEeeeccCCCCCCHHHHHHHHHHHHHHHhCCCCEEEEEcCCCCCccccchhHHHHHHHHHHHHHHHHhC--CCEEEECh
Q gi|45553317|re  341 IIVNIGSVDIMRGKPLVQIEHDFRLLIKEMHNMRLVPILTNLAPLGNYCHDKVLCDKIYRFNKFIRSECC--HLKVIDIH  418 (468)
Q Consensus       341 VvI~lGtND~~~g~s~e~f~~~l~~LI~~lr~~ga~VIL~t~~P~~~~~~~~~~~~~~~~~i~~~N~~ir~~A~--gv~fIDl~  418 (468)
                     |+|++|+||..++.+.+.+.++++.+|+.+++.+++||+.+.....      ..+.....|+++++++|+  ++.++|++
T Consensus        66 v~~~~G~ND~~~~~~~~~~~~~~~~~~~~~~~~~~~~~~l~~~~~~~~~~~~~~~~~~~~~~~~a~~~~~~~~~~  140 (190)
T 1ivnA00          66 VIIELGGNDLLRGLPPEETRANLRAIIEKLRAAGAKVLLVGMPPPPNY-----GNEYLEAFNAIYRELAKEYGVPLVDFF  140 (190)
T ss_pred            EEEEcCccccccCCCHHHHHHHHHHHHHHHHhCcCEEEEEeccCCCCcc-----CHHHHHHHHHHHHHHHHhcCcEEEeCh


Q ss_pred             HHHhhhccccHhhcCCCCcCCCCcHHHHHHHHHHHHHHHHHHHHHHHH
Q gi|45553317|re  419 SCLINERGVVRFDCFQASPRQVTGSKEPYLFWNKIGRQRVLQVIET  464 (468)
Q Consensus       419 ~~~~~~~g~~~~~~~~~dDGvHPn~~~~Gy~~lA~~i~~~I~~~~L~~~  464 (468)
                     ..+....    .+++.|++|+.+  +||++||+.+.+.|.++++.
T Consensus       141 ~~~~~~~~~~~~~~~~~D~~Hp~~~~~g~~~~~~~~~~~~~~~~~~~~~  180 (190)
T 1ivnA00         141 LELVAED----PELLQADGIHPNA--EGYRLIAEAILPALEKLLKE  180 (190)
T ss_pred            HHHhcCC----hhhcCCCCCCcCH--HHHHHHHHHHHHHHHHHHhh
```

No 50

>1fxw_F Alpha2, platelet-activating factor acetylhydrolase IB beta subunit; alpha beta hydrolase fold; 2.10A {Bos taurus} SCOP: c.23.10.3 PDB: 1vyh _A
Probab=99.88 E-value=1.2e-21 Score=189.29 Aligned_cols=178 Identities=16% Similarity=0.174 Sum_probs=0.0

```
Q ss_pred               CccEEEEEcchhccCCCCCCCcccccccccccchHHHHHHHHHHHHhhcCCCceEEEeCCCcCCccHHHHHHHHHH-HH-hcC
Q gi|45553317|re  260  TILTSGTYNDSLLTINSDYDAYLLDFPLMGDDFMLYLARMELKCRFRRHERVLQSGLCVSGLTINGARNRLKR-VQ-LPE  337 (468)
Q Consensus       260  ~~~~Iv~iGDSIT~G~~~~~~~~~~~~~liGds~~~~La~~~l~~~~~~~~~~V~N~G~~isG~ts~~~~~rl~~-~l-~~~  337 (468)
                       ...+|+|+|||+|.|..           .+..|...+.          ++.+.|.|  ++|.++.++..++.. .+ ..+
T Consensus        38  ~~~~i~~~GDS~t~g~~-----------~~~~~~~~~~~~----------~~~v~n~g--~~G~~~~~~~~~~~~~~~~~~~~   94 (229)
T 1fxw_F          38  KEPDVLFVGDSMVQLMQ-----------QYEIWRELFS----------PLHALNFG--IGGDTTRHVLWRLKNGELENIK   94 (229)
T ss_dssp              CCCSEEEEESHHHHGGG-----------GSHHHHHHTG----------GGTEEEEE--CTTCCHHHHHHHHHTTTTSSCC
T ss_pred              CCCCEEEEccchhcCCC-----------CcccHHHhCC----------CCCeEEec--cCchhHHHHHHHhhccccccCC


Q ss_pred               CCeEEEEeeeccCCCCCCHHHHHHHHHHHHHHHHhC--CCEEEEEcCCCCCccccchhhHHHHHHHHHHHHHHHHhC---CC
Q gi|45553317|re  338  GTQIIVNIGSVDIMRGKPLVQIEHDFRLLIKEMHNM--RLVPILTNLAPLGNYCHDKVLCDKIYRFNKFIRSECC---HL  412 (468)
Q Consensus       338  PDlVvI~lGtND~~~g~s~e~f~~~l~~LI~~lr~~--ga~VIL~t~~P~~~~~~~~~~~~~~~~~i~~~N~~ir~~A~---gv  412 (468)
                       ||+|||++|+||+  +.+.++|.++++++|+.+++.  +++|||++++|......  .....+..+|++++++++   ++
T Consensus        95  pd~vvi~~G~ND~--~~~~~~~~~~~~~~~~~i~~l~~~~p~~~vv~~~~~~p~~~~~~~--~~~~~~~~~~~~n~~l~~~~~~~~~v  170 (229)
T 1fxw_F          95  PKVIVVWVGTNNH--ENTAEEVAGGIEAIVQLINTRQPQAKIIVLGLLPRGEKPN--PLRQKNAKVNQLLKVSLPKLANV  170 (229)
T ss_dssp              CSEEEEECCTTCT--TSCHHHHHHHHHHHHHHHHHHHCTTCEEEEECCCCSSSCC--HHHHHHHHHHHHHHHHSSSSSE
T ss_pred              CCEEEEEeccCCC--CCCHHHHHHHHHHHHHHHHHHHCCCCeEEEEeCCCCCCcch--hhHHHHHHHHHHHHHHHHHHhcCCc


Q ss_pred               EEEEChHHHhhhcccccHhhcCCCCcCCCCcHHHHHHHHHHHHHHHHHHHHHHHhc
Q gi|45553317|re  413  KVIDIHSCLINERGVVRFDCFQASPRQVTGSKEPYLFWNKIGRQRVLQVIETSLE  467 (468)
Q Consensus       413  ~fIDl~~~~~~~~~g~~~~~~~~~~dDGvHPn~~~~Gy~~lA~~i~~~I~~~L~~~~a  467 (468)
                       .|||++..+.+..+.....++.|+ +|||.  +||++||+.|.+.|.|.+++.+...
T Consensus       171  ~~vD~~~~~~~~~~~~~~~~~~~~~~~~~~Dg-~Hpn~-~~G~~~~~a~~l~~~~l~~~l~~~~l~~~~~  222 (229)
T 1fxw_F         171  QLLDTDGGFVHSDGAISCHDMFDF-LHLTG--GGYAKICKPLHELIMQLLEETPE  222 (229)
T ss_dssp              EEECCCCSCBCTTSCBCTTTBTTS-SSBCH--HHHHHHHHHHHHHHHHHHC-----
T ss_pred              EEEechhhhcccCCCcccccccCCC-cCccH--HHHHHHHHHHHHHHHHHHHhccCCc
```

No 51

>SUPFAM template c.23.10 SGNH hydrolase (52266) SCOP seed sequence: d1es9a_.
Probab=99.88 E-value=1.1e-20 Score=168.46 Aligned_cols=176 Identities=20% Similarity=0.269 Sum_probs=0.0

```
Q ss_pred               CCCCccEEEEEcchhccCCCCCCCcccccccccccchHHHHHHHHHHHHhhcCCCceEEEeCCCcCCccHHHHHHHHHH-HHh
Q gi|45553317|re  257  PTPTILTSGTYNDSLLTINSDYDAYLLDFPLMGDDFMLYLARMELKCRFRRHERVLQSGLCVSGLTINGARNRLKR-VQL  335 (468)
Q Consensus       257  ~~P~~~~Iv~iGDSIT~G~~~~~~~~~~~~~liGds~~~~La~~~l~~~~~~~~~~V~N~G~~isG~ts~~~~~rl~~-~l~  335 (468)
                       ..+...+++++|||+|.|..           ..+.|...+.          +..+.|.|  ++|.++.++..++.. .+.
T Consensus        30  ~~~~~~~~~~~~~~~GdS~~~~~~~~------------~~~~~~~~~~~~~~~~~~~~~~~~~~~n~G--~~G~~~~~~~~~~~~~~~~~~~~   86 (212)
T SUPFAM0046616   30  AKPKPVRIVFLGDSITAGWG-----------LTEGWPELLA----------GLTVINLG--IGGDTTADLLARLERDVLD   86 (212)
T ss_pred              hcCCCCcEEEecccchhhccc-----------ccchHHHHhc----------CCceEeec--cccchHHHHHHHHHhhhh


Q ss_pred               -cCCCeEEEEeeeccCCCCCCHHHHHHHHHHHHHHHHhC--CCEEEEEcCCCCCccccchhhHHHHHHHHHHHHHH-HhC-
Q gi|45553317|re  336  -PEGTQIIVNIGSVDIMRGKPLVQIEHDFRLLIKEMHNM--RLVPILTNLAPLGNYCHDKVLCDKIYRFNKFIRS-ECC-  410 (468)
Q Consensus       336  -~~PDlVvI~lGtND~~~g~s~e~f~~~l~~LI~~lr~~--ga~VIL~t~~P~~~~~~~~~~~~~~~~~~i~~~N~~ir~-~A~-  410 (468)
                        .+|||+|+|++|+||.  +.+.+.+.++++.+++.+++.  +++|+++++|.....  ...+.+..+|+.+++ +|.
T Consensus        87  ~~~P~~v~~~~G~ND~--~~~~~~~~~~~~~~~~~~~~p~~~~~~~~~~~~--~~~~~~~~~~~~~~~~~~~~~~~  162 (212)
T SUPFAM0046616   87  RLKPDLVVLLLGTNDL--GLSPEEILANLEALIELLRAKLPGAKIVLLGILPVGELGD--RLNEKIAALNEALRELLAKE  162 (212)
T ss_pred              ccCCCEEEEEecCCCC--CCCHHHHHHHHHHHHHHHHHhCCCCeEEEEeccccccchh--hHHHHHHHHHHHHHHHHhhh


Q ss_pred               -CCEEEEChHHHhhhcccccHhhcCCCCcCCCCcHHHHHHHHHHHHHHHHHHH
Q gi|45553317|re  411  -HLKVIDIHSCLINERGVVRFDCFQASPRQVTGSKEPYLFWNKIGRQRVLQVI  462 (468)
Q Consensus       411  -gv~fIDl~~~~~~~~~g~~~~~~~~~~dDGvHPn~~~~Gy~~lA~~i~~~I~~~L  462 (468)
                        ++.|+|++..+.+..+...  ..+..||+|||.  .||..|++.+.+.|.+.|
T Consensus       163  ~~~~~~~~~~~~~~~~~~~~~~~~~~-~~~~~D~~H~~~~-~~G~~~~~~~~~~~~~~l~~~~l  212 (212)
T SUPFAM0046616  163  EGVPFVDLYSLLLDEDGLLP-ELLTADGLHPNA--AGYALLAEALLPALLELL  212 (212)
T ss_pred              cCceEeechhhhcccccc-cccccCcCcCH--HHHHHHHHHHHHHHHHHHhC
```

No 52

>1z8hA00
Probab=99.88 E-value=1.5e-20 Score=182.13 Aligned_cols=186 Identities=20% Similarity=0.273 Sum_probs=0.0

```
Q ss_pred               CCccEEEEEcchhccCCCCCCCcccccccccccchHHHHHHHHHHHHhhcCCCceEEEeCCCcCCccHHHHHHHHHH-HH---
Q gi|45553317|re  259  PTILTSGTYNDSLLTINSDYDAYLLDFPLMGDDFMLYLARMELKCRFRRHERVLQSGLCVSGLTINGARNRLKR-VQ---  334 (468)
Q Consensus       259  P~~~~Iv~iGDSIT~G~~~~~~~~~~~~~liGds~~~~La~~~l~~~~~~~~~~V~N~G~~isG~ts~~~~~rl~~-~l---  334 (468)
                       ....+|+++|||+|.|..+.          -+.+|...+..  .+.....  .+.+.|.|  ++|.++.++..++.. ..
T Consensus         2  ~~~~~~~~~~~GDS~t~g~g~~---------~~~~~~~~~~~~~~~~~~-~~~~~~~~~~~-~~~~~~~~n~G--~~g~~~~~~~~r~~~~~~~~~~~   68 (202)
T 1z8hA00           2  AEKVRIVALGDSLTEGYGDP---------EGGGWVARLAA-RLKEKGP-DVTVINLG--VSGDTTAGLLARLEEDVLLRK   68 (202)
T ss_pred              CCCcEEEEEchhhccCcCCC---------CCCchHHHHHH-HHHHHCc-CcEEEEee--ECchhHHHHHHHHHHHHHhc


Q ss_pred               -hcCCCeEEEEeeeccCCC-----CCCHHHHHHHHHHHHHHHHhCCCEEEEEcCCCCCccccchhhHHHHHHHHHHHHHHHHH
Q gi|45553317|re  335  -LPEGTQIIVNIGSVDIMR-----GKPLVQIEHDFRLLIKEMHNMRLVPILTNLAPLGNYCHDKVLCDKIYRFNKFIRSE  408 (468)
Q Consensus       335  -~~~PDlVvI~lGtND~~~-----g~s~e~f~~~l~~LI~~lr~~ga~VIL~t~~P~~~~~~~~~~~~~~~~~i~~~N~~ir~~  408 (468)
                        ..+||+|+|.+|+||+..     +.+.+.+..+++.+++.++. +++|++++++........  ....+..+|+.++++
T Consensus        69  ~~~~~~~~~~~~~~~G~ND~~~~~~~~~~~~~~~~~~~~~~~~~~~~~~~~~~~~~-~~~~~~~~~~~~~~~~~~~~~~~~~~-~~~~~~  146 (202)
T 1z8hA00          69  EALKPDLVVLSIGGNDLLRVKGREGVDPEEYRENLREILERLRA-GAKVLLVGPLPPPEYGMPE-LNERIAELNAALREL  146 (202)
T ss_pred              ccCCCCeEEEEechhhchHhhCCCCCcCHHHHHHHHHHHHHHHHHcC-CCcEEEEeCcCCCcCCCh-hHHHHHHHHHHHHHH


Q ss_pred               hC--CCEEEEChHHHhhhcccccHhhcCCCCcCCCCcHHHHHHHHHHHHHHHHHHHH
Q gi|45553317|re  409  CC--HLKVIDIHSCLINERGVVRFDCFQASPRQVTGSKEPYLFWNKIGRQRVLQVI  462 (468)
Q Consensus       409  A~--gv~fIDl~~~~~~~~~g~~~~~~~~~~dDGvHPn~~~~Gy~~lA~~i~~~I~~~L  462 (468)
                       |.  ++.|+|++..|........ +++..||+|||+.  +||..||+.+.+.+....
T Consensus       147  ~~~~~~~~~~~~~~~~~~~~~~~~~~~~~~~~~~~~~~-~~~~~D~~Hp~~~-~~G~~~~~~~~~~~~~~~~~~~~  199 (202)
T 1z8hA00         147  AKEYGVPLVDLFDLLLDGEGGLP-ELLAADGLHPNA--AGYALIAEAVLEALKKLL  199 (202)
T ss_pred              HHhCCeEEEehHHHHHhccccCh-hhcCCCCCcCH--HHHHHHHHHHHHHHHHHHh
```

No 53

>**SUPFAM template** c.23.10 SGNH hydrolase (**52266**) SCOP seed sequence: d1jrla_.

```
Probab=99.87  E-value=4.1e-20  Score=160.64  Aligned_cols=175  Identities=20%  Similarity=0.245  Sum_probs=0.0

Q ss_pred             ccEEEEEcchhccCCCCCCCcccccccccchHHHHHHHHHHHHhhcCCCceEEEeCCCcCCccHHHHHHHHHHHHhcC-CC
Q gi|45553317|re  261 ILTSGTYNDSLLTINSDYDAYLLDFPLMGDDFMLYLARMELKCRFRRHERVLQSGLCVSGLTINGARNRLKRVQLPE-GT  339 (468)
Q Consensus      261 ~~~Iv~iGDSIT~G~~~~~~~~~~~~~~liGds~~~~La~~~l~~~~~~~~~~~V~N~G~~isG~ts~~~~~~rl~~~l~~~-PD  339 (468)
                    ..+|+++|||+|.|....           .+..|...+...+      ..++.+.|.|  ++|.++.++..++...+... ||
T Consensus        1 ~~~i~~~GDS~~~~~~~~~~~--------------------------~~~~~~~~~~~~n~g--~~g~~~~~~~~~~~~~~~~~~~~~~~   64 (179)
T SUPFAM0047668    1 PLRILLLGDSITAGYGLS---------PGGGWPALLAEAL-----GAGVTVINLG--ISGDTTADGLARLDALLLALKPD   64 (179)
T ss_pred             CceEEEecCchhhccccc---------ccccHHHHHHHHh-----ccCceEEeec--cccchHHHHHHHHHHHHHHHhcCCC


Q ss_pred             eEEEEeeeccCCCCCCHHHHHHHHHHHHHHHHHhCCCEEEEEcCCCCCccccchhhHHHHHHHHHHHHHHHhC--CCEEEEC
Q gi|45553317|re  340 QIIVNIGSVDIMRGKPLVQIEHDFRLLIKEMHNMRLVPILTNLAPLGNYCHDKVLCDKIYRFNKFIRSECC--HLKVIDI  417 (468)
Q Consensus      340 lVvI~lGtND~~~g~s~e~f~~~l~~LI~~lr~~ga~VIL~t~~P~~~~~~~~~~~~~~~~~~~i~~~N~~ir~~A~--gv~fIDl  417 (468)
                    +|+|++|+||...+.+.+.+..+++.+++.+++.++.+++++++++...     ...+....+++.++++|.  ++.++|+
T Consensus       65 ~v~~~~G~ND~~~~~~~~~~~~~~~~~~~~~~~~~~~~~~~~~~~~~~----~~~~~~~~~~~~~~~~~~~~~~~~~~~d~  139 (179)
T SUPFAM0047668   65 LVIILLGTNDLLRGVSPEEFRANLRALIERLRAPGAKVVLVTPPPPPN-----YLNDYLAALNDALRELAKELGVPLVDL  139 (179)
T ss_pred             EEEEEeccccccccCCHHHHHHHHHHHHHHHHhccCCeEEEEeeccccc-----hhhHHHHHHHHHHHHHHHHHcCCeEech


Q ss_pred             hHHHhhhcccccHhhcCCCCcCCCCcHHHHHHHHHHHHHHHHH
Q gi|45553317|re  418 HSCLINERGVVRFDCFQASPRQVTGSKEPYLFWNKIGRQRVLQVI  462 (468)
Q Consensus      418 ~~~~~~~~~~g~~~~~~~~~~~dDGvHPn~~~~Gy~~lA~~i~~~~I~~~~L  462 (468)
                    +..+....+    +++..||+|||.  .||..+++.+.+.|.+.+
T Consensus      140 ~~~~~~~~~~~~~----~~~~~~D~~H~~~--~~g~~~~~~~~~~~~~l~~~~~~  178 (179)
T SUPFAM0047668  140 FALLLDLDG----ELLLADGLHPNA--AGYRLIAEALAEALLKLL  178 (179)
T ss_pred             HHHHhhhcc----ccccccCCcCH--HHHHHHHHHHHHHHHhhc
```

No 54

>**TIGR03304** TIGR03304 outer membrane insertion C-terminal signal; InterPro:
Beta-barrel outer

membrane proteins (OMP) rely on Omp85-like proteins for insertion into the outer membrane. The targeting signal for Omp85 is usually found in the C terminus of OMPs, and the target sequences differ somewhat by species. Observations in Escherichia coli mutant PhoE protein and neisserial OMPs suggest that there are alternative, less-efficient recognition sites in the protein that mediate binding to Omp85 [].

This entry represents a targeting
signal for outer membrane insertion.
.

```
Probab=99.79  E-value=3.2e-23  Score=6.95  Aligned_cols=6  Identities=17%  Similarity=0.385  Sum_probs=0.0

Q ss_pred             EEeeec
Q gi|45553317|re  343 VNIGSV  348 (468)
Q Consensus      343 I~lGtN  348 (468)
                    +.+|.+
T Consensus        2 ~~~g~~    7 (10)
T TIGR03304        2 FGLGVR    7 (10)
```

No 55

>**1es9_A** PAF-AH, platelet-activating factor acetylhydrolase IB gamma subunit; alpha/beta hydrolase fold; 1.30A
{Bos taurus} SCOP: **c.23.10.3** PDB: **1wab _A 1fxw _A 1bwr _A 1bwq _A 1bwp _A 3dt9 _A* 3dt6 _A* 3dt8 _A***

```
Probab=99.87  E-value=1.3e-21  Score=189.48  Aligned_cols=175  Identities=13%  Similarity=0.151  Sum_probs=0.0

Q ss_pred             CccEEEEEcchhccCCCCCCCcccccccccchHHHHHHHHHHHHhhcCCCceEEEeCCCcCCccHHHHHHHHHH-HHh-cC
Q gi|45553317|re  260 TILTSGTYNDSLLTINSDYDAYLLDFPLMGDDFMLYLARMELKCRFRRHERVLQSGLCVSGLTINGARNRLKR-VQL-PE  337 (468)
Q Consensus      260 ~~~~Iv~iGDSIT~G~~~~~~~~~~~~~~liGds~~~~La~~~l~~~~~~~~~~~V~N~G~~isG~ts~~~~~~rl~~~~l~-~~  337 (468)
                    ...+|+|+|||+|.|..          .+..|...+.          ++.++|.|  ++|.++.++..++.. .+. .+
T Consensus       37 ~~~~i~~~GDS~t~g~~~~-----------~~~~~~~~~~~~~~~~~~---------~~v~n~g--~~G~~~~~~~~~~~~~   93 (232)
T 1es9_A         37 KEPEVVFIGDSLVQLMH-----------QCEIWRELFS----------PLHALNFG--IGGDSTQHVLWRLENGELEHIR   93 (232)
T ss_dssp             CCCSEEEEESHHHHTHH-----------HHSCHHHHTG----------GGCEEEEE--CTTCCHHHHHHHCCCCCTTTCC
T ss_pred             CCCCEEEEecchhhccC-----------CcchHHHHcC----------CCceEEec--cCccccHHHHHHHHhcCcccccCC


Q ss_pred             CCeEEEEeeeccCCCCCCHHHHHHHHHHHHHHHHHhC--CCEEEEEcCCCCCccccchhhHHHHHHHHHHHHHHHhC---CC
Q gi|45553317|re  338 GTQIIVNIGSVDIMRGKPLVQIEHDFRLLIKEMHNM--RLVPILTNLAPLGNYCHDKVLCDKIYRFNKFIRSECC---HL  412 (468)
Q Consensus      338 PDlVvI~lGtND~~~g~s~e~f~~~l~~LI~~lr~~~~ga~VIL~t~~P~~~~~~~~~~~~~~~~~~~i~~~N~~ir~~A~~~~~gv  412 (468)
                    ||+|+|++|+||+.  .+.++|.++++++|+.+++.  +++|||++++|......     .....+..+|+++++++++   ++
T Consensus       94 pd~vvi~~G~ND~~~~~~~~~~~~~~~~~~~i~~l~~~~~~p~~~~~~~~~~~~~~~~~~~~~~~~~~n~~l~~~~~~a~~~~~v  169 (232)
T 1es9_A         94 PKIVVVWVGTNNHG--HTAEQVTGGIKAIVQLVNERQPQARVVVLGLLPRGQHPN--PLREKNRRVNELVRAALAGHPRA  169 (232)
T ss_dssp             CSEEEEECCTTCTT--SCHHHHHHHHHHHHHHHHHSTTCEEEEECCCCCCCCC--HHHHHHHHHHHHHHHHHSCTTE
T ss_pred             CCEEEEEeccCCCC--CCHHHHHHHHHHHHHHHHHCCCCeEEEecCCCCCcccch--hHHHHHHHHHHHHHHHHhcCCCCe


Q ss_pred             EEEEChHHHhhhcccccHhhcCCCCcCCCCcHHHHHHHHHHHHHHHHHH
Q gi|45553317|re  413 KVIDIHSCLINERGVVRFDCFQASPRQVTGSKEPYLFWNKIGRQRVLQVIET  464 (468)
Q Consensus      413 ~fIDl~~~~~~~~~~g~~~~~~~~~~~dDGvHPn~~~~Gy~~lA~~i~~~~I~~~~L~~  464 (468)
                    .|||++..+.+..+.....++.|+ +|||.  +||++||+|.+.|.+++.+
T Consensus      170 ~iD~~~~~~~~~~~~~~~~~~~~~~~~~~~Dg-~Hpn~~~G~~~~a~~i~~~~l~~~l~~  218 (232)
T 1es9_A        170 HFLDADPGFVHSDGTISHHDMYDY-LHLSR--LGYTPVCRALHSLLLRLLTQ  218 (232)
T ss_dssp             EEECCCCCCSCTTSCCCTTTBTTS-SSBCH--HHHHHHHHHHHHHHHHHC--
T ss_pred             EEEeCChhhcCCCCCCCCcccCcC-CCCCH--HHHHHHHHHHHHHHHHHhcC
```

No 56

>**cd01829** SGNH_hydrolase_peri2 SGNH_peri2; putative periplasmic member of the SGNH-family of hydrolases, a diverse family of lipases and esterases. The tertiary fold of the enzyme is substantially different from that of the alpha/beta hydrolase family and unique among all known hydrolases; its active site closely resembles the Ser-His-Asp(Glu) triad found in other serine hydrolases.

```
Probab=99.87  E-value=6.7e-21  Score=182.74  Aligned_cols=170  Identities=14%  Similarity=0.110  Sum_probs=0.0

Q ss_pred             EEEEEcchhccCCCCCCCcccccccccchHHHHHHHHHHHHhhcCCCceEEEeCCCcCCccHH-----HHHHHHHHHhcC
Q gi|45553317|re  263 TSGTYNDSLLTINSDYDAYLLDFPLMGDDFMLYLARMELKCRFRRHERVLQSGLCVSGLTIN-----GARNRLKRVQLPE  337 (468)
Q Consensus       263 ~Iv~iGDSIT~G~~~~~~~~~~~~~~liGds~~~~La~~~l~~~~~~~~~~V~N~G~~isG~ts~-----~~~~rl~~~l~~~  337 (468)
                        ||+|+|||+|.|               ++.++...+..         ..++.+.|.|  .+|.+..      ++..+++..+...
T Consensus         1 ril~~GDSlt~g-------------~~~~~~~~~~~~~--------~~~~~v~n~~---~g~g~~~~~~~~~~~~~~~~~~~~   58 (200)
T cd01829         1 RVLVIGDSLAQG-------------LAPGLLRALAD--------NPGIRVINRS--KGSSGLVRPDFFDWPEKLKELIAEE   58 (200)
T ss_pred             CEEEEeccHHHH-------------HHHHHHHHHhh--------CCCeEEEEcc--ccccCCcCCCCcccHHHHHHHHhcC


Q ss_pred             -CCeEEEEeeeccCCCCCCH-------------HHHHHHHHHHHHHHhCCCEEEEEcCCCCCccccchhhHHHHHHHHHHH
Q gi|45553317|re  338 -GTQIIVNIGSVDIMRGKPL-------------VQIEHDFRLLIKEMHNMRLVPILTNLAPLGNYCHDKVLCDKIYRFNKF  404 (468)
Q Consensus       338 -PDlVvI~lGtND~~~g~s-------------e~f~~~l~~LI~~lr~~ga~VIL~t~~P~~~~~~~~~~~~~~~i~~~N~~  404 (468)
                   ||+|+|++|+||+......                ++|.++++++++.+++.+++||+++++++|.....    ..+.+..+|+.
T Consensus        59 ~pd~vii~~G~ND~~~~~~~~~~~~~~~~~~~~~~~~~l~~l~~~l~~~~~~vi~~~~p~~~~~~~----~~~~~~~~~~~n~~  134 (200)
T cd01829        59 KPDVVVVFLGANDRQDIRDGDGYLKFGSPEWEEEYRQRIDELLNVARAKGVPVIWVGLPAMRSPK----LSADMVYLNSL  134 (200)
T ss_pred             CCCEEEEEecCcccccccCCCccccCChhHHHHHHHHHHHHHHHHHHHHhCCCCcEEEEeCCCCCchh----HHHHHHHHHHH


Q ss_pred             HHHHhC--CCEEEEChHHHhhhcccccHh----------hcCCCCcCCCCcHHHHHHHHHHHHHHHHHH
Q gi|45553317|re  405 IRSECC--HLKVIDIHSCLINERGVVRFD----------CFQASPRQVTGSKEPYLFWNKIGRQRVLQ  460 (468)
Q Consensus       405 ir~~A~--gv~fIDl~~~~~~~~~~~g~~~~~~~~~----------~~~dDGvHPn~~~~Gy~~lA~~i~~~I~~  460 (468)
                  ++++|+  ++.|||++..|....+.....         ++..||+|||.  +||+.||+.+.+.|++
T Consensus       135 ~~~a~~~~~~~~~vd~~~~~~~~~~~~~~~~~g~~~~~~~~DgvH~t~~~G~~~~a~~~~~~~i~~  200 (200)
T cd01829       135 YREEVAKAGGEFVDVWDGFVDENGRFTYSGTDVNGKKVRLRTNDGIHFTA--AGGRKLAFYVEKLIRR  200 (200)
T ss_pred             HHHHHHHcCCEEEEchHhhcCCCCceeeeccCCCCcEEEEEcCCCCCCH--HHHHHHHHHHHHHHhhC
```

No 57

>**3dc7_A** Putative uncharacterized protein LP_3323; NESG LPR109 X-RAY LP_3323, structural genomics, PSI-2, prote structure initiative; 2.12A {Lactobacillus plantarum} SCOP: **c.23.10.9**

```
Probab=99.86  E-value=3.3e-22  Score=194.92  Aligned_cols=189  Identities=13%  Similarity=0.039  Sum_probs=0.0

Q ss_pred             CCCCCccEEEEEcchhccCCCCCCCCcccccccccchHHHHHHHHHHHHhhcCCCceEEEeCCCcCCccHH----HHHHHHH
Q gi|45553317|re  256 TPTPTILTSGTYNDSLLTINSDYDAYLLDFPLMGDDFMLYLARMELKCRFRRHERVLQSGLCVSGLTIN----GARNRLK  331 (468)
Q Consensus       256 ~~~P~~~~Iv~iGDSIT~G~~~~~~~~~~~~~~liGds~~~~La~~~l~~~~~~~~~~V~N~G~~isG~ts~------~~~~rl~  331 (468)
                    .......+|+|+|||||.|.....               + +|+..|+..+       ..+.+.|.|  ++|.++.    .+..++.
T Consensus        16 ~~~~~~~~~i~~lGDSit~G~g~~~~~----------~~-~~~~~~l~~~l-----------~~~v~N~g--~~G~t~~~~~~~~~~~~~~   77 (232)
T 3dc7_A        16 NGHVSFKRPAWLGDSITANNGLAT----------V-HYHDILAADW------DVERSDNLG--ISGSTIGSRYDAMAVRYQ   77 (232)
T ss_dssp             --CBCCSSEEEEESTTTSTTCSSS----------S-CHHHHHHHHH------TCSCCEEEE--CTTCCSSTTSSCHHHHGG
T ss_pred             cCccccCEEEEEeccccCCCCCCC----------c-cHHHHHHHHc------CcceeeeeC--ccCCeecCChHHHHHH


Q ss_pred             HHHhcCCCCeEEEEEeeeccCCCCCCCHHHHHH---------HHHHHHHHHHhC--CCEEEEEcCCCCCcccc------hhhHH
Q gi|45553317|re  332 RVQLPEGTQIIVNIGSVDIMRGKPLVQIEH---------DFRLLIKEMHNM--RLVPILTNLAPLGNYCH------DKVLC  395 (468)
Q Consensus       332 ~~l~~~~PDlVvI~lGtND~~~g~s~e~f~~~~~~~~~~~~~~~~l~~LI~~lr~~--ga~VIL~t~~P~~~~~~~~~~~~~~~~~~~~~  395 (468)
                     . +..+||+|+|++|+|+|+..+.+.+++..           +++++|+|+.+++.  +++|+|+++++++++.....           .....
T Consensus        78 ~-~~~~pd~vii~~G~ND~~~~~~~~~~~~~~~~~~~~~~~~~~f~~~l~~~i~~~~~~~P~~~~iv~~~p~~~~~~~~~~~~~~~~~~~~~~~  156 (232)
T 3dc7_A        78 A-IPEDADFIAVFGGVNDYGRDQPLGQYGDCDMTTFYGALMMLLTGLQTNWPTVPKLFISAIHIGSDFGGSFSAVTNGLG  156 (232)
T ss_dssp             G-SCTTCSEEEEECCHHHHTTCCCCTTCCSTTSHHHHHHHHHHHHHHHHCTTSCEEEECCCCSCSBTTBCSSCCTTS
T ss_pred             h-cCCCCCEEEEEecccccccCcCCCcccccHHHHHHHHHHHHHHHHHCCCCeEEEEeCCccCccCCcccccccccc


Q ss_pred             HHHHHHHHHHHHHHhC--CCEEEEChHH-HhhhccccHhhcCCCCcCCCCcHHHHHHHHHHHHHHHHHHHH
Q gi|45553317|re  396 DKIYRFNKFIRSECC--HLKVIDIHSC-LINERGVVRFDCFQASPRQVTGSKEPYLFWNKIGRQRVLQVIETS  465 (468)
Q Consensus       396 ~~i~~~N~~ir~~A~--gv~fIDl~~~~~~~~~~~~~~~~~g~~~~~~~~~~dDGvHPn~~~~Gy~~lA~~i~~~I~~~L~~~  465 (468)
                  ..+.+||++++++|+  ++++||++..  ...........+++..||+|||.  +||++||+.|+.+|+.+|.+.+.+.+.+
T Consensus       157 ~~~~~~~~~i~~~a~~~~v~~iD~~~~~~~~~~~~~~~~~~~~~DgiHpn~~~G~~~iA~~i~~~l~~~~~~  227 (232)
T 3dc7_A       157 YRQSDYEAAIAQMTADYGVPHLSLYRDAGMTFAIPAQAAIYSVDTLHPNN--AGHRVIARKLQSFLDSHFLEH  227 (232)
T ss_dssp             CCHHHHHHHHHHHHHHHHTCCEEEHHHHSSCCTTSHHHHHHHBSSSSBCH--HHHHHHHHHHHHHHHHHHCGGG
T ss_pred             hhHHHHHHHHHHHHHHhcCCCcEEechhhcCCcccccchhccCCCCCCcCH--HHHHHHHHHHHHHHHHHHHhh
```

No 58

>**SUPFAM template** c.23.10 SGNH hydrolase (**52266**) SCOP seed sequence: d1fxwf_.

```
Probab=99.86  E-value=4.2e-20  Score=164.64  Aligned_cols=174  Identities=19%  Similarity=0.237  Sum_probs=0.0

Q ss_pred             CCccEEEEEcchhccCCCCCCCCcccccccccchHHHHHHHHHHHHhhcCCCceEEEeCCCcCCccHHHHHHHHHH-HHh-c
Q gi|45553317|re  259 PTILTSGTYNDSLLTINSDYDAYLLDFPLMGDDFMLYLARMELKCRFRRHERVLQSGLCVSGLTINGARNRLKR-VQL-P  336 (468)
Q Consensus       259 P~~~~Iv~iGDSIT~G~~~~~~~~~~~~~~liGds~~~~La~~~l~~~~~~~~~~V~N~G~~isG~ts~~~~~~rl~~~-~l~-~  336 (468)
                    ....+++++|||+|+|.|..            ..+.|...+.          +..+.|.|  ++|.++.++..++.. .+.  .
T Consensus        32 ~~~~~~~~~~GdS~~~~~~~~~--------------~~~~~~~~~~~~~~~~~~~~~~~~~~~n~G~~~~g~~~~~~~~~~~~~~~~~~~~   88 (212)
T SUPFAM0046880   32 EKPLRILFLGDSITAGWG-----------LSGGWRELLA----------GLEVINLG--ISGDTTADLLARLEEDVLDNL   88 (212)
T ss_pred             cCCCcEEEeechhhhhcc-----------ccchHHHHhc----------CCceEeec--ccchHHHHHHHHHhhhhhcc


Q ss_pred             CCCeEEEEeeeccCCCCCCCHHHHHHHHHHHHHHHHhC--CCEEEEEcCCCCCcccchhhHHHHHHHHHHHHHHHHhC---C
Q gi|45553317|re  337 EGTQIIVNIGSVDIMRGKPLVQIEHDFRLLIKEMHNM--RLVPILTNLAPLGNYCHDKVLCDKIYRFNKFIRSECC---H  411 (468)
Q Consensus       337 ~PDlVvI~lGtND~~~g~s~e~f~~~l~~LI~~lr~~~~ga~VIL~t~~P~~~~~~~~~~~~~~~~i~~~N~~ir~~A~~~~g  411 (468)
                  +||+|+|++|+||+.  +.+.+.+.+.+++..+++.+++.  +++|++||++++|......  ...+.+..+|+.++++|+.   +
T Consensus        89 ~p~~v~~~~G~ND~~~~~~~~~~~~~~~~~~~~~~~~~~~~~~~~~p~~~~~~~~~~~~~~~~~~~~~~~~~~~~~~~n~~~~~~~~~~~  164 (212)
T SUPFAM0046880   89 KPDLVVLMLGTNDL--GLSPEEIAENLRALIELIRAKLPGAKILLLGLLPVGERPN--YLNERIKELNELLKELAKELPG  164 (212)
T ss_pred             CCCEEEEeccCCC--CCCHHHHHHHHHHHHHHHHHHhCCCCeEEEEecccccccc--chHHHHHHHHHHHHHHHHHhcCC


Q ss_pred             CEEEEChHHHhhhcccccHhhcCCCCcCCCCcHHHHHHHHHHHHHHHHHH
Q gi|45553317|re  412 LKVIDIHSCLINERGVVRFDCFQASPRQVTGSKEPYLFWNKIGRQRVLQVI  462 (468)
Q Consensus       412 v~fIDl~~~~~~~~~~~g~~~~~~~~~dDGvHPn~~~~Gy~~lA~~i~~~I~~~L  462 (468)
                  +.|+|++..+.+..+.....++.|+ +|||.  .||+.|++.+.+.|.+.+.|
T Consensus       165 ~~~~~~~~~~~~~~~~~~~~~~~~~~~~D~~H~~~~g~~~~~~~~~~~~~~~l~~~L  212 (212)
T SUPFAM0046880  165 VTFVDLYDLLLDEDGTLPELLTADG-LHPNA--AGYRLLAEALLPALLELL  212 (212)
T ss_pred             ceEeehhhhccccccccCCC-CCCCH--HHHHHHHHHHHHHHHHHhC
```

No 59

>**4hyq_A** Phospholipase A1; glycerophospholipid, hydrolase; HET: 1PE; 1.75A {Streptomyces albidoflavus}

```
Probab=99.86  E-value=7e-22  Score=190.33  Aligned_cols=189  Identities=8%  Similarity=-0.097  Sum_probs=0.0

Q ss_pred                CccEEEEEcchhccCCCCCCC--cccccccccchHHHHHHHHHHHHhhcCCCceEEEeCCCcCCccHHHHHHHHHHHHhcC
Q gi|45553317|re  260 TILTSGTYNDSLLTINSDYDA--YLLDFPLMGDDFMLYLARMELKCRFRRHERVLQSGLCVSGLTINGARNRLKRVQLPE  337 (468)
Q Consensus       260 ~~~~Iv~iGDSIT~G~~~~~~--~~~~~~liGds~~~~La~~~l~~~~~~~~~~V~N~G~~isG~ts~~~~~rl~~~l~~~  337 (468)
                     +.++|+++|||||.|......  ........+.+|+..++..+-.      ..+.|.|  ++|.++.++..+....+..+
T Consensus         1 a~~~i~~~GDSit~G~~~~~~~~~~~~~~~~~~~~~~~l~~~l~~-----------~n~g-~~G~~~~~~~~~~~~~~~~
T 4hyq_A           1 AAGGYVALGDSYSSGVGAGSYDSGSGDCRRTPKAYPALWAAANSP------ASFDFVA--CSGAVTSDVLNKQMGPLNSS   72 (236)
T ss_dssp              CCSEEEEEESHHHHTTTSCCCCGGGGGGCCCTTSHHHHHHHHHCC------SEEEECC--CTTCCHHHCCCCCCTTCCTT
T ss_pred              CCccEEEeccchhcCCCCCCCCCCCCCCCCcCcccHHHHHHHhcCC------Ceeeeee--ecCcchhhhhHHHHhhcCCC


Q ss_pred                CCeEEEEeeeccCC-----------------------CCCCHHHHHHHHHHHHHHHHh--CCCEEEEEcCCCCCcccc--
Q gi|45553317|re  338 GTQIIVNIGSVDIM-----------------------RGKPLVQIEHDFRLLIKEMHN--MRLVPILTNLAPLGNYCH--  390 (468)
Q Consensus       338 PDlVvI~lGtND~~-----------------------~g~s~e~f~~~l~~LI~~lr~~~ga~VIL~t~~P~~~~~~~~--  390 (468)
                     ||+|+|++|+||+.                        ...+.++|..+++++|+.+++  .+++|+|++++|+.....
T Consensus        73 ~d~v~i~~G~ND~~~~~~~~~~~~~~~~~~~~~~~~~~~~l~~~i~~i~~~~p~~~i~~~~~p~~~~~~~~c  152 (236)
T 4hyq_A          73 TSLVSLTIGGNDAGFADVMTTCVLQSEANCIARVNTAKAFVESTLPGRLDSVYSQVRAKAPSANVVVLGYPRFYKLNGTC  152 (236)
T ss_dssp              EEEEEEECSTTTTTHHHHHHHHHHSCHHHHHHHHHHHHHHHHHHHTHHHHHHHHHHHHHHHHCTTCEEEEECCCCCBCCCSCC
T ss_pred              CCEEEEEecCccccHHHHHHHHHhcCCccchhhHHHHHHHHHHHHHHHHHHHHHHHHHHHHHHHHHCCCCEEEEeCCCccCCCCCCc


Q ss_pred                --------hhhHHHHHHHHHHHHHHHHHhC--CCEEEECHHHHHhhcccccHhhc-------CCCCcCCCCcHHHHHHHHHH
Q gi|45553317|re  391 --------DKVLCDKIYRFNKFIRSECC--HLKVIDIHSCLINERGVVRFDCF-------QASPRQVTGSKEPYLFWNKI  453 (468)
Q Consensus       391 --------~~~~~~~i~~~N~~ir~~A~--~gv~fIDl~~~~~~~~~g~~~~~~~~~~~~~~~dDGvHPn~~~~Gy~~lA~~  453 (468)
                             .....+.+..+|++++++|+  ++.|||++..|.+........++       ..||+|||.  +||++||+.
T Consensus       153 ~~~~~~~~~~~~~~~~~~~~n~~i~~~a~~~~v~~vD~~~~~~~~~~~~~~~~~~~~~~~~~~~Dg~Hpn~~~~G~~~~A~~  230 (236)
T 4hyq_A         153 VAGLTEGERTAINGAADLLNSVISKRAADHGYAYGDIAAAFTGHEICSGDSWLHSVKWTGINDSYHPTA--AGQSGGYLP  230 (236)
T ss_dssp              TTSCCHHHHHHHHHHHHHHHHHHHHHHHHHHHHTTCEEECHHHHTTTCSBTSSSCSBCCCCSSSSCCTTCBCH--HHCCCCCHH
T ss_pred              CCCCCHHHHHHHHHHHHHHHHHHHHHHHHHHHHHHcCCEEecCccccCCCCCCCCcchhccCcccccCCCCCCCH--HHHHHHHHHH


Q ss_pred                HHHHH
Q gi|45553317|re  454 GRQRV  458 (468)
Q Consensus       454 i~~~I  458 (468)
                     +.+.|
T Consensus       231 i~~~l  235 (236)
T 4hyq_A         231 VLNSK  235 (236)
T ss_dssp              HHHHH
T ss_pred              HHHhh
```

No 60

>1es9A00

```
Probab=99.86  E-value=4.5e-20  Score=180.60  Aligned_cols=175  Identities=18%  Similarity=0.285  Sum_probs=0.0

Q ss_pred                CCCCccEEEEEcchhccCCCCCCCccccccccccchHHHHHHHHHHHHhhcCCCceEEEeCCCcCCccHHHHHHHHHH-HHh
Q gi|45553317|re  257 PTPTILTSGTYNDSLLTINSDYDAYLLDFPLMGDDFMLYLARMELKCRFRRHERVLQSGLCVSGLTINGARNRLKR-VQL  335 (468)
Q Consensus       257 ~~P~~~~Iv~iGDSIT~G~~~~~~~~~~~~~~~~liGds~~~~La~~~l~~~~~~~~~~~~~V~N~G~~isG~ts~~~~~~rl~~-~l~  335 (468)
                     ......+|+|+|||||.+......+...+.                     +..++|.|  ++|+++.+++.+++. .+.
T Consensus        34 ~~~~~~~~~~~~~~~~GdS~~~~~~~~~~~~~~~~~~~~~~~~~~~~~~~~~~~~~~~~~~n~G--~~g~~~~~~~~~~~r~~~~~~~~~~~~   90 (216)
T 1es9A00          34 AKEKEPDVVFLGDSITEGWGDSEVWEELLP----------------------GLKVLNLG--IGGDTTENVLWRLENGVLD   90 (216)
T ss_pred              cCCCCCCEEEEchHHhcCCCCCCChHHHHcC----------------------CCCEEEcc--cCHhhHHHHHHHHHHhhhh


Q ss_pred               -cCCCeEEEEeeeccCCCCCCHHHHHHHHHHHHHHHHHHhC--CCEEEEEcCCCCCccccchhhHHHHHHHHHHHHHHHH-hC-
Q gi|45553317|re  336 -PEGTQIIVNIGSVDIMRGKPLVQIEHDFRLLIKEMHNM--RLVPILTNLAPLGNYCHDKVLCDKIYRFNKFIRSE-CC-  410 (468)
Q Consensus       336 -~~PDlVvI~lGtND~~~g~s~e~f~~~l~~LI~~lr~~~ga~VIL~t~~P~~~~~~~~~~~~~~~~~~i~~~N~~ir~~~A~~-  410 (468)
                      .+|++|+|++|+|+  +.+.+++..++++++.+++.  +++|+++++|......  ...+.+.++|+.+++ +.
T Consensus        91 ~~~p~~~~~~~~~G~Nd~--~~~~~~~~~~~~~~~~~p~~~~p~~~~~~~~~~~~~~~~~~~n~~~~~~~~   166 (216)
T 1es9A00          91 GLKPKVVVLMIGTNDL--GRSAEEIAENLKAIVERIRAKLPGAKILLLSILPVGERPN--ELNEKIQELNELLKELLAKE  166 (216)
T ss_pred              cCCCCEEEEEeeECCC--CCCHHHHHHHHHHHHHHHHHHHCCCCeEEEEEeeccCcCcc--cHHHHHHHHHHHHHHHHHHhcC


Q ss_pred                -CCEEEECHHHHhhhcccccHhhcCCCCcCCCCcHHHHHHHHHHHHHHHHHH
Q gi|45553317|re  411 -HLKVIDIHSCLINERGVVRFDCFQASPRQVTGSKEPYLFWNKIGRQRVLQV  461 (468)
Q Consensus       411 -gv~fIDl~~~~~~~~g~~~~~~~~~~~dDGvHPn~~~~Gy~~lA~~i~~~I~~~  461 (468)
                      ++.|+|++..|.+..+....+++|+|||+.  +||+.|++.+.+.+|.++
T Consensus       167 ~~~~~~~~~~~~~~~~~~~~~~~~~~~~~D~lH~~~--~~gy~~~~~~~~~~~~l~~~  215 (216)
T 1es9A00         167 GVTFLDLNSLLLDEDGKLKSELTP-DGLHLNE--KGYEVWAEALEPALKKL  215 (216)
T ss_pred              CCeEEEEcCHHHhCCCcCcCHHHCC-CCcCcCH--HHHHHHHHHHHHHHHHHHhc
```

No 61

>**cd01844** SGNH_hydrolase_like_6 SGNH_hydrolase subfamily. SGNH hydrolases are a diverse family of lipases and esterases. The tertiary fold of the enzyme is substantially different from that of the alpha/beta hydrolase family and unique among all known hydrolases; its active site closely resembles the Ser-His-Asp(Glu) triad found in other serine hydrolases.

```
Probab=99.86  E-value=1.6e-20  Score=176.81  Aligned_cols=167  Identities=12%  Similarity=-0.011  Sum_probs=0.0

Q ss_pred                EEEEEcchhccCCCCCCCCccccccccccchHHHHHHHHHHHHhhcCCCceEEEeCCCcCCccHHHHHHHHHHHHhcCCCCeEE
Q gi|45553317|re  263 TSGTYNDSLLTINSDYDAYLLDFPLMGDDFMLYLARMELKCRFRRHERVLQSGLCVSGLTINGARNRLKRVQLPEGTQII  342 (468)
Q Consensus       263 ~Iv~iGDSIT~G~~~~~~~~~~~~liGds~~~~La~~~l~~~~~~~~~~~V~N~G~~isG~ts~~~~~rl~~~l~~~PDlVv  342 (468)
                     +|+|+|||||+|.....        -+.+|...++...       ++.++|.|  ++|.+..+.. ..+-....+||+|+
T Consensus         1 ~i~~~GDSiT~G~~~~~-----------~~~~w~~~~~~~~----------v~N~G--~i~G~~~~~~~----~~~~~~pd~vv   62 (177)
T cd01844           1 PWVFYGTSISQGACASR--------PGMAWTAILARRL-------GLEVINLG--FSGNARLEPE-VAELLRDVPADLYI   62 (177)
T ss_pred              CEEEEeChhhCCCCCCC--------CCCChHHHHHHHc-------CCcEEEee--ecCccccchh-HHHHHhcCCCCEEE


Q ss_pred                EEeeeccCCCCCCCHHHHHHHHHHHHHHHHHhC--CCEEEEEcCCCCCCccccchhhHHHHHHHHHHHHHHHHhC--------CC
Q gi|45553317|re  343 VNIGSVDIMRGKPLVQIEHDFRLLIKEMHNM--RLVPILTNLAPLGNYCHDKVLCDKIYRFNKFIRSECC--------HL  412 (468)
Q Consensus       343 I~lGtND~~~g~s~e~f~~~l~~LI~~lr~~~ga~VIL~t~~P~~~~~~~~~~~~~~~~i~~~N~~ir~~A~~--------gv  412 (468)
                     |++|+||....   ++.++++.+|+.+++.  +++|++++++|...........++++++........   ++
T Consensus        63 ~~~GtND~~~~~~~~~~~~~~~~~~~i~~~p~~~~i~~~~~~~~~~~~~~~~~~~~~~~~~~~~~~~~~~~~~~~~~~~v  139 (177)
T cd01844          63 DCGPNIVGAEA---MVRERLGPLVKGLRETHPDTPILLVSPRYCPDAELTPGRGKLTLAVRRALREAFEKLRADGVPNL  139 (177)
T ss_pred              EEccccCCccH---HHHHHHHHHHHHHHHHHCCCCCEEEEeCCCCCCCCCCCCccchHHHHHHHHHHHHHHHHHHHHhcCCCCE


Q ss_pred                EEEEChHHHhhhcccccHhhcCCCCcCCCCcHHHHHHHHHHHHHHHH
```

```
Q gi|45553317|re  413 KVIDIHSCLINERGVVRFDCFQASPRQVTGSKEPYLFWNKIGRQRV  458 (468)
Q Consensus       413 ~fIDl~~~~~~~~g~~~~~~~~dDGvHPn~~~~Gy~~lA~~i~~~I  458 (468)
                      .|+|.+..+...      ..+..||+|||+  +||++||+.+.+.|
T Consensus       140 ~~id~~~~~~~~-----~~~~~DglHPn~--~G~~~~A~~l~~~~  177 (177)
T cd01844        140 YYLDGEELLGPD------GEALVDGIHPTD--LGHMRYADRFEPVL  177 (177)
T ss_pred            EEecchHhcCCc------ccCCCCCCCCH--HHHHHHHHHHhhC
```

No 62

>**pfam14606** Lipase_GDSL_3 GDSL-like Lipase/Acylhydrolase family.
Probab=99.86 E-value=3.5e-20 Score=175.32 Aligned_cols=166 Identities=11% Similarity=-0.004 Sum_probs=0.0

```
Q ss_pred             EEEEEcchhccCCCCCCCcccccccccchHHHHHHHHHHHhhcCCCceEEEeCCCcCCccHHHHHHHHHHHhcCCCeEE
Q gi|45553317|re  263 TSGTYNDSLLTINSDYDAYLLDFPLMGDDFMLYLARMELKCRFRRHERVLQSGLCVSGLTINGARNRLKRVQLPEGTQII  342 (468)
Q Consensus       263 ~Iv~iGDSIT~G~~~~~~~~~~~~~~liGds~~~La~~~l~~~~~~~~~~~V~N~G~~isG~ts~~~~~rl~~~l~~~PDlVv  342 (468)
                      +|+|+|||||.|.....         .+.+|+..++.+        ++.++|.|   ++|.++.+. ...+-+...+||+|+
T Consensus         3 ~i~~~GDSiT~G~~~~~--------~~~~~~~~~~~~~l~~~-------~~~~v~N~G~~i~G~~~~~~-~~~~~~~~~~~~d~v~   64 (178)
T pfam14606        3 PIVFYGTSITQGASASR--------PGMAYTNILGRRL-------GRPVVNLG--FSGNGRMEP-EVADLIAELDARVFV   64 (178)
T ss_pred              EEEEEcChhhCCcCCCC--------CCCCHHHHHHHHh-------CCCEEEec--ccCcCccch-hHHHHHhcCCCcEEE
```

```
Q ss_pred             EEeeeccCCCCCCHHHHHHHHHHHHHHHHhC--CCEEEEEcCCCCCcccchhhHHHHHHHHHHHHHHHhC--------CC
Q gi|45553317|re  343 VNIGSVDIMRGKPLVQIEHDFRLLIKEMHNM--RLVPILTNLAPLGNYCHDKVLCDKIYRFNKFIRSECC--------HL  412 (468)
Q Consensus       343 I~lGtND~~~g~s~e~f~~~l~~LI~~lr~~~ga~VIL~t~~P~~~~~~~~~~~~~~~~~i~~~N~~ir~~A~--------gv  412 (468)
                      |++|+|      .+.++|.+++++||+++++.   +++|++++++|.............+.++|+.++++++         ++
T Consensus        65 i~~G~N-----~~~~~~~~~~~~~~i~~~r~~~~p~~~Ivl~~~~~~~~~~~~~~~~~~~~~~~~n~~~~~~~~~~~~~~~~~~~~v  139 (178)
T pfam14606       65 IDCLPN-----LSVEEIRERMLPFVKRLRDAHPDTPILLVEDPMYTRGDFDPSRGKEVRAKNKALYEAFETLKKEGVKNL  139 (178)
T ss_pred              EECCCC-----CCHHHHHHHHHHHHHHHHHCCCCCEEEEeCCCCcccccCchhhHHHHHHHHHHHHHHHHHhCCCCE
```

```
Q ss_pred             EEEECh HHHhhhcccccHhhcCCCCcCCCCcHHHHHHHHHHHHHHH
Q gi|45553317|re  413 KVIDIHSCLINERGVVRFDCFQASPRQVTGSKEPYLFWNKIGRQRVL  459 (468)
Q Consensus       413 ~fIDl~~~~~~~~g~~~~~~~~dDGvHPn~~~~Gy~~lA~~i~~~I~  459 (468)
                      .|+|.+..+     .....+..||+|||+  +||++||+.+.+.|+
T Consensus       140 ~~id~~~~~~~~-----~~~~~~Dg~Hpn~--~Gy~~~A~~l~~~i~  178 (178)
T pfam14606       140 YYIPGNELI------GDDGEATVDGIHPTD--LGMMRYADAYEPVIR  178 (178)
T ss_pred              EEEccHHhc------CCCcCCCCCCCCCCH--HHHHHHHHHHHHHhC
```

No 63

>**cd01833** XynB_like SGNH_hydrolase subfamily, similar to Ruminococcus flavefaciens XynB. Most likely a secreted hydrolase with xylanase activity. SGNH hydrolases are a diverse family of lipases and esterases. The tertiary fold of the enzyme is substantially different from that of the alpha/beta hydrolase family and unique among all known hydrolases; its active site closely resembles the Ser-His-Asp(Glu) triad found in other serine hydrolases.
Probab=99.85 E-value=1.6e-20 Score=172.77 Aligned_cols=147 Identities=15% Similarity=0.143 Sum_probs=0.0

```
Q ss_pred             cEEEEEcchhccCCCCCCCcccccccccchHHHHHHHHHHHhhcCCCCceEEEeCCCcCCccHHHHHHHH-HHHHhcCCCe
Q gi|45553317|re  262 LTSGTYNDSLLTINSDYDAYLLDFPLMGDDFMLYLARMELKCRFRRHERVLQSGLCVSGLTINGARNRL-KRVQLPEGTQ  340 (468)
Q Consensus       262 ~~Iv~iGDSIT~G~~~~~~~~~~~~~liGds~~~La~~~l~~~~~~~~~~~V~N~G~~isG~ts~~~~~rl~~~l~~~PDl  340 (468)
                      ++|+|+||||+|.|                                   ...|.|  ++|.++.++..++ ......+|+
T Consensus         1 ~~i~~iGDS~t~G~------------------------------------~~~n~g~~~~G~~~~~~~~~~~~~~~~~pd~  43 (157)
T cd01833          1 LRIMPLGDSITWG------------------------------------DKDHEG--HSGYLIDQIAAAADWVLAAKPDV  43 (157)
T ss_pred              CceeecCcccceec------------------------------------CCccCC--CchHhHHHHHHHhhHhhCCCCE
```

```
Q ss_pred             EEEEeeeccCCCCCCHHHHHHHHHHHHHHHHhC--CCEEEEEcCCCCCcccchhhHHHHHHHHHHHHHHHhC-------C
Q gi|45553317|re  341 IIVNIGSVDIMRGKPLVQIEHDFRLLIKEMHNM--RLVPILTNLAPLGNYCHDKVLCDKIYRFNKFIRSECC-------H  411 (468)
Q Consensus       341 VvI~lGtND~~~g~s~e~f~~~l~~LI~~lr~~~ga~VIL~t~~P~~~~~~~~~~~~~~~~~i~~~N~~ir~~A~-------g  411 (468)
                      |++|++|+|+|..+.+.+.+++.+++++|+.+++.   +++|+++++++++.......      ..+.++|+.++++++       +
T Consensus        44 vvi~~G~ND~~~~~~~~~~~~~~~~~~~~~~~v~~~~~~~p~~~ii~~~~~~~~~~~~~~~~~~~~n~~l~~~~~~~~~~~~~~~~~~~~~~~~~~~~~~~+
T cd01833         44 VLLHLGTNDLVLNRDPDTAPDRLRALIDQMRAANPDVKIIVATLIPTTDASG----NARIAEYNAAIPGVVADLRTAGSP  119 (157)
T ss_pred              EEEecccccccccCCHHHHHHHHHHHHHHHHhhCCCCeEEEEeCCCCCchh----hhHHHHHHHHHHHHHHHHhhcCCCCc
```

```
Q ss_pred             CEEEEChHHHhhhcccccHhhcCCCCcCCCCcHHHHHHHHHHHHHHH
Q gi|45553317|re  412 LKVIDIHSCLINERGVVRFDCFQASPRQVTGSKEPYLFWNKIGRQRV  458 (468)
Q Consensus       412 v~fIDl~~~~~~~~g~~~~~~~~dDGvHPn~~~~Gy~~lA~~i~~~I  458 (468)
                      +.|+|++..+..      +++..||+|||+  +||++.||+.+++.|
T Consensus       120 ~~~~d~~~~~~~~~~~~~~~~~Dg~Hp~~~~~Gy~~~a~~~~~~l  157 (157)
T cd01833        120 VVLVDMSTGYTT-------ADDLYDGLHPND--QGYKKMADAWYEAL  157 (157)
T ss_pred              eEEEecccccCCc-------cccCCCCCCCCCH--HHHHHHHHHHHHhC
```

No 64

>**2o14_A** Hypothetical protein YXIM; NESG, X-RAY, SR595, structural genomics, PSI-2, protein structure initiative; 2.10A {Bacillus subtilis} SCOP: **b.18.1.32 c.23.10.8**
Probab=99.85 E-value=2.8e-20 Score=196.23 Aligned_cols=192 Identities=11% Similarity=0.045 Sum_probs=0.0

```
Q ss_pred             CCCCCCccEEEEEcchhccCCCCCCCcccccccccchHHHHHHHHHHHhhcCCC-ceEEEeCCCcCCccHHHHHH--HHHH
Q gi|45553317|re  256 TPTPTILTSGTYNDSLLTINSDYDAYLLDFPLMGDDFMLYLARMELKCRFRRH-ERVLQSGLCVSGLTINGARN--RLKR  332 (468)
Q Consensus       256 ~~~P~~~~Iv~iGDSIT~G~~~~~~~~~~~~~liGds~~~La~~~l~~~~~~~~~-~~V~N~G~~isG~ts~~~~~--rl~~  332 (468)
                      ...+...+|+++||||||.|..........       +|...|+.      .+...+.+.|.|  ++|.++.+++.  +++.
T Consensus       157 ~~p~~~~~Iv~lGDSit~G~~~~~~~~~~~------~~w~~~L~~~~~~~~~~l~~~~~v~N~g~~~G~ts~~~~~~~~~~~l~~  223 (375)
T 2o14_A          157 DQPVTNRTIYVGGDSTVCNYYPLNSSKQA------GWGQMLPH-----YIDKHTFQVRNMA--SGGQIARGFRNDGQLEA  223 (375)
T ss_dssp             SSCCCCCEEEEEECTTTSCCSSTTTCSBC------CHHHHGGG-----TSCTTTCEEEECC--CTTCCHHHHHHSSHHHH
T ss_pred             cCCCCCceEEEecccccccCCCCccCCCCC------CHHHHHHH-----HhcccCceEEEec--CCCCcchhhhhhcchHHH
```

```
Q ss_pred             HHh-cCC-CeEEEEeeeccCCCC--CCHHHHHHHHHHHHHHHHHhCCCEEEEEcCCCCCCc-ccchhhHHHHHHHHHHHHH
Q gi|45553317|re  333 VQL-PEG-TQIIVNIGSVDIMRG--KPLVQIEHDFRLLIKEMHNMRLVPILTNLAPLGN-YCHDKVLCDKIYRFNKFIRS  407 (468)
Q Consensus       333 ~l~~~P-DlVvI~lGtND~~g--~s~e~f~~~l~~LI~~lr~~ga~VIL~t~~P~~~~~~~~~~~~~~~~~i~~~N~~ir~  407 (468)
                      ++. .+| |+|++|+|+|.+.  .+.++|.++|++++++++.+|+++++.   +.+........+++||||+++
T Consensus       224 ~l~~~~p~d~VvI~~G~ND~~~~~~~~~~~~~~l~~li~~lr~~~a~vvlvtP~~~~~~~~~~~~~~~~~~~~~~~~~~~~~i~~
T 2o14_A          224 ILKYIKPGDYFMLQLGINDTNPKHKESEAEFKEVMRDMIRQVKAKGADVILSTPQGRATDFTSEGIHSSVNRWYRASILA  303 (375)
T ss_dssp             HHTTCCTTCEEEEECCTGGGCGGGCCCHHHHHHHHHHHHHHHTTTCEEEEECCCCCTTCBCTTSCBCTTSTTHHHHHH
T ss_pred             HHHhCCCCCEEEEEeecCCccCCCCCcCCHHHHHHHHHHHHHHHHHHHHHCCCEEEEECCCCCCCCCcchhhHHHHHHHHHH
```

```
Q ss_pred              HhC--CCEEEECHHHHhhhcccccHh-----hcCCCCcCCCCcHHHHHHHHHHHHHHHHHHH
Q gi|45553317|re   408 ECC--HLKVIDIHSCLINERGVVRFD-----CFQASPRQVTGSKEPYLFWNKIGRQRVLQVI  462 (468)
Q Consensus        408 ~A~--gv~fIDl~~~~~~~~g~~~~~-----~~~dDGvHPn~~~~Gy~~lA~~i~~~I~~~L  462 (468)
                       +|+  ++.|||++..+.+.......+     ++..||+|||.  +||++||+.|.+.|.+++
T Consensus        304 ~A~~~~v~~vDl~~~~~~~~~~~~g~~~~~~~~~~~~~DgvHpn~--~G~~~iA~~l~~~L~~~~  363 (375)
T 2o14_A           304 LAEEEKTYLIDLNVLSSAYFTSIGPERTLGLYMDGDTLHPNR--AGADALARLAVQELKRQG  363 (375)
T ss_dssp              HHHHTTCEEECHHHHHHHHHHHHCHHHHHTTBCTTCSSSBBH--HHHHHHHHHHHHHHHHTT
T ss_pred              HHHHcCCcEEecHHHHHHHHHhcCcccccceecCCCCCCCCH--HHHHHHHHHHHHHHHHhc
```

No 65

>1esc_A Esterase; 2.10A {Streptomyces scabiei} SCOP: c.23.10.1 PDB: 1esd _A 1ese _A
Probab=99.84 E-value=1.9e-20 Score=189.76 Aligned_cols=197 Identities=8% Similarity=-0.027 Sum_probs=0.0

```
Q ss_pred              CCCCccEEEEEcchhccCCCCCCC-----cccccccccchHHHHHHHHHHHhhcCCCc---eEEEeCCCcCCccHHHHHH
Q gi|45553317|re   257 PTPTILTSGTYNDSLLTINSDYDA-----YLLDFPLMGDDFMLYLARMELKCRFRRHE---RVLQSGLCVSGLTINGARN  328 (468)
Q Consensus        257 ~~P~~~~Iv~iGDSIT~G~~~~~~-----~~~~~~~~liGds~~~~La~~~l~~~~~~~~~---~V~N~G~~isG~ts~~~~~  328 (468)
                       ..+...+|+++|||||.|......     ........+.+|...|++.+-..    ++   .+.|.|  ++|.++.++..
T Consensus          1 ~p~~~~~iv~lGDSit~G~g~~~~~~~~~~~~~~~~~~~~~~~~~~~~l~~~l~~~----g~~~~~~~n~g--~sG~t~~~~~~   74 (306)
T 1esc_A             1 APADPVPTVFFGDSYTANFGIAPVTNQDSERGWCFQAKENYPAVATRSLADK----GITLDVQADVS--CGGALIHHFWE   74 (306)
T ss_dssp              ---CEEEEEEECCSHHHHTTTCSSBTTTTSGGGGGTCBTTCHHHHHHHHHHTT----TCEEEEEEECC--CTTCCGGGGTS
T ss_pred              CCCCCCcEEEECccccCCCCCCCCcCCCCCCCCCCCcccHHHHHHHHHHhc----CCCcceeeEee--cCCCchhcccc


Q ss_pred              -------------HHHHHHhcCCCeEEEEeeeccCCCCCCH---------------------------------------
Q gi|45553317|re   329 -------------RLKRVQLPEGTQIIVNIGSVDIMRGKPL---------------------------------------  356 (468)
Q Consensus        329 -------------rl~~~l~~~PDlVvI~lGtND~~~g~s~---------------------------------------  356 (468)
                                   ++. .+..+||+|+|++|+||+......
T Consensus         75 ~~~~~~~~~~~~~~~~~~-~~~~~~~~dlV~i~~G~ND~~~~~~~~~~~~~c~~~~~~~~~~~~p~~p~~~~~~~~~~c~~~~~~~~~~~~~~~~  153 (306)
T 1esc_A            75 KQELPFGAGELPPQQD-ALKQDTQLTVGSLGGNTLGFNRILKQCSDELRKPSLLPGDPVDGDEPAAKCGEFFGTGDGKQW  153 (306)
T ss_dssp              CEECGGGCCEECCGGG-GCCTTCCEEEECCCHHHHTHHHHHHHTCTTTTSSCSSCCCCSSTTSCGGGHHHHTTTSHHHHH
T ss_pred              cccCCccccCHHHHH-hcCCCCCEEEEEecCccHhHHHHHHHHHhcccccccccccccccCCcchhhhcccccchhH


Q ss_pred              -----HHHHHHHHHHHHHHHhC--CCEEEEEcCCCCCccc----------------------chhhHHHHHHHHHHHHHH
Q gi|45553317|re   357 -----VQIEHDFRLLIKEMHNM--RLVPILTNLAPLGNYC----------------------HDKVLCDKIYRFNKFIRS  407 (468)
Q Consensus        357 -----e~f~~~l~~LI~~lr~~~~~ga~VIL~t~~P~~~~~~~~~~~~~~~~~~~~~~~~~~~~~~~~~~~~~~~~~~~i~~~N~~ir~  407 (468)
                            ++|..+|.++|+.++++   +++|+|++++++....                       ........+.+||+++++
T Consensus        154 ~~~~~~~~~~~~~~l~~~~i~~ir~~~~p~a~Iv~~~~~p~~~~~~~~~~~~~~~~c~~~~~g~~~~~~~~~~~~~~~~~~~~~~~~~~~~~~~~n~~i~~  233 (306)
T 1esc_A           154 LDDQFERVGAELEELLDRIGYFAPDAKRVLVGYPRLVPEDTTKCLTAAPGQTQLPFADIPQDALPVLDQIQKRLNDAMKK  233 (306)
T ss_dssp              HHHHHHHHHHHHHHHHHHHHHHHHHSTTCEEEEECCCCCSCSCGGGGGSCCTTCSSCTTTTCCTTTHHHHHHHHHHHHHHHHH
T ss_pred              HHHHHHHHHHHHHHHHHHHHHHHHHCCCCeEEEecCccccCCCCCcccccccccCCcccccchhHHHHHHHHHHHHHHHHHH


Q ss_pred              HhC--CCEEEECHHHHhhhcccccH-hhcC-------CC--------CcCCCCcHHHHHHHHHHHHHHHHHHHH
Q gi|45553317|re   408 ECC--HLKVIDIHSCLINERGVVRF-DCFQ-------AS--------PRQVTGSKEPYLFWNKIGRQRVLQVI  462 (468)
Q Consensus        408 ~A~--gv~fIDl~~~~~~~~g~~~~-~~~~~~~~~~~~~~~dD--------GvHPn~~~~Gy~~lA~~i~~~I~~~L  462 (468)
                       +|+  ++.|||++..|.+...+... .|+.         .|         ++|||.  +||++||+.|.+.|.+++
T Consensus        234 ~a~~~~v~~vD~~~~~~~~~~~~~~~~~~~~~~~~~~~~~~~~~~~~~~D~~g~~~~~~~~HPn~--~G~~~iA~~l~~~l~~~l  304 (306)
T 1esc_A           234 AAADGGADFVDLYAGTGANTACDGADRGIGGLLEDSQLELLGTKIPWYAHPND--KGRDIQAKQVADKIEEIL  304 (306)
T ss_dssp              HHHTTTCEEECTGGGCTTSSTTSTTSCSBCCSSSEEEEESSSCEEECSSCBCH--HHHHHHHHHHHHHHHHHHT
T ss_pred              HHHHcCCEEEeCccccCCCCCCCCCCcceecccCCcccccccccCCcCCCH--HHHHHHHHHHHHHHHHHHHh
```

No 66

>4nrd_A Uncharacterized protein; lipase_GDSL_LKE protein, PF16255 family, structural genomics center for structural genomics, JCSG; 2.10A {Bacteroides ovatus}
Probab=99.83 E-value=1.4e-20 Score=183.82 Aligned_cols=175 Identities=14% Similarity=0.153 Sum_probs=0.0

```
Q ss_pred              cCCCCCCcccccccccchhhcccccccccccccCCcCCCCCCccEEEEEcchhccCCCCCCCcc------------cc
Q gi|45553317|re   217 HQPSCRTQSQKTEPTENRHLGIFVHPFNGMNIMKRRHEMTPTPTILTSGTYNDSLLTINSDYDAYL------------LD  284 (468)
Q Consensus        217 ~~p~~~~~~~~~~~~~~~~~~~~~~~~~~g~~~~~~~~~~~e~~~~P~~~~Iv~iGDSIT~G~~~~~~~~~~~--------------~~  284 (468)
                       +||..+|..+.                                  ....+|+||||||.|.+...+..                .+
T Consensus          9 ~~~~~~~~~~~~~~~~-------------------------------------------------------~~~~~~i~~lGDSiT~G~G~~~~~~~~~~~~~yp~~~~~~~~~~  57 (227)
T 4nrd_A             9 HHHHENLYFQG-----------------------------------QSLKSISILGDSYSTFEGYLQPDTNSIWYYVSPRQQTD  57 (227)
T ss_dssp              -----------------------------------------------CCCCEEEEEESGGGCCTTCCSSTTSCCSCCSSCSSSCS
T ss_pred              cccccccCCc----------------------------------cCCCeEEEEecccccCCcccCCCCcccccccccccccC


Q ss_pred              ccccchHHHHHHHHHHHhhcCCCce-EEEeCCCcCCccHHHH------------HHHHHHHHhcCCCeEEEEeeeccCC
Q gi|45553317|re   285 FPLMGDDFMLYLARMELKCRFRRHER-VLQSGLCVSGLTINGA------------RNRLKRVQLPEGTQIIVNIGSVDIM  351 (468)
Q Consensus        285 ~~liGds~~~~La~~~l~~~~~~~~~~~~~~~~~V~N~G~~isG~ts~~~~~~~~~~~~~~-------------~~rl~~~l~~~PDlVvI~lGtND~~~  351 (468)
                       ..-....|+..++.        ..+..  ++|.|  ++|.++.+.              ..++..+  .+||+|+|++|+||+|+.
T Consensus         58 ~~~~~~~~~~~~~~~l~~~~~~~~~~~~~~~--------g~~~v~N~g~~~~G~t~~~~~~~~~~~~~~~~~~~~~~~rl~~~~~~~~~~pd~vvi~~G~ND~~  126 (227)
T 4nrd_A            58 VTSVKQTWWHKFIK-------ENNYRLCVNNS--FSGATICNTGYNQADYSDRSFITRMDKL--GCPDIIFIFGATNDCW  126 (227)
T ss_dssp              CCCGGGSHHHHHHH-------HTTCEEEEEEE--CTTCCSSSBCGGGCBCTTTCHHHHTTCS--CSCSEEEEECCHHHHH
T ss_pred              cCccccCCHHHHHH-------HcCcchheeee--ecceeEEeccccCCcccccHHHHHhcC--CCCCEEEEEeecCCCC


Q ss_pred              CCCC-------------HHHHHHHHHHHHHHHHHhC--CCEEEEEcCCCCCcccchhhHHHHHHHHHHHHHHHHhC--CCEE
Q gi|45553317|re   352 RGKP-------------LVQIEHDFRLLIKEMHNM--RLVPILTNLAPLGNYCHDKVLCDKIYRFNKFIRSECC--HLKV  414 (468)
Q Consensus        352 ~g~s-------------e~f~~~l~~LI~~lr~~~~~ga~VIL~t~~P~~~~~~~~~~~~~~~~~~~~~~i~~~N~~ir~~~A~~~gv~f  414 (468)
                       .+.+                .++|..+|.++|+.+++.   +++||++++      +.+..||+++++|+  ++.|
T Consensus        127 ~~~~~~~~~~~~~~~~~~~~~~~~~~~~l~~li~~i~~~~~p~~ii~~~p~----------------------------n~~~~a~~~~~~  194 (227)
T 4nrd_A           127 AGSPLGDYKYEGWTKEDLYTFRPAMAYLLDHMIDRYPNVEIYFLLNS------------GLKEEFNESVRAICNHYNIDC  194 (227)
T ss_dssp              HTCCCCCCCCSCCCTTGGGSHHHHHHHHHHHHHHHHSTTCEEEEECT------------TCCHHHHHHHHHHHHHHHTTCEE
T ss_pred              cccCCcccccccccccchhhHHHHHHHHHHHHHHHHHHHHHCCCCeEEEEcCc------------HHHHHHHHHHHHHHHHcCCcE


Q ss_pred              EEChHHHhhhcccccHhhcCCCCcCCCCcHHHHHHHHHHHHHHHHHH
Q gi|45553317|re   415 IDIHSCLINERGVVRFDCFQASPRQVTGSKEPYLFWNKIGRQRVLQ  460 (468)
Q Consensus        415 IDl~~~~~~~~g~~~~~~~~~~~dDGvHPn~~~~Gy~~lA~~i~~~I~~  460 (468)
                       ||++..                 ..||+|||.  +||++||+.|.+.|.+
T Consensus        195 vD~~~~~~~~~~~~~~~~~~~~Dg~Hpn~~~~G~~~~~A~~l~~~~l~~  225 (227)
T 4nrd_A           195 IELHDI-------------DKKSGHPSI--KGMEQISEQIKMFMRK  225 (227)
T ss_dssp              EECCSC-------------CEETTEECH--HHHHHHHHHHHHHHHHHH
T ss_pred              EeCCCC-------------CCCCCCCCH--HHHHHHHHHHHHHHhh
```

No 67

>PF13472 Lipase_GDSL_2: GDSL-like Lipase/Acylhydrolase family; PDB: 1ES9 _A 1WAB _A 3DT9 _A 1BWQ _A 1FXW _A 3DT6 _A 1BWR _A 3DT8 _A 1BWP _A 2WAA _A ....
Probab=99.83 E-value=2.2e-19 Score=165.76 Aligned_cols=166 Identities=22% Similarity=0.245 Sum_probs=0.0

```
Q ss_pred                    EEEcchhccCCCCCCcccccccccchHHHHHHHHHHHhhcCCCceEEEeCCCcCCcccHHHHHHHHHHH----HhcCCCe
Q gi|45553317|re  265 GTYNDSLLTINSDYDAYLLDFPLMGDDFMLYLARMELKCRFRRHERVLQSGLCVSGLTINGARNRLKRV----QLPEGTQ  340 (468)
Q Consensus       265 v~iGDSIT~G~~~~~~~~~~~~~~liGds~~~~La~~~l~~~~~~~~~~V~N~G~~isG~ts~~~~~~rl~~~-----l~~~PDl  340 (468)
                         ||+|||+|.|....          .+.+|+..|+..     ...++.+.|.|  ++|.++.++..++...     ...+||+
T Consensus         1 v~~GDS~t~g~~~~----------~~~~~~~~~l~~~~~----------~~~n~~~--~~G~~~~~~~~~~~~~~~~~~~~~~~d~   64 (179)
T PF13472_consen    1 VFLGDSITAGYGAP---------NNGSYPDRLAER-----PGRGIEVYNLG--VSGATSSDFLARLQRDVLRFKDPKPDL   64 (179)
T ss_dssp                    EEEESHHHHTTTTS---------SCTSHHHHHHHH-----HTCCEEEEEEE---TT-BHHHHHHHHHHHCHHHCGTTCSE
T ss_pred                    CEEcccccCCCCC---------CCCCHHHHHHHh-----hCCCcEEEEEe--ecCcccHhHHHHHHHHHHhhhccCCCCE
```

```
Q ss_pred                    EEEEeeeccCCCC----CCHHHHHHHHHHHHHHHHHhCCCEEEEEcCCCCCccccch---hhHHHHHHHHHHHHHHHhC--C
Q gi|45553317|re  341 IIVNIGSVDIMRG----KPLVQIEHDFRLLIKEMHNMRLVPILTNLAPLGNYCHD---KVLCDKIYRFNKFIRSECC--H  411 (468)
Q Consensus       341 VvI~lGtND~~~g----~s~e~f~~~l~~LI~~lr~~ga~VIL~t~~P~~~~~~~~~~~-------~~~~~i~~~N~~ir~~A~--g  411 (468)
                         |||++|+||...+    .+.+++..+++++|+.++..+ +|++++++|.......    ......+..+|++++++|+  +
T Consensus        65 vvi~~G~ND~~~~~~~~~~~~~~~~~l~~~i~~~~~~~~-vi~~~~~~~~~~~~~~~~~~~~~~~~~~~~~~~~~~~~~~~~~~~a~~~~~  143 (179)
T PF13472_consen   65 VVISFGTNDVLNGDENDTSPEQYEQNLRRIIEQLRPHG-PVILVSPPPRGPDPRDPKQDYLNRRIDRYNQAIRELAKKYG  143 (179)
T ss_dssp                    EEEE--HHHHCTCTTCHHHHHHHHHHHHHHHHHHHHTTS-EEEEEE-SCSSSSTTTTHTTCHHHHHHHHHHHHHHHHHHHHHCT
T ss_pred                    EEEEcccccccccccccccHHHHHHHHHHHHHHhhcccC-cEEEecCCCCcccccccccchhhhhhHHHHHHHHHHHHHHHHHHcC
```

```
Q ss_pred                    CEEEECHHHHhhhcccccHhhcCCCCcCCCCcHHHHHHH
Q gi|45553317|re  412 LKVIDIHSCLINERGVVRFDCFQASPRQVTGSKEPYLFW  450 (468)
Q Consensus       412 v~fIDl~~~~~~~~~g~~~~~~~~~~dDGvHPn~~~~Gy~~l  450 (468)
                         +.|||++..|.+........++.|+ +|||.  +||++|
T Consensus       144 ~~~id~~~~~~~~~~~~~~~~~~~~D~~Hp~~~~G~~~~~  179 (179)
T PF13472_consen  144 VPFIDLFDAFDDHDGWFPKYYFSDG-VHPNP--AGHQLI  179 (179)
T ss_dssp                    EEEEEHHHHHBTTTSCBHTCTBTTS-SSBBH--HHHHHH
T ss_pred                    CEEEECHHHHcccccccchhhcCCCC-CCcCH--HHhCcC
```

No 68

>template ISOAMYL ACETATE-HYDROLYZING ESTERASE 1.
Probab=99.82 E-value=2.7e-20 Score=188.51 Aligned_cols=176 Identities=15% Similarity=0.175 Sum_probs=0.0

```
Q ss_pred                    EEEEEcchhccCCCCCCCccccccccchHHHHHHHHHHHHhhcCCCceEEEeCCCcCCccHHHHHHHHHHHHHhcC-----
Q gi|45553317|re  263 TSGTYNDSLLTINSDYDAYLLDFPLMGDDFMLYLARMELKCRFRRHERVLQSGLCVSGLTINGARNRLKRVQLPE-----  337 (468)
Q Consensus       263 ~Iv~iGDSIT~G~~~~~~~~~~~~~~liGds~~~~La~~~l~~~~~~~~~~V~N~G~~isG~ts~~~~~~rl~~~l~~~-----  337 (468)
                         +|+.||||||+.....++|..                .|...|-++.+|+|+|  +||++++.++.-|.+++...
T Consensus        26 ~~~LFGDSItQfsF~~gGWGa----------------~Lad~~~RK~DV~nRG--fSGYNTRWAl~~Lpr~~~~~~~~a~~   87 (277)
T PTHR14209       26 RIVLFGDSITQFSFQDGGWGA----------------ALADKLVRKADVVNRG--FSGYNTRWALKVLPRIFPKAAGAAD   87 (277)
T ss_pred                    hHHhhhHHHHHHhccccccHHH----------------HHHHHHHHHHHhHhccc--cchhHHHHHHHHHHHhhhcCCcccccc
```

```
Q ss_pred                    CCeEEEEEeeeccCC--------CCCCHHHHHHHHHHHHHHHHhC---CCEEEEEcCCCCCc-------------ccchhh
Q gi|45553317|re  338 GTQIIVNIGSVDIM--------RGKPLVQIEHDFRLLIKEMHNM---RLVPILTNLAPLGN-------------YCHDKV  393 (468)
Q Consensus       338 PDlVvI~lGtND~~~~--------~g~s~e~f~~~l~~LI~~lr~~~~~~ga~VIL~t~~P~~~~~~~~~~~~~~~~~~~~~~~~~  393 (468)
                         |-.|+|.+|+||+.          ..+++++|.+||++|+..++..   ..+|||+|++|+.+                ......
T Consensus        88 pvavTvFFGANDa~L~~~~~n~~QHVPLdEY~~NLr~iv~~lks~~~~~~riIlITPPP~~E~~~~~~~~~~~e~~~g~~~~~R  167 (277)
T PTHR14209       88 PVAVTVFFGANDAALPEDENPKQHVPLDEYKDNLRAIVAYLKSHGWPKTRIILITPPPVDEAAWEKECYGEILKGCKLNR  167 (277)
T ss_pred                    hhhhhhcccchHhhhccccccCCCCCChHHHHHHHHHHHHHHHHHccCCccceecCCCCccCHHHHHHHhhhhcccCcccc
```

```
Q ss_pred                    HHHHHHHHHHHHHHHHhC--CCEEEECHHHHhhhcccccHhhcCCCCcCCCCcHHHHHHHHHHHHHHHHHHH
Q gi|45553317|re  394 LCDKIYRFNKFIRSECC--HLKVIDIHSCLINERGVVRFDCFQASPRQVTGSKEPYLFWNKIGRQRVLQ  460 (468)
Q Consensus       394 ~~~~i~~~N~~ir~~A~--gv~fIDl~~~~~~~~~~g~~~~~~~~~~dDGvHPn~~~~Gy~~lA~~i~~~I~~  460 (468)
                         .++....|.++..++|+  ++.+||+|+++.|....++. +.+-.||+|+|+  +||+++.+.|.+.|.+
T Consensus       168 ~N~~~g~YA~Ac~~vA~e~g~~v~DLw~amQ~~~~~~~~~~~~L~DGLHLs~~~~~G~~~~l~~e~~~~~l~~  232 (277)
T PTHR14209      168 TNEVAGEYAKACVEVAQECGVDVIDLWTAMQKDGGWF--SSLLSDGLHLSP--KGNEFLFEELVPVLEK  232 (277)
T ss_pred                    ccHhHHHHHHHHHHHHHHHHcCCCCcchhhHHhhhccccH--HHhccccccCH--HHHHHHHHHHHHHHHHH
```

No 69

>4m8k_A Hypothetical protein, GDSL-like lipase/acylhydrol protein; GDSL-like lipase/acylhydrolase family, PF13472 family, struc genomics; 1.90A {Bacteroides uniformis}
Probab=99.82 E-value=6.8e-21 Score=186.87 Aligned_cols=177 Identities=15% Similarity=0.112 Sum_probs=0.0

```
Q ss_pred                    CcCCCCCCccEEEEEcchhccCCCCC-CCcccccccc------------cchHHHHHHHHHHHhhcCCCceE-EEeCCCc
Q gi|45553317|re  253 HEMTPTPTILTSGTYNDSLLTINSDY-DAYLLDFPLM------------GDDFMLYLARMELKCRFRRHERV-LQSGLCV  318 (468)
Q Consensus       253 ~e~~~~P~~~~Iv~iGDSIT~G~~~~~-~~~~~~~~~li------------Gds~~~~La~~~l~~~~~~~~~V~-N~G~~i  318 (468)
                         .+..+.....+|+|+||||.|.+..  .....++.-.                ...|+..+..        ..++.+ .|.|  +
T Consensus        12 ~~~~~~~~~~~i~~lGDSiT~G~g~~~~~~~~~~~~~~~~~~~~~~~~l~~~l~~~~~~~~~w~~~~~~~~~~~~~~~g~~~~N~g~~~   82 (236)
T 4m8k_A           12 HENLYFQGQKKVSILGDSYSTFYGHVSPAANLCWYGVPGEKKENDVTKVEETWWYRFIH-------EHGFQLERNNS--Y   82 (236)
T ss_dssp                    -CCHHHHCCCEEEEEECGGGCCTTSSSSTTSCCSSSCTTCCCSSSCCSGGGSHHHHHHH-------HTTCEEEEEEE--C
T ss_pred                    ccCccccCCCceEEEEeCccccCCCccCCcccceechhhcccccCCCCcccccchHHHHHH-------hCCcceEEecc--c
```

```
Q ss_pred                    CCccHHHH------------HHHHHHHHhcCCCeEEEEeeeccCCCCCC------------HHHHHHHHHHHHHHHHHhC
Q gi|45553317|re  319 SGLTINGA------------RNRLKRVQLPEGTQIIVNIGSVDIMRGKP------------LVQIEHDFRLLIKEMHNM  373 (468)
Q Consensus       319 sG~ts~~~~~~~~~~~~~~~~~~~rl~~~l~~~PDlVvI~lGtND~~~g~s~~~~~~~~~~~~~~~~~e~f~~~l~~LI~~lr~~  373 (468)
                         +|.++.+.                ..++...  .+||+|+|++|+|+..+.+              .++|.++++++|+.+++.
T Consensus        83 ~G~t~~~~~~~~~~~~~~~~~~~l~~l~~~~pd~Vvi~~G~ND~~~~~~~~~~~~~~~~~~~~~~~~~~~~~~l~~~i~~l~~~  160 (236)
T 4m8k_A           83 SGSTVCHTGYEKADYSDRSFITRIHNL--GTPDIILVFGGTNDSWAGAPIGAYQYDGWTKADLYSFRPAFCYLLASLKQL  160 (236)
T ss_dssp                    TTCCSSSBCGGGCBCTTTCGGGSTTCS--CSCSEEEEECCHHHHHHTCCCCCCCSCCCTTGGGSHHHHHHHHHHHHHHH
T ss_pred                    cCcccccccccccccccchhhhhhcc--CCCCEEEEEeecCCCcccCCCCCCcccchhhhccHHHHHHHHHHHHHHHHHHh
```

```
Q ss_pred                    --CCEEEEEcCCCCCccccchhhHHHHHHHHHHHHHHHhC--CCEEEECHHHHhhhcccccHhhcCCCCcCCCCcHHHHHH
Q gi|45553317|re  374 --RLVPILTNLAPLGNYCHDKVLCDKIYRFNKFIRSECC--HLKVIDIHSCLINERGVVRFDCFQASPRQVTGSKEPYLF  449 (468)
Q Consensus       374 --ga~VIL~t~~P~~~~~~~~~~~~~~~~i~~~N~~ir~~A~--gv~fIDl~~~~~~~~~g~~~~~~~~~~dDGvHPn~~~~Gy~~  449 (468)
                            +++||+++++             ..+..+|++++++|+  ++.|||++..                ..||+|||.  +||++
T Consensus       161 ~p~~~iil~~~~~~~~~~~~~~~~~~~~~~~~~~~~~~~n~~~~~~~~~~~~~~~~~~~a~~~~~~~~~~~~~~~~~~~~~~vD~~~~~~~~~~~~~~~~~~~~~~~~~~DgiHpn~~~~G~~~  213 (236)
```

```
T 4m8k_A          161 YPAARIYNITNS------------ELSEEVTDSMDEICRHYGIENIRLHDI-------------DKQWGHPSV--QGMQS  213 (236)
T ss_dssp             CTTSEEEEEECT------------TCCHHHHHHHHHHHHHHTCEEEECCSC-------------CEETTEECH--HHHHH
T ss_pred             CCCCEEEEEeCh------------HHHHHHHHHHHHHHHHcCCcEEECccC-------------cccCCCCCH--HHHHH


Q ss_pred             HHHHHHHHHHHHHHHHhc
Q gi|45553317|re  450 WNKIGRQRVLQVIETSLE  467 (468)
Q Consensus       450 lA~~i~~~I~~~L~~~~a  467 (468)
                      ||+.+.+.|.+++++.+.
T Consensus       214 ~A~~l~~~l~~~l~~~~~  231 (236)
T 4m8k_A          214 IDAQVWESVSPILEASVS  231 (236)
T ss_dssp             HHHHHHHHHTTC------
T ss_pred             HHHHHHHHHHHHHHHhcC
```

No 70

>**4lhs_A** Uncharacterized protein; two domains protein, N-terminus GXDLY domain (PF14607 family terminus lipase GDSL 3 (PF14606 family); HET: MSE; 1.40A {Bacteroides ovatus}
Probab=99.81 E-value=3.8e-19 Score=185.32 Aligned_cols=179 Identities=11% Similarity=-0.002 Sum_probs=0.0

```
Q ss_pred             cCCCCCCccEEEEEcchhccCCCCCCCcccccccccchHHHHHHHHHHHhhcCCCCceEEEeCCCcCCccHHHHHHHHHH
Q gi|45553317|re  254 EMTPTPTILTSGTYNDSLLTINSDYDAYLLDFPLMGDDFMLYLARMELKCRFRRHERVLQSGLCVSGLTINGARNRLKRV  333 (468)
Q Consensus       254 e~~~~P~~~~Iv~iGDSIT~G~~~~~~~~~~~~~~liGds~~~La~~~l~~~~~~~~~~~V~N~G~~isG~ts~~~~~rl~~~  333 (468)
                      ++.+.|...+|+|+|||||.|.....         .+.+|...+++.+        ++.++|.|   ++|.++.+  .++.+.
T Consensus       149 ~~~~~~~~~~~~iv~iGDSit~G~~~~~~-----------------~~la~~l---------~~~~v~N~g--~sG~~~~~--------  209 (343)
T 4lhs_A          149 RKGENPFKEKIVVYGSSILQGASASR--------PGMAYPARLSRSS-------GYNFINLG--LSGNGKME--KEVAEM  209 (343)
T ss_dssp             EECCCCCEEEEEEEECHHHHTTTCSS--------GGGSHHHHHHHHH-------SEEEEEEE--CTTTCSCC--HHHHHH
T ss_pred             ccCCCCCCCEEEEEecccccCCCCCC--------CcchHHHHHHHHHc-------CCCEEEEe--cCCccccc--HHHHHH


Q ss_pred             Hh-cCCCeEEEEeeeccCCCCCCHHHHHHHHHHHHHHHHhC--CCEEEEEcCCCCC----cccchhhHHHHHHHHHHHHH
Q gi|45553317|re  334 QL-PEGTQIIVNIGSVDIMRGKPLVQIEHDFRLLIKEMHNM--RLVPILTNLAPLG----NYCHDKVLCDKIYRFNKFIR  406 (468)
Q Consensus       334 l~-~~PDlVvI~lGtND~~~g~s~e~f~~~l~~LI~~lr~~~~ga~VIL~t~~P~~~----~~~~~~~~~~~~~i~~~N~~ir  406 (468)
                      +. .+||+|+|++|+||     +.++|.++++.+|+.+++.  +++||++++++..    ..............++++++
T Consensus       210 l~~~~pd~Vvi~~G~ND----~~~~~~~~~l~~li~~lr~~~P~~~ivl~~~~~~~~~~~~~~~~~~~~~~~~~~~~~~~~~
T 4lhs_A          210 LADIDADAFILDCIPNP-----SPKEITDRTVDFVMTLRQKHPDTPIIVIQTLIRETGNFNQKARENVKRQNEAIAEQVE  284 (343)
T ss_dssp             HHTSCCSEEEEECCSSS-----CHHHHHHHHHHHHHHHHHHHCSSSCEEEECCCCGGGGTCHHHHHHHHHHHHHHHHHHH
T ss_pred             hhcCCCCEEEEEeeCCC-----CHHHHHHHHHHHHHHHHHHHhCCCCCEEEEcCCCCCccccCchHHHHHHHHHHHHHHHH


Q ss_pred             HHhC----CCEEEEChHHHhhhcccccHhhcCCCCcCCCCcHHHHHHHHHHHHHHHHHHHHH
Q gi|45553317|re  407 SECC----HLKVIDIHSCLINERGVVRFDCFQASPRQVTGSKEPYLFWNKIGRQRVLQVIET  464 (468)
Q Consensus       407 ~~A~----gv~fIDl~~~~~~~~~~~g~~~~~~~~~~~dDGvHPn~~~~Gy~~lA~~i~~~I~~~L~~  464 (468)
                      ++++    ++.|+|++..+.+..       .+..||+|||.  +||++||+.+.+.+.|.+++++
T Consensus       285 ~~a~~g~~~v~~vD~~~~~~~~~~~----------~~~DgvHPn~~~G~~~iA~~l~~~l~~~l~~  338 (343)
T 4lhs_A          285 VLRKKNVKNLYFIKEDQFLGTDH------EGTVDGTHPND--LGFDRMLKKYKPAISKILKI  338 (343)
T ss_dssp             HHHHTTCSSEEEECCCCTTCSSS------CSBSSSSBCH--HHHHHHHHHHHHHHHHHHHCC
T ss_pred             HHHhCCCCCeEEeccHHhcCCCc------cCCCCCCCCH--HHHHHHHHHHHHHHHHHHhCc
```

No 71

>**2vpt_A** Lipolytic enzyme; esterase, hydrolase; 1.40A {Clostridium thermocellum}
Probab=99.81 E-value=3.5e-19 Score=172.79 Aligned_cols=179 Identities=11% Similarity=0.097 Sum_probs=0.0

```
Q ss_pred             CccEEEEEcchhccCCCCCCCcccccccccchHHHHHHHHHHHhh------------cCCCceEEEeCCCcCCccHHHHH
Q gi|45553317|re  260 TILTSGTYNDSLLTINSDYDAYLLDFPLMGDDFMLYLARMELKCR------------FRRHERVLQSGLCVSGLTINGAR  327 (468)
Q Consensus       260 ~~~~Iv~iGDSIT~G~~~~~~~~~~~~~~liGds~~~La~~~l~~~~~~~~~~~~~~~~~~~~~V~N~G~~isG~ts~~~~~  327 (468)
                      ..+||+++|||||.|......         +|...|..++....            ........|.|  ++|.++.++.
T Consensus         4 ~~lrIl~iGDSiT~G~~~~~~~~~~~~~~~~~~~~~l~~~l~~~~~~~~~~~vg~~~~g~~~~~~~~~n~g~~~~G~~~~~~~~~  72 (215)
T 2vpt_A            4 KTIKIMPVGDSCTEGMGGGEMG---------SYRTELYRLLTQAGLSIDFVGSQRSGPSSLPDKDHEG--HSGWTIPQIA   72 (215)
T ss_dssp             CEEEEEEEESHHHHTCSSSTTC---------TTHHHHHHHHHHTTCEEEECCSEECCCTTCSCCEEEE--CTTCCHHHHH
T ss_pred             CceEEEEEchhhccCcCCCCCC---------chHHHHHHHHHhcCCeeEEEecccCCCCCcccCCcccc--ccccHhHHH


Q ss_pred             HHHHH-HHhcCCCeEEEEeeeccCCCCCCHHHHHHHHHHHHHHHHhC--CCEEEEEcCCCCCccccchhhHHHHHHHHHHH
Q gi|45553317|re  328 NRLKR-VQLPEGTQIIVNIGSVDIMRGKPLVQIEHDFRLLIKEMHNM--RLVPILTNLAPLGNYCHDKVLCDKIYRFNKF  404 (468)
Q Consensus       328 ~rl~~~~l~~~PDlVvI~lGtND~~~g~s~e~f~~~l~~LI~~lr~~~~ga~VIL~t~~P~~~~~~~~~~~~~~~~~i~~~N~~  404 (468)
                      .++..  ....+||+|+|++|+||+|+..+.+...  ++|+.+|++++++.  ++.|++++.+.....           .++|+.
T Consensus        73 ~~~~~~~~~~~~~~pD~vii~~G~ND~~~~~~~~~~~~~~~li~~~~~~p~~~~ii~~~~~~~~~~~~~~~~~~~~~~~~~~~~~n~~  141 (215)
T 2vpt_A           73 SNINNWLNTHNPDVVFLWIGGNDLLLNGNLNA--TGLSNLIDQIFTVKPNVTLFVADYYPWPEAI---------KQYNAV  141 (215)
T ss_dssp             HHHHHHHHHHCCSEEEEECCHHHHHHHCCCCH--HHHHHHHHHHHHHCTTCEEEEECCCSCSGGG---------HHHHTT
T ss_pred             HHHHHHHhcCCCCEEEEeCCccccccCCCCChHH--HHHHHHHHHHHHHhCCCCeEEEEeeCCchHHH---------HHHHHH


Q ss_pred             HHHHhC-------CCEEEEChHHHhhhcccccHhhcCCCCcCCCCcHHHHHHHHHHHHHHHHHHHHHHHHHHhc
Q gi|45553317|re  405 IRSECC-------HLKVIDIHSCLINERGVVRFDCFQASPRQVTGSKEPYLFWNKIGRQRVLQVIETSLE  467 (468)
Q Consensus       405 ir~~A~-------gv~fIDl~~~~~~~~~~~g~~~~~~~~~~~dDGvHPn~~~~Gy~~lA~~i~~~I~~~L~~~~a  467 (468)
                      +.++++       ++.++|++..+....      +.+..||+|||.  +||++||+.+.++++|.++|+...+
T Consensus       142 ~~~~~~~~~~~~~~~~~~~~~~~~~~~~~~v~~vd~~~~~~~~~~~~~~~~~~~~DgvHpn~~~~G~~~iA~~~~~~~~~l~~~~~~~~~  204 (215)
T 2vpt_A          142 IPGIVQQKANAGKKVYFVKLSEIQFDRN-----TDISWDGLHLSE--IGYKKIANIWYKYTIDILRALAG  204 (215)
T ss_dssp             HHHHHHHHHHTTCCEEEECGGGSCCCHH-----HHBCTTSSSBCH--HHHHHHHHHHHHHHHHHHHHHHHHHHHC
T ss_pred             HHHHHHHHhcCCCcEEEEeccCChhcCCCc-----ccCCCCCCCCH--HHHHHHHHHHHHHHHHHHHhccC
```

No 72

>**cd01831** Endoglucanase_E_like Endoglucanase E-like members of the SGNH hydrolase family; Endoglucanase E catalyzes the endohydrolysis of 1,4-beta-glucosidic linkages in cellulose, lichenin and cereal beta-D-glucans.
Probab=99.81 E-value=1.7e-18 Score=162.67 Aligned_cols=160 Identities=14% Similarity=0.043 Sum_probs=0.0

```
Q ss_pred             EEEEEcchhccCCCCCCC----ccccccccchHHHHHHHHHHHhhcCCCCceEEEeCCCcCCccHHHHHHHHHHHHhcCC
Q gi|45553317|re  263 TSGTYNDSLLTINSDYDA----YLLDFPLMGDDFMLYLARMELKCRFRRHERVLQSGLCVSGLTINGARNRLKRVQLPEG  338 (468)
Q Consensus       263 ~Iv~iGDSIT~G~~~~~~~~~~~~~~liGds~~~La~~~l~~~~~~~~~~~V~N~G~~isG~ts~~~~~rl~~~l~~~P  338 (468)
                      ||+|+|||||.|.+....    ......-...+|+..+++++        +..+.+.|  ++|.+                |
T Consensus         1 ~i~~iGDSit~G~g~~~~~~~~~~~~~~~~~~~~~~~~~~~~~~~~~~~la~~l~~~~~~~~~~~~~~~~~G~g~~~~~~~~p   56 (169)
T cd01831          1 KIEFIGDSITCGYGVTGKSRCDFSAATEDPSLSYAALLARAL-------NAEYSIIA--YSGIG---------------P   56 (169)
T ss_pred             CEEEEeccccccCCCCCCCCccccchhhhhHHHHHHHHHh-------CCceEEEe--ccCCC---------------C
```

```
Q ss_pred              CeEEEEeeeccCCCCC--CHHHHHHHHHHHHHHHHhC--CCEEEEEcCCCCCccccchhhHHHHHHHHHHHHHHhC-CCE
Q gi|45553317|re  339 TQIIVNIGSVDIMRGK--PLVQIEHDFRLLIKEMHNM--RLVPILTNLAPLGNYCHDKVLCDKIYRFNKFIRSECC-HLK  413 (468)
Q Consensus       339 DlVvI~lGtND~~~g~--s~e~f~~~l~~LI~~lr~~--ga~VIL~t~~P~~~~~~~~~~~~~~~~~i~~~N~~ir~~A~-gv~  413 (468)
                       |+|||++|+||.....  +.++|..+++++|+.+++.  ++.|+++.+........   .+..+.+++.+++... ++.
T Consensus        57 d~vvi~~GtND~~~~~~~~~~~~~~~~~~~~~~~i~~lr~~~p~~~ii~~~~p~~~~~~~~~~~---~~~~~~~i~~~~~~~~~~~~~~~~v~  133 (169)
T cd01831         57 DLVVINLGTNDFSTGNNPPGEDFTNAYVEFIEELRKRYPDAPIVLMLGPMLFGPYGT---EEEIKRVAEAFKDQKSKKVH  133 (169)
T ss_pred              CEEEEECCcccccCCCCCHHHHHHHHHHHHHHHHHHHCCCCeEEEEeCCCcCCccch---HHHHHHHHHHHhcCCCceEE


Q ss_pred              EEEChHHHhhhcccccHhhcCCCCcCCCCcHHHHHHHHHHHHHHHHH
Q gi|45553317|re  414 VIDIHSCLINERGVVRFDCFQASPRQVTGSKEPYLFWNKIGRQRVL  459 (468)
Q Consensus       414 fIDl~~~~~~~~~g~~~~~~~~~dDGvHPn~~~~Gy~~lA~~i~~~I~  459 (468)
                       |+|+...+.        .....|++|||.  +||++||+.+.+.|+
T Consensus       134 ~vd~~~~~~~--------~~~~~~~D~~HPn~--~G~~~iA~~l~~~I~  169 (169)
T cd01831        134 YFDTPGILQ--------HNDIGCDWHPTV--AGHQKIAKHLLPAIK  169 (169)
T ss_pred              EEecCCcccc--------cCCcCCCCCcH--HHHHHHHHHHHHhC
```

No 73

>lcl|consensus
Probab=99.80 E-value=2.1e-18 Score=163.68 Aligned_cols=181 Identities=14% Similarity=0.105 Sum_probs=0.0

```
Q ss_pred              CCccEEEEEcchhccCCCCCCCcccccccccchHHHHHHHHHHHhhcCCCCceEEEeCCCcCCccHHHHHHHHHHHHhcC-
Q gi|45553317|re  259 PTILTSGTYNDSLLTINSDYDAYLLDFPLMGDDFMLYLARMELKCRFRRHERVLQSGLCVSGLTINGARNRLKRVQLPE-  337 (468)
Q Consensus       259 P~~~~Iv~iGDSIT~G~~~~~~~~~~~~~~~~liGds~~~~La~~~l~~~~~~~~~~~~V~N~G~~isG~ts~~~~~~rl~~~~l~~~-  337 (468)
                       |...+|+.||||||+-+...++|..                   .|...|.++.+|+..|   ++|++++.++..+.+++...
T Consensus         4 ~~rp~i~LFGdSItq~sF~~gGwGA----------------~Lad~y~RkaDv~~RG--ysGynSRwALkvL~~ifp~~~  65 (245)
T lcl|consensus    4 PMRPRIVLFGDSITQFSFTDGGWGA----------------ALADLYSRKADVLLRG--YSGYNSRWALKVLPEIFPKGS  65 (245)
T ss_pred              cccccEEEecchhhhcccCCchhH----------------HHHHHHHHhhhhhcc--cccchhHHHHHHhhhhccccc


Q ss_pred              ---CCeEEEEEeeeccCCC----C----CCHHHHHHHHHHHHHHHHhC--CCEEEEEcCCCCCc-----------ccchhh
Q gi|45553317|re  338 ---GTQIIVNIGSVDIMR----G----KPLVQIEHDFRLLIKEMHNM--RLVPILTNLAPLGN-----------YCHDKV  393 (468)
Q Consensus       338 ---PDlVvI~lGtND~~~----g----s~e~f~~~l~~LI~~lr~~--ga~VIL~t~~P~~~~----------------~~~~~~  393 (468)
                          |-+|+|.+|+||+..     +    ++.++|.+|+++|+.-++..   ..+||+++++|++.              ......
T Consensus        66 s~~p~lvtVffGaNDs~l~~~~~~~~~~~hvPl~Ey~dNlr~iv~~lks~~~~~~riIlitPpp~de~~~~~~~~~~e~~~~~~~~~~R  145 (245)
T lcl|consensus   66 SIQPVLVTVFFGANDSCLPEPSSLGQHVPLEEYKDNLRKIVSHLKSLSPETRIILITPPPVDEEAWEKQEQEPYVLGPER  145 (245)
T ss_pred              cCCceEEEEEecCccccCCCCCCCCccCHHHHHHHHHHHHHHhhccCCcceEEEecCCCcCHHHHHHhccchhcchh


Q ss_pred              HHHHHHHHHHHHHHHHHhC--CCEEEEChHHHhhhcccccHhhcCCCCcCCCCcHHHHHHHHHHHHHHHHHHH
Q gi|45553317|re  394 LCDKIYRFNKFIRSECC--HLKVIDIHSCLINERGVVRFDCFQASPRQVTGSKEPYLFWNKIGRQRVLQV  461 (468)
Q Consensus       394 ~~~~i~~~N~~ir~~A~--gv~fIDl~~~~~~~~g~~~~~~~~~dDGvHPn~~~~Gy~~lA~~i~~~I~~~~  461 (468)
                       .++....|+++..++|+  ++.+||++|+.|..  .......+.||+|++.  +||+++.+.|.+.|.+.
T Consensus       146 tNe~~~~Ya~ac~~la~e~~l~~vdlws~~Q~~~~~dw~~~~ltDGLHlS~~~~G~~ivf~Ei~kvl~ea  211 (245)
T lcl|consensus  146 TNETVGTYAKACANLAQEIGLYVVDLWSKMQE--SDDWQTSCLTDGLHLSP--KGNKIVFDEILKVLKEA  211 (245)
T ss_pred              hhhHHHHHHHHHHHHHHHHhCCeeeHHhhhh--cccHHHHhcccceeecc--ccchhHHHHHHHHHHhc
```

No 74

>3skv_A SSFX3; jelly roll, GDSL/SGNH fold, alpha/beta hydrolase fold, trans; 2.49A {Streptomyces SP}
Probab=99.80 E-value=1.5e-18 Score=183.73 Aligned_cols=188 Identities=11% Similarity=0.023 Sum_probs=0.0

```
Q ss_pred              cCCCCCCccEEEEEcchhccCCCCCCCcccccccccchHHHHHHHHHHHhhcCCCCceEEEeCCCcCCccHHHHHHHHHHH
Q gi|45553317|re  254 EMTPTPTILTSGTYNDSLLTINSDYDAYLLDFPLMGDDFMLYLARMELKCRFRRHERVLQSGLCVSGLTINGARNRLKRV  333 (468)
Q Consensus       254 e~~~~P~~~~Iv~iGDSIT~G~~~~~~~~~~~~~~~~liGds~~~~La~~~l~~~~~~~~~~~~V~N~G~~isG~ts~~~~~~rl~~~  333 (468)
                       ++.+.+ ..+|+|+|||||.|.....        .+.+|...+++.+      ++.+.|.|  ++|++........+.+
T Consensus       179 ~~~~~~~~~~Iv~iGDSiT~G~~~~~~~~~~~~~~~~~w~~~la~~l~~~~~~~~~~~~v~N~g~~sG~~~~~~~~~~~~~~l  240 (385)
T 3skv_A         179 EKDTSS-KPHWIHYGDSICHGRGAAS--------PSRTWLALAARAE-------GLDLQSLS--FAADGSHLQPMFARLI  240 (385)
T ss_dssp              ECCCCC-CCEEEEEECSSCTTTCSS--------GGGSHHHHHHHHH-------TCEEEEEC--CTGGGSCCHHHHHHH
T ss_pred              ccCCCC-CcEEEEECchhhCCCCCCC--------CCCCHHHHHHHHhC-------CCcEEEee--ccCccccCHhHHHHH


Q ss_pred              HhcCCCeEEEEeeeccCCCCCCCHHHHHHHHHHHHHHHHhC--CCEEEEEcCCCCCcccch-----hhHHHHHHHHHHHHH
Q gi|45553317|re  334 QLPEGTQIIVNIGSVDIMRGKPLVQIEHDFRLLIKEMHNM--RLVPILTNLAPLGNYCHD-----KVLCDKIYRFNKFIR  406 (468)
Q Consensus       334 l~~~PDlVvI~lGtND~~~g~s~e~f~~~l~~LI~~lr~~--ga~VIL~t~~P~~~~~~~~~~~~~~~~~~~~~i~~~N~~ir  406 (468)
                       ...+||+|+|++|+||+..+.+.+.++|.++|++|+.+++.   +++||++++++....      ....+....++++++
T Consensus       241 ~~~pdlVvI~lG~ND~~~~~~~~~~~~~~l~~li~~lr~~~P~a~Ivl~~~~~~p~~~~~~~~~~~~~~~~~~~~~~~l~~~~~~~  320 (385)
T 3skv_A         241 RDLPADLISLRVGTSNFMDGDGFVDFPANLVGFVQIIRERHPLTPIVLGSSVYSPFWDELPADDKPTVADYREQVVKVAE  320 (385)
T ss_dssp              HHSCCSEEEEEESHHHHTTTCCTTTHHHHHHHHHHHHHHTTCSSSCEEEEECCCCTTTTSCCTTSCCHHHHHHHHHHHHH
T ss_pred              hcCCCCEEEEEecccccccCCHHHHHHHHHHHHHHHHHCCCCcEEEEeCCCCcccccCCCccccchHHHHHHHHHHHH


Q ss_pred              HHhC----CCEEEEChHHHhhc-cccHhhcCCCCcCCCCcHHHHHHHHHHHHHHHHHH
Q gi|45553317|re  407 SECC----HLKVIDIHSCLINER-GVVRFDCFQASPRQVTGSKEPYLFWNKIGRQRVLQV  461 (468)
Q Consensus       407 ~~A~----gv~fIDl~~~~~~~-g~~~~~~~~~dDGvHPn~~~~Gy~~lA~~i~~~I~~~~  461 (468)
                       ++++    ++.|||++..+.+.. .....+++..||+|||.  +||++||+.+.+.|.+.
T Consensus       321 ~~a~~g~~~v~~iD~~~~~~~~~~~~~~~~~~~~~DgvHPn~~~~G~~~iA~~l~~~l~~~  378 (385)
T 3skv_A         321 LLRKHGDQNVHYLDGMRVWGPERGMELYLEKPDKYPTHPNA--VGHEIFAESSRREMAAL  378 (385)
T ss_dssp              HHHHTTCTTEEEECHHHHSCTTCCGGGBCSCTTSCCCSBCH--HHHHHHHHHHHHHHHHT
T ss_pred              HHHhcCCCCEEEEcchHhcCcccccccccccCCCccCCCCCH--HHHHHHHHHHHHHHHhh
```

No 75

>cd00229 SGNH_hydrolase SGNH_hydrolase, or GDSL_hydrolase, is a diverse family of lipases and esterases. The tertiary fold of the enzyme is substantially different from that of the alpha/beta hydrolase family and unique among all known hydrolases; its active site closely resembles the typical Ser-His-Asp(Glu) triad from other serine hydrolases, but may lack the carboxlic acid.
Probab=99.80 E-value=5.9e-18 Score=154.91 Aligned_cols=173 Identities=17% Similarity=0.150 Sum_probs=0.0

```
Q ss_pred              EEEEcchhccCCCCCCCcccccccccchHHHHHHHHHHHhhcCCCCceEEEeCCCcCCccHHHHHHHH---HHHHhcCCCCe
Q gi|45553317|re  264 SGTYNDSLLTINSDYDAYLLDFPLMGDDFMLYLARMELKCRFRRHERVLQSGLCVSGLTINGARNRL---KRVQLPEGTQ  340 (468)
Q Consensus       264 Iv~iGDSIT~G~~~~~~~~~~~~~~~~liGds~~~~La~~~l~~~~~~~~~~~~V~N~G~~isG~ts~~~~~~rl~~~~~l~~~PDl  340 (468)
                       |+++|||+|.|..............              ............+.|.|  ++|.++.+....+  ......+||+
T Consensus         1 i~~iGDS~~~g~~~~~~~~~~~~~~~~~~~~~~~~~~~~~~~~~~~~~n~~~~~~g~~~~~~~~~~~~~~~~~~~~~~~~~~  68 (187)
T cd00229          1 ILVIGDSITAGYGASSGSTFYSLLL----------YLLLLAGGPGVEVINLG--VSGATTADALRRLGLRLALLKDKPDL  68 (187)
```

```
T ss_pred               CeeeechhhhccCCCCCCchHHHHH----------HHHhcCCCCceEEEecc--cccHHHHHHHHhhhhhhhhhccCCCE


Q ss_pred               EEEEeeeccCCCC--CCHHHHHHHHHHHHHHHH--hCCCEEEEEcCCCCCccccchhhHHHHHHHHHHHHHHHhC--C---
Q gi|45553317|re    341 IIVNIGSVDIMRG--KPLVQIEHDFRLLIKEMH--NMRLVPILTNLAPLGNYCHDKVLCDKIYRFNKFIRSECC--H---   411 (468)
Q Consensus        341 VvI~lGtND~~~g--~s~e~f~~~l~~LI~~lr--~~ga~VIL~t~~P~~~~~~~~~~~~~~~~i~~~N~~ir~~A~~-g---   411 (468)
                        |+|++|+||+...  .+.+++.+.++++++.++  ..+++|++++++|......  ........+|+.++++++  +
T Consensus         69 vvi~~G~ND~~~~~~~~~~~~~~~~~~~i~~~~~~~~~~~~v~~~~~~~~~~~~~~--~~~~~~~~~~~~~~~~~~~~~~~~~~~   146 (187)
T cd00229          69 VIIELGTNDLGRGGDTSIDEFKANLEELLDALRERAPGAKVILITPPPPPPREG--LLGRALPRYNEAIKAVAAENPAPS   146 (187)
T ss_pred               EEEEecccccccccCCHHHHHHHHHHHHHHHHHHhCCCCeEEEEeCCCCCcchh--hhhhHHHHHHHHHHHHHHhcCCcC


Q ss_pred               -CEEEEChHHHhhhcccccHhhcCCCCcCCCCcHHHHHHHHHHHHH
Q gi|45553317|re    412 -LKVIDIHSCLINERGVVRFDCFQASPRQVTGSKEPYLFWNKIGRQ  456 (468)
Q Consensus        412 -v~fIDl~~~~~~~~~g~~~~~~~~~~dDGvHPn~~~~Gy~~lA~~i~~  456 (468)
                         +.|+|++..+...    ...++..|++|||.  .||+.||+.+.+
T Consensus        147 ~~~~id~~~~~~~~~~----~~~~~~~D~~Hp~~--~~G~~~~a~~i~~  186 (187)
T cd00229         147 GVDLVDLAALLGDE----DKSLYSPDGIHPNP--AGHKLIAEALAS  186 (187)
T ss_pred               ceEEEEHHHhhccc----chhhccccCCcCH--HHHHHHHHHHHh
```

No 76

>**pfam13472** Lipase_GDSL_2 GDSL-like Lipase/Acylhydrolase family. This family of presumed lipases and related enzymes are similar to pfam00657.

Probab=99.79 E-value=5.8e-18 Score=154.20 Aligned_cols=166 Identities=17% Similarity=0.202 Sum_probs=0.0

```
Q ss_pred               EEEcchhccCCCCCCCccccccccchHHHHHHHHHHHHhhcCCCceEEEeCCCcCCccHHHHHHHHHHH--hcCCCeEE
Q gi|45553317|re    265 GTYNDSLLTINSDYDAYLLDFPLMGDDFMLYLARMELKCRFRRHERVLQSGLCVSGLTINGARNRLKRVQ--LPEGTQII   342 (468)
Q Consensus        265 v~iGDSIT~G~~~~~~~~~~~~~~liGds~~~~La~~~l~~~~~~~~~~~V~N~G~~isG~ts~~~~~~rl~~~l--~~~~PDlVv   342 (468)
                        +++|||+|.|....           .+.+|...++..      ....+.+.|.|   ++|.++..+..++...+   ..+||+|+
T Consensus          1 ~~iGDS~~~g~~~~~---------~~~~~~~~~~~l~~~~~-----~~~~~~~~n~~~--~~g~~~~~~~~~~~~~~~~~~~~~~~~~~~~d~iv    64 (173)
T pfam13472          1 VFLGDSITAGYGDS---------RGLGWPGRLAAR-----LGAGVEVVNLG--VSGATTADGLARLEAEVLAAFKPDLVV    64 (173)
T ss_pred               CccccccccCCC---------CCCchHHHHHh-----cCCCceEEEcc--ccCccHHHHHHHhhcccccCCCCEEE


Q ss_pred               EEeeeccCCCC---CCHHHHHHHHHHHHHHHHHhCCCEEEEEcCCCCCccccchhhHHHHHHHHHHHHHHHhC--CCEEEEC
Q gi|45553317|re    343 VNIGSVDIMRG---KPLVQIEHDFRLLIKEMHNMRLVPILTNLAPLGNYCHDKVLCDKIYRFNKFIRSECC--HLKVIDI   417 (468)
Q Consensus        343 I~lGtND~~~g---~s~e~f~~~l~~LI~~lr~~ga~VIL~t~~P~~~~~~~~~~~~~~~~~~i~~~~N~~ir~~A~~-gv~fIDl   417 (468)
                        |++|+||+...   .+.++|.+.+.+.++++.+.+.+|++++++|.......  ........+|+++++++|+  ++.+||+
T Consensus         65 i~~G~ND~~~~~~~~~~~~~~~~~~~i~~~~~~~~~~~~i~~~~~~~~~~~~~~~~~~~~~-~~~~~~~~~~~~~~~~~~~~a~~~~~~~~~~id~   143 (173)
T pfam13472         65 LSLGTNDLGAGRLDASLEAFEAALEAILDAARPAAAPVLVVGPPPVLPEASL-LLNARLAALNEALREVAARYGVPYVDL   143 (173)
T ss_pred               EEeeccccccccccCCHHHHHHHHHHHHHHHHHHhcCCCEEEEeCCCccccchH-HHHHHHHHHHHHHHHHHHhcCCcEEcc


Q ss_pred               hHHHhhhcccccHhhcCCCCcCCCCcHHHHHHH
Q gi|45553317|re    418 HSCLINERGVVRFDCFQASPRQVTGSKEPYLFW  450 (468)
Q Consensus        418 ~~~~~~~~~~g~~~~~~~~~~dDGvHPn~~~~Gy~~l  450 (468)
                        +..+.........  .++..|++|||.  +||++|
T Consensus        144 ~~~~~~~~~~~~~~~~~-~~~~~D~~Hp~~--~~G~~~~  173 (173)
T pfam13472        144 YAALRDHEGWLK-DLLAPDGIHPNA--AGYALI  173 (173)
T ss_pred               hHHhhcccccc-cccccccCcCh--hhhccC
```

No 77

>**SUPFAM template** c.23.10 SGNH hydrolase (**52266**) SCOP seed sequence: d1deoa_.

Probab=99.79 E-value=4e-17 Score=147.35 Aligned_cols=187 Identities=16% Similarity=0.124 Sum_probs=0.0

```
Q ss_pred               cEEEEEcchhccCCCCCCCccccccccchHHHHHHHHHHHHhhcCCCceEEEeCCCcCCccHHHHHH--HHHHHHhc--C
Q gi|45553317|re    262 LTSGTYNDSLLTINSDYDAYLLDFPLMGDDFMLYLARMELKCRFRRHERVLQSGLCVSGLTINGARN--RLKRVQLP--E   337 (468)
Q Consensus        262 ~~Iv~iGDSIT~G~~~~~~~~~~~~~~~~liGds~~~~La~~~l~~~~~~~~~~~V~N~G~~isG~ts~~~~~~~~--rl~~~l~~~~~~-   337 (468)
                        .+|+++|||++.+........         .|...|..         .-++.+.|.|  ++|.++.+...    +++.++..   +
T Consensus          1 ~~~~~~~~GDS~~~~~~~~~~~~~~~~~~~~~~~~~~~~~W~~~l~~~~~~~~~~~~~~~~~~N~a~~~~G~~~~~~~~~~~~~~~~~~~~~~~~   63 (233)
T SUPFAM0036388      1 KRIVLLGDSLTAGYGLGSGGT--------GWGDLLAS-------YLGVEVVNLA--ISGATTRSFLAEGRLDAVLDLLKK   63 (233)
T ss_pred               CceeEEcCCCCCCCCCCcccc--------cHHHHHHh-------hcCCceeeec--cCCcchhhHHHHHHHHHHHhhccC


Q ss_pred               CCeEEEEeeeccC-------------------------CCCCCHHHHHHHHHHHHHHHHhCCCEEEEEcCCCCCccccchh
Q gi|45553317|re    338 GTQIIVNIGSVDI-------------------------MRGKPLVQIEHDFRLLIKEMHNMRLVPILTNLAPLGNYCHDK   392 (468)
Q Consensus        338 PDlVvI~lGtND~-------------------------~~g~s~e~f~~~l~~LI~~lr~~ga~VIL~t~~P~~~~~~~~~~~~~   392 (468)
                        +|+|+|.+|.||.                         ..+.+.+++..+++.+++.+++.++++++++..+.....  .
T Consensus         64 ~~~v~~~~~G~ND~~~~~~~~~~~~~~~~~~~~~~~~~~~~~~~~~~~~~~~~~~~~~~~~~~~~~~~~~~l~~~~~~~~~~~~~~~~~l~~~~~~~~~~~~~~~~~-~   142 (233)
T SUPFAM0036388     64 PDLVLIEFGHNDAKLLGLDNLRLLLPGLGKELLVLVLNLRGVPVDEFKANLEAIIDRLRAAGAKVVLLTPLPRLNFGS-G   142 (233)
T ss_pred               CCEEEEEeccCCccccccccccceeeecccCcceeeeeeccccCCCHHHHHHHHHHHHHHHHhcCceEEEEEeeeccccCc-c


Q ss_pred               hHHHHHHHHHHHHHHHHhC--CCEEEEChHHHhhh----cccccHhhcCCCCcCCCCcHHHHHHHHHHHHHHHHHHHHHHHHh
Q gi|45553317|re    393 VLCDKIYRFNKFIRSECC--HLKVIDIHSCLINE----RGVVRFDCFQASPRQVTGSKEPYLFWNKIGRQRVLQVIETSL   466 (468)
Q Consensus        393 ~~~~~i~~~~N~~ir~~A~~-gv~fIDl~~~~~~~~~~~-~~g~~~~~~~~~~dDGvHPn~~~~Gy~~lA~~i~~~I~~~L~~~~~   466 (468)
                        ........++++++++|+  ++.++|++..+..    .......++..|++|||+  .|++.+|+.|.+.+.+.|.+.+.+.+..
T Consensus        143 ~~~~~~~~~~~~~~~~~~~~~~~~~~A~~~~~~~~~~~~~d~~~~~~~~~~~~~~~~~~~~~~~~~~~~~~~~~~~~~~~~~~~D~~H~~~~~-~G~~~~~~~~~~~~~~~~~~~~~~   220 (233)
T SUPFAM0036388    143 TLRRYLEAYNEAARELAEELGVPLVDLFALLAELLNDLGPAGLPDYFLADGLHLNP--KGARLLAELVAKALKELLLPLA   220 (233)
T ss_pred               hhhhHHHHHHHHHHHHHHhcCCceEeehHHHHHHhhccCCcccccccCCCCCH--HHHHHHHHHHHHHHHHhhhhhh


Q ss_pred               cC
Q gi|45553317|re    467 EY   468 (468)
Q Consensus        467 a~   468 (468)
                        ++
T Consensus        221 ~~   222 (233)
T SUPFAM0036388    221 KL   222 (233)
T ss_pred               hh
```

No 78

>**cd01823** SEST_like SEST_like. A family of secreted SGNH-hydrolases similar to Streptomyces scabies esterase (SEST), a causal agent of the potato scab disease, which hydrolyzes a specific ester bond in suberin, a plant lipid. The tertiary fold of this enzyme is substantially different from that of the alpha/beta hydrolase family and

unique among all known hydrolases; its active site closely resembles two of the three components of typical Ser-His-Asp(Glu) triad from other serine hydrolases, but may lack the carboxylic acid.

Probab=99.79 E-value=2.8e-18 Score=171.73 Aligned_cols=188 Identities=12% Similarity=0.054 Sum_probs=0.0

```
Q ss_pred             cEEEEEcchhccCCCCCCCcc---ccccccchHHHHHHHHHHHHhhcCCCceEEEeCCCcCCccHHHHH-------HHHH
Q gi|45553317|re  262 LTSGTYNDSLLTINSDYDAYL---LDFPLMGDDFMLYLARMELKCRFRRHERVLQSGLCVSGLTINGAR-------NRLK  331 (468)
Q Consensus       262 ~~Iv~iGDSIT~G~~~~~~~~~~---~~~~liGds~~~~La~~~l~~~~~~~~~~V~N~G~~isG~ts~~~~~-------~rl~  331 (468)
                        ++++++|||+|.|........   .+-.....+|+.++++.+-..     .+.+.|.|  ++|.++.+++        ....
T Consensus         1 ~~~valGDS~~aG~g~~~~~~~~~~~~~~c~~s~~~y~~~la~~l~~~~----~~~~~~~n~a--~sG~~~~~~~~~~~~~~~~~~q~   74 (259)
T cd01823         1 VRYVALGDSYAAGPGAGPLDDGPDDGCRRSSNSYPTLLARALGDE----TLSFTDVA--CSGATTTDGIEPQQGGIAPQA   74 (259)
T ss_pred             CCEEEecchhhcCCCCCccCCCCCCCCcCCcccHHHHHHHHhCCC----CCceeEEe--eeCccccccccccCCChHHH


Q ss_pred             HHHhcCCCeEEEEeeeccCCCC---------------------------CCHHHHHHHHHHHHHHHhC--CCEEEEEcC
Q gi|45553317|re  332 RVQLPEGTQIIVNIGSVDIMRG---------------------------KPLVQIEHDFRLLIKEMHNM--RLVPILTNL  382 (468)
Q Consensus       332 ~~l~~~~PDlVvI~lGtND~~~g---------------------------~s~e~f~~~l~~LI~~lr~~~~ga~VIL~t~  382 (468)
                   ..+...+|+|+|.+|+||+...                                ...+.|..+|..+|+.+++.  +++|+|+|+++
T Consensus        75 ~~l~~~~d~V~i~iGgND~~~~~~~~~~~~~c~~~~~~~~~~~~~~~~~~~~~~~~~~~~~~~~~~~~~l~~il~~i~~~~p~~~I~l~~y  154 (259)
T cd01823        75 GALDPDTDLVTITIGGNDLGFADVVKACILTGGGSSLAQEKGAADGARDAALDEVGARLKAVLDRIRERAPNARVVVVGY  154 (259)
T ss_pred             cccCCCCCEEEEEeCCccCHHHHHHHHhhccCcCCcchhccccHHHHHHHHHHHHHHHHHHHHhhCCCcEEEEecC


Q ss_pred             CCCCc-----------------ccchhhHHHHHHHHHHHHHHHhC--C---CEEEECHHHHhhhcccccHh---------
Q gi|45553317|re  383 APLGN-----------------YCHDKVLCDKIYRFNKFIRSECC--H---LKVIDIHSCLINERGVVRFD---------  431 (468)
Q Consensus       383 ~~P~~~~~---------------~~~~~~~~~~~~~~i~~~~N~~ir~~A~--g---v~fIDl~~~~~~~~~~~g~~~~~~---------  431 (468)
                   ++...                 ....+...+.+..+|++++++|+  +   +.|||++..|.....+.....
T Consensus       155 ~~~~~~~~~~~~~~~~~~~~~~~~~~~~~~~~~~~~~~~~~~~~~~~~~~~~~~ln~~i~~~a~~~g~~~v~~VD~~~~f~~~~~~c~~~~~~~~~~~~~~~~~~~~~  234 (259)
T cd01823       155 PRLFPPDGGDCDKSCSPGTPLTPADRPELNQLVDKLNALIRRAAADAGDYKVRFVDTDAPFAGHRACSPDPWSRSVLDLL  234 (259)
T ss_pred             cccccCCCCCccccccccccCCCCHHHHHHHHHHHHHHHHHHHHHHHHHHHhCCCCeEEEECcccCCccccCCCCccccC


Q ss_pred             -hcCCCCcCCCCcHHHHHHHHHHHHHHH
Q gi|45553317|re  432 -CFQASPRQVTGSKEPYLFWNKIGRQR  457 (468)
Q Consensus       432 -~~~~dDGvHPn~~~~~~Gy~~~lA~~i~~~~  457 (468)
                   .+..|++|||.  +||++||+.+.++
T Consensus       235 ~~~~~~d~~HPn~--~~~G~~~~iA~~~i~~~~  259 (259)
T cd01823       235 PTRQGKPFHPNA--AGHRAIADLIVDA  259 (259)
T ss_pred             CCCCCccccCCH--HHHHHHHHHHhhC
```

No 79

>**TIGR04294** TIGR04294 prepilin-type processing-associated H-X9-DG domain; InterPro:
This entry

describes a region of approximately 16 residues found typically about 30 residues away from the C terminus of large numbers of proteins in the Planctomycetes, Lentisphaerae, and Verrucomicrobia, on proteins with a prepilin-type N-terminal cleavage/methylation domain (see TIGR02532 from TIGRFAMs). The motif H-X(9)-D-G is nearly invariant. Single genomes may encode over 200 such proteins.
.

Probab=99.64 E-value=1.2e-19 Score=9.09 Aligned_cols=6 Identities=17% Similarity=0.274 Sum_probs=0.0

```
Q ss_pred             CCEEEE
Q gi|45553317|re  411 HLKVID  416 (468)
Q Consensus       411 gv~fID  416 (468)
                   ++.|+|
T Consensus         7 N~lf~D   12 (16)
T TIGR04294       7 NVLFAD   12 (16)
```

No 80

>**pfam00657** Lipase_GDSL GDSL-like Lipase/Acylhydrolase.

Probab=99.74 E-value=4.4e-17 Score=154.79 Aligned_cols=177 Identities=15% Similarity=0.098 Sum_probs=0.0

```
Q ss_pred             EEEEcchhccCCCCCCCcccccccccchHHHHHHHHHHHHhhcCCC----ceEEEeCCCcCCccHH--------HHHHHHH
Q gi|45553317|re  264 SGTYNDSLLTINSDYDAYLLDFPLMGDDFMLYLARMELKCRFRRH----ERVLQSGLCVSGLTIN--------GARNRLK  331 (468)
Q Consensus       264 Iv~iGDSIT~G~~~~~~~~~~~~~~~~liGds~~~~La~~~l~~~~~~~~~~~~----~~V~N~G~~isG~ts~~~~~~~~~~~~~rl~  331 (468)
                   |+++|||||.|......          .+|...+.. .+...+...    +.+.|.|  ++|.++.          .......
T Consensus         1 i~~~GDSit~g~~~~~~~~~~~~~~~~~~~~~l~~~~~~~~~~~~~~~~~~~n~~~~~~~G~~~~~~~~~~~~~~~~~~~~~~~~~
T pfam00657       1 IVAFGDSLTDGYGDGGG---------NGWGAGLAD-RLTSDVGLNQFWGVDYFNRG--ISGRTSDSQALGLLQELLRLVD   68 (219)
T ss_pred             CeeecccccCCCCCCc---------cchhhhhh-hhhhhccccccceeecc--cCCCccccccchhhHHHhhcccc


Q ss_pred             HHHhcCCCeEEEEeeeccC-------CCCCCHHHHHHHHHHHHHHHHH-hCCCEEEEEcCCCCCccccch------------
Q gi|45553317|re  332 RVQLPEGTQIIVNIGSVDI-------MRGKPLVQIEHDFRLLIKEMH-NMRLVPILTNLAPLGNYCHD------------  391 (468)
Q Consensus       332 ~~l~~~~PDlVvI~lGtND~~~~~~~~~~~~g~s~e~f~~~l~~LI~~lr~~~ga~VIL~t~~P~~~~~~~~~~~~~~~~~~~~~~~~~
                   ......||+|+|++|++|+       ....+.++|..+++.|+.++ +.+..+|++..++.......
T Consensus        69 ~~~~~~~~~~~vvi~~G~ND~~~~~~~~~~~~~~~~~~~~~~~~~~~~i~~l~~~~~~~~~~~~ii~~~~~~~~~~~~~~~~~~~~~~~~~~~~~  148 (219)
T pfam00657      69 VDDQFSPDLVTIFIGTNDLITKSTKSDQNVSVPEFIDNLRSLIDLLRSRNGARIIILITPVLINLPPLGASQKNVDAAGC  148 (219)
T ss_pred             hhcccCCcEEEEEeccchhhccccccchhhhhhhhhhhhhhhhhhhcCCceeecccceeeccCCCCChhhcchhhH


Q ss_pred             -hhHHHHHHHHHHHHHHHhC---------CCEEEEChHHHhhccc-ccHhhcCCCCcCCCCcHHHHHHHHHHHH
Q gi|45553317|re  392 -KVLCDKIYRFNKFIRSECC---------HLKVIDIHSCLINERGV-VRFDCFQASPRQVTGSKEPYLFWNKIG  454 (468)
Q Consensus       392 -~~~~~~~i~~~N~~ir~~A~~~~~~~~~~~~~~~~~gv~fIDl~~~~~~~~~~g~~~~~~~~~~~~~~~~dDGvHPn~~~~~~Gy~~~lA~~~i  454 (468)
                    ....+....||+.++++++++        ++.|+|++..+.......++....+++..||+++.  +||++||+.|
T Consensus       149 ~~~~~~~~~~~~~~~~~~~n~~l~~~~~~~~~~~~~~~~~~~~~~~~~~~~~~~~v~~~~d~~~~~~~~~~~~~~~~~~~~~~~D~~Hp~~--~~G~~~~~a~~i  219 (219)
T pfam00657     149 LERLNEAVADYNSALRELAEELRKLKGADVPYVDLYSIFQDLDGIQNPSSYLFWDGLHPSE--KGYKAMAEAI  219 (219)
T ss_pred             HHHHHHHHHHHHHHHHHHHHHHhcCCCCcEEEehHHHHHHHHhcCCccceeecccCCchH--HHHHHHHhhC
```

No 81

>**4k7j_A** GDSL-like lipase/acylhydrolase family protein; alpha/beta fold, peptidoglycan hydrolase; HET: EPE; 1.97A {Neisseria meningitidis} PDB: **4k40_A** **4k3u_A*** **4k9s_A**

Probab=99.72 E-value=4.7e-17 Score=172.32 Aligned_cols=155 Identities=6% Similarity=0.047 Sum_probs=0.0

```
Q ss_pred             CCCceEEEeCCCcCCccHHHHH----HHHHHHHhcCCCeEEEEeeeccCCCC-CCCHHHHHHHHHHHHHHHhC--CCEEE
```

```
Q gi|45553317|re  306 RRHERVLQSGLCVSGLTINGAR----NRLKRVQLPEGTQIIVNIGSVDIMR-GKPLVQIEHDFRLLIKEMHNM--RLVPI  378 (468)
Q Consensus       306 ~~~~~V~N~G~~isG~ts~~~~----~rl~~~l~~~PDlVvI~lGtND~~~~-g~s~e~f~~~l~~LI~~lr~~--ga~VI  378 (468)
                      ..++.|.|.|  ++|.++.++.    ...+.+...+||+|+|++|+|+.. +.+.++|.++++++|+.+++.  +++|+
T Consensus       206 ~~gv~v~N~G--isG~t~~~~~~~~~~~~~~~~~l~~~~pdlViI~~GtND~~~~~~~~~~~~~~~~~~l~~li~~ir~~~P~a~Iv  283 (379)
T 4k7j_A          206 AGGITVSAMG--INGAQLTQWSKWRADRMNDLAQTGADLVILSYGTNEAFNNNIDIADTEQKWLDTVRQIRDSLPAAGIL  283 (379)
T ss_dssp            TCEEEEEEEE--CTTCCGGGGGGSCTTHHHHHHTTCCSEEEEECCHHHHTCTTCCHHHHHHHHHHHHHHHHHHHCTTCEEE
T ss_pred            CCCEEEEccc--CCCcchhhcccccHHHHHHHhhhCCCEEEEECcccccCCCCCHHHHHHHHHHHHHHHHHHHCCCCcEE


Q ss_pred            EEcCCCCCc--ccchhhHHHHHHHHHHHHHHHHhC--CCEEEEChHHHhhhc---ccccHhhcCCCCcCCCCcHHHHHHHH
Q gi|45553317|re  379 LTNLAPLGN--YCHDKVLCDKIYRFNKFIRSECC--HLKVIDIHSCLINER---GVVRFDCFQASPRQVTGSKEPYLFWN  451 (468)
Q Consensus       379 L~t~~P~~~--~~~~~~~~~~~~i~~~N~~ir~~A~--gv~fIDl~~~~~~~~~~---g~~~~~~~~~dDGvHPn~~~~~Gy~~lA  451 (468)
                      |++++|...  ........+.+..+|++++++|+  ++.|||++..+.+..   .+....++..||+||||.  +|||++||
T Consensus       284 l~~~~~~~~~~~~~~~~~~~~~~~~~~n~~i~~~A~~~~v~~vD~~~~~~~~~~~~~~~~~~~~~~~~DgvHpn~~~~~Gy~~iA  361 (379)
T 4k7j_A          284 IIGAPESLKNTLGVCGTRPVRLTEVQQMQRRVARQGQTMFWSWQNAMGGICSMKNWLNQGWAAKDGVHFSA--KGYRRAA  361 (379)
T ss_dssp            EEECCCCCSCCCSTTCCCCTTHHHHHHHHHHHHHHHHTTCEEEEHHHHTTSSSCHHHHHHTTSBCTTSSSBCH--HHHHHHH
T ss_pred            EECCCChhccCCCccccchHHHHHHHHHHHHHHHHHcCCEEEEhHHhhCCCcchhhhhccccccCCcccccCH--HHHHHHH


Q ss_pred            HHHHHHHHHHHHH
Q gi|45553317|re  452 KIGRQRVLQVIET  464 (468)
Q Consensus       452 ~~i~~~~I~~~L~~  464 (468)
                      +.+.+.|.+++++
T Consensus       362 ~~l~~~~L~~~l~~  374 (379)
T 4k7j_A          362 EMLADSLEELVRS  374 (379)
T ss_dssp            HHHHHHHHHHHHT
T ss_pred            HHHHHHHHHHHHH
```

No 82

>**4dev_A** Acetyl-xylan esterase EST2A; hydrolase; 2.00A {Butyrivibrio proteoclasticus} PDB: **3u37 _A**
Probab=99.71 E-value=7.2e-17 Score=172.64 Aligned_cols=181 Identities=10% Similarity=0.018 Sum_probs=0.0

```
Q ss_pred            CCCCCCccEEEEEcchhccCCCC-----CCCcccccccccchHHHHHHHHHHHhhcCCCCceEEEeCCCCcCCc--------
Q gi|45553317|re  255 MTPTPTILTSGTYNDSLLTINSD-----YDAYLLDFPLMGDDFMLYLARMELKCRFRRHERVLQSGLCVSGL--------  321 (468)
Q Consensus       255 ~~~~P~~~~~Iv~iGDSIT~G~~~~~-----~~~~~~~~~~~~~~liGds~~~~La~~~l~~~~~~~~~~~V~N~G~~isG~--------  321 (468)
                      +.+ +...+|+|+|||||.|...     .........-.+.+|+..+++.+       +..+.|.|  ++|.
T Consensus       160 ~~p~~~~~~~iv~iGDSiT~G~g~~~~~~~~~~~~~~~~~~~~~~~~~~y~~~la~~L~~~~~~~~~~~~~~~~~a~~~sG~~~~~~~~~~~~~g  229 (408)
T 4dev_A          160 NVP-VYSRKLEFIGDSITSGEGSYGAFDDVDWIPMYMSASANYATMTAKAL-------NADYHLVS--QGGWGVFCGWDN  229 (408)
T ss_dssp            CCC-CCCCEEEEEESTTTTTTSSCTTCCCCGGGCCSSSCHHHHHHHHHT-------TCEEEEEE--CTTCBSSSCTTC
T ss_pred            CCC-CCCceEEEEeCcHhhcCCCcCccccCCCCCCccccccHHHHHHHHh-------CccceeeE--ecCCceeccCCC


Q ss_pred            ----cHHHHHHHHH-----------------HHHhcCCCCeEEEEeeeccCCCC--------------------------C
Q gi|45553317|re  322 ----TINGARNRLK-----------------RVQLPEGTQIIVNIGSVDIMRG--------------------------K  354 (468)
Q Consensus       322 ----ts~~~~~rl~-----------------~~l~~~PDlVvI~lGtND~~~g--------------------------~  354 (468)
                          +......++.                 +....+||+|+|++|+|+..+                          .
T Consensus       230 ~~~~~~~~~~~~~~~~~~~~~~~~~~~~G~~~~~~lg~~~~~~~d~~~~~~~pdlVvI~lGtND~~~~~~~~~~p~~~~~~~~~g~~~~~~~~~~~~~~~~g~~~~~  309 (408)
T 4dev_A          230 DVRHNLPSVYEKVCGLAKGEMNEELGAQEEYDFASWQPDAIIVNLGTNDVTSFNQPEFLNPDDGKTYKMRTNTDGTRNRE  309 (408)
T ss_dssp            CTTCBGGGGSSBTTTTCCSHHHHHTTTTSBCCGGGCCCSEEEEEECCHHHHHHTTSCCEECTTTSCEECCCBCTTSSBCHH
T ss_pred            CCCCChhHHHHhhccccccccccCCcccccccCCCCEEEEeccCCCCcccccccccccccccchhhhcccccccccc


Q ss_pred            CHHHHHHHHHHHHHHHHHhC--CCEEEEEcCCCCCccccchhhHHHHHHHHHHHHHHHHhC--CCEEEEChHHHhhhcccccH
Q gi|45553317|re  355 PLVQIEHDFRLLIKEMHNM--RLVPILTNLAPLGNYCHDKVLCDKIYRFNKFIRSECC--HLKVIDIHSCLINERGVVRF  430 (468)
Q Consensus       355 s~e~f~~~l~~LI~~lr~~--~ga~VIL~t~~P~~~~~~~~~~~~~~~i~~~N~~ir~~A~--gv~fIDl~~~~~~~~~~~g~~~~~~  430 (468)
                      +.++|.++|++|+.+++.  +++||+++ +|+     .....+.+.+++.+.+++.+.  ++.|||++..           ..
T Consensus       310 ~~~~~~~~~~l~~li~~ir~~~P~a~Ivl~~p~~~~~~~~~~~~~~~~~~~~~~~~~~~~~~~~~~~~i~~la~~a~~~~v~~vD~~~~~~~~~~~~~~~~~~~~~~~  375 (408)
T 4dev_A          310 DELKIVSAIIDFLTMLRKHNPNAQIIWSY-GML-----GSDLNLVITEGINKYKENAGDEKVSFFQLPNT--------TM  375 (408)
T ss_dssp            HHHHHHHHHHHHHHHHHHHHCTTCEEEEEE-CTT-----CCTTHHHHHHHHHHHHHHHHTCCSEEEEECCCC--------CG
T ss_pred            cHHHHHHHHHHHHHHHHHHCCCCEEEEEe-CCC-----CchHHHHHHHHHHHHHHHHHcCCCCEEEEECCCC--------Cc


Q ss_pred            hhcCCCCcCCCCcHHHHHHHHHHHHHHHHHHHHH
Q gi|45553317|re  431 DCFQASPRQVTGSKEPYLFWNKIGRQRVLQVI  462 (468)
Q Consensus       431 ~~~~dDGvHPn~~~~~Gy~~lA~~i~~~~I~~~L  462 (468)
                      +++.|+ +|||.  +||++||+.+.+.|.+++
T Consensus       376 ~~~~Dg-vHPn~--~~G~~~~A~~l~~~~l~~~l  404 (408)
T 4dev_A          376 ENFGSH-MAPGP--KSHQNAAKELVDYLRNKL  404 (408)
T ss_dssp            GGBCST-TSBCH--HHHHHHHHHHHHHHHHHH
T ss_pred            CCCCCC-cCcCH--HHHHHHHHHHHHHHHHHh
```

No 83

>**COG2755** TesA Lysophospholipase L1 and related esterases [Amino acid transport and metabolism]
Probab=99.71 E-value=6.8e-16 Score=150.40 Aligned_cols=189 Identities=16% Similarity=0.170 Sum_probs=0.0

```
Q ss_pred            EEEEEcchhccCCCCCCCcccccccccchHHHHHHHHHHHHhhcCCCCeEEEeCCCCcCCcHHHHHHHHHHHHHhcC--CCe
Q gi|45553317|re  263 TSGTYNDSLLTINSDYDAYLLDFPLMGDDFMLYLARMELKCRFRRHERVLQSGLCVSGLTINGARNRLKRVQLPE--GTQ  340 (468)
Q Consensus       263 ~Iv~iGDSIT~G~~~~~~~~~~~~~~~~~~~~liGds~~~~La~~~l~~~~~~~~~~~V~N~G~~isG~ts~~~~~rl~~~l~~~--PDl  340 (468)
                      +++++|||+|.|.....       -....|...++..+..........+.|.|  ++|.++.+++.++...+...  +|+
T Consensus        10 ~i~~~GDSlt~g~~~~~~~~~~~~~~~~~~~~~~~~~~~~~~~~~~~~~l~~~~l~~~~~~~~~~~~~~~~~~n~~~~~s~~~~~~~~g~~rl~~~l~~~~~~~d~  80 (216)
T COG2755         10 TVLALGDSLTAGYGPAN-------RLEGGWVQVLAKRLVAKGVAVINLVLNGG--ISGDTSAGGLQRLPALLKQHLPPDL  80 (216)
T ss_pred            cEEEeehhhhhcccCCC-------cccccHHHHHHHHhcCcceeeeeecce--eecCCchhHHHHhHHHHhccCCCCE


Q ss_pred            EEEEeeeccC--CCCCCH--HHHHHHHHHHHHHHHHHhCC-------CEE-EEEcCCCCCccccchhhHHHHHHHHHHHHHHHH
Q gi|45553317|re  341 IIVNIGSVDI--MRGKPL--VQIEHDFRLLIKEMHNMR-------LVP-ILTNLAPLGNYCHDKVLCDKIYRFNKFIRSE  408 (468)
Q Consensus       341 VvI~lGtND~~~~~g~s~~~e~f~~~l~~LI~~lr~~g-------a~V-IL~t~~P~~~~~~~~~~~~~~~~i~~~N~~ir~~  408 (468)
                      |+|++|+|+.  .++.+  +.+..++++.+..+++++       ..+ ++...+|+.........+.....+++.++++++
T Consensus        81 v~i~lG~ND~~~~~~~~~~~~~~~~~~~~~~~~~~~~~~~~~~~~~~~~~~~~~~~~~~~~~~~~~~~~~~~~~~~~~~~~li~~~~~~~~~~~~~~~~~~~~~~~~~~~~~~p~~~~~~~~~~~~~~~~~~~~~~~~l  160 (216)
T COG2755         81 VIIMLGGNDIGPLRGFSPNAAEFTKNLRQLIQALRKANPLAGLTPIPVLLVGIAPPFIATDFPTYGADWFHAANEILAQL  160 (216)
T ss_pred            EEEEeccccccchhccCCCHHHHHHHHHHHHHHHHHhCCccccccceEEEEecCCCCcccchhHHHHHHHHHHHHHHHHH


Q ss_pred            hC--CCEEEEChHHHhhhcccccHhhcCCCCcCCCCcHHHHHHHHHHHHHHHHHHHHHHHH
Q gi|45553317|re  409 CC--HLKVIDIHSCLINERGVVRFDCFQASPRQVTGSKEPYLFWNKIGRQRVLQVIET  464 (468)
Q Consensus       409 A~--gv~fIDl~~~~~~~~~~~g~~~~~~~~~dDGvHPn~~~~~Gy~~lA~~i~~~~I~~~L~~  464 (468)
                      +.  .+++.|++..+.+ +...+.....||+|+|||.  +||++.||+.+.+.|.+.+.+..
```

```
T Consensus       161 a~~~~v~~~d~~~~~~~--~~~~~~~~~~~Dg~H~n~--~Gy~~~a~~l~~~l~~~~~~  214 (216)
T COG2755         161 ANELFVPLADLFDAGVD--GGRLPELLTEDGLHPNA--KGYQALAEALAEVLAKLLKL  214 (216)
T ss_pred             hhhcCccchHHHhccc--ccccccccccCCcCcCh--hhHHHHHHHHHHHHHHHHHhcc
```

No 84

>cd01840 SGNH_hydrolase_yrhL_like yrhL-like subfamily of SGNH-hydrolases, a diverse family of lipases and esterases. The tertiary fold of the enzyme is substantially different from that of the alpha/beta hydrolase family and unique among all known hydrolases; its active site closely resembles the Ser-His-Asp(Glu) triad found in other serine hydrolases. Most members of this sub-family appear to co-occur with N-terminal acyltransferase domains. Might be involved in lipid metabolism.
Probab=99.70 E-value=5.8e-16 Score=143.64 Aligned_cols=144 Identities=10% Similarity=0.097 Sum_probs=0.0

```
Q ss_pred             EEEEcchhccCCCCCCCcccccccccchHHHHHHHHHHHHhhcCCCCceEEEeCCCcCCccHHHHHHHHHHHHhcC--CCeE
Q gi|45553317|re  264 SGTYNDSLLTINSDYDAYLLDFPLMGDDFMLYLARMELKCRFRRHERVLQSGLCVSGLTINGARNRLKRVQLPE--GTQI  341 (468)
Q Consensus       264 Iv~iGDSIT~G~~~~~~~~~~~~~~~~~liGds~~~~La~~~l~~~~~~~~~~~~~V~N~G~~isG~ts~~~~~~rl~~~l~~~~--PDlV  341 (468)
                      |++||||+|.|                        ....++..|+.   +.-.|   ..|.++.++...++......   +|+|
T Consensus         2 i~~iGDSv~~~~----------------------~~~~l~~~~p~~---~~ida--~v~r~~~~~~~~~~~~~~~~~~~~~~~~~~V   54 (150)
T cd01840         2 ITAIGDSVMLD----------------------SSPALQEIFPN---IQIDA--KVGRQMSEAPDLIRQLKDSGKLRKTV   54 (150)
T ss_pred             eEEEechHHhc----------------------cHHHHHHCCC---CEEec--cccCcHHHHHHHHHHHHHHcCCCCCEE
```

```
Q ss_pred             EEEeeeccCCCCCCHHHHHHHHHHHHHHHHHhCCCEEEEEcCCCCCccccchhhHHHHHHHHHHHHHHHHHhC---CCEEEECh
Q gi|45553317|re  342 IVNIGSVDIMRGKPLVQIEHDFRLLIKEMHNMRLVPILTNLAPLGNYCHDKVLCDKIYRFNKFIRSECC---HLKVIDIH  418 (468)
Q Consensus       342 vI~lGtND~~~g~s~e~f~~~l~~LI~~lr~~ga~VIL~t~~P~~~~~~~~~~~~~~~~~i~~~~N~~ir~~A~---gv~fIDl~  418 (468)
                      ||++|+||.        .+.+++++++++.+.  .+.+|+|+++..          ...+...+|+.++++|+    +++++|++
T Consensus        55 vi~lGtN~~~~-----------~l~~~i~~~~~-----~~v~lv~~~~---------~~~~~~~~~~~~~~n~~~~~~~~A~~~~~~v~~iDw~  117 (150)
T cd01840        55 VIGLGTNGP-------FTKDQLDELLDALG-PDRQVYLVNPHV---------PRPWEPDVNAYLLDAAKKYKNVTIIDWY  117 (150)
T ss_pred             EEEeCCCCc-------ccHHHHHHHHHHHhC-CCCEEEEEeCCC---------CcchHHHHHHHHHHHHHHHHCCCCEecChH
```

```
Q ss_pred             HHHhhhcccccHhhcCCCCcCCCCcHHHHHHHHHHHHHHHH
Q gi|45553317|re  419 SCLINERGVVRFDCFQASPRQVTGSKEPYLFWNKIGRQRV  458 (468)
Q Consensus       419 ~~~~~~~~~g~~~~~~~~~~~dDGvHPn~~~~~Gy~~lA~~~i~~~~I  458 (468)
                      ....+     .++++..||+|||.  +||+.+|+.+.++|
T Consensus       118 ~~~~~~-----~~~~~~~~~~~DgiHPn~~~~~G~~~~~a~~i~~ai  150 (150)
T cd01840       118 KAAKG-----HPDWFYGDGVHPNP--AGAKLYAALIAKAI  150 (150)
T ss_pred             HHhcc-----ChhhcCCCCCCcCH--HHHHHHHHHHHHHhC
```

No 85

>2o14A02
Probab=99.69 E-value=6.3e-15 Score=144.43 Aligned_cols=192 Identities=15% Similarity=0.160 Sum_probs=0.0

```
Q ss_pred             cEEEEEcchhccCCCCCCCcccccccccchHHHHHHHHHHHHhhc-CCCCceEEEeCCCcCCccHHHHH--HHHHHHHhc--
Q gi|45553317|re  262 LTSGTYNDSLLTINSDYDAYLLDFPLMGDDFMLYLARMELKCRF-RRHERVLQSGLCVSGLTINGAR--NRLKRVQLP--  336 (468)
Q Consensus       262 ~~Iv~iGDSIT~G~~~~~~~~~~~~~~~~liGds~~~~La~~~l~~~~~~-~~~~~~V~N~G~~isG~ts~~~~~~--~rl~~~l~~--  336 (468)
                      .+|+++|||++.+..........         +|...|..      .+ ...+.|.|.|   ++|.++..+.   .+++.++..
T Consensus         2 ~~~~~~~GDS~~~~~~~~~~~~~~~~~~~~~~-------w~~~~~~~~~~~~~~~~~~~~~~~~~~~~~n~a--~~G~~~~~~~~~~~~~~~~~~~~~~~~  68 (214)
T 2o14A02         2 PTIVLAGDSTTAGYGASNGEPEA------GWGQVLAE-----FLDEEGVKVVNHA--ISGRSSRSFLAEGRLDKVLKKLK  68 (214)
T ss_pred             CEEEEEccccccCCCCCCCccc------CHHHHHHh-----HcCCCCeEEEEeC--cCcCCHHHHHHHhhhHHHHHHHhcC
```

```
Q ss_pred             CCCeEEEEEeeeccCCC--CCCHHHHHHHHHHHHHHHHhCCCEEEEEcCCCCCcccc-chhhHHHHHHHHHHHHHHHHhC--C
Q gi|45553317|re  337 EGTQIIVNIGSVDIMR--GKPLVQIEHDFRLLIKEMHNMRLVPILTNLAPLGNYC-HDKVLCDKIYRFNKFIRSECC--H  411 (468)
Q Consensus       337 ~PDlVvI~lGtND~~~~--g~s~e~f~~~l~~LI~~lr~~ga~VIL~t~~P~~~~~~~~~-~~~~~~~~~~~~~~i~~~N~~ir~~A~--g  411 (468)
                      .+|+|+|.+|.||...  ..+...|..+++.+|+.+++.++.+|++++.+.+...+.  ...........|+.+++++++   +
T Consensus        69 ~~~~v~i~~G~ND~~~~~~~~~~~~~~~~~~~l~~~~~~~~~~~~~~~~~~~~~~~~~~~~~~~~~~~~~~~~~~~~~~~~~~~a~~~~~  148 (214)
T 2o14A02        69 KGDYVLIQFGHNDQKERSTEPVTTYKENLRRLIKEARAKGAKPVLVTPTPRRSFDEESGKIEDTLGDYAEAIREVAKEEG  148 (214)
T ss_pred             CCCEEEEEeeeCCCCCCCCCCCHHHHHHHHHHHHHHHHHHCCCEEEEEEeeccCCCCCCCCCeecccHHHHHHHHHHHHHHcC
```

```
Q ss_pred             CEEEECHHHHhhhc-----ccccHhhcCCCCcCCCCcHHHHHHHHHHHHHHHHHHHHHHHHhcC
Q gi|45553317|re  412 LKVIDIHSCLINER-----GVVRFDCFQASPRQVTGSKEPYLFWNKIGRQRVLQVIETSLEY  468 (468)
Q Consensus       412 v~fIDl~~~~~~~~~~~~~~~~-----g~~~~~~~~~~~dDGvHPn~~~~~Gy~~lA~~~i~~~~I~~~~L~~~~~a~  468 (468)
                      +.+||+...+....       ......++..|++||+.   .|+..+|+.+.+.|.+...+...+
T Consensus       149 ~~~iD~~~~~~~~~~~~~~~~~~~~~~~G~~~~~~~~~~~~~~~~D~~H~~~~~~~G~~~~~A~~~~~~~~~~~~~~~~~~~  208 (214)
T 2o14A02       149 VPLIDLNAASAALVEKLGPEKSKKLFLSEDNTHPNE--AGAKLIARLVAEALKKEVLELAKY  208 (214)
T ss_pred             ceEEehHHHHHHHHHHHChHhcccccCCcCCCCCCh--HHHHHHHHHHHHHHHhhhhHHHHHH
```

No 86

>3bzw_A Putative lipase; protein structure initiative II, (PSI-II), NYSGXRC, structural genomics; 1.87A {Bacteroides thetaiotaomicron vpi-5482} SCOP: c.23.10.9
Probab=99.68 E-value=2.2e-16 Score=156.53 Aligned_cols=183 Identities=13% Similarity=0.063 Sum_probs=0.0

```
Q ss_pred             CccEEEEEcchhccCCCCCCCcccccccccchHHHHHHHHHHHHhhcCCCCceEEEeCCCcCCccHHHHHHHHHHHHhc---
Q gi|45553317|re  260 TILTSGTYNDSLLTINSDYDAYLLDFPLMGDDFMLYLARMELKCRFRRHERVLQSGLCVSGLTINGARNRLKRVQLP---  336 (468)
Q Consensus       260 ~~~~Iv~iGDSIT~G~~~~~~~~~~~~~~~~liGds~~~~La~~~l~~~~~~~~~~~~~V~N~G~~isG~ts~~~~~~rl~~~l~~---  336 (468)
                      ...||+|+|||||.|+....              ..+.|...+..++         +..++|.|   ++|.++.....++......
T Consensus        25 ~~kkI~flGDSiT~G~g~~~~~----------~~~~y~~~l~~~l------------~~~~N~g--v~G~~~~~~~~~~~~~~~~~~~~~~~  87 (274)
T 3bzw_A        25 QGKKVGYIGDSITDPNCYGD--------NIKKYWDFLKEWL-------GITPFVYG--ISGRQWDDVPRQAEKLKKEHGG  87 (274)
T ss_dssp             TTCEEEEEESTTTCTTTTGG--------GCCCHHHHHHHHH-------CCEEEECC--CTTCCGGGHHHHHHHHHHHHTT
T ss_pred             CcCEEEEEcchhhcCCCCCC--------ccchHHHHHHHHc-------CCeEEEEe--cCCCCcCcccchhhHhhhhhccC
```

```
Q ss_pred             CCCeEEEEEeeeccCCCCC----------------------------------CHHHHHHHHHHHHHHHHhC--CCEEEEEc
Q gi|45553317|re  337 EGTQIIVNIGSVDIMRGK----------------------------------PLVQIEHDFRLLIKEMHNM--RLVPILTN  381 (468)
Q Consensus       337 ~PDlVvI~lGtND~~~g~----------------------------------s~e~f~~~l~~LI~~lr~~~~~ga~VIL~t  381 (468)
                      .+|+|+|.+|+||...+.                                  +.+.+...+..+++.+++.   ...|++++
T Consensus        88 ~~d~ivi~~g~Nd~~~~~~~~~~~~~~~~~~~~~~~~~~~~~~~~~~~~~~~~~~~~~~~~i~~l~~~~~~~~~~i~~~~  167 (274)
T 3bzw_A        88 EVDAILVFMGTNDYNSSVPIGEWFTEQEEQVLSAHGEMKKMVTRKKRTPVMTQDTYRGRINIGITQLKKLFPDKQIVLLT  167 (274)
T ss_dssp             TCCEEEEECCCCCCHHHHTTCCCCSEEEEEEEEEEEESSCCEEEEEEEEECCCSSSHHHHHHHHHHHHHHHHCTTSEEEEEC
T ss_pred             CCCEEEEecCCcccccccChhhhhhhhhhhhhhccccchhhhhcccccccCCHHHHHHHHHHHHHHHHhCccccEEEEe
```

```
Q ss_pred             CCCCCc-----------ccchhhHHHHHHHHHHHHHHHHhC--CCEEEEChHHH----hhhcccccHhhcCCCCcCCCCcH
```

```
Q gi|45553317|re  382 LAPLGN-----------YCHDKVLCDKIYRFNKFIRSECC--HLKVIDIHSCL----INERGVVRFDCFQASPRQVTGSK  444 (468)
Q Consensus       382 ~~P~~~-----------~~~~~~~~~~i~~~N~~ir~~A~--gv~fIDl~~~~----~~~~g~~~~~~~~~dDGvHPn~~~  444 (468)
                      +.+...           .............+++.++++|.  ++.+||++..+    ...........+..||+|||.
T Consensus       168 ~~~~~~~~~~~~~~~~~~~~~~~~~~~~~~~~~~i~~~~~~~~~~v~~iDl~~~~~~~~~~~~~~~~~~~~~~~~~D~vHpn~--  245 (274)
T 3bzw_A          168 PLHRSLANFGDKNVQPDESYQNGCGEYIDAYVQAIKEAGNIWGIPVIDFNAVTGMNPMVEEQLIYFYDAGYDRLHPDT--  245 (274)
T ss_dssp             CCCCCCEECSTTEEECCTTBCCTTSCCHHHHHHHHHHHCCCCCCCEECHHHHTCCCTTSGGGGGGEEETTTEEEEECH--
T ss_pred             eccccccccccccccccccchhHHHHHHHHHHHHHHHHHHcCceEEechhhhcccccccccccccccCCCCCcCCCH--


Q ss_pred             HHHHHHHHHHHHHHHHH
Q gi|45553317|re  445 EPYLFWNKIGRQRVLQV  461 (468)
Q Consensus       445 ~Gy~~lA~~i~~~I~~~  461 (468)
                      .||++||+.|++.|.++
T Consensus       246 ~Gh~~iA~~i~~~L~~~  262 (274)
T 3bzw_A          246 KGQERMARTLMYQLLAL  262 (274)
T ss_dssp             HHHHHHHHHHHHHGGGS
T ss_pred             HHHHHHHHHHHHHHHcC
```

No 87

>4i8i_A Hypothetical protein; 5-stranded beta sheet flanked by 8 helices fold, structural joint center for structural genomics, JCSG; HET: MSE; 1.50A {Bacteroides uniformis}

Probab=99.67 E-value=2.9e-15 Score=151.76 Aligned_cols=178 Identities=10% Similarity=-0.004 Sum_probs=0.0

```
Q ss_pred             CCCCccEEEEEcchhccCCCCCCcccccccccchHHHHHHHHHHHHhhcCCCCceEEEeCCCcCCccHHHHHH--------
Q gi|45553317|re  257 PTPTILTSGTYNDSLLTINSDYDAYLLDFPLMGDDFMLYLARMELKCRFRRHERVLQSGLCVSGLTINGARN--------  328 (468)
Q Consensus       257 ~~P~~~~Iv~iGDSIT~G~~~~~~~~~~~~~~liGds~~~~La~~~l~~~~~~~~~~~V~N~G~~isG~ts~~~~~~--------  328 (468)
                      ..+..++|+|+|||||                    |+++..+|...+-..  +..+.|.|.|  ++|.|+.+...
T Consensus         6 ~~~~~~~Il~iGdSlT----------------~~~~p~~l~~~l~~~--g~~~~v~N~g--isG~t~~~~~~~~~~~~pd   65 (271)
T 4i8i_A            6 ANNDTIKVLAIGNSFS----------------QDAVEQYLHELGEAE--GITMIIGNMF--IGGCSLERHVQNIRNNAPA   65 (271)
T ss_dssp             ---CEEEEEEEEESHHH----------------HHHHSSSHHHHHHTT--TCEEEEEEEE--CTTCCHHHHHHHHHTTCCC
T ss_pred             CCCCceEEEEEccCCC----------------CCcHHHHHHHHHHhc--CCCceEEEeee--cCCccHHHHHhcccccccc


Q ss_pred             ------------------HHHHHHhcC-CCeEEEEeeeccCCCCCCHHHHHHHHHHHHHHHHh---CCCEEEEEcCCCCC
Q gi|45553317|re  329 ------------------RLKRVQLPE-GTQIIVNIGSVDIMRGKPLVQIEHDFRLLIKEMHN---MRLVPILTNLAPLG  386 (468)
Q Consensus       329 ------------------rl~~~l~~~-PDlVvI~lGtND~~~g~s~e~f~~~l~~LI~~lr----~ga~VIL~t~~P~~  386 (468)
                                        ++...+...  ||+|||..|.|+..   ..+.|++++++|++.+++    .+++|||+++.+..
T Consensus        66 ~~~~~ig~ng~~~~~~~~~l~~~L~~~~~D~Vil~~~~~~~~~---~~~~~~~~~l~~li~~~r~~~~~~~a~viL~~~~~~~  142 (271)
T 4i8i_A           66 YAYRKVEKDGEKTETRSMTIEKALADEKWDYISVQQASPLSG---IYDSYKASLPELVNYIRERIGKETVLMMHQTWAYA  142 (271)
T ss_dssp             EEEEEECTTSCEEEEEEECHHHHHHHSCCSEEEECCCGGGTT---CHHHHHHHHHHHHHHHHHTTSCTTCEEEEEECCCCC
T ss_pred             ccccccccCCccccccccchHHHHhccCCCCEEEeccCccCCC---CchhHHHHHHHHHHHHHHHhcCCCCEEEEEcCcCCC


Q ss_pred             cccc-------hhhHHHHHHHHHHHHHHHhC--CC-EEEEChHHHhhhcccccHhhcCCCCcCCC-CcHHHHHHHHHHH
Q gi|45553317|re  387 NYCH-------DKVLCDKIYRFNKFIRSECC--HL-KVIDIHSCLINERGVVRFDCFQASPRQVT-GSKEPYLFWNKIGR  455 (468)
Q Consensus       387 ~~~~-------~~~~~~~~i~~~N~~ir~~A~--gv-~fIDl~~~~~~~~~g~~~~~~~~~dDGvHPn-~~~~Gy~~lA~~i~  455 (468)
                      ....       .....+....+.++++++|+  ++ .|||++..|..........+..||+|||  .  +||.++|+.++
T Consensus       143 ~~~~~~~~~~~~~~~~~~~~~~~~~~~~~~~~lA~~~~~~~~lip~~~~~~~~~~~~~~~~~~l~~DgiHPn~--~G~~liA~~~~  220 (271)
T 4i8i_A          143 TNANHTGFKNYDQNQMKMYTSIVDAVKKAANLVGIKKIIPSGTAIQNARTSFIGDHMNRDGYHLDLT--IGRYTAACTWF  220 (271)
T ss_dssp             TTCCCGGGGGGTTCHHHHHHHHHHHHHHHHHHHTCCEEEEHHHHHHHHHHSTTCSCCBSSSSSBCTT--HHHHHHHHHHH
T ss_pred             cCCCCccccccCccHHHHHHHHHHHHHHHHHHhhcCCCEEccHHHHHHHHHhCCCcccccCCCCCCCCc--ccHHHHHHHHH


Q ss_pred             HHHH
Q gi|45553317|re  456 QRVL  459 (468)
Q Consensus       456 ~~I~  459 (468)
                      +.|.
T Consensus       221 ~~l~  224 (271)
T 4i8i_A          221 EALT  224 (271)
T ss_dssp             HHHH
T ss_pred             HHHh
```

No 88

>2wao_A Endoglucanase E; plant cell WALL degradation, carbohydrate metabolism, polysaccharide degradation, esterase, hydrolase, cellulases; HET: BGC; 1.80A {Clostridium thermocellum} PDB: 2wab _A*

Probab=99.65 E-value=2e-15 Score=157.29 Aligned_cols=211 Identities=9% Similarity=-0.010 Sum_probs=0.0

```
Q ss_pred             hhhcccccccccccccCCcCCCCCCccEEEEEcchhccCCCCCCCccccc-----ccccchHHHHHHHHHHHhhcCCC
Q gi|45553317|re  234 RHLGIFVHPFNGMNIMKRRHEMTPTPTILTSGTYNDSLLTINSDYDAYLLDF-----PLMGDDFMLYLARMELKCRFRRH  308 (468)
Q Consensus       234 ~~~~~~~~~~~g~~~~~~~~~e~~~~P~~~~Iv~iGDSIT~G~~~~~~~~~~~~~-----~liGds~~~~La~~~l~~~~~~~~~  308 (468)
                      ++.......+.|.....-.....+.|...||.|||||||.|..........     .-...+|...+++.+       +
T Consensus        95 ~e~~~~~~~~~~~~~~~~~~l~~~~~~~~~rI~fiGDSIT~G~g~~~~~~~~~~~~~~~~~~~sy~~~la~~l-------~  167 (341)
T 2wao_A           95 TEASLGEVQFLGFDFGSGKLLAAPKPLERKIEFIGDSITCAYGNEGTSKEQSFTPKNENSYMSYAAITARNL-------N  167 (341)
T ss_dssp             SCGGGCCEEEEEEECTTSEECCCCCCCSEEEEEEESHHHHTTTTTCCCTTSCCCGGGCCGGGSHHHHHHHHT-------T
T ss_pred             cccccccEEEEEEEecCCceecCCCCCCcEEEEEccccccCCccccCCCCCCccccccCccccCHHHHHHHHh-------C


Q ss_pred             ceEEEeCCCcCCccHH---------HHHHHHHHHHhc----------CCCeEEEEeeeccCCCCC-CHHHHHHHHHHHHH
Q gi|45553317|re  309 ERVLQSGLCVSGLTIN---------GARNRLKRVQLP----------EGTQIIVNIGSVDIMRGK-PLVQIEHDFRLLIK  368 (468)
Q Consensus       309 ~~V~N~G~~isG~ts~---------~~~~rl~~~l~~----------~PDlVvI~lGtND~~~g~-s~e~f~~~l~~LI~  368 (468)
                      ..+.+.|  ++|..+.          .+.+++...+..          .||+|||++|+||...+. ..+.|..+|..||+
T Consensus       168 a~~~~~a--sG~gi~~~~~~~~~~~~~~~~~~~~~~~~~~~~~~~~~d~~~~~Pd~VvI~lGtND~~~~~~~~~~~~~~~~~~~~~~~~~i~  245 (341)
T 2wao_A          168 ASANMIA--WSGIGLTMNYGGAPGPLIMDRYPYTLPYSGVRWDFSKYVPQVVVINLGTNDFSTSFADKTKFVTAYKNLIS  245 (341)
T ss_dssp             EEEEEEE--CTTCCSSCCGGGCCCCCHHHHTTEEETTTTEECCGGGCCCSEEEEECCHHHHSSSCCCHHHHHHHHHHHHH
T ss_pred             CCeEEee--ecceEEEcccCCCCCccHHHHHHhhhccccccccccCCCCEEEEEeccccccccCccHHHHHHHHHHHHH


Q ss_pred             HHHhC-CCEEEEEcCCCCCCccccHhhHHHHHHHHHHHHHHHHhC-CCEEEEChHHHhhhcccccHhhcCCCCCcCCCCCcHHH
Q gi|45553317|re  369 EMHNM-RLVPILTNLAPLGNYCHDKVLCDKIYRFNKFIRSECC-HLKVIDIHSCLINERGVVRFDCFQASPRQVTGSKEP  446 (468)
Q Consensus       369 ~lr~~-ga~VIL~t~~P~~~~~~~~~~~~~~~~~i~~~N~~ir~~A~-gv~fIDl~~~~~~~~~g~~~~~~~~~dDGvHPn~~~~G  446 (468)
                      .+++. ....|++..+|...........+.+.++.+.+++...  .+.++|+.        .....+++.|+ +|||.  +|
T Consensus       246 ~ir~~~p~a~Iv~~~~~~~~~~~~~~~~~~~~~~~~~~~~~~~~~~~~v~~~d~~-------~~~~~~~~~~D~~HPn~--~G  315 (341)
T 2wao_A          246 EVRRNYPDAHIFCCVGPMLWGTGLDLCRSYVTEVVNDCNRSGDLKVYFVEFP-------QQDGSTGYGED-WHPSI--AT  315 (341)
T ss_dssp             HHHHHCTTCEEEEEECSSCCHHHHHHHHHHHHHHHHHHHHHTTCCSEEEEECC-------CCCSTTCCCGG-GCCCH--HH
T ss_pred             HHHHHCCCCeEEEEeccccCCccHHHHHHHHHHHHHHHHHHcCCCcEEEEccc-------CcCccCCCCcC-CCCCH--HH
```

```
Q ss_pred                HHHHHHHHHHHHHHHHH
Q gi|45553317|re  447 YLFWNKIGRQRVLQVIE  463 (468)
Q Consensus       447 y~~lA~~i~~~I~~~L~  463 (468)
                      |++||+.|.+.|+++|+
T Consensus       316 h~~~A~~l~~~i~~~lg  332 (341)
T 2wao_A          316 HQLMAERLTAEIKNKLG  332 (341)
T ss_dssp             HHHHHHHHHHHHHHHHT
T ss_pred              HHHHHHHHHHHHHHhcC
```

No 89

>**template** PLATELET-ACTIVATING FACTOR ACETYLHYDROLASE.
Probab=99.65 E-value=4.9e-16 Score=194.77 Aligned_cols=175 Identities=18% Similarity=0.260 Sum_probs=0.0

```
Q ss_pred                 ccccccchHHHHHHHHHHHHhhcCCCCceEEEeCCCcCCccHHHHHHHHHHHHhcC--CCeEEEEeeeccCCCCCCHHHHHH
Q gi|45553317|re   284 DFPLMGDDFMLYLARMELKCRFRRHERVLQSGLCVSGLTINGARNRLKRVQLPE--GTQIIVNIGSVDIMRGKPLVQIEH  361 (468)
Q Consensus       284 ~~~liGds~~~~La~~~l~~~~~~~~~~~V~N~G~~isG~ts~~~~~rl~~~l~~~--PDlVvI~lGtND~~~g~s~e~f~~  361 (468)
                       +++|+||+.+..+.+.+++..|+.-.+.+|+|  |+|+++.++++||+.-...+  |.+|||++||||  ++.+.|++..
T Consensus      1714 dVlFvGDS~VQLmqQ~EiWRELFSPLHALNFG--IGGD~TqHVLWRL~NGELEnI~PKvvVvWVGTNN--H~~TAEeVag 1789 (1956)
T PTHR11852     1714 DVLFVGDSLVQLMQQYEIWRELFSPLHALNFG--IGGDTTQHVLWRLENGELENIRPKVVVVWVGTNN--HGHTAEEVAG 1789 (1956)
T ss_pred               ceeeecCCcccccccchhhhhcCcchhhhccc--cchhhhhHhhhhhhhcchhcccccceeEEeeCCcc--ccccHHHHHh
```

```
Q ss_pred                 HHHHHHHHHHHhC--CCEEEEEcCCCCCccccchhhHHHHHHHHHHHHHHHHhC---CCEEEECHHHHhhhcccccHhhcCCC
Q gi|45553317|re   362 DFRLLIKEMHNM--RLVPILTNLAPLGNYCHDKVLCDKIYRFNKFIRSECC---HLKVIDIHSCLINERGVVRFDCFQAS  436 (468)
Q Consensus       362 ~l~~LI~~lr~~--ga~VIL~t~~P~~~~~~~~~~~~~~i~~~N~~ir~~A~~---gv~fIDl~~~~~~~~~g~~~~~~~~~dD  436 (468)
                      .+++||..+.++  .++|||+++.|++..+.  ..+++..++|+.+++...    +|.|+|.+..|.-.+|.+-...|.|-
T Consensus      1790 GI~AIVqlin~rqPqAkviVLGLLPRGe~PN--PLR~KN~~VN~Ll~~~L~~l~~v~lLD~D~GFVHSDGtIS~hDMfDy 1867 (1956)
T PTHR11852     1790 GIEAIVQLINTRQPQAKVIVLGLLPRGEKPN--PLREKNAKVNELLRASLPGLPNVQLLDADPGFVHSDGTISHHDMFDY 1867 (1956)
T ss_pred               hHHHHHHHHHHhcCCCeeeecccccCCCC--ccccHHHHhhHHHHHHHhcCcCCCccccCCCCCcccCCcccCCcchhhccccc
```

```
Q ss_pred                 CcCCCCcHHHHHHHHHHHHHHHHHHHHHHHhc
Q gi|45553317|re   437 PRQVTGSKEPYLFWNKIGRQRVLQVIETSLE  467 (468)
Q Consensus       437 GvHPn~~~~Gy~~lA~~i~~~I~~~L~~~~~a  467 (468)
                       +|+|.   .||+.+.+.|-+.|.++|+++-+
T Consensus      1868 -LHLt~---~GY~~~CkpLH~Ll~qLLee~pe 1895 (1956)
T PTHR11852     1868 -LHLTR--LGYAKVCKPLHELLLQLLEETPE 1895 (1956)
T ss_pred               -cCCCc--cchhhhhHHHHHHHHHHhccCcc
```

No 90

>**PF14606** Lipase_GDSL_3: GDSL-like Lipase/Acylhydrolase family; PDB: **3SKV _B.**
Probab=99.63 E-value=4e-15 Score=140.77 Aligned_cols=166 Identities=12% Similarity=0.079 Sum_probs=0.0

```
Q ss_pred                 EEEEEcchhccCCCCCCCcccccccccchHHHHHHHHHHHHhhcCCCCceEEEeCCCcCCccHHHHHHHHHHHHhcCCCeEE
Q gi|45553317|re   263 TSGTYNDSLLTINSDYDAYLLDFPLMGDDFMLYLARMELKCRFRRHERVLQSGLCVSGLTINGARNRLKRVQLPEGTQII  342 (468)
Q Consensus       263 ~Iv~iGDSIT~G~~~~~~~~~~~~~~~liGds~~~~La~~~l~~~~~~~~~~~V~N~G~~isG~ts~~~~~~rl~~~l~~~PDlVv  342 (468)
                      +++++|+|||+|++...+..          +|+..++++++          ++.++|.|  +||.. .--....+-+....+|+++
T Consensus        3 ~~v~YGsSItqG~~Asrpg~--------~~~~~~~aR~l---------~~~~iNLG--fsG~~~le~~~a~~ia~~~a~~~~~   64 (178)
T PF14606_consen   3 RWVAYGSSITQGACASRPGM--------AYPAILARRL-------GLDVINLG--FSGNG-KLEPEVADLIAEIDADLIV   64 (178)
T ss_dssp             EEEEEE-TT-TTTT-SSGGG--------SHHHHHHHHH-------T-EEEEEE---TCCC-S--HHHHHHHHHS--SEEE
T ss_pred              eEEEECChhhcCCCCCCCcc--------cHHHHHHHHc-------CCCeEeee--eCccc-ccCHHHHHHHHhcCCCCEEE
```

```
Q ss_pred                 EEeeeccCCCCCCHHHHHHHHHHHHHHHHHHHhC--CCEEEEEcCCCCCccccchhhHHHHHHHHHHHHHHHHhC--------CC
Q gi|45553317|re   343 VNIGSVDIMRGKPLVQIEHDFRLLIKEMHNM--RLVPILTNLAPLGNYCHDKVLCDKIYRFNKFIRSECC--------HL  412 (468)
Q Consensus       343 I~lGtND~~~g~s~e~f~~~l~~LI~~lr~~--ga~VIL~t~~P~~~~~~~~~~~~~~i~~~N~~ir~~A--------gv  412 (468)
                      |.+|.|     .+.++|.+.+..+|+.+|+.  ++.|++++..+.............++|+++++..+        ++
T Consensus       65 ld~~~N-----~~~~~~~~~~~fv~~iR~~hP~tPIllv~~~~~~~~~~~~~~~~~~~~~~~~~~r~~v~~l~~~g~~nl  139 (178)
T PF14606_consen  65 LDCGPN-----MSPEEFRERLDGFVKTIREAHPDTPILLVSPIPYPAGYFDNSRGETVEEFREALREAVEQLRKEGDKNL  139 (178)
T ss_dssp             EEESHH-----CCTTTHHHHHHHHHHHHHHTT-SSS-EEEEE----TTTTS--TTS--HHHHHHHHHHHHHHHHHHHHTT-TTE
T ss_pred              EEeecC-----CCHHHHHHHHHHHHHHHHhCCCCCEEEEecCCcccccccCchHHHHHHHHHHHHHHHHHHHHHHHHHHhcCCCcE
```

```
Q ss_pred                 EEEECHHHHhhhcccccHhhcCCCCcCCCCcHHHHHHHHHHHHHHHHH
Q gi|45553317|re   413 KVIDIHSCLINERGVVRFDCFQASPRQVTGSKEPYLFWNKIGRQRVL  459 (468)
Q Consensus       413 ~fIDl~~~~~~~~g~~~~~~~~~dDGvHPn~~~~Gy~~lA~~i~~~I~  459 (468)
                      .|||-.+.+.+.+..     +.+.|+ +|||+  .||..||+.+.+.|+
T Consensus      140 ~~l~g~~llg~d~~~~~~~e~tvDg-vHP~D--~lG~~~~a~~l~~~ir  178 (178)
T PF14606_consen 140 YYLDGEELLGDDH-----EATVDG-VHPND--LGMMRMADALEPVIR  178 (178)
T ss_dssp             EEE-HHHCS-----------------------------------
T ss_pred              EEeCchhhcCccc-----cccccc-ccccc--cccccccccccCC
```

No 91

>**PF00657** Lipase_GDSL: GDSL-like Lipase/Acylhydrolase; InterPro: A variety of lipolytic enzymes with serine as part of the active site have been identified []. Members of this entry include; Aeromonas hydrophila lipase, Vibrio mimicus arylesterase, Vibrio parahaemolyticus thermolabile haemolysin, rabbit phospholipase (AdRab-B), and Brassica napus anter-specific proline-rich protein.; GO: 0016788 hydrolase activity, acting on ester bonds, 0006629 lipid metabolic process; PDB: **2WAO _A 2WAB _A 1V2G _A 1U8U _A 1JRL _A 1IVN _A 1J00 _A 1DEO _A 1K7C 1PP4 _A ....**
Probab=99.63 E-value=4.2e-15 Score=144.54 Aligned_cols=182 Identities=14% Similarity=0.035 Sum_probs=0.0

```
Q ss_pred                 EEEEcchhccCCCCCCCcccccccccchHHHHHHHHHHHHhhcCCCCceEEEeCCCcCCccc---------------HHHHHH
Q gi|45553317|re   264 SGTYNDSLLTINSDYDAYLLDFPLMGDDFMLYLARMELKCRFRRHERVLQSGLCVSGLT---------------INGARN  328 (468)
Q Consensus       264 Iv~iGDSIT~G~~~~~~~~~~~~~~~liGds~~~~La~~~l~~~~~~~~~~~V~N~G~~isG~t---------------s~~~~~  328 (468)
                      |++||||+|+|       .....-..+.+.+...+.........++.+.|.|  ++|.+                ......
T Consensus        1 i~~fGDS~td~-----~~~~~~~~~~~~~~~~~l~~~~~~~~~~~~~~~~~~~~n~a~~~G~~~~~~~~~~~~~~~~~~~  73 (234)
T PF00657_consen   1 IVVFGDSLTDG-----GGDSNGGGWPEGLANNLSSCLGANQRNSGVDVSNYA--ISGATSDGDLYNLWAQVQNISQQISR  73 (234)
T ss_dssp             EEEEESHHHT-----TTSSTTCTHHHHHHHHCHHCCHHHHHCTTEEEEEEE---TT--CC-HGGCCCCTCHHHHHHHHH
T ss_pred              CEEEeehhccc-----CCCCCCcchhhhHHHHHHhhccccccCCCCCCeeccc--cCCCccccccchhhHHHHHHHHhhc
```

```
Q ss_pred                 HHHHHHhcCCCeEEEEeeeccC-------CCCCCHHHHHHHHHHHHHHHHHhCCCE-----EEEEcCCC-----------
Q gi|45553317|re   329 RLKRVQLPEGTQIIVNIGSVDI-------MRGKPLVQIEHDFRLLIKEMHNMRLV-----PILTNLAP-----------  384 (468)
Q Consensus       329 rl~~~l~~~PDlVvI~lGtND~-------~~g~s~e~f~~~l~~LI~~lr~~ga~-----VIL~t~~P-----------  384 (468)
```

```
                       ........++|+|+|++|+||+        ....+.+.+.+.+.++|+.++..+++      .+++...|
T Consensus         74 ~~~~~~~~~~~lv~i~~G~ND~~~~~~~~~~~~~~~~~~~~~~~~~~~i~~l~~~~~~~~~~~~~~~~~~~~~~~~~~~~~~~~~~  153 (234)
T PF00657_consen    74 LLDSKSFYDPDLVVIWIGTNDYFNNRDSSDNNTSVEEFVENLRNAIKRLRSNGARLIIVANIVVINLPPIGCLPAWSSNN  153 (234)
T ss_dssp             HHHHHHHHTTSEEEEE-SHHHHSSCCSCSTTHHHHHHHHHHHHHHHHHHHHHTTTEEEEEEEEEEEEHHC-GGGSTTHHHTH
T ss_pred             ccccccCCcceEEEecccCcchhhcccchhhhhHhhHhhhhhhhhhhHHhccCCcccccccccccccccccccccccc


Q ss_pred             CCccccchhhHHHHHHHHHHHHHHHHHHh--------C--CCEEEECHHHHhhcccccH---hhcCCCCcCCCCcHHHHHHHH
Q gi|45553317|re  385 LGNYCHDKVLCDKIYRFNKFIRSEC--------C--HLKVIDIHSCLINERGVVRF---DCFQASPRQVTGSKEPYLFWN  451 (468)
Q Consensus       385 ~~~~~~~~~~~~~~i~~~N~~ir~~A--------~--gv~fIDl~~~~~~~~~g~~~~~-----~~~dDGvHPn~~~~Gy~~lA  451 (468)
                       ................||+.+++++         .  .+.++|++..+....+...+   .++..|++|||.  +||+++|
T Consensus        154 ~~~~~~~~~~~~~~~~~~~n~~l~~~~~~~~l~~~~~~~~~~~~v~~~D~~~~~~~~~~~~~~~~~~~~~~~~~~~D~~Hpt~--~g~~~iA  231 (234)
T PF00657_consen   154 KDSASCIERLNAIVAAFNSALREVAAQLRKDYPKGANVPYFDIYSIFSDMYGIQNPENDKYMFWDGVHPTE--KGHKIIA  231 (234)
T ss_dssp             TTTCTTHHHHHHHHHHHHHHHHHHHHHHHHCHHHHCTEEEEEHHHHHHHHHHHHGGHHHCBBSSSSB-H--HHHHHHH
T ss_pred             ccccccchhhHHHHHHHHHHHHHHhhhcccccccCCceEEEEHHHHHHHHhhhccCccccceeccCCCcCCCH--HHHHHHH


Q ss_pred             HHH
Q gi|45553317|re  452 KIG  454 (468)
Q Consensus       452 ~~i  454 (468)
                       +.|
T Consensus        232 ~~i  234 (234)
T PF00657_consen   232 EYI  234 (234)
T ss_dssp             HHH
T ss_pred             cCC
```

No 92

>2waa_A Acetyl esterase, xylan esterase, putative, AXE2C; carbohydrate binding, plant cell WALL degradation, hydrolase, cellulases; 1.80A {Cellvibrio japonicus}
Probab=99.62 E-value=3.4e-15 Score=156.41 Aligned_cols=182 Identities=13% Similarity=0.027 Sum_probs=0.0

```
Q ss_pred             CCCCCCccEEEEEcchhccCCCC----CCCccccccccchHHHHHHHHHHHhhcCCCCceEEEeCCCcCCcc--------
Q gi|45553317|re  255 MTPTPTILTSGTYNDSLLTINSD----YDAYLLDFPLMGDDFMLYLARMELKCRFRRHERVLQSGLCVSGLT--------  322 (468)
Q Consensus       255 ~~~~P~~~~Iv~iGDSIT~G~~~~----~~~~~~~~~~~~~liGds~~~~La~~~l~~~~~~~~~~V~N~G~~isG~t--------  322 (468)
                       +.+.+...||+|||||||.|...    ...+...+.-...+|...+++.+          +..+.+.+  ++|.+
T Consensus        126 ~~~~~~~~rrI~fiGDSIT~G~g~~~~~~~~~~~~~~~~~~~~~~~~~y~~~la~~L-------gad~~~v~--~sG~gv~~~~~~g~  196 (347)
T 2waa_A          126 PAPVLPQRKILVLGDSVTCGEAIDRVAGEDKNTRWWNARESYGMLTAKAL-------DAQVQLVC--WGGRGLIRSWNGK  196 (347)
T ss_dssp             SCCCCCSEEEEEEESTTTTTTTTCCTTSCCCGGGCCSTTSHHHHHHHHT-------TEEEEEEE--CTTCCSSCCTTSC
T ss_pred             cCCCCCCCcEEEEEchhhcCCCCCCCCCCCCCcccccccccHHHHHHHHh-------CCCcEEEe--eeceEEecCCCCC


Q ss_pred             --HHHHHHHHH------------HHHhcCCCeEEEEeeeccCCCCC-CHHHHHHHHHHHHHHHHHHhC--CCEEEEEcCCCC
Q gi|45553317|re  323 --INGARNRLK------------RVQLPEGTQIIVNIGSVDIMRGK-PLVQIEHDFRLLIKEMHNM--RLVPILTNLAPL  385 (468)
Q Consensus       323 --s~~~~~~rl~~~~~~~~~~~~~~~~~~~l~~~~PDlVvI~lGtND~~~g~~-s~e~f~~~~l~~LI~~lr~~~~--ga~VIL~t~~P~  385 (468)
                         ...+...+.            .....+||+|||++|+|..+.  +.+.|..+|..+|+.+++.  +++||++..++.
T Consensus        197 ~~~~~~~~~~~~~~~~~~~~~~~~~~~~~~~~~~~~~~~~~~~d~~~~~PDvVvI~lGtND~~~~~~~~~~~~~~~~~~y~~~i~~ir~~~~P~a~Iil~~~~~~~  276 (347)
T 2waa_A          197 TDDANLPDFYQFTLGDTGQAPQWDHHRYQPDLIISAIGTNDFSPGIPDRATYINTYTRFVRTLLDNHPQATIVLTEGAIL  276 (347)
T ss_dssp             SSSCCHHHHTTBSSCCSTTCCBCCGGGCCCSEEEECCCHHHHSSSCCCHHHHHHHHHHHHHHHHHHHCTTCEEEECCCSSC
T ss_pred             CCCCchhhhhhhcccccccccchhhccCCCEEEEEcccccCCCCCCCCcHHHHHHHHHHHHHHHHHHhCCCCeEEEEecCCC


Q ss_pred             CccccchhhHHHHHHHHHHHHHHHHHhC--CCEEEECHHHHhhcccccHhhcCCCCcCCCCcHHHHHHHHHHHHHHHHHHHH
Q gi|45553317|re  386 GNYCHDKVLCDKIYRFNKFIRSECC--HLKVIDIHSCLINERGVVRFDCFQASPRQVTGSKEPYLFWNKIGRQRVLQVIE  463 (468)
Q Consensus       386 ~~~~~~~~~~~~~~~~i~~~N~~ir~~A~--gv~fIDl~~~~~~~~~g~~~~~~~~~dDGvHPn~~~~Gy~~lA~~i~~~~I~~~L~  463 (468)
                              .......+++.++++++   +..++++....       ....+.+|  +|||.  +||++|+.|+.+.|+++|+|+
T Consensus        277 --------~~~~~~~~~~~~~~i~~~~~~~~~~~~~v~~~~~~~~~~~~-------~~~~gdd~HPn~~~Gh~~mA~~l~~~~I~~ilg  338 (347)
T 2waa_A          277 --------NGDKKAALVSYIGETRQQLHSNRVFYASSS-------HHPGDNSD-AHPTK--DQHAAMARELTPQLRQIMD  338 (347)
T ss_dssp             --------CHHHHHHHHHHHHHHHHHHHCCTTEEECCCC-------CCCBTTB-SSCCH--HHHHHHHHHHHHHHHHHHT
T ss_pred             --------CccHHHHHHHHHHHHHHHHHhCCCeEEEecCC-------CCCCCCCC-CCCCH--HHHHHHHHHHHHHHHHHHhC
```

No 93

>3bzwF00
Probab=99.60 E-value=9.6e-14 Score=139.25 Aligned_cols=184 Identities=17% Similarity=0.131 Sum_probs=0.0

```
Q ss_pred             CccEEEEEcchhccCCCCCCCccccccccccchHHHHHHHHHHHHhhcCCCCceEEEEeCCCcCCccHHHHHHHHHHHHhcC--
Q gi|45553317|re  260 TILTSGTYNDSLLTINSDYDAYLLDFPLMGDDFMLYLARMELKCRFRRHERVLQSGLCVSGLTINGARNRLKRVQLPE--  337 (468)
Q Consensus       260 ~~~~Iv~iGDSIT~G~~~~~~~~~~~~~~~~~liGds~~~~La~~~l~~~~~~~~~~V~N~G~~isG~ts~~~~~~rl~~~~l~~~--  337 (468)
                       ...+|+++|||||.+........       .|...|..++        +..+.|.|  ++|.++.+....+.......
T Consensus          6 ~~~~~~~~~~GDS~t~~~~~~~~~~~~~~~~~~~~~~~~~~~y~~~L~~~~~~~~~~~~~~~G~~~G~~~~~~~~~~~~~~~~~~~~~~~~  68 (255)
T 3bzwF00          6 KGKKVVVLGDSITDGNGVGDASK---------NYGQYLAERL--------GAEVINYG--ISGNTTNDLIEQLEQLKDEIKE  68 (255)
T ss_pred             CCCEEEEEccccCCCCCcchHH---------HHHHHHHHHh--------CCeEEEEe--eCCCCHHHHHHHHHHHHhccC


Q ss_pred             -CCeEEEEeeeccCCCCC----------------------------------CHHHHHHHHHHHHHHHHhC--CCEEEEEc
Q gi|45553317|re  338 -GTQIIVNIGSVDIMRGK----------------------------------PLVQIEHDFRLLIKEMHNM--RLVPILTN  381 (468)
Q Consensus       338 -PDlVvI~lGtND~~~g~~----------------------------------s~e~f~~~~l~~LI~~lr~~~~--ga~VIL~t  381 (468)
                        +|+|+|.+|+||+..+.                                   +...|...+..+|..+++.  .+.|+|++
T Consensus         69 ~~~~~~i~~G~ND~~~~~~~~~~~~~~~~~~~v~~~~~~~~~~~~~~~~~~~~~~~~~~~~~~~~~~~~~~~~~~~~~~~~~~~i~~~t  148 (255)
T 3bzwF00         69 KVDLVIIFLGTNDFLAGVPVGEWFDNTEEIVELARTEVKKKVVELRRVKDTDAKTFYGALNELIKQLKEKFPDAKIVLLT  148 (255)
T ss_pred             CCCeEEEEEEcCCCCCCCCCCCHhHHHHHHHHHHHHHHHHHhCCCCeEEecCCCCCCCHHHHHHHHHHHHHHHHHHHCcCCCcEEEEC


Q ss_pred             CCCCCc-----------ccchhhHHHHHHHHHHHHHHHHHhC--CCEEEECHH-HHhhhcccccHhh---cCCCCcCCCCcH
Q gi|45553317|re  382 LAPLGN-----------YCHDKVLCDKIYRFNKFIRSECC--HLKVIDIHS-CLINERGVVRFDC---FQASPRQVTGSK  444 (468)
Q Consensus       382 ~~P~~~~------------~~~~~~~~~~~~~i~~~N~~ir~~A~--gv~fIDl~~~~~~~~~g~~~~~~~~---~~~dDGvHPn~~~  444 (468)
                       +.+...            ..........+..+.++++|.  ++++||++. ............+   +..|++|||.
T Consensus        149 ~~~~~~~~~~~~~~~~~~~~~~~~~~~~~n~~~~~g~~~~~~~~~~~~~~~~~~~~~~~~~~~~~~~~~~~~~~~~~~~~~~~~~~~~~D~lHpn~--  226 (255)
T 3bzwF00        149 PLKRLEAKGGEKNVQPTEEEQNKLGETLKDYVKAIKEAAEELGVPVIDLYSKAGLNPAIDEQRELKKDYMTDGLHPNE--  226 (255)
T ss_pred             cCchhhhhCCcCCCCcccchhhcCCccHHHHHHHHHHHHHHHhCCCEEecccchhcchHhhHHHhhCcccCCCCCCH--


Q ss_pred             HHHHHHHHHHHHHHHHHHH
Q gi|45553317|re  445 EPYLFWNKIGRQRVLQVI  462 (468)
Q Consensus       445 ~Gy~~lA~~i~~~~I~~~L  462 (468)
                       +||+.+|..|...|...+
T Consensus        227 ~G~~~~a~~~~~~~~~~~~  244 (255)
T 3bzwF00        227 KGHELLAKELQKQLKALL  244 (255)
```

```
T ss_pred             hhHHHHHHHHHHHHHHHH
```

No 94

>**SUPFAM template** c.23.10 SGNH hydrolase (**52266**) SCOP seed sequence: d1esda_.
Probab=99.57 E-value=2e-13 Score=129.19 Aligned_cols=197 Identities=14% Similarity=0.106 Sum_probs=0.0

```
Q ss_pred             ccEEEEEcchhccCCCCCC-----CcccccccccchHHHHHHHHHHHHhhcCCCceEEEeCCCcCCccHHHHHHHHHHH--
Q gi|45553317|re  261 ILTSGTYNDSLLTINSDYD-----AYLLDFPLMGDDFMLYLARMELKCRFRRHERVLQSGLCVSGLTINGARNRLKRV--  333 (468)
Q Consensus       261 ~~~Iv~iGDSIT~G~~~~~-----~~~~~~~~liGds~~~~La~~~l~~~~~~~~~~~V~N~G~~isG~ts~~~~~rl~~~--  333 (468)
                      ..++++||||++.|.....     .....=..-+++|+..++. .+.........+.+.|   .+|.++.++.......
T Consensus         2 ~~~~v~lGDS~~~G~g~~~~~~~~~~~~~~C~r~~~~yp~~~~~~-~~~~~~~~~~~~~~--~~G~~~~~~~~~~~~~~~~~  78 (302)
T SUPFAM0046617     2 PLRYVALGDSYTAGPGAGPLLDAPGAEDGCLRSSNNYPALLAA-ALGLKGIPDVDLVNLA--CSGATTADVLNRLDGGLG  78 (302)
T ss_pred             CCCEEEEcccccccCCCccccCCCCCCccccCCCchHHHHHH-HhccccCCcceeeee--cCcccccccccccccccc


Q ss_pred             ----------HhcCCCeEEEEeeeccC-------------------------------------------CCCCCHHHH
Q gi|45553317|re  334 ----------QLPEGTQIIVNIGSVDI-------------------------------------------MRGKPLVQI  359 (468)
Q Consensus       334 ----------l~~~PDlVvI~lGtND~-------------------------------------------~~g~s~e~f  359 (468)
                                +..++++|+|.+|.||.                                           ..+...+.+
T Consensus        79 ~~~~~~~~~~~~~~~~~~~v~~~~~G~ND~~~~~~~~~~~c~~~~~~~~~~~~~~~~~~~~~~~~~~~~~~~~~~~~~~~~~~~  158 (302)
T SUPFAM0046617    79 EVDVLPQIDALNPDTDLVTLTIGGNDLGFADVLTACVLALLLLSLLAGLLVDLLSLGAGCGDALDAARDKTLRDLTLDAL  158 (302)
T ss_pred             ccchHHHHhhcCCCCCeEEEEecCCcchhhHHHHHHHHHHhhcchhhhhhhhhhhhccccchHHHHHHHhHhhhhhhhHHHH


Q ss_pred             HHHHHHHHHHHHHhC--CCEEEEEcCCCCCccccchh----------------------hHHHHHHHHHHHHHHHhC--CCE
Q gi|45553317|re  360 EHDFRLLIKEMHNM--RLVPILTNLAPLGNYCHDK----------------------VLCDKIYRFNKFIRSECC--HLK  413 (468)
Q Consensus       360 ~~~l~~LI~~lr~~--ga~VIL~t~~P~~~~~~~~---------------------~~~~~~i~~~N~~ir~~A~~~gv~  413 (468)
                      ..+|..+++.+++.  +++|+|++.+...+.....                       ...+....+|+.+++.|+  ++.
T Consensus       159 ~~~l~~~~~~~~~~~~~~~~P~~~~~~~~~~~~~~~~~~~~~~~~~~~~~~~~~~~~~~~~~~~~~~~~~~~~~~~~~~~~~~~a~~~~~~~  238 (302)
T SUPFAM0046617   159 AANLDEVLDAIRAAAPNARVVLVGYPTLFPPDGTVCLLSLLGLLKVLLLPLTQADLALLNDLVDALNAAIAKAAAAAGVT  238 (302)
T ss_pred             HHHHHHHHHHHHHHhCCCCeEEEeccccccCCCccccccccccccceeeeeccccHHHHHHHHHHHHHHHHHHHHHHHHHHHcCce


Q ss_pred             EEEChHHHhhhccccc----------------HhhcCCCCcCCCCcHHHHHHHHHHHHHHHHHHH
Q gi|45553317|re  414 VIDIHSCLINERGVVR----------------FDCFQASPRQVTGSKEPYLFWNKIGRQRVLQVI  462 (468)
Q Consensus       414 fIDl~~~~~~~~~~g~~~~----------------~~~~~~dDGvHPn~~~~Gy~~lA~~i~~~~I~~~L  462 (468)
                      |+|+...+.+...+..                ......+++|||.  .||..+|+.+.+.+.+.+
T Consensus       239 ~~~~~~~~~~~~~~~~~~~~~~~~~~~~~~~~~~~~~~~~~~~~~~~~HP~~--~~G~~~~~a~~~~~~~~~~~~  301 (302)
T SUPFAM0046617   239 FVDVAAAFRGHDACSAGDPWVNGVLLLSTLLLLGLLLADSFHPNA--AGYRALADALLDALEELL  301 (302)
T ss_pred             EeeccccccccccccCcccccccccceeehhccccccccccCCch--HHHHHHHHHHHHHHHHhhc
```

No 95

>**2w9x_A** AXE2A, CJCE2B, putative acetyl xylan esterase; carbohydrate esterase family 2, hydrolase; 2.00A {Cellvibrio japonicus}
Probab=99.55 E-value=2.4e-14 Score=151.59 Aligned_cols=207 Identities=12% Similarity=0.004 Sum_probs=0.0

```
Q ss_pred             hhhcccccccccccccCCcCCCCCCccEEEEEcchhccCCCCCCCccc-------ccccccchHHHHHHHHHHHHhhcC
Q gi|45553317|re  234 RHLGIFVHPFNGMNIMKRRHEMTPTPTILTSGTYNDSLLTINSDYDAYLL-------DFPLMGDDFMLYLARMELKCRFR  306 (468)
Q Consensus       234 ~~~~~~~~~~~~g~~~~~~~~~e~~~~P~~~~~Iv~iGDSIT~G~~~~~~~~~~-------~~~liGds~~~~La~~~l~~~~~~  306 (468)
                      ++.+.....++|...........+.+...||.|||||.|.+.......       ...-...+|...+++.+
T Consensus       115 tE~~~g~~~f~g~~~d~~~~~~~~~p~~~rrIefiGDSIT~GyG~~~~~~~~~~~~~~~~~~~~~n~~~sy~~~~a~~L------  188 (366)
T 2w9x_A          115 TETQSTSGRFLGFYTDPSAKPLALPKRKRQIEFIGDSFTVGYGNTSPSRECTDEELFKTTNSQMAFGPLTAKAF------  188 (366)
T ss_dssp             SCCSSCCEEECCC---------CCCCCCCEEEEEESHHHHTTTTTCSCSCCCHHHHHHHCCGGGSHHHHHHHHH------
T ss_pred             eccCCCCceEEEEEeCCCccccCCCCCCCEEEEEccccccCCCCCCccCcCcccccccccccHHHHHHHHh------


Q ss_pred             CCceEEEeCCCcCCccH----------HHHHHHHHHHhc----------CCCeEEEEeeeccCCCCCC-----------
Q gi|45553317|re  307 RHERVLQSGLCVSGLTI----------NGARNRLKRVQLP----------EGTQIIVNIGSVDIMRGKP-----------  355 (468)
Q Consensus       307 ~~~~V~N~G~~isG~ts--------------~~~~~rl~~~l~~---------~~PDlVvI~lGtND~~~g~s-----------  355 (468)
                       +..+.+.+  +||.++               ..+...+...+..           +||+|||++||+||+.+..
T Consensus       189 -~ad~~~a~~~SG~gv~~~~~g~~~~~~~~~~~y~~~~~~~~~~~~~~~~~~~PDvVvI~LGtND~~~~~~~~~~~~~~~~~~~~
                                                                                       265 (366)
T 2w9x_A          189 -DADYQINA--SSGFGIVRNYNGTSPDKSLLSLYPYTLNNPDQLYHNKHWKPQVIVIGLGTNDFSTALNDNERWKTREAL  265 (366)
T ss_dssp             -TCEEEEEE--CTTCCSSCCGGGCSTTCCHHHHTTBSSSSTTCBCCCTTCCCSEEEEECCHHHHSSCCCTTSSCCSHHHH
T ss_pred             -CCCceEEe--ccCCEEEcCCCCCCCCcchhhhhhhhccCcccccccCCCCEEEEeCcccccCCCcccccchhhH


Q ss_pred             HHHHHHHHHHHHHHHHHhC--CCEEEEEcCCCCCcccchhhHHHHHHHHHHHHHHHHhC-CCEEEEChHHHhhhcccccHhh
Q gi|45553317|re  356 LVQIEHDFRLLIKEMHNM--RLVPILTNLAPLGNYCHDKVLCDKIYRFNKFIRSECC-HLKVIDIHSCLINERGVVRFDC  432 (468)
Q Consensus       356 ~e~f~~~l~~LI~~lr~~--ga~VIL~t~~P~~~~~~~~~~~~~~~~~~i~~~N~~ir~~A~~gv~fIDl~~~~~~~~~~g~~~~~~~~~
                      .++|..+.|.++|+.+++.  +++|+|||++++.       ..+....++++++++.. +...++..     .....++
T Consensus       266 ~~~~~~~~y~~~i~~lr~~~~P~a~Iil~~~~~~~~~~~~~~~~~~~~~~~~~~~~i~~vv~~~~~~~~~~~~~~~~~~~~~~~~~~v~~~~~~~~~~~~~~~~~~d~  330 (366)
T 2w9x_A          266 HADYVANYVKFVKQLHSNNARAQFILMNSDQS--------NGEIAEQVGKVVAQLKGGGLHQVEQI-------VFKGLDY  330 (366)
T ss_dssp             HHHHHHHHHHHHHHHHHHCTTCEEEEEEESCG--------GGHHHHHHHHHHHHHHHHTTCCCEEEE-------EECCCCC
T ss_pred             HHHHHHHHHHHHHHHHhhCCCCeEEEEcCCCCc--------chHHHHHHHHHHHHHHHhCCCCeeeeec-------cccCCCC


Q ss_pred             cCCCCcCCCCcHHHHHHHHHHHHHHHHHHHHHHhc
Q gi|45553317|re  433 FQASPRQVTGSKEPYLFWNKIGRQRVLQVIETSLE  467 (468)
Q Consensus       433 ~~dDGvHPn~~~~Gy~~lA~~i~~~~I~~~L~~~~a  467 (468)
                      +.+|  +|||.  +|||++|||+.|.+.|++.+.-.++
T Consensus       331 ~gd~~HPn~~~Gh~~mA~~l~~~~I~~~~~g~W~~  362 (366)
T 2w9x_A          331 SGCH-WHPSA--NDDQLLANLLITHLQQKKGIWLE  362 (366)
T ss_dssp             CBGG-GBCCH--HHHHHHHHHHHHHHHHHTBTTSCC-
T ss_pred             CCcC-cCcCH--HHHHHHHHHHHHHHHHHHhhhhHhh
```

No 96

>**COG2845** Uncharacterized protein conserved in bacteria [Function unknown]
Probab=99.49 E-value=1.6e-12 Score=131.31 Aligned_cols=211 Identities=14% Similarity=0.079 Sum_probs=0.0

```
Q ss_pred             CCCCcCCCCCCCcccCCCCCCCcccccccccchhhcccccccccccccCCcCCCCCCCccEEEEEcchhccCCCCCCCCccc
Q gi|45553317|re  204 DAPEEPINLAPPGHQPSCRTQSQKTEPTENRHLGIFVHPFNGMNIMKRRHEMTPTPTILTSGTYNDSLLTINSDYDAYLL  283 (468)
Q Consensus       204 ~~~~~~~~~~~~~~~~~p~~~~~~~~~~~~~~~~~~~~~~~~g~~~~~~~~~~e~~~~P~~~~~Iv~iGDSIT~G~~~~~~~~~~~~
                      .+++.|..-++++-.|.+.....++..                          +....|+..+|+++|||+..|
T Consensus        82 ~s~~~p~~~~~~~~~~~~p~~~a~a~~~~~~~~~~~~~~~~~~~~~~~~~~~~~~~~~~~~~~~~~~~~~~~~~k~~~~a~kvLvvGDslm~g---------  129 (354)
```

```
T COG2845           82 PSLDMPRASAASEVAPRPGATAEPATT-----------------------IAAKSRDADKVLVVGDSLMQG---------  129 (354)
T ss_pred              CCCCCcccccccccCCCCCCCCCcccch-----------------------hhhhCCCCCEEEEechHHhhh---------


Q ss_pred              ccccccchHHHHHHHHHHHHhhcCCCceEEEeCCCcCCccHH-----HHHHHHHHHHhcC--CCeEEEEeeeccCCCCC--
Q gi|45553317|re  284 DFPLMGDDFMLYLARMELKCRFRRHERVLQSGLCVSGLTIN-----GARNRLKRVQLPE--GTQIIVNIGSVDIMRGK--  354 (468)
Q Consensus       284 ~~~liGds~~~~La~~~l~~~~~~~~~~~V~N~G~~isG~ts~-----~~~~rl~~~l~~~~--PDlVvI~lGtND~~~g~--  354 (468)
                          +++..-..|+.       ..++.|.+..  .+.....     ++...+.+.+...   |.+|||++|.||-..-.
T Consensus       130 ----la~gl~~al~t-------~~~i~i~~~s--n~SSGlvr~dYfdWpk~i~~~l~~~~~~~a~vVV~lGaND~q~~~~g  196 (354)
T COG2845        130 ----LAEGLDKALAT-------SPGITIVTRS--NGSSGLVRDDYFDWPKAIPELLDKHPKPAAVVVMLGANDRQDFKVG  196 (354)
T ss_pred              ----hHHHHHHHhcc-------CCCcEEEEee--cCCCCccccccccHHHHHHHHHhcCCccEEEEEecCCCHHhcccC


Q ss_pred              ----------CHHHHHHHHHHHHHHHHHHhCCCEEEEEcCCCCCccchhhHHHHHHHHHHHHHHHHhC--CCEEEECHHHHH
Q gi|45553317|re  355 ----------PLVQIEHDFRLLIKEMHNMRLVPILTNLAPLGNYCHDKVLCDKIYRFNKFIRSECC--HLKVIDIHSCLI  422 (468)
Q Consensus       355 ----------s~e~f~~~l~~LI~~lr~~ga~VIL~t~~P~~~~~~~~~~~~~~~~~~i~~~N~~ir~~A~--gv~fIDl~~~~~~  422 (468)
                                ..++|.+.+.++++.++.+..+|+|+++|++....    +++.+..+|.++++.++  +-.|||+++.|.
T Consensus       197 d~~~kf~S~~W~~eY~kRvd~~l~ia~~~~~~~V~WvGmP~~r~~~~----l~~dm~~ln~iy~~~vE~~~gk~i~i~d~~v  272 (354)
T COG2845        197 DVYEKFRSDEWTKEYEKRVDAILKIAHTHKVPVLWVGMPPFRKKK----LNADMVYLNKIYSKAVEKLGGKFIDIWDGFV  272 (354)
T ss_pred              CeeeecCchHHHHHHHHHHHHHHHHHHhcccCCcEEEeeCCCccccc----cchHHHHHHHHHHHHHHHHHHhCCeEEEEecccc


Q ss_pred              hhccc-----------ccHhhcCCCCcCCCCcHHHHHHHHHHHHHHHHHHHHHH
Q gi|45553317|re  423 NERGV-----------VRFDCFQASPRQVTGSKEPYLFWNKIGRQRVLQVIETS  465 (468)
Q Consensus       423 ~~~g~-----------~~~~~~~~~dDGvHPn~~~~Gy~~lA~~i~~~I~~~L~~~  465 (468)
                      ++.|.           ..-.+..+||+|.|.  +|-+.+|..+.+.|...++..
T Consensus       273 ~e~G~~f~~~~~~D~NGq~vrlR~~DGIh~T~-~~~Gkrkla~~~~~k~I~~~l~~a  324 (354)
T COG2845        273 DEGGKDFVTTGVDINGQPVRLRAKDGIHFTK--EGKRKLAFYLEKPIRAELETA  324 (354)
T ss_pred              ccCCceeEEeccccCCceEEEeccCCceech--hhHHHHHHHHHHHHHHhhhccc
```

No 97

>cd01846 fatty_acyltransferase_like Fatty acyltransferase-like subfamily of the SGNH hydrolases, a diverse family of lipases and esterases. The tertiary fold of the enzyme is substantially different from that of the alpha/beta hydrolase family and unique among all known hydrolases; its active site closely resembles the Ser-His-Asp(Glu) triad found in other serine hydrolases. Might catalyze fatty acid transfer between phosphatidylcholine and sterols.

Probab=99.48 E-value=2e-12 Score=131.17 Aligned_cols=187 Identities=12% Similarity=0.062 Sum_probs=0.0

```
Q ss_pred              EEEEEcchhccCCCCCC-----------CcccccccccchHHHHHHHHHHHHhhcCCCceEEEeCCCcCCccHHH------
Q gi|45553317|re  263 TSGTYNDSLLTINSDYD-----------AYLLDFPLMGDDFMLYLARMELKCRFRRHERVLQSGLCVSGLTING------  325 (468)
Q Consensus       263 ~Iv~iGDSIT~G~~~~~~~~-----------~~~~~~~~~~~liGds~~~~La~~~l~~~~~~~~~~~V~N~G~~isG~ts~~~------  325 (468)
                      ++++||||+++-+....           ++..+----|..|..+|++ .++..+  .....|.+  ++|+++..
T Consensus         1 ~l~vFGDS~sD~Gn~~~~~~~~~~~~~~~~~~~~~grfSnG~~w~d~la~-~lg~~~~--~~~g~NfA--~~GA~~~~~~~~~~~~~  75 (270)
T cd01846          1 RLVVFGDSLSDTGNIFKLTGGSNPPPSPPYFGGRFSNGPVWVEYLAA-TLGLSG--LKQGYNYA--VGGATAGAYNVPPY  75 (270)
T ss_pred              CEEEeCCCCccCCccccccCCCCCCCCCCccCCCCCCccHHHHHHH-HhCCCc--CCCCceEe--ecCccccCCcCccc


Q ss_pred              ------HHHHHHHHHhcCC------CeEEEEeeeccCCCC--------CCHHHHHHHHHHHHHHHHHhCCCE-EEEEcCCC
Q gi|45553317|re  326 ------ARNRLKRVQLPEG------TQIIVNIGSVDIMRG--------KPLVQIEHDFRLLIKEMHNMRLV-PILTNLAP  384 (468)
Q Consensus       326 ------~~~rl~~~l~~~~P------DlVvI~lGtND~~~g--------~s~e~f~~~l~~LI~~lr~~ga~~VIL~t~~P  384 (468)
                            +..+++..+....      ++++|++|+|+...        ...+++...+.+.|++|.+.|++ |+|++++|
T Consensus        76 ~~~~~~~l~~Qv~~f~~~~~~~~~~~~~~~~~l~~i~~G~ND~~~~~~~~~~~~~~~~~~~~~~~~~~~~~~~~~~i~~L~~~Gar~i~v~~~~p~  155 (270)
T cd01846         76 PPTLPGLSDQVAAFLAAHKLRLPPDTLVAIWIGANDLLNALDLPQNPDTLVTRAVDNLFQALQRLYAAGARNFLVLNLPD  155 (270)
T ss_pred              CCCCCCHHHHHHHHHHhccccCCCCcEEEEEecccHHhhhcccccchhhHHHHHHHHHHHHHHHHHHHcCCCEEEEecCCC


Q ss_pred              CCccccchh--------hHHHHHHHHHHHHHHHhC---------CCEEEECHHHHhhhc---------------------
Q gi|45553317|re  385 LGNYCHDK--------VLCDKIYRFNKFIRSECC---------HLKVIDIHSCLINER---------------------  425 (468)
Q Consensus       385 ~~~~~~~~~~~~~~~~~~~~~~~~~~~~~~~~~~i~~~N~~ir~~A~~~~~~~~~~~~~~gv~fIDl~~~~~~~~~~~~~~~~~~~~~~~~~~~~~~~~~~~~  425 (468)
                      +...+...        ..+.....||+.+++...         .+.++|++..+.+.-
T Consensus       156 ~~~~P~~~~~~~~~~~~~~~~~~~~~~~~~~~~~N~~L~~~l~~l~~~~~~~~~~~i~~D~~~~~~~~i~~np~~yGf~~~~~~~C~~~~~~~~  235 (270)
T cd01846        156 LGLTPAFQAQGDAVAARATALTAAYNAKLAEKLAELKAQHPGVNILLFDTNALFNDILDNPAAYGFTNVTDPCLDYVYSY  235 (270)
T ss_pred              CCCCcccccCccchHHHHHHHHHHHHHHHHHHHHHHhhCCCCeEEEEhHHHHHHHHHhHhhhcCCCCCCcchhhhCCCCC


Q ss_pred              -----ccccHhhcCCCCcCCCCcHHHHHHHHHHHH
Q gi|45553317|re  426 -----GVVRFDCFQASPRQVTGSKEPYLFWNKIGRQ  456 (468)
Q Consensus       426 -----g~~~~~~~~~~dDGvHPn~~~~Gy~~lA~~i~~  456 (468)
                           .....+++..|++|||.  ++|+++|.+.+
T Consensus       236 ~~~~~~~c~~~~~~~~~y~fwD~~HpT~~-~~~~~~~~~iA~~~~~~~  269 (270)
T cd01846        236 SPREACANPDKYLFWDEVHPTT--AVHQLIAEEVAA  269 (270)
T ss_pred              CCcCcCCCccccEEEecCCCCCH--HHHHHHHHHHHHh
```

No 98

>cd01824 Phospholipase_B_like Phospholipase-B_like. This subgroup of the SGNH-family of lipolytic enzymes may have both esterase and phospholipase-A/lysophospholipase activity. It's members may be involved in the conversion of phosphatidylcholine to fatty acids and glycerophosphocholine, perhaps in the context of dietary lipid uptake. Members may be membrane proteins. The tertiary fold of the SGNH-hydrolases is substantially different from that of the alpha/beta hydrolase family and unique among all known hydrolases; Its active site closely resembles two of the three components of typical Ser-His-Asp(Glu) triad from other serine hydrolases.

Probab=99.41 E-value=1.5e-11 Score=126.48 Aligned_cols=198 Identities=11% Similarity=0.018 Sum_probs=0.0

```
Q ss_pred              CCCccE-EEEEcchhccCCC--------CCCCcccccccccch-----------HHHHHHHHHHHhhcCCCc-----eEE
Q gi|45553317|re  258 TPTILT-SGTYNDSLLTINS--------DYDAYLLDFPLMGDD-----------FMLYLARMELKCRFRRHE-----RVL  312 (468)
Q Consensus       258 ~P~~~~~~Iv~iGDSIT~G~~~~~~~~~~~~~~~~~~~~~~~~~~~~~~~~liGds~~~~~~~~~~~~~~~~~~~~~~~~~La~~~l~~~~~~~~~~~~~~~~~~~V~  312 (468)
                      .|.-++ |+++|||+|.|..        ....+....-.+|.+           +...+...+.+...+.+.     ...
T Consensus         6 rP~DI~viaAlGDSltag~ga~~~~~~~~~~~~~~~~~e~rg~s~~~Ggd~~~~~~~~TLpNiLk~fnp~l~G~s~g~~~~~~~~~~~~~~~~~~  85 (288)
T cd01824          6 RPGDIKVIAALGDSLTAGNGAGSANNLDLLTEYRGLSWSIGGDSTLRGLTTLPNILREFNPSLYGYSVGTGDETLPDSGF  85 (288)
T ss_pred              chhhCeEEEEcchHhhccCCcccccccccccCCCEEEeCCCCcccchhHHHHHHHHHhCCccccccCCCCcccCCcccccee


Q ss_pred              EeCCCcCCccHHHHHHHHHHHHHHhc-----------CCCeEEEEeeeccCCCCCC------HHHHHHHHHHHHHHHHHHhC--
Q gi|45553317|re  313 QSGLCVSGLTINGARNRLKRVQLP-----------EGTQIIVNIGSVDIMRGKP------LVQIEHDFRLLIKEMHNM--  373 (468)
Q Consensus       313 N~G~~isG~ts~~~~~~~~rl~~~l~~~~~~~~~~~~~~~~~~~~~~~~PDlVvI~lGtND~~~g~~s~~~~~~~~~~~~e~f~~~l~~LI~~lr~~~~--  373 (468)
                      |.|  ++|+++.++..+++.++..           ++++|+|++|+|++....      ++.|.++++++++.+++.
T Consensus        86 N~A~~v~Ga~s~dL~~Qa~~lv~~~k~~~~~id~~~dwKlVtI~IG~NDlC~~c~~~~~~~~~~~~~~~~~~~~~~~l~~aL~~Lr~~~~P  163 (288)
T cd01824         86 NVA--EPGAKSEDLPQQARLLVRRMKKDPRVDFKNDWKLITIFIGGNDLCSLCEDANPGSPQTFVKNLRKALDILRDEVP  163 (288)
```

```
 T ss_pred              eec--ccCccHHHHHHHHHHHHHHHHhhccccccccCccEEEEEEccccccccCCCccccCHHHHHHHHHHHHHHHHHHHCC


 Q ss_pred              CCEEEEEcCCCCC---------------------------cccchhhHHHHHHHHHHHHHHHHhC---------CCEEEECh
 Q gi|45553317|re  374 RLVPILTNLAPLG---------------------------NYCHDKVLCDKIYRFNKFIRSECC---------HLKVIDIH  418 (468)
 Q Consensus       374 ga~VIL~t~~P~~---------------------------~~~~~~~~~~~~i~~~N~~ir~~A~---------gv~fIDl~  418 (468)
                        .+.|+|++++...                          .........+...+|++.++++++          .|.+-.+.
 T Consensus       164 ra~Vnlv~~~~~~~~l~~~~~~~~~~~C~~~~~~~~~C~C~~~~~~~~~~~~~~~~~~~~~~y~~~~~~~i~~~~~~~~~~~~f~Vv~qPf~  243 (288)
 T cd01824         164 RAFVNLVGLLNVASLRSLTKKPLQCETLLAPECPCLLGPTENSYQDLKKFYKEYQNEVEEIVESGEFDREDFAVVVQPFF  243 (288)
 T ss_pred              CcEEEEEcCCChHHHHHhcCCCcccccccCcCCCcCCCccchHHHHHHHHHHHHHHHHHHHHhccccccCCCcEEEeccc


 Q ss_pred              HHHhhhc---ccccHhhcCCCCcCCCCcHHHHHHHHHHHHHHHHHH
 Q gi|45553317|re  419 SCLINER---GVVRFDCFQASPRQVTGSKEPYLFWNKIGRQRVLQ  460 (468)
 Q Consensus       419 ~~~~~~~~~~~---g~~~~~~~~~~~dDGvHPn~~~~Gy~~lA~~i~~~~I~~  460 (468)
                       .......    | .+..+++.|++|||.  +||..+|+.++..+.+
 T Consensus       244 ~~~~~~p~~~~~g-~d~~~~~~~~DcfHps~--~gha~~A~~lwn~mle  285 (288)
 T cd01824         244 EDTSLPPLPDG-PDLSFFSPDCFHFSQ--RGHAIAANALWNNLLE  285 (288)
 T ss_pred              ccccccccCC-CCchhccCCCCCcCH--HHHHHHHHHHHHHHhhc
```

No 99

>**TIGR01167** TIGR01167 LPXTG cell wall anchor domain; InterPro:
This LPXTG motif-containing region is

found at the C terminus of many surface proteins of Streptococcus and Streptomyces species. Cleavage between the Thr and Gly by sortase or a related enzyme leads to covalent anchoring at the new C-terminal Thr to the cell wall. Proteins that do not lie at the C terminus or are not found in Gram-positive bacteria are probably false-positive. A common feature of proteins containing this domain appears to be a high proportion of charged and zwitterionic residues immediately upstream of the LPXTG motif.

.

Probab=99.10 E-value=1.4e-13 Score=6.78 Aligned_cols=9 Identities=11% Similarity=0.460 Sum_probs=0.0

```
 Q ss_pred              CeEEEEeee
 Q gi|45553317|re  339 TQIIVNIGS  347 (468)
 Q Consensus       339 DlVvI~lGt  347 (468)
                        +.+++.+|.
 T Consensus        10 ~~~~~~~~~~G~   18 (34)
 T TIGR01167        10 NSLLLLLGL   18 (34)
```

No 100

>**template** PERIPLASMIC BROAD-SPECIFICITY ESTERASE/LIPASE/PROTEASE.
Probab=99.37 E-value=3.4e-12 Score=118.26 Aligned_cols=178 Identities=17% Similarity=0.112 Sum_probs=0.0

```
 Q ss_pred              EEEEEcchhccCCCCCCCccccccccchHHHHHHHHHHHHHhhcCCCCceEEEeCCCcCCccHHHHHHHHHHHHHhcC-CCeE
 Q gi|45553317|re  263 TSGTYNDSLLTINSDYDAYLLDFPLMGDDFMLYLARMELKCRFRRHERVLQSGLCVSGLTINGARNRLKRVQLPE-GTQI  341 (468)
 Q Consensus       263 ~Iv~iGDSIT~G~~~~~~~~~~~~~~~~liGds~~~~La~~~l~~~~~~~~~~~~~V~N~G~~isG~ts~~~~~~rl~~~l~~~~-PDlV  341 (468)
                        +++++|||++.|....          ..+.|+..+...+...   .....++|.|   +||+|+...+.|+...+... ++.|
 T Consensus        38 ~~~~~~Gds~~ag~g~~----------~~~~~~~~~~~l~~~~~~~~~~~--g~~~~~~~na~--~sG~t~~~g~~rl~~~~~~~~~~~~~~~~  104 (224)
 T PTHR30383        38 TLLVVGDSLSAGLGLD----------ASEAFPALLEKALAEK--GIDYRVVNAG--ISGDTSSGGLARLPWLLAEEKPDLV  104 (224)
 T ss_pred              hhheehhhHHHhhccc----------hhHHHHHHHHHHHHHHh--hhhhhheeee--ecccchhhhHHHHHHHHHhhccccee


 Q ss_pred              EEEeeeccCCCCCCHHHHHHHHHHHHHHHHHhCCCCEEEEEcCCCCCccccchhhHHHHHHHHHHHHHHHHhC--CCEEEEChH
 Q gi|45553317|re  342 IVNIGSVDIMRGKPLVQIEHDFRLLIKEMHNMRLVPILTNLAPLGNYCHDKVLCDKIYRFNKFIRSECC--HLKVIDIHS  419 (468)
 Q Consensus       342 vI~lGtND~~~g~s~e~f~~~l~~LI~~lr~~ga~VIL~t~~P~~~~~~~~~~~~~~~~~~~~~~i~~~N~~ir~~A~~--gv~fIDl~~  419 (468)
                        ++.+|.||..+|..+....+++.+++..+++.+..++++++.-..++      .......|+...-++++  .+.++.++-
 T Consensus       105 ~~~~G~nd~lrG~~p~~~~~~~~l~~~~~~~~~~~~~~~~~~~~~~~l~~~~~~~~~~~n~-----G~~y~~~f~~~~p~~a~~~~~~~~l~pff~  179 (224)
 T PTHR30383       105 VVELGANDGLRGIDPAQTEKNLAEIVQKVKARGAKVLLLGIKLPPNY-----GRRYAEAFSAVYPKVAKEEDVPLVPFFL  179 (224)
 T ss_pred              EeeecccchhhhhcchhhhhhhhhhhhhhhhhhhhhhhHhchhhhhhhhhhhhhhhh-----hhhhhhhhhhhhhhhhhhhhhhhhhhhhhh


 Q ss_pred              HHhhhcccccHhhcCCCCcCCCCcHHHHHHHHHHHHHHHHHHHHHH
 Q gi|45553317|re  420 CLINERGVVRFDCFQASPRQVTGSKEPYLFWNKIGRQRVLQVIET  464 (468)
 Q Consensus       420 ~~~~~~~~~g~~~~~~~~~~~~~dDGvHPn~~~~Gy~~lA~~i~~~~I~~~~L~~  464 (468)
                        .-....    ..+.+.||+||+.  +|+..+++.+.+.+..++..
 T Consensus       180 ~gv~~~~----~~~~~q~dGihp~~--~~~~~~~~~~~~~~~~~~~~~~~~~~~~~~~~~~  218 (224)
 T PTHR30383       180 EGVAGV----PGLNQADGIHPTA--EGQPILADNVLPTLQPLLKN  218 (224)
 T ss_pred              HHHHHH----Hhhhhhcccchhh--HHHHHHHHHHHHHHHHHHHHhh
```

Done!

Please cite as appropriate:

***HHpred*: Söding, J. (2005) Protein homology detection by HMM-HMM comparison. Bioinformatics 21: 951-960.**

PSIPRED: Jones, D.T. (1999) Protein secondary structure prediction based on position-specific scoring matrices. JMB 292: 195-202.

PDB: Bourne, PE. *et al.* (2004) The distribution and query systems of the RCSB Protein Data Bank. NAR 32: D223.

Interpro: Mulder NJ, Apweiler R, Attwood TK, Bairoch A, Bateman A, Binns D, Bradley P, Bork P, Bucher P, Cerutti L, Copley R, Courcelle E, Das U, Durbin R, Fleischmann W, Gough J, Haft D, Harte N, Hulo N, Kahn D, Kanapin A, Krestyaninova M, Lonsdale D, Lopez R, Letunic I, Madera M, Maslen J, McDowall J, Mitchell A, Nikolskaya AN, Orchard S, Pagni M, Ponting CP, Quevillon E, Selengut J, Sigrist CJ, Silventoinen V, Studholme DJ, Vaughan R, Wu CH. (2005) InterPro, progress and status in 2005. NAR 33: D201-205.

Pfam: Bateman A, Coin L, Durbin R, Finn RD, Hollich V, Griffiths-Jones S, Khanna A, Marshall M, Moxon S, Sonnhammer EL, Studholme DJ, Yeats C, Eddy SR. (2004) The Pfam protein families database. NAR 32: D138-141.

CDD: Marchler-Bauer A, Anderson JB, Cherukuri PF, DeWeese-Scott C, Geer LY, Gwadz M, He S, Hurwitz DI, Jackson JD, Ke Z, Lanczycki CJ, Liebert CA, Liu C, Lu F, Marchler GH, Mullokandov M, Shoemaker BA, Simonyan V, Song JS, Thiessen PA, Yamashita RA, Yin JJ, Zhang D, Bryant SH. (2005) CDD: a Conserved Domain Database for protein classification.. NAR 33: D192-196.

COG: Tatusov RL, Fedorova ND, Jackson JD, Jacobs AR, Kiryutin B, Koonin EV, Krylov DM, Mazumder R, Mekhedov SL, Nikolskaya AN, Rao BS, Smirnov S, Sverdlov AV, Vasudevan S, Wolf YI, Yin JJ, Natale DA. (2003) The COG database: an updated version includes eukaryotes. BMC Bioinformatics 4: 41.

PANTHER: Mi H, Lazareva-Ulitsky B, Loo R, Kejariwal A, Vandergriff J, Rabkin S, Guo N, Muruganujan A, Doremieux O, Campbell MJ, Kitano H, Thomas PD. (2005) The PANTHER database of protein families, subfamilies, functions and pathways. NAR 33: D284-288.

TIGRFAMs: Haft DH, Selengut JD, White O. (2003) The TIGRFAMs database of protein families. NAR 31: 371-373.

Superfamily: Madera M, Vogel C, Kummerfeld SK, Chothia C and Gough J. (2004) The SUPERFAMILY database in 2004: additions and improvements. NAR 32: D235-D239.

© 2008-2014, **Dept. of Protein Evolution at the Max Planck Institute for Developmental Biology**, Tübingen Release-2.18.1
