# Supplementary material for: Functional equivalence of germ plasm organizers
Source: PLoS Genet. 2018 Nov 6;14(11):e1007696. doi: 10.1371/journal.pgen.1007696 (PMC6219760; doi:10.1371/journal.pgen.1007696)
Supplement: S4 Table — (DOCX) [file pgen.1007696.s009.docx]

**Table S4: Plasmids and Primers related to Experimental Procedures.**

**Plasmids**

| Name | Backbone | Insert | Cloning strategy |
| --- | --- | --- | --- |
| **pCS2^+^ buc-eGFP** | pCS2^+^ | *buc-egfp* | (1) |
| **pCS2^+^ buc(1-361)-eGFP**  **pCS2^+^ buc(1-601)-eGFP** | pCS2^+^  pCS2^+^ egfp | *buc^p43^-egfp*  *buc^p106^-egfp* | *buc^p43^* sequence was amplified from pCS2^+^buc-eGFP, using the primers ClaI_buc_for, Buc-p43_XbaI_rev. The PCR product was cut with ClaI/XbaI and ligated into ClaI/XbaI digested pCS2^+^ buc-eGFP releasing wt *buc*.  *buc^p106^* sequence was amplified from pCS2^+^buc-eGFP, using the primers BamHI_bucp106_for, Buc-p106_XbaI_rev. The PCR product was inserted into precut (BamHI and XbaI) pCS2^+^ eGFP by In-Fusion cloning. |
| **pCS2^+^ osk(139-468)-eGFP** | pCS2^+^ | *osk-egfp* | *osk* was amplified from pBS-sOsk (gift from A. Eprussi, EMBL Heidelberg, Germany) using the primers Osk_BamHI_fw and Osk_XbaI_rev. The PCR product was cut with BamHI/XbaI and ligated into BamHI/XbaI digested pCS2^+^-eGFP |
| **pCS2^+^ osk(139-253)-eGFP** | pCS2^+^ | *osk^084^-egfp* | *osk^084^* sequence was amplified from pCS2+ osk-eGFP, using the primers Osk_BamHI_fw, Osk-084_XbaI_rev. The PCR product was cut with BamHI/XbaI and ligated into BamH/XbaI digested pCS2+ osk-eGFP releasing wt *osk*. |
| **pCSDestEx Hermes**  **pCSDestEx Vasa**  **pCS2^+^ bucVC**  **pCS2^+^ vasaVN**  **pGCSMyc-Myl12.2**  **pGCS-Ziwi-GFP**  **pGCs-eGFP-Vasa**  **pCS2+hFUS**  **pCS+mGFP**  **pCS2+Buc-mGFP**  **pCS2+Osk-mGFP**  **pDONROsk**  **pCSDest2Osk-Cherry** | pCSDEST  pCSDEST  pCS2^+^  pCS2^+^  pCS2^+^  pCS2+  pCS2+  pCS2+  pCS2+  pCS2+mGFP  pCS2+mGFP  pDONR221  pCSDest2 | *hermes*  *vasa*  *bucVC*  *vasaVN*  *myl12.2*  *ziwi-GFP*  *eGFP-Vasa*  *hFUS*  *monomeric GFP*  *buc*  *osk*  *osk*  *osk cherry* | *hermes* was amplified from pCS2^+^GFP-hermes with attB1 and attB2 sites on the forward and reverse primers, respectively. Using the Gateway system it was recombined into pDONR221 vector and then later into pCSDEST vector (2).  *vasa* was amplified from pBS vasa with attB1 and attB2 sites on the forward and reverse primers, respectively. Using the Gateway system it was recombined into pDONR221 vector and then later into pCSDEST vector.  *buc* was amplified from pCS2^+^ and inserted N-terminal to the Venus half in the pCS2^+^VC plasmid (3) using In-Fusion cloning.  *vasa* was amplified from pCSDestExVasa and inserted N-terminal to the Venus half in the pCS2^+^VN plasmid using Infusion cloning.  Non-muscle myosin light chain 12.2 was amplified from cDNA and cloned into the pGSNMyc plasmid (4) using Gateway cloning.  Ziwi was amplified from pCS2+Ziwi using forward and reverse primers including attB1 and attB2 sites, respectively. The PCR product was first recombined into pDONR221 and then into pGCSC6-eGFP destination vector (addgene catalog #85729) using Gateway cloning (4).  pDONR221-Vasa was recombined into pGCS N6-eGFP destination vector (addgene catalog # 85723) using Gateway cloning.  hFUS was amplified from pCS2+hFUS-eGFP using hFUSfwd and -rev primers. pCS2+ plasmid was linearized with BamHI and XbaI restriction enzymes. The PCR product was ligated into linearized vector by In-Fusion cloning.  mGFP was amplified from pmEGFP-1 (addgene catalog #36409) using pmEGFP-fwd and -rev primers and inserted into XbaI digested pCS2+ by In-Fusion cloning.  Buc was amplified from pCS2+Buc with primers Buc_in_fusion_BamHI-fwd and -rev and inserted into BamHI linearized pCS2+mGFP by In-Fusion cloning.  Osk was amplified from pCS2+Osk with primers osk_in_fusion_BamHI-fwd and -rev and inserted into BamHi linearized pCS2+mGFP by In-Fusion cloning.  osk was amplified from pCS2+Osk with primers osk _gateway-fwd and -rev and inserted into pDONR221 using Gateway cloning.  pDONROsk and p3mCherry (2) were used as Donor-vectors to insert into pCSDest2 by multisite gateway cloning. |

Other plasmids were pCS2^+^ziwi (gift from R. Ketting. IMB, Mainz, Germany), pCS2^+^VC, pCS2^+^VN, pCS2^+^XveloI (gifts from S. Richts, K. Henningfeld, M. Claußen & T. Pieler, GZMB Göttingen, Germany), pBSvasa, pSP64gfp-nos-3’UTR and pCS2+cherry-nos-3´UTR (gifts from E. Raz, ZMBE, Münster, Germany), pCS2^+^GFP-hermes (gift from K. Inoue, Kobe University, Japan), pCS2+Velo-GFP, pCS2+hFus-GFP (gifts from E. Boke, CRG Barcelona, Spain). All plasmids were verified by sequencing.

**Primers**

| Name | Sequence (5’ to 3’ direction) | Purpose |
| --- | --- | --- |
| ClaI_buc-fwd | GGGATCGATAATGTGGATCTCTGGAAACAG | pCS2^+^ plasmids |
| Buc-p43_XbaI-rev | GCTCTAGAGCTGTAGGAATAAGCACTGCC | pCS2^+^ plasmids |
| Osk_BamHI-fwd | GGGGGATTCATGACCATCATCGAGAGCAAC | pCS2^+^ plasmids |
| Osk_XbaI-rev | GGGTCTAGAATACTCCAGACTCGTTTCAAT | pCS2^+^ plasmids |
| Osk-084_XbaI-rev  BamH1_bucp106-fwd  Buc-p106_XbaI-rev  Cherry-fwd  Chery-rev  GFP-fwd  GFP-rev | GCTCTAGATGGTATGTTCTCCAGGGACGG  TCTTTTTGCAGGATCATGGAAGGAATAAATAACAATTCACAACCAATGGGAG TGCTCACCATTCTAGAAGTCGACCTTCTCAATGGAGT  CAGACCGCCAAGCTGAA  CATGGTCTTCTTCTGCATTA  ACCATCTTCTTCAAGGACGACGG  GCCGTTCTTCTGCTTGTCGGCC | pCS2^+^ plasmid  pCS2^+^ plasmid    Cherry-nos RNA detection  GFP-SV40-RNA detection |
| 18SrRNA fwd (human)  18SrRNA-rev (human)  18SrRNA-fwd (zebrafish)  18SrRNA-rev (zebrafish)  ef1α-fwd  ef1α-rev  nanos-3'-UTR-fwd  nanos-3'-UTR-rev  SV40-3'-UTR-fwd  SV40-3'-UTR-rev  attB1 hermes  attb2 hermes  attB1 vasa  attB2 vasa  pDONR-Ziwi-fwd  pDONR-Ziwi-NO STOP-rev  hFud-fwd (in fusion)  hFus-rev (in-fusion)  pmEGFP-fwd  pmEGFP-rev  Buc_in_fusion_BamHI-fwd  Buc_in_fusion_BamHI-rev  Osk_in_fusion_BamHI-fwd  Osk_in_fusion_BamHI-rev  sOSK_gateway-fwd  sOSK_gateway-rev | AACTGAGGCCATGATTAA  GGAACTACGACGGTATCTGA  CGGCTACCACATCCAAGGAA  GCTGGAATTACCGCGGCT  CTTCTCAGGCTGACTGTGC  CCGCTAGCATTACCCTCC  CTACACCATCGTGGAACAG  GGAGCATCAATGTCCGC  CTACACCATCGTGGAACAG  AGCAATAGCATCACAAATTTCAC  GGGGACAAGTTTGTACAAAAAAGCAGGCTTAATGAGTGTCAAGTCCGAC  GGGGACCACTTTGTACAAGAAAGCTGGGTATTAACAGAACTGTCGGGA  GGGGACAAGTTTGTACAAAAAAGCAGGCTTAATGGATGACTGGGAGGAA  GGGGACCACTTTGTACAAGAAAGCTGGGTATTATTCCCATTCCTCATC  GGGGACAAGTTTGTACAAAAAAGCAGGCTTAATGACAGGACGAGCAAGAGCA  GGGGACCACTTTGTACAAGAAAGCTGGGTTCAGGTAATACAGGAAGTCATCCAGATTCATG  TCTTTTTGCAGGATCATGGCCTCAAACGATTATACC  TCACTATAGTTCTAGCTAATACGGCCTCTCCCTGC  AGGCCTCTCGAGCCTCTAGATGGTGAGCAAGGGCGAGGA  CGACTCACTATAGTTCTAGTTACTTGTACAGCTCGTCCAT  TTGTTCTTTTTGCAGGATCATGGAAGGAATAAATAACAATTCACAACC  TCGAATCGATGGGATCACATTTTAAACTGCTCAACATACCTC  TTGTTCTTTTTGCAGGATCATGACCATCATCGAGAGCAAC  TCGAATCGATGGGATCGTGGTATGTTCTCCAGGGACGG  GGGGACAAGTTTGTACAAAAAAGCAGGCTATATGACCATCATCGAGAGCAAC  GGGGACCACTTTGTACAAGAAAGCTGGGTAGTGGTATGTTCTCCAGGGACGG | 18SrRNA detection  18SrRNA detection    ef1α-RNA detection  nos-RNA detection  SV40-RNA detection  hermes cloning    vasa cloning    Ziwi-eGFP cloning    pCS2+ hFus    pCS2+mGFP    pCS2+Buc-mGFP    pCS2+Osk-mGFP    pDONROsk |

1. Bontems F, Stein A, Marlow F, Lyautey J, Gupta T, Mullins MC, et al. Bucky ball organizes germ plasm assembly in zebrafish. Curr Biol. 2009;19(5):414-22.

2. Villefranc JA, Amigo J, Lawson ND. Gateway compatible vectors for analysis of gene function in the zebrafish. Dev Dyn. 2007;236(11):3077-87.

3. Harvey SA, Smith JC. Visualisation and quantification of morphogen gradient formation in the zebrafish. PLoS Biol. 2009;7(5):e1000101.

4. Wang HY, Li Y, Xue T, Cheng N, Du HN. Construction of a series of pCS2+ backbone-based Gateway vectors for overexpressing various tagged proteins in vertebrates. Acta Biochim Biophys Sin (Shanghai). 2016.
